# Ring-opening decarbonylative C(sp^3^)–C(sp^3^) cross-electrophile coupling of cyclic imides with unactivated alkyl chlorides

**DOI:** 10.1039/d6sc00815a

**Published:** 2026-03-11

**Authors:** Niklas J. Lentelink, Peter M. F. Pânzar, Nathalie A. V. Rowlinson, Bill Morandi

**Affiliations:** a Laboratorium für Organische Chemie ETH Zürich, Vladimir-Prelog Weg 3, HCI 8093 Zürich Switzerland morandib@ethz.ch

## Abstract

Herein we report a nickel-mediated decarbonylative cross-electrophile coupling of *N*-Boc succinimides and glutarimides with unactivated alkyl chlorides. The transformation proceeds *via* selective endocyclic N–C(O) activation, which opens a new entry point into C(sp^3^)–C(sp^3^) cross-electrophile coupling and, through incorporation of the ring-opened imide scaffold, establishes a highly modular platform to rapidly build molecular complexity. *In situ* halide exchange enables the use of abundant alkyl chlorides, while broad functional group tolerance grants access to structurally diverse α- and β-substituted amides. As a result, the method provides a new retrosynthetic disconnection to aliphatic amides, exemplified by the synthesis of densely substituted carbocyclic amides and novel capsaicin precursors. The transformation further exhibits catalytic turnover under modified conditions, demonstrating the catalytic potential of this underexplored activation mode.

## Abstract 2

Selective nickel-mediated endocyclic N–C(O) activation establishes cyclic imides as competent electrophiles in decarbonylative C(sp^3^)–C(sp^3^) cross-electrophile coupling with unactivated alkyl chlorides.

## Graphical abstract

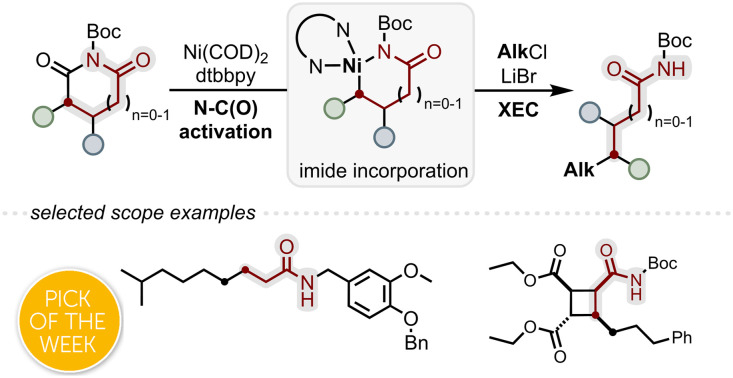

## Introduction

**Fig. 1:**
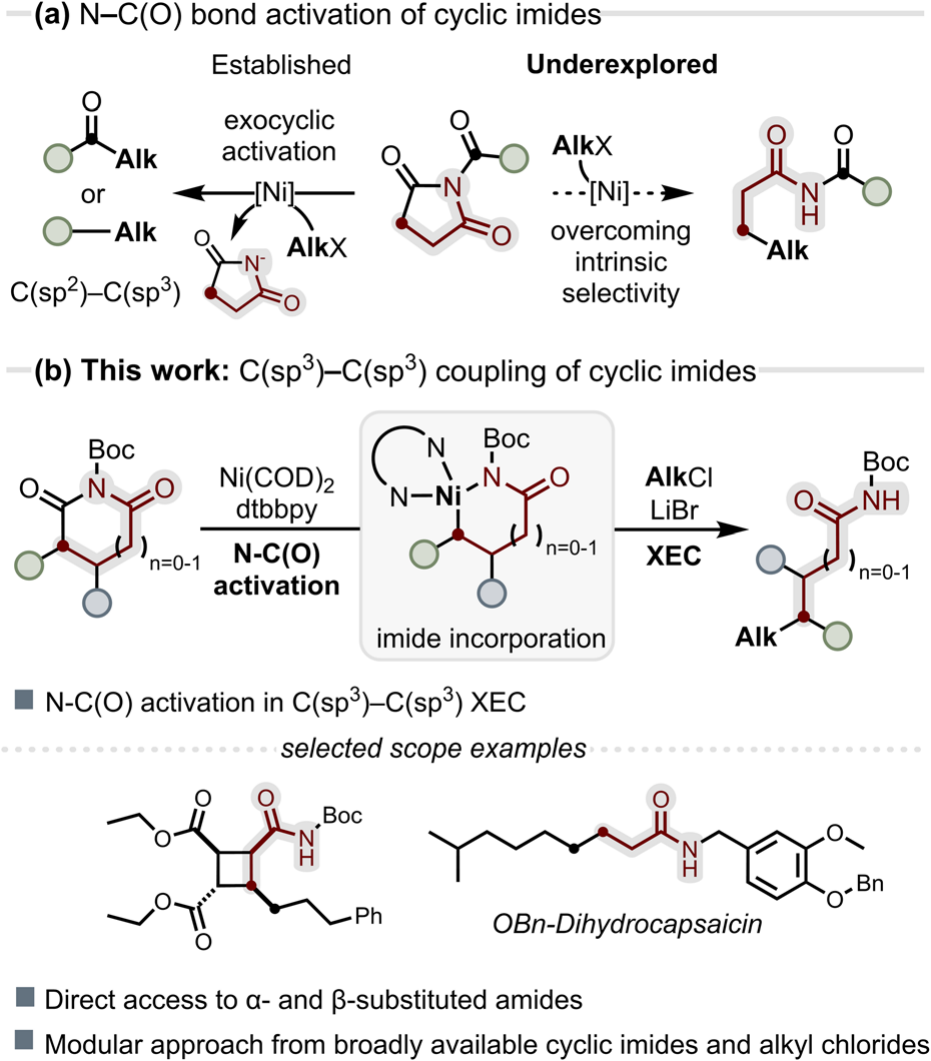
(a) Cyclic imides engage in XEC through exocyclic N–C(O) bond cleavage enabled by twisted amide reactivity, whereas endocyclic activation remains unexplored; (b) endocyclic activation of *N*-Boc cyclic imides integrates the imide scaffold into C(sp^3^)–C(sp^3^) coupling products.

Nickel-catalysed cross-electrophile coupling (XEC) has emerged as a powerful strategy for forming carbon–carbon bonds between two electrophiles under reductive conditions.^1,2^ In particular, the construction of C(sp^3^)–C(sp^3^) bonds has demonstrated the synthetic value of XEC, offering access to aliphatic scaffolds that remain challenging to construct using traditional cross-coupling methods.^3–5^ Although the number of C(sp^3^)–C(sp^3^) XEC protocols has grown over the last decade, they still rely predominantly on a narrow subset of electrophiles, most commonly alkyl halides.^6^ Beyond classical challenges, such as the desirable use of abundant yet less reactive alkyl chlorides,^7^ recent efforts have focused on diversifying the electrophile pool to address limitations in cross-coupling selectivity and availability. Electrophile classes such as allyl carbonates,^8–11^ alkyl tosylates,^12–18^ mesylates,^19–21^ (redox active) esters^22–26^ and Katritzky salts^23,27,28^ have been explored as alternatives, broadening the substrate scope in XEC chemistry. In parallel, the use of acyl electrophiles as alkylnickel precursors *via* nickel-catalysed decarbonylation has emerged as a mechanistically distinct activation strategy.^29^ While most acyl examples rely on the cleavage of O–C(O) bonds as present in anhydrides^30–33^ or 2-pyridyl esters,^34^ N–C(O) activation offers an attractive alternative route to nickel–acyl and nickel–aryl intermediates. Cyclic imides have been applied in this context through exocyclic N–C(O) bond cleavage, where their intrinsic reactivity as twisted amides enables C(sp^2^)–C(sp^3^) couplings.^35–41^ This preference for exocyclic activation stems from ground-state destabilisation of the exocyclic amide bond, facilitating its highly selective cleavage (Fig. 1a).^38^ In contrast, activation of the non-destabilised endocyclic N–C(O) bonds has remained underexplored in XEC, preventing desirable incorporation of the ring-opened, decarbonylated imide scaffold into cross-coupled products. Overcoming this intrinsic bias for exocyclic activation would enable imide scaffold incorporation and simultaneously open a new entry point into C(sp^3^)–C(sp^3^) XEC. Given that cyclic imides are readily accessible from inexpensive feedstock chemicals, this unconventional activation would further offer a versatile and modular platform for the streamlined synthesis of complex aliphatic amide scaffolds.

We previously reported a nickel-mediated *N*-Boc lactam activation for formal CO deletion.^42^ However, this reactivity did not translate into intermolecular coupling and thus offered only limited synthetic applicability. We hypothesised that the greater intrinsic electrophilicity of *N*-Boc cyclic imides would enable endocyclic N–C(O) cleavage by Ni(0) without relying on strongly σ-donating NHC ligands. Instead, we envisioned that π-accepting bipyridyl ligands could promote this activation while stabilising open-shell Ni(i)/Ni(iii) species central to radical XEC pathways, thereby unlocking previously elusive C(sp^3^)–C(sp^3^) cross-coupling reactivity (Fig. 1b).^43,44^

Herein, we describe the successful implementation of this methodology to provide a modular, decarbonylative, ring-opening cross-electrophile coupling of *N*-Boc succinimides and glutarimides with unactivated alkyl chlorides. The transformation proceeds efficiently under nickel-mediated conditions and, under modified conditions, is also amenable to a catalytic variant with synthetically applicable efficiency. Use of widely available cyclic imides and alkyl chlorides enables direct access to complex α- and β-substituted aliphatic amides, including multi-substituted carbocycles. Given the importance of amides in biologically active drug compounds, this modular strategy offers valuable building blocks with potential to accelerate early-stage discovery campaigns.^45,46^

## Results and discussion

**Fig. 2:**
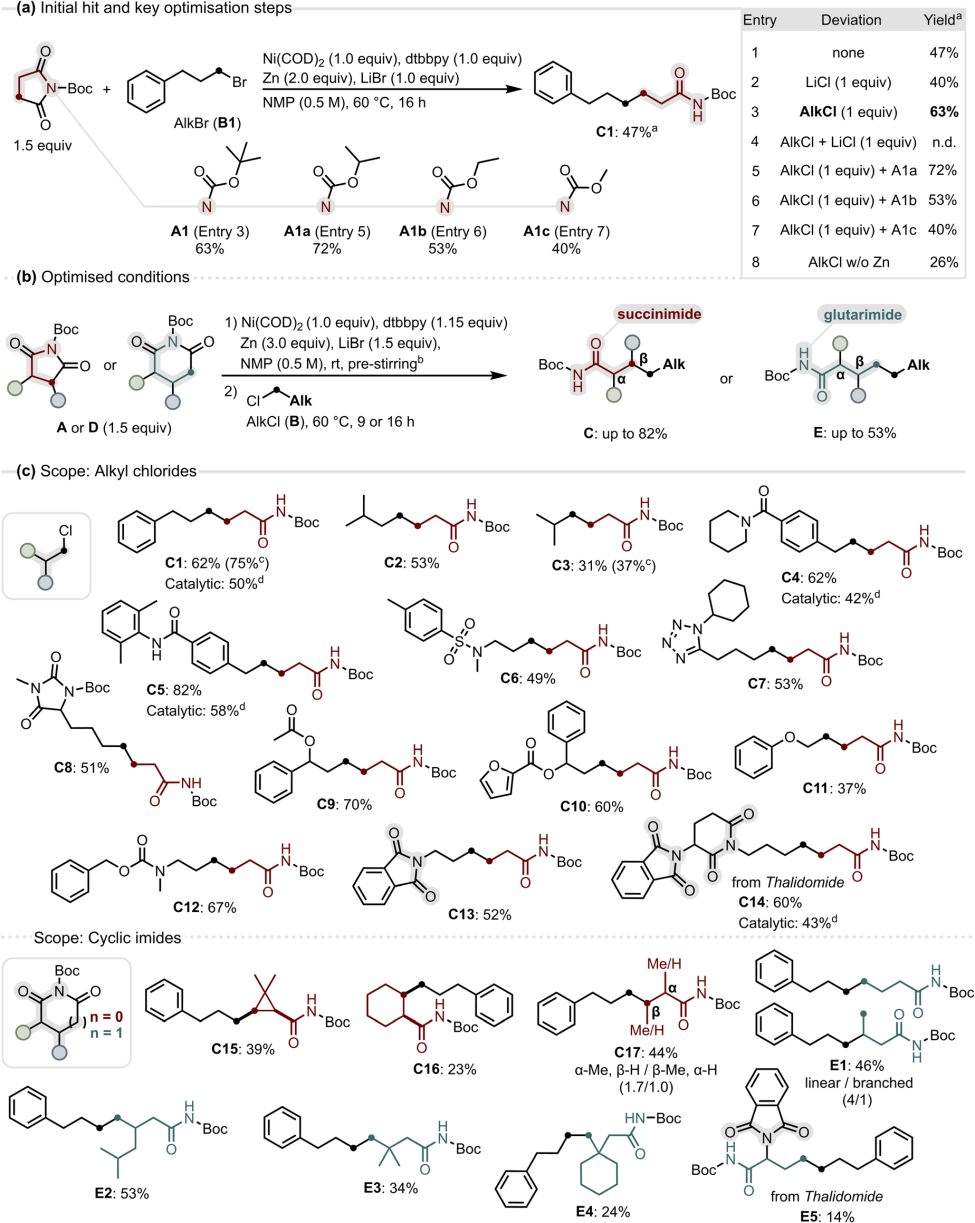
(a) Discovery and key optimisation steps; (b) optimised conditions for the XEC of *N*-Boc cyclic imides with alkyl chlorides; (c) scope of the decarbonylative XEC, reactions run on 0.2 mmol scale, yields refer to isolated products; ^*a*^LC-MS yield using 6-phenyl-1-(piperidin-1-yl)hexan-1-one as internal standard; ^*b*^pre-stirring times differ for succinimides and glutarimides, Ni(COD)_2_ and dtbbpy were pre-stirred separately (see SI, S4); ^*c*1^H-NMR yield using 1,3,5-trimethoxybenzene as internal standard; ^*d*^ catalytic conditions use 25 mol% Ni(COD)_2_ loading and an increased ligand/nickel ratio of 1.5/1.0 (see SI, S4).

We initiated our investigation by examining the reaction between *N*-Boc succinimide (A1) and (3-bromopropyl)benzene (B1) under nickel-mediated conditions using 4,4′-di-*tert*-butyl-2,2′-bipyridine (dtbbpy) as ligand (Fig. 2a, entry 1). The desired decarbonylative C(sp^3^)–C(sp^3^) XEC product was obtained in 47% yield after 16 h at 60 °C, accompanied by minor amounts of homocoupling products. Encouraged by this promising result, we next investigated the role of the halide salt additive, as such salts can tune the reduction potential of Zn^47^ and the redox properties of Ni-intermediates in organic solvents.^48^ Replacement of LiBr with LiCl led to a slightly diminished yield but resulted in rapid (<10 min) and nearly quantitative consumption of the alkyl bromide, with concurrent formation of the corresponding alkyl chloride as product formation continued (Fig. 2a, entry 2). Prompted by this observation, we tested alkyl chlorides in combination with LiBr, reasoning that *in situ* halide exchange under the reaction conditions might enable the use of more abundant but less reactive alkyl chlorides.^49^ This approach delivered the desired product in 63% yield (Fig. 2a, entry 3). We attribute the enhanced efficiency to the lower concentration of the reactive alkyl bromide, which is generated *in situ* from the alkyl chloride, the latter serving as a reservoir for controlled radical generation. Consistent with this hypothesis, no product was obtained when alkyl chlorides were used in the presence of LiCl under otherwise identical conditions (Fig. 2a, entry 4). Having established the pronounced influence of the alkyl halide identity on coupling efficiency, we next examined the impact of the carbamate protecting group. Structurally related *N*-carbamate-substituted succinimides were evaluated under the previously established conditions. All tested carbamates formed the desired decarbonylative product, with *N*-Boc (63%, A1, Fig. 2a, entry 3) and isopropyl carbamate (72%, A1a, Fig. 2a, entry 5) delivering higher yields than ethyl (53%, A1b, Fig. 2a, entry 6) and methyl carbamate (40%, A1c, Fig. 2a, entry 7). Collectively, these results highlight the advantage of sterically demanding *N*-substituents for efficient coupling, which we attribute to enhanced steric shielding of the exocyclic N–C(O) bond. Although the isopropyl carbamate A1a (Fig. 2a, entry 5) afforded a slightly higher yield than *N*-Boc (Fig. 2a, entry 3), subsequent optimization focused on *N*-Boc cyclic imides due to the widespread use of Boc protection and the facile installation and removal of the Boc group. Combining these key findings with further optimisation (see SI and S2) gave conditions suitable for both *N*-Boc succinimides and *N*-Boc glutarimides affording amides C and E*via* decarbonylative C(sp^3^)–C(sp^3^) coupling in up to 83% and 53% yield, respectively. During the optimisation studies, we observed that omitting the reductant led to a pronounced decrease in yield (Fig. 2a, entry 8), suggesting that the reductant may restore oxidised, unreactive nickel species generated *via* unproductive pathways such as exocyclic activation. This observation motivated a separate optimisation, which ultimately provided a catalytic variant of the method. In this context, we also re-examined alternative carbamate protecting groups and while several enabled product formation, none outperformed *N*-Boc activation (see SI, and S2).

With the optimised conditions in hand, we evaluated the generality of the decarbonylative XEC, first probing the scope of alkyl chlorides. To test the influence of steric effects, we examined branched substrates: remotely branched alkyl chlorides coupled in good to high yields (C1, C2), whereas an α-branched substrate, with increased steric bulk close to the coupling site, gave a moderate yield (C3).

Analysing functional group tolerance, we found that tertiary and secondary amides as well as a sulfonamide were compatible, delivering the coupled products in good to excellent yields (C4–C6). Heteroatom-rich substrates such as a tetrazole and a hydantoin derivative, coupled in good yields (C7, C8), and ester- or furan-containing substrates reacted smoothly to give ε-substituted amides (C9, C10). A β-phenoxy alkyl chloride also gave the desired product, albeit in moderate yield, showing that β-heteroatom substitution can be tolerated (C11). A more sensitive benzyl carbamate group (Cbz) also remained untouched, allowing orthogonal protection of amines under the reaction conditions (C12). To our delight, *N*-Boc imides underwent selective activation in the presence of other cyclic imides, allowing chemoselective coupling in substrates containing phthalimide (C13) and in a thalidomide derivative with both glutarimide and phthalimide groups (C14). With the nickel-mediated alkyl chloride scope defined, we turned to probing the catalytic potential of the transformation. Under modified conditions with 25% nickel loading, products C1, C4, C5, and C14 were obtained in synthetically useful yields (50, 42, 58, 43%), demonstrating that catalytic turnover is feasible.

Having explored the alkyl chloride scope, we next examined a variety of cyclic imides under nickel-mediated conditions. To our satisfaction, their coupling proved less sensitive to steric effects than for alkyl chlorides, and even α-substituted imides were competent substrates. This enabled the use of symmetrical bicyclic succinimides as substrates, yielding 1,2-disubstituted cyclopropyl- and cyclohexyl amides in moderate yields (C15, C16). The formation of the *cis*-configured cyclopropyl amide C15 indicates that Ni-mediated activation of the cyclic imide proceeds diastereoretentively, preserving the relative stereochemistry of the imide in the amide product. A monosubstituted succinimide gave a mixture of α- and β-substituted amides, favouring α-substitution (C17) by activation of the more accessible N–C(O) bond. We then evaluated differently substituted *N*-Boc glutarimides. Surprisingly, unsubstituted *N*-Boc glutarimide afforded a mixture of a linear and a branched amide, with the linear product predominating (E1). The formation of the branched product may arise from nickel chain walking occurring after activation of the glutarimide.^50–52^ Introduction of a 4-substituent effectively prevented product branching and enabled selective formation of the expected linear amide in a yield comparable to succinimides (E2). By comparison, sterically demanding 4,4-disubstitution reduced efficiency but nevertheless afforded access to β,β-disubstituted amides, including a 1,1-disubstituted cyclohexyl amide (E3, E4). Further highlighting the importance of *N*-Boc substitution for imide activation, reaction of *N*-Boc thalidomide afforded the α-amino acid precursor E5 in synthetically useful, albeit modest, yield. By contrast, we had observed that the glutarimide moiety in thalidomide remained unreactive when *N*-alkyl substituted (C14).

**Fig. 3:**
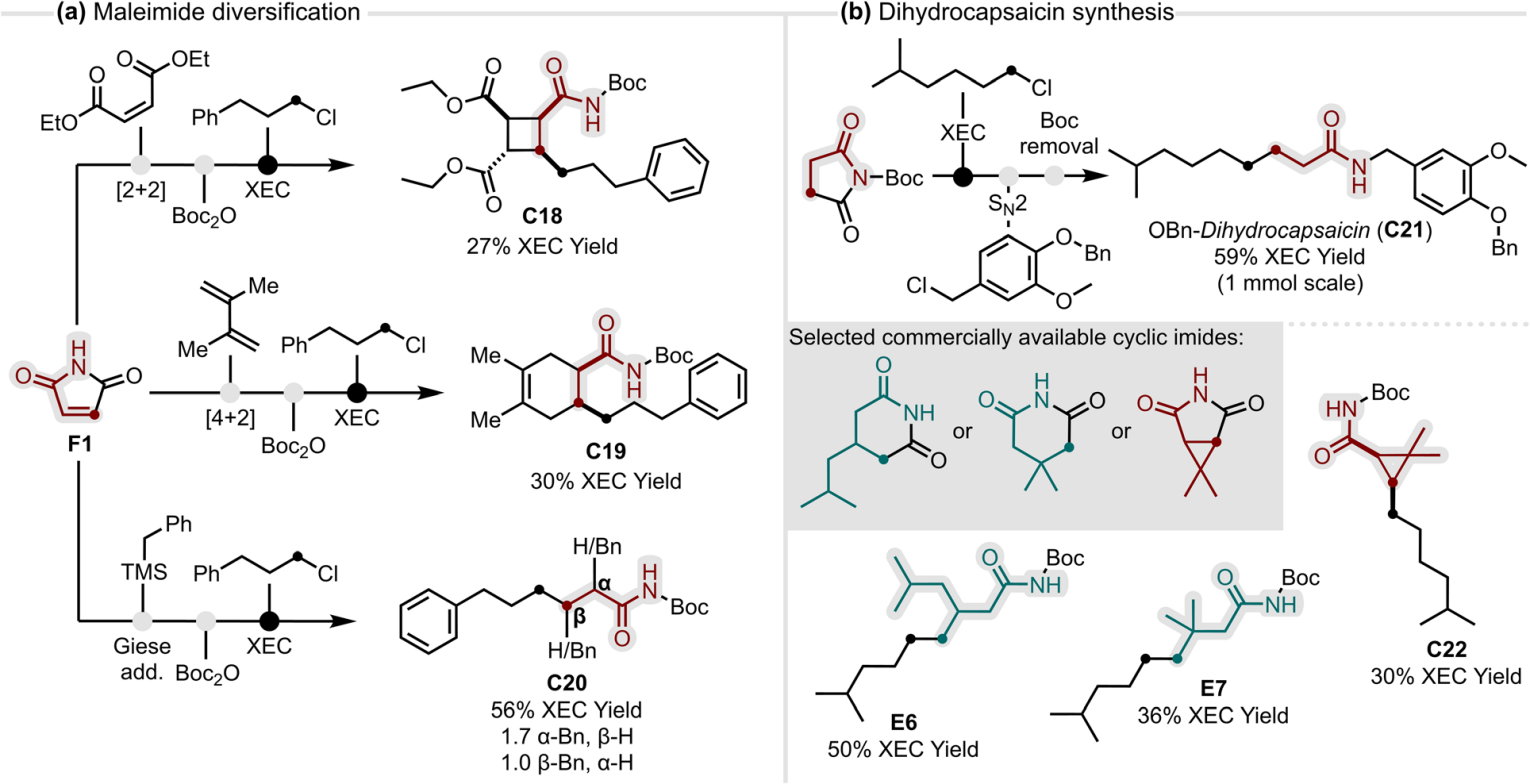
(a) Modular diversification of maleimide into densely substituted carbocycles and linear amides *via* decarbonylative XEC; (b) modular XEC approach to dihydrocapsaicin and unnatural capsaicin precursors from commercially available building blocks.

Building on the demonstrated generality of our method, we sought to further highlight the modularity of the XEC platform, emphasising its potential to generate molecular complexity from simple and inexpensive building blocks. To this end, we envisioned two complementary case studies. The first showcases feedstock maleimide F1, as a versatile building block whose broad reactivity profile enables straightforward diversification into distinct carbocyclic and linear amide scaffolds (Fig. 3a). Representative sequences include a [2 + 2] cycloaddition leading to a highly substituted cyclobutane C18, a Diels–Alder reaction giving 1,2,4,5-tetrasubstituted cyclohexene amide C19, and a Giese addition of benzylic radicals affording α- and β-substituted amides C20 as a mixture of regioisomers. Crucially, positioning the XEC as the final step is enabled by the robustness of the method, allowing even complex imide precursors to be transformed into the corresponding amide scaffolds.

In contrast to the maleimide case study, which maximised diversity from a single feedstock, we next explored the feasibility of our method for the targeted construction of biologically active scaffolds (Fig. 3b). Starting from a small pool of commercially available cyclic imides and alkyl chlorides, the dihydrocapsaicin scaffold was rapidly assembled with focused α- and β-modifications. The *N*-Boc imide activation proved particularly valuable, as its facile downstream removal enabled direct access to benzyl-protected dihydrocapsaicin C21 in three steps, underscoring the practicality of the chosen sequence. The key XEC step was performed on 1 mmol scale (five times scale-up) without loss of efficiency, further highlighting the robustness of the conditions. With the sequence established, three dihydrocapsaicin precursors were prepared in a single XEC step, delivering a β-isobutyl amide E6, the β,β-dimethyl derivative E7 and a dimethylcyclopropyl amide C22 in synthetically useful yields.

**Fig. 4:**
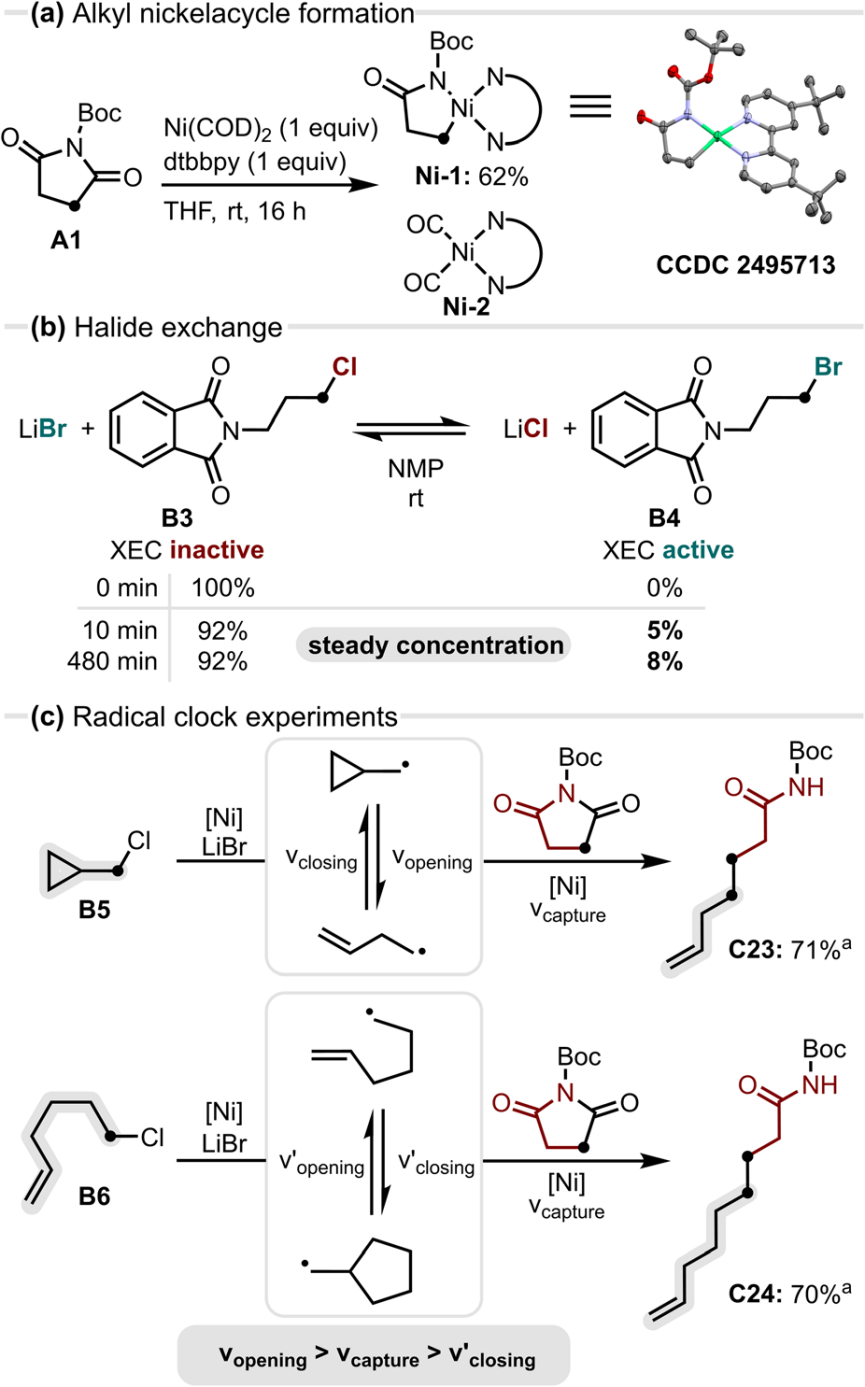
(a) Formation of an alkyl nickelacycle *via* endocyclic N–C(O) activation; (b) *in situ* halide exchange converting XEC-inactive alkyl chlorides into active alkyl bromides; (c) radical clock experiments confirming alkyl radical involvement and estimation of the capture timescale; ^*a*1^H-NMR yield using internal standard 1,3,5-trimethoxybenzene.

To gain insight into the underlying reactivity, we finally examined the key steps of the transformation (Fig. 4). Endocyclic N–C(O) activation of *N*-Boc succinimide by Ni(0) at room temperature gave both an alkyl nickelacycle Ni-1 and a Ni–CO complex Ni-2, with crystal structures confirming their identity (See SI, S6 + S8). Formation of the Ni–CO complex Ni-2 suggests catalyst poisoning and may contribute to the reduced efficiency observed in the catalytic variant. Having confirmed the endocyclic N–C(O) activation of succinimide A1, the observed *in situ* halide exchange was examined by stirring 3-chloro-*N*-phthaloylpropylamine (B3) in the presence of LiBr, resulting in approximately 5% conversion to the corresponding alkyl bromide B4 within 10 min, with no significant increase after 8 h at rt (see SI, S6). Finally, radical clock experiments were conducted to probe the involvement of alkyl radicals. The cyclopropyl clock B5 opened cleanly to give the terminal alkene C23, while the slower hexenyl clock B6 did not cyclise before radical capture by nickel, again leading to a terminal alkene C24. These results indicate the formation of alkyl radicals, with radical capture occurring on a timescale between cyclopropyl ring opening and hexenyl cyclisation, potentially involving the alkyl nickelacycle Ni-1 (for a mechanistic proposal see SI, S6.4).

## Conclusions

In conclusion, we have developed a nickel-mediated decarbonylative cross-electrophile coupling of *N*-Boc succinimides and glutarimides with unactivated alkyl chlorides through selective endocyclic N–C(O) activation. This activation mode enables incorporation of the imide backbone into the amide products, creating a modular platform for the streamlined assembly of complex aliphatic scaffolds. The method exploits readily available alkyl chlorides and cyclic imides while displaying broad functional group tolerance, boding well for immediate application in the synthesis of valuable amide building blocks.

## Author contributions

N. J. L. conceived the project. N. J. L., P. M. F. P. and N. A. V. R. conducted the experimental work and analysed the data. B. M. supervised the research. N. J. L. and B. M. wrote the manuscript with input from all authors.

## Conflicts of interest

There are no conflicts to declare.

## Supplementary Material

SC-OLF-D6SC00815A-s001

SC-OLF-D6SC00815A-s002

## Data Availability

The supporting data has been provided as part of the supplementary information (SI). Supplementary information: synthetic procedures, NMR spectra and further experimental details. NMR raw data supporting the characterization of the reported compounds are available at Zenodo (DOI: https://doi.org/10.5281/zenodo.18837224.) See DOI: https://doi.org/10.1039/d6sc00815a. CCDC 2495712 and 2495713 contain the supplementary crystallographic data for this paper.^70*a*,*b*^ The authors have cited additional references within the SI.^53–69^
