# Supplementary material for: Ring-opening decarbonylative C(sp3)–C(sp3) cross-electrophile coupling of cyclic imides with unactivated alkyl chlorides
Source: Chem Sci. 2026 Mar 11;17(17):8479–85. doi: 10.1039/d6sc00815a (PMC12977082; doi:10.1039/d6sc00815a)
Supplement: SC-017-D6SC00815A-s001 [file SC-017-D6SC00815A-s001.pdf]

Supplementary Information (SI) for Chemical Science.
This journal is © The Royal Society of Chemistry 2026

*Supplementary Information*

# Ring-Opening Decarbonylative C(sp$^3$)–C(sp$^3$) Cross-Electrophile Coupling of Cyclic Imides with Unactivated Alkyl Chlorides

Niklas J. Lentelink[1], Peter M. F. Pânzar[1], Nathalie A. V. Rowlinson[1] and Bill Morandi[1]*

[1]Laboratorium für Organische Chemie, ETH Zürich, 8093 Zürich, Switzerland

*Corresponding author, Email: morandib@ethz.ch

**Table of Contents**

1. General Information ........ 3

2. Reaction Optimization ........ 4

2.1 Optimization of nickel-mediated reaction conditions ........ 4

2.1.1 Evaluation of different *N*-substituents ........ 5

2.2 Optimization of nickel-catalyzed reaction conditions ........ 6

2.2.1 Influence of nickel loading ........ 6

2.2.2 Influence of ligands ........ 7

2.2.3 Influence of halide salts ........ 8

2.2.4 Influence of coordinating additives and solvents ........ 9

2.2.5 Influence of inverse stoichiometry ........ 9

2.2.6 Influence of addition procedure ........ 10

2.2.7 Evaluation of different *N*-substituents ........ 10

2.3 Commonly observed side products ........ 11

3. Synthesis and characterization of starting materials ........ 12

3.1 *N*-Boc cyclic imides ........ 12

3.2 Alkyl chlorides ........ 15

3.3 Alternative *N*-carbamate succinimides ........ 20

4. Synthesis and characterization of cross-coupled products ........ 22

4.1 Additional Substrates ........ 34

4.2 Failed Substrates ........ 35

5. Case studies ........ 36

5.1 Maleimide diversification: Synthesis and characterization of cyclic imides ........ 36

5.2 Maleimide diversification: Synthesis and characterization of cross-coupled products ........ 38

5.3 Capsaicin precursors: Synthesis and characterization of cross-coupled products ........ 40

5.4 Capsaicin: Synthesis and characterization of OBn-dihydrocapsaicin ........ 41

6. Mechanistic studies ........ 42

6.1 Synthesis and characterization of nickel complexes ........ 42

6.2 Halide exchange studies ........ 43

6.3 Radical clock studies ........ 44

6.4 Mechanistic Proposal ........ 45

7. NMR spectra ........ 46

8. XRD Data ........ 111

8.1 X-ray structure parameters of nickel complexes ........ 111

8.2 ORTEP plots of X-ray structures and solid-state parameters ........ 112

9. References ........ 114

## 1. General Information

Air- and moisture-sensitive manipulations were performed in an MBraun glovebox under argon. Glassware was oven-dried and stored warm until use. Solvents for sensitive operations were obtained in Acros SureSeal bottles and used as received. Bis(1,5-cyclooctadiene)nickel(0) (Strem) and 4,4'-di-tert-butyl-2,2'-dipyridyl (Fluorochem) were used as received. Unless otherwise specified, reagents for substrate synthesis were purchased from commercial suppliers and used without further purification.

NMR spectra were recorded on Bruker Avance III 400 MHz, Bruker Neo 400 MHz, or Bruker Avance III 500 MHz spectrometers equipped with BBFO probes at the NMR facility of ETH Zürich. $^{1}H$ chemical shifts were referenced to residual non-deuterated solvent signals, and $^{13}C$ spectra were referenced to the corresponding deuterated solvent signals. Coupling constants (J) are reported in Hz. Multiplicities are denoted as s (singlet), d (doublet), t (triplet), q (quartet), p (pentet), hept (heptet), m (multiplet), br (broad), app (apparent), and combinations thereof. High-resolution mass spectra (HRMS) were obtained at the mass spectrometry service of the Laboratories of Organic Chemistry, ETH Zürich.

LC-MS measurements were performed on an Agilent 1260 Infinity II system equipped with a DAD HS diode array detector (UV-VIS), an ELSD, and an InfinityLab LC/MSD iQ single-quadrupole mass detector (ESI mode), using a YMC-PACK ODS-AQ column (250 x 4.6 mm, 5 um, 120 A).

Analytical thin-layer chromatography (TLC) was carried out on Merck silica gel 60 F254 plates (0.25 mm). Spots were visualized by UV light (λ = 254 nm) or by staining with $KMnO_4$. Flash column chromatography was performed on a Biotage Isolera One using self-packed silica gel cartridges (SiliaFlash P60, 40–63 μm, Silicycle) with technical-grade solvents.

Single crystalline samples were measured on a Rigaku Oxford Diffraction XtaLAB Synergy-R kappa diffractometer equipped with a Rigaku HyPix Arc150 HPAD detector and using microfocus rotating anode Cu-Kα radiation with mirror optics (λ = 1.54178 Å). All measurements were carried out at 100K using an Oxford Cryosystems Cryostream 800 sample cryostat. Data were integrated using CrysAlisPro and corrected for absorption effects using a combination of empirical (ABSPACK) and numerical corrections.[53] The structures were solved using SHELXT2[54] and refined by full-matrix least-squares analysis (SHELXL)[55,56] using the program package OLEX2.[57] Non-hydrogen atoms were refined anisotropically and hydrogen atoms were constrained to ideal geometries and refined with fixed isotropic displacement parameters (in terms of a riding model). Supplementary crystallographic data for this paper, including structure factors and refinement instructions, can be obtained free of charge from The Cambridge Crystallographic Data Centre, 12 Union Road, Cambridge CB2 1EZ, UK (fax: +44(1223)-336-033; e-mail: deposit@ccdc.cam.ac.uk), or via https://www.ccdc.cam.ac.uk/structures.

Safety note: Although formation of the volatile and toxic species $Ni(CO)_4$ is highly unlikely under the reported conditions and scale, caution is required during workup outside the glovebox. All operations must be carried out in well-ventilated fume hoods.

# 2. Reaction Optimization

## 2.1 Optimization of nickel-mediated reaction conditions

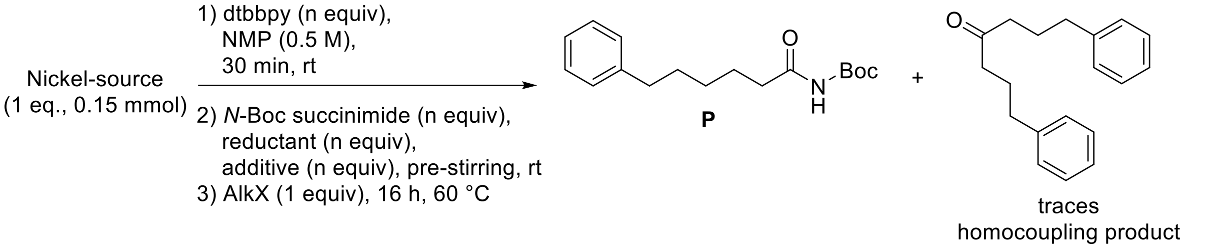


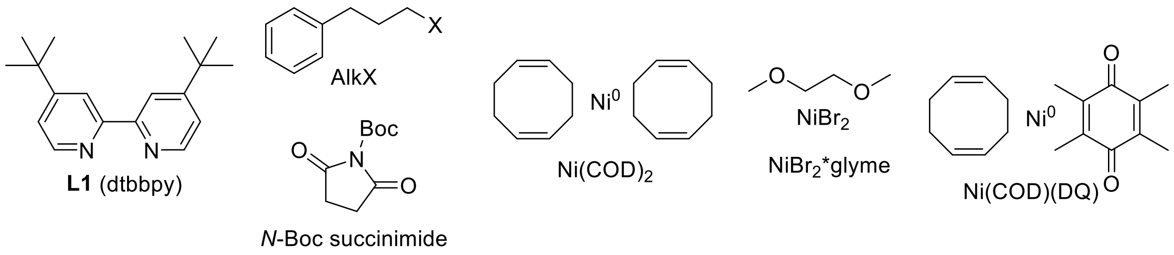


***Supplementary Table 1. Optimization of nickel-mediated reaction conditions. Yields were determined by calibrated LC-MS measurements with internal standard 6-phenyl-1-(piperidin-1-yl)hexan-1-one.***

| Entry | Ni | L1 eq. | Imide eq. | Red. | Red. eq. | Add. | Add. eq. | Pre-stirring [min] | AlkX | Yield P [%] |
|---|---|---|---|---|---|---|---|---|---|---|
| initial | $Ni(COD)_2$ | 1 | 2 | Zn | 2 | LiBr | 1 | 45 | AlkBr | 47 |
| 1 | $Ni(COD)_2$ | 1 | 2 | Zn | 2 | LiCl | 1 | 45 | AlkBr | 40 |
| 2 | $Ni(COD)_2$ | 1 | 2 | Zn | 2 | LiBr | 1 | 45 | AlkCl | 63 |
| 3 | $Ni(COD)_2$ | 1 | 2 | Zn | 2 | LiCl | 1 | 45 | AlkCl | n.d. |
| 4 | $Ni(COD)_2$ | 1 | 2 | none | none | LiBr | 1 | 45 | AlkBr | 27 |
| 5 | $Ni(COD)_2$ | 1 | 2 | none | none | LiBr | 1 | 45 | AlkCl | 26 |
| 6 | $NiBr_2$*glyme | 1.25 | 2 | Zn | 3 | LiBr | 1 | none | AlkCl | traces |
| 7 | $NiBr_2$*glyme | 1.25 | 2 | Mn | 3 | LiBr | 1 | none | AlkCl | 18 |
| 8 | Ni(COD)(DQ) | 1.25 | 2 | Zn | 3 | LiBr | 1 | none | AlkCl | 8 |
| 9 | $NiBr_2$*glyme | 1.25 | 2 | Zn | 3 | LiBr | 1 | 15 | AlkCl | traces |
| 10 | $NiBr_2$*glyme | 1.25 | 2 | Mn | 3 | LiBr | 1 | 15 | AlkCl | 15 |
| **final** | **$Ni(COD)_2$** | **1.15** | **1.5** | **Zn** | **3** | **LiBr** | **1.5** | **45** | **AlkCl** | **75** |

Optimization of nickel-mediated and nickel-catalyzed conditions was conducted partly in parallel, with results from each campaign feeding back into the other. This non-linear interplay allowed advances in one system to immediately inform the optimization strategy of the other. Importance of ligand identity, additives, stoichiometry and order of reagent addition were evaluated during optimization of the catalytic conditions (see Section 2.2).

### 2.1.1 Evaluation of different *N*-substituents

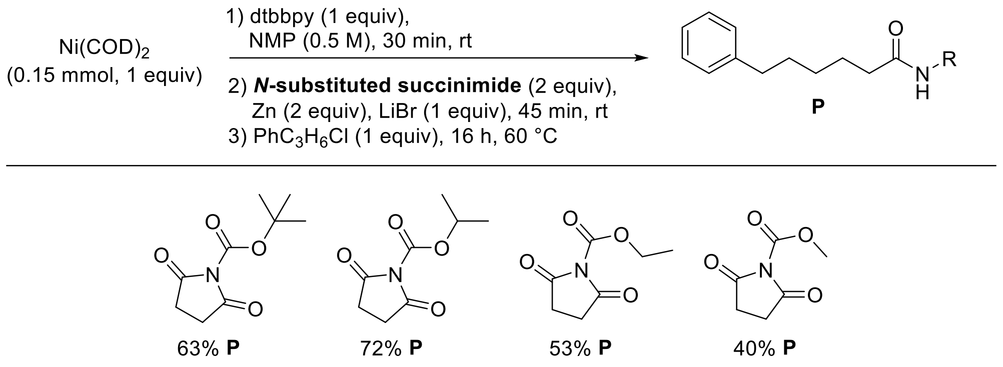


***Supplementary Figure 1. Influence of N-substitution of cyclic imides on coupling performance. Yields were determined by calibrated LC-MS measurements with internal standard 6-phenyl-1-(piperidin-1-yl)hexan-1-one.***

## 2.2 Optimization of nickel-catalyzed reaction conditions

### 2.2.1 Influence of nickel loading

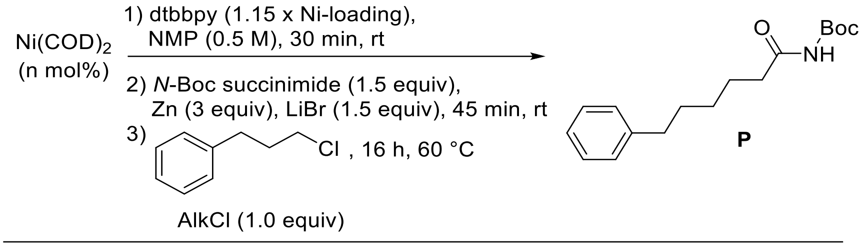


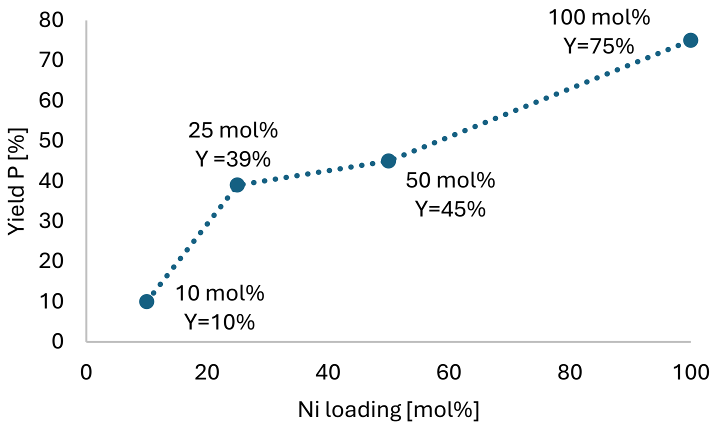


***Supplementary Figure 2. Influence of nickel loading on yield. Yields were determined by calibrated LC-MS measurements with internal standard 6-phenyl-1-(piperidin-1-yl)hexan-1-one.***

### 2.2.2 Influence of ligands

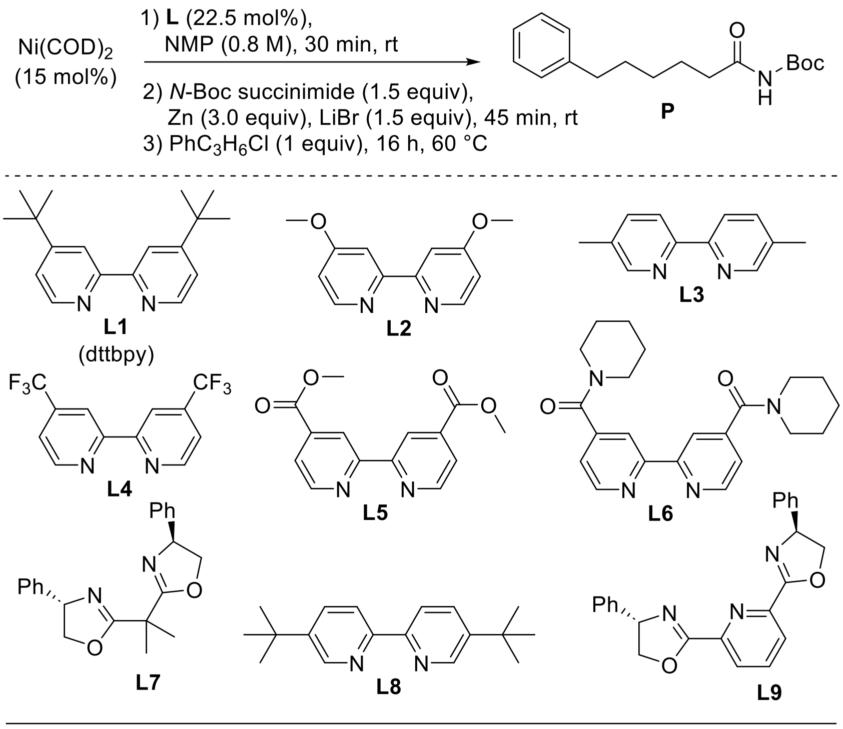


***Supplementary Table 2. Influence of ligands. Yields were determined by calibrated LC-MS measurements with internal standard 6-phenyl-1-(piperidin-1-yl)hexan-1-one.***

| Entry | Ligand | Yield P [%] |
|---|---|---|
| 1 | **L1** | 29 |
| 2 | **L2** | 19 |
| 3 | **L3** | 22 |
| 4 | **L4** | 1 |
| 5 | **L5** | 5 |
| 6 | **L6** | 13 |
| 7 | **L7** | 0 |
| 8 | **L8** | 22 |
| 9 | **L9** | 0 |

### 2.2.3 Influence of halide salts

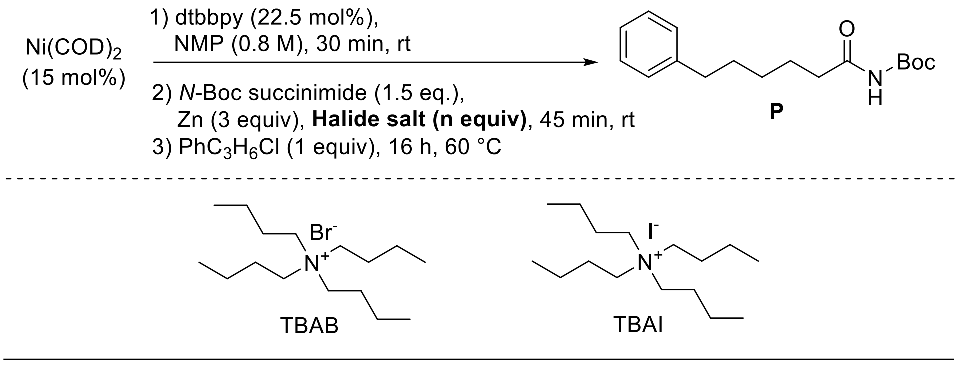


***Supplementary Table 3. Influence of halide salts. Yields were determined by calibrated LC-MS measurements with internal standard 6-phenyl-1-(piperidin-1-yl)hexan-1-one.***

| Entry | Halide salt | Equiv. | Yield P [%] |
|---|---|---|---|
| 1 | **LiCl** | 0.75 | n.d. |
| 2 | **LiBr** | 0.25 | 21 |
| 3 | **LiBr** | 0.75 | 27 |
| 4 | **LiBr** | 1.5 | 29 |
| 5 | **LiBr** | 2 | 20 |
| 6 | **LiBr** | 4 | n.d. |
| 7 | **LiBr + FeBr$_3$** | 1.0 + 0.2 | 17 |
| 8 | **TBAB** | 0.75 | 25 |
| 9 | **TBAB** | 2 | 33 |
| 10 | **MgBr2** | 1.5 | n.d. |
| 11 | **LiI** | 0.75 | 26 |
| 12 | **NaI** | 1 | 31 |
| 13 | **TBAI** | 1.25 | 31 |

## 2.2.4 Influence of coordinating additives and solvents

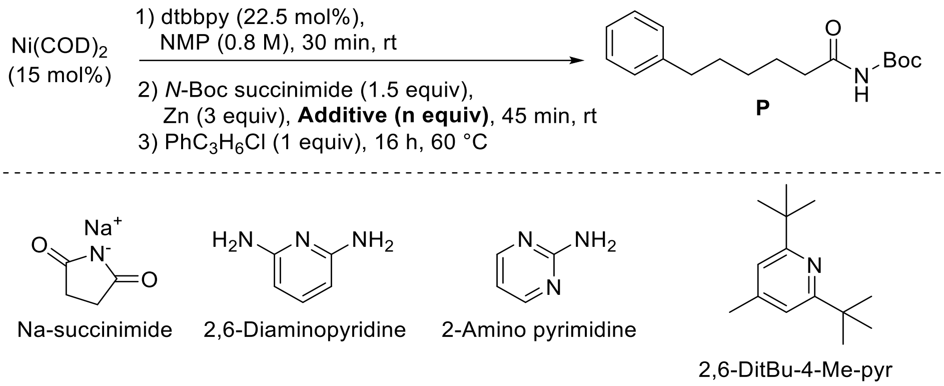


***Supplementary Table 4. Influence of coordinating additives and solvents. Yields were determined by calibrated LC-MS measurements with internal standard 6-phenyl-1-(piperidin-1-yl)hexan-1-one.***

| Entry | Additive | Equiv. | Solvent | Yield P [%] |
|---|---|---|---|---|
| 1 | LiBr | 1.5 | NMP/MeCN (1:1) | 22 |
| 2 | LiBr + Succinimide | 1.5 + 0.25 | NMP | 34 |
| 3 | LiBr + Na-Succinimide | 1.5 + 0.25 | NMP/Dioxane (1:1) | 31 |
| 4 | LiBr + 2,6-Diaminopyridine | 1.5 + 0.25 | NMP | 36 |
| 5 | LiBr + 2,6-Diaminopyridine | 1.5 + 0.5 | NMP | 36 |
| 6 | LiBr + Pyridine | 1.5 + 0.25 | NMP | 27 |
| 7 | LiBr + 2-Amino pyrimidine | 1.5 + 0.25 | NMP | 29 |
| 8 | LiBr + 2,6-DitBu-4-Me-Pyr | 1.5 + 0.25 | NMP | 27 |

## 2.2.5 Influence of inverse stoichiometry

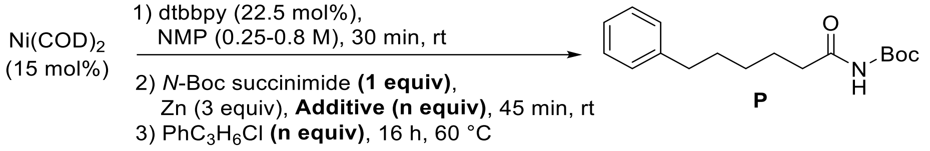


***Supplementary Table 5. Influence of inverse stoichiometry. Yields were determined by calibrated LC-MS measurements with internal standard 6-phenyl-1-(piperidin-1-yl)hexan-1-one.***

| Entry | AlkCl Equiv. | Additive | Equiv. | conc. [M] | Yield P [%] |
|---|---|---|---|---|---|
| 1 | 2.5 | LiBr | 1.5 | 0.8 | 17 |
| 2 | 2.5 | TBAB | 1.5 | 0.8 | 28 |
| 3 | 2.5 | LiI | 0.75 | 0.8 | 32 |
| 4 | 2.5 | LiBr | 1 | 0.25 | 35 |
| 5 | 2.5 | TBAI | 0.5 | 0.8 | 13 |
| 6 | 2.5 | TBAI | 0.25 | 0.8 | 20 |

### 2.2.6 Influence of addition procedure

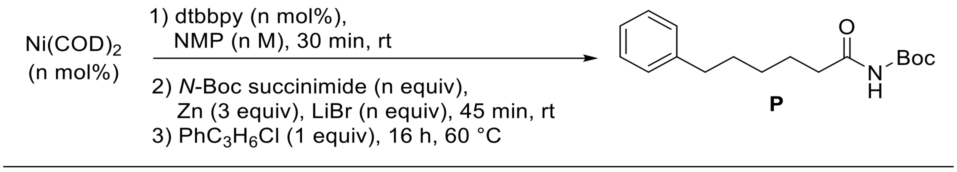


***Supplementary Table 6. Influence of addition procedure. Yields were determined by calibrated LC-MS measurements with internal standard 6-phenyl-1-(piperidin-1-yl)hexan-1-one.***

| Entry | Comment | Imide equiv. | LiBr equiv. | conc. [M] | Yield P [%] |
|---|---|---|---|---|---|
| 1 | 2 batches of 10% $Ni(COD)_2$ + 15% dtbbpy, added 6 h apart | 2 | 1 | 0.5 | 28 |
| 2 | 2 batches of 10% $Ni(COD)_2$ + 15% dtbbpy, added 2 h apart | 1.5 | 1.5 | 0.6 | 29 |
| 3 | 2 batches of 10% $Ni(COD)_2$ + 15% dtbbpy | | | | |
| | Second batch added in NMP over 10 h by syringe pump | 2 | 1 | 0.5 | 29 |
| 4 | $Ni(COD)_2$ 20% + dtbbpy 30% added over 4 h by syringe pump in NMP | 2 | 1.5 | 0.6 | 14 |
| 5 | AlkCl added over 4 h in NMP | 1.5 | 1.5 | 0.6 | 19 |
| 6 | AlkCl added over 1 h in NMP | 1.5 | 1.5 | 0.6 | 19 |

### 2.2.7 Evaluation of different *N*-substituents

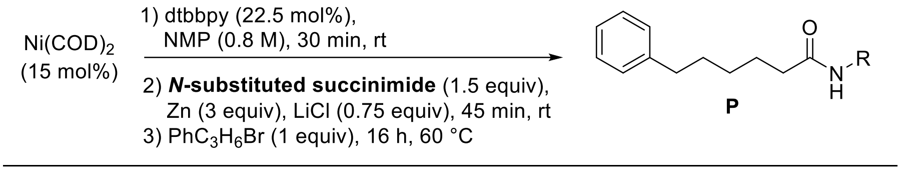


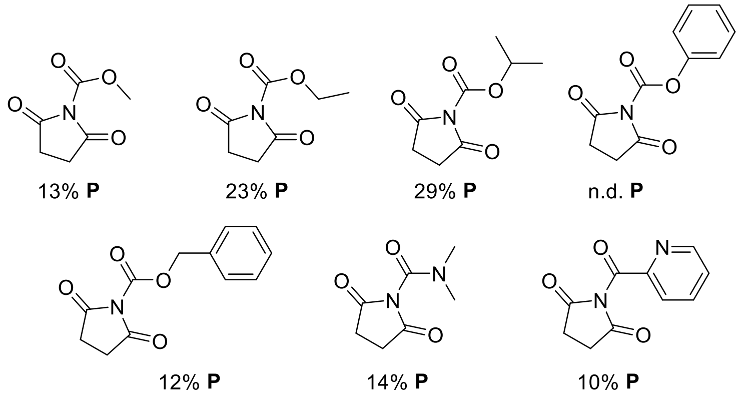


***Supplementary Figure 3. Influence of N-substitution of cyclic imides on coupling performance. Yields were determined by calibrated LC-MS measurements with internal standard 6-phenyl-1-(piperidin-1-yl)hexan-1-one.***

## 2.3 Commonly observed side products

**Alkyl chloride derived side product:**

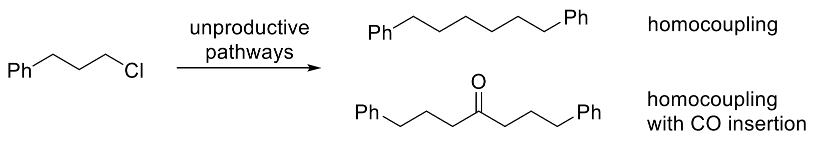


***N*-Boc imide derived side product:**

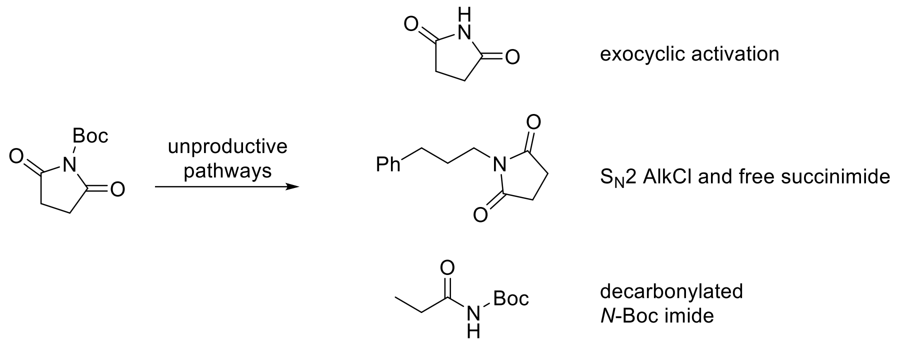


***Supplementary Figure 4. Commonly observed side products in the coupling reaction between alkyl chlorides and N-Boc cyclic imides. Representative examples shown here were analyzed by LC-MS and $^{1}$H-NMR spectroscopy.***

## 3. Synthesis and characterization of starting materials

### 3.1 *N*-Boc cyclic imides

**General procedure for the preparation of *N*-Boc cyclic imides (GP1):**

To a solution of the free cyclic imide (1.0 equiv) and 4-dimethylaminopyridine (DMAP, 0.05 equiv) in 1,4-dioxane (0.35 M) was added dropwise di-tert-butyl dicarbonate ($Boc_2O$, 1.3 equiv) in 1,4-dioxane (~3.5 M) over 15 min at rt. The mixture was stirred until completion was indicated by thin-layer-chromatography (typically < 1 h), then concentrated *in vacuo*. The residue was dissolved in EtOAc, washed with water and brine, dried over $MgSO_4$, filtered, and concentrated *in vacuo*. The crude product was purified by recrystallization or column chromatography.

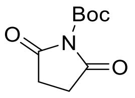


***N*-Boc succinimide (A1):** Was prepared from succinimide (10.0 g, 100.9 mmol, 1 eq.), DMAP (616.5 mg, 5.05 mmol, 0.05 eq.) and $Boc_2O$ (28.63 g, 131.2 mmol, 1.3 eq.) according to GP1. Purification by recrystallization from *n*-hexane and DCM, afforded the product (17.0 g, 83.0 mmol, 82%) as a colorless, crystalline solid. The NMR data are in accordance with those reported.[58]

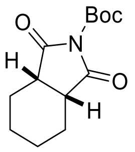


***N*-Boc hexahydrophthalimide (A2):** Was prepared from hexahydrophthalimide (600 mg, 3.92 mmol, 1 eq.), DMAP (23.9 mg, 0.20 mmol, 0.05 eq.) and $Boc_2O$ (1.11 g, 5.09 mmol, 1.3 eq.) according to GP1. Purification by recrystallization from *n*-hexane and DCM, afforded the product (490 mg, 1.90 mmol, 48%) as a colorless, crystalline solid. The NMR data are in accordance with those reported.[59]

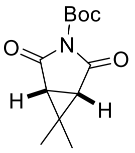


***N*-Boc 6,6-dimethyl-3-azabicyclo[3.1.0]hexane-2,4-dione (A3):** Was prepared from 6,6-dimethyl-3-azabicyclo[3.1.0]hexane-2,4-dione (1.50 g, 10.8 mmol, 1 eq.), DMAP (65.8 mg, 0.54 mmol, 0.05 eq.) and $Boc_2O$ (3.06 g, 14.0 mmol, 1.3 eq.) according to GP1. Purification by recrystallization from *n*-hexane and acetone, afforded the product (1.78 g, 7.47 mmol, 69%) as a colorless, crystalline solid.

$^1$H NMR (400 MHz, CDCl3) δ 2.36 (s, 2H), 1.55 (s, 9H), 1.35 (s, 3H), 1.26 (s, 3H).

$^{13}C$ NMR (101 MHz, $CDCl_3$) δ 169.8, 146.3, 86.0, 34.3, 33.9, 27.9, 26.4, 16.0.

HRMS (ESI) m/z calcd. for $C_{12}H_{17}NNaO_4$ ($[M+Na]^+$): 262.1050, found: 262.1048.

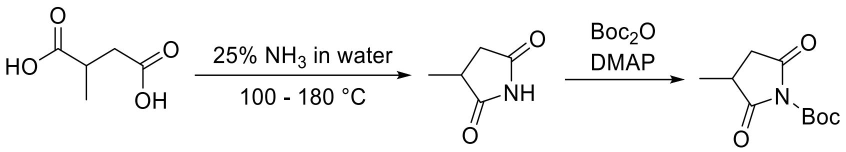


***N*-Boc 3-methylpyrrolidine-2,5-dione (A4):** The free imide was prepared according to a literature procedure.[60] The *N*-Boc imide was prepared from 3-methylpyrrolidine-2,5-dione (5.70 g, 50.4 mmol, 1 eq.), DMAP (308 mg, 2.52 mmol, 0.05 eq.) and $Boc_2O$ (14.3 g, 65.5 mmol, 1.3 eq.) according to GP1. Purification by column chromatography (10-100% EtOAc in *n*-hexane) afforded the product (7.30 g, 34.2 mmol, 68%) as a colorless, waxy solid.

$^{1}H$ NMR (400 MHz, CDCl3) δ 3.02 – 2.83 (m, 2H), 2.47 – 2.30 (m, 1H), 1.56 (s, 9H), 1.39 – 1.33 (m, 3H).

$^{13}C$ NMR (101 MHz, $CDCl_3$) δ 176.4, 172.2, 146.7, 86.3, 36.9, 35.2, 27.9, 16.5.

HRMS (ESI) m/z calcd. for $C_{10}H_{15}NNaO_4$ ($[M+Na]^+$): 236.0893, found: 236.0890.

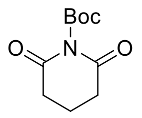


***N*-Boc glutarimide (D1):** Was prepared from glutarimide (500 mg, 4.42 mmol, 1 eq.), DMAP (27.0 mg, 0.22 mmol, 0.05 eq.) and $Boc_2O$ (1.25 g, 5.75 mmol, 1.3 eq.) according to GP1. Purification by column chromatography (12-80% EtOAc in *n*-hexane) afforded the product (730 mg, 3.40 mmol, 77%) as a light-yellow solid. The NMR data are in accordance with those reported.[61]

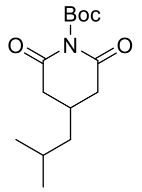


***N*-Boc 4-isobutylpiperidine-2,6-dione (D2):** Was prepared from 4-isobutylpiperidine-2,6-dione (1.83 g, 10.8 mmol, 1 eq.), DMAP (66.0 mg, 0.54 mmol, 0.05 eq.) and $Boc_2O$ (3.06 g, 14.0 mmol, 1.3 eq.) according to GP1. Purification by column chromatography (5-40% EtOAc in *n*-hexane) afforded the product (2.14 g, 7.95 mmol, 74%) as a colorless solid. The NMR data are in accordance with those reported.[62]

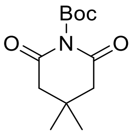


***N*-Boc 4,4-dimethylpiperidine-2,6-dione (D3):** Was prepared from 4,4-dimethylpiperidine-2,6-dione (1.25 g, 8.84 mmol, 1 eq.), DMAP (54.0 mg, 0.44 mmol, 0.05 eq.) and $Boc_2O$ (2.51 g, 11.5 mmol, 1.3 eq.) according to GP1. Purification by recrystallization from *n*-hexane and DCM, afforded the product (1.20 g, 4.90 mmol, 55%) as a colorless, crystalline solid.

$^1$H NMR (400 MHz, CDCl3) δ 2.49 (s, 4H), 1.56 (s, 9H), 1.12 (s, 6H).

$^{13}$C NMR (101 MHz, $CDCl_3$) δ 169.8, 149.0, 86.4, 45.7, 30.0, 27.8, 27.6.

HRMS (ESI) m/z calcd. for $C_{12}H_{19}NNaO_4$ ($[M+Na]^+$): 264.1206, found: 264.1202.

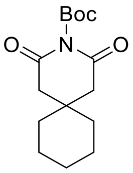


***N*-Boc 3-azaspiro[5.5]undecane-2,4-dione (D4):** Was prepared from 3-azaspiro[5.5]undecane-2,4-dione (520 mg, 2.87 mmol, 1 eq.), DMAP (17.5 mg, 0.14 mmol, 0.05 eq.) and $Boc_2O$ (814 mg, 3.73 mmol, 1.3 eq.) according to GP1. Purification by column chromatography (10-20% EtOAc in *n*-hexane) afforded the product (533 mg, 1.90 mmol, 66%) as a colorless solid.

$^1$H NMR (400 MHz, $CDCl_3$) δ 2.53 (s, 4H), 1.54 (s, 9H), 1.51 – 1.40 (m, 10H).

$^{13}$C NMR (101 MHz, $CDCl_3$) δ 169.7, 149.0, 86.31, 43.3, 36.0, 32.7, 27.6, 25.6, 21.5.

HRMS (ESI) m/z calcd. for $C_{15}H_{23}NNaO_4$ ($[M+Na]^+$): 304.1519, found: 304.1518.

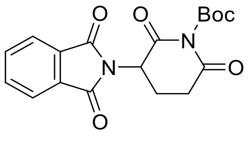


***N*-Boc Thalidomide (D5):** Was prepared from Thalidomide (351 mg, 1.36 mmol, 1 eq.), DMAP (14.1 mg, 0.12 mmol, 0.09 eq.) and $Boc_2O$ (696 mg, 3.19 mmol, 2.35 eq.) according to GP1. Purification by column chromatography (12-100% EtOAc in *n*-hexane) afforded the product (416 mg, 1.16 mmol, 85%) as a light-yellow solid.

$^1$H NMR (400 MHz, CDCl3) δ 7.92 – 7.86 (m, 2H), 7.79 – 7.74 (m, 2H), 5.07 – 5.00 (m, 1H), 3.05 – 2.74 (m, 3H), 2.20 – 2.09 (m, 1H), 1.56 (s, 9H).

$^{13}$C NMR (101 MHz, $CDCl_3$) δ 168.8, 167.3, 166.2, 147.9, 134.6, 131.9, 124.0, 87.0, 49.6, 31.8, 27.6, 22.0.

HRMS (ESI) m/z calcd. for $C_{18}H_{18}N_2NaO_6$ ($[M+Na]^+$): 381.1057, found: 381.1054.

**3.2 Alkyl chlorides**

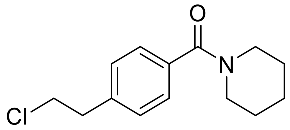

**(4-(2-chloroethyl)phenyl)(piperidin-1-yl)methanone (B7):** To a solution of 4-(2-chloroethyl)benzoic acid (1.00 g, 5.42 mmol, 1.0 equiv) in DCM (20 mL) containing a catalytic amount of DMF was added dropwise oxalyl chloride (791 mg, 6.23 mmol, 545 µL, 1.15 equiv) in DCM (4 mL) at rt. The mixture was stirred for 2 h, and formation of the acid chloride was monitored by TLC. After completion, the reaction was concentrated *in vacuo*. The residue was dissolved in DCM (20 mL) and cooled to 0 °C, and piperidine (924 mg, 10.9 mmol, 1.10 mL, 2.0 equiv) was added dropwise. The mixture was stirred to completion (16 h), diluted with DCM (15 mL), and the organic layer was washed with 2 M HCl (1 × 15 mL) and brine, dried over $MgSO_4$, filtered, and concentrated *in vacuo*. The crude product was purified by column chromatography (10–100% EtOAc in *n*-hexane) to afford the product (1.18 g, 4.70 mmol, 87%) as a yellow viscous oil.

$^1$H NMR (400 MHz, CDCl3) δ 7.37 – 7.33 (m, 2H), 7.26 – 7.22 (m, 2H), 3.80 – 3.60 (m, 4H), 3.35 (br s, 2H), 3.08 (t, J = 7.3 Hz, 2H), 1.72 – 1.44 (m, 6H).

$^{13}$C NMR (101 MHz, $CDCl_3$) δ 170.2, 139.6, 135.2, 129.0, 127.3, 48.5, 44.7, 39.0, 26.3, 24.7.

HRMS (ESI) m/z calcd. for $C_{14}H_{19}ClNO$ ($[M+H]^+$): 252.1150, found: 252.1146.

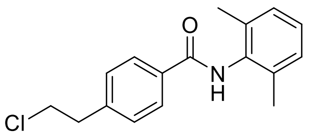

**4-(2-chloroethyl)-*N*-(2,6-dimethylphenyl)benzamide (B8):** To a solution of 4-(2-chloroethyl)benzoic acid (500 mg, 2.71 mmol, 1.0 equiv) in DCM (10 mL) containing a catalytic amount of DMF was added dropwise oxalyl chloride (396 mg, 3.12 mmol, 272 µL, 1.15 equiv) in DCM (2 mL) at rt. The mixture was stirred for 2 h, and formation of the acid chloride was monitored by TLC. After completion, the reaction was concentrated *in vacuo*. The residue was dissolved in DCM (10 mL) and cooled to 0 °C, and 2,6-dimethylaniline (492 mg, 4.06 mmol, 1.10 mL, 1.5 equiv) was added dropwise, followed by triethylamine (411 mg, 4.06 mmol, 566 uL, 1.5 equiv). The mixture was stirred to completion, diluted with DCM (15 mL), and the organic layer was washed with 2 M HCl (1 × 15 mL) and brine, dried over $MgSO_4$, filtered, and concentrated *in vacuo*. The crude

product was purified by column chromatography (5–40% EtOAc in *n*-hexane) to afford the product (596 mg, 2.07 mmol, 77%) as a colorless solid.

$^1$H NMR (400 MHz, $CDCl_3$) δ 7.93 – 7.86 (m, 2H), 7.39 – 7.35 (m, 2H), 7.34 (br s, 1H), 7.18 – 7.09 (m, 3H), 3.77 (t, *J* = 7.1 Hz, 2H), 3.16 (t, *J* = 7.1 Hz, 2H), 2.29 (s, 6H).

$^{13}$C NMR (101 MHz, $CDCl_3$) δ 165.7, 142.3, 135.7, 134.0, 133.3, 129.4, 128.5, 127.7, 127.6, 44.7, 39.0, 18.7.

HRMS (ESI) m/z calcd. for $C_{17}H_{18}ClNNaO$ ($[M+Na]^+$): 310.0969, found: 310.0966.

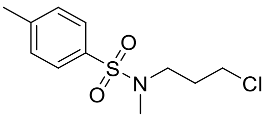


***N*-(3-chloropropyl)-*N*,4-dimethylbenzenesulfonamide (B9):** 3-Chloro-N-methylpropan-1-amine hydrochloride (500 mg, 3.47 mmol, 1.0 equiv) was suspended in DCM (14.0 mL), and 4-methylbenzenesulfonyl chloride (794 mg, 4.17 mmol, 1.2 equiv) was added. To the stirred mixture, triethylamine (773 mg, 1.06 mL, 7.64 mmol, 2.2 equiv) was added slowly at rt, and the solution was stirred for 16 h. The reaction mixture was quenched with 2 M HCl (15 mL), and the layers were separated. The aqueous phase was extracted with DCM (15 mL), and the combined organic layers were washed with water (15 mL) and brine (15 mL), dried over $MgSO_4$, filtered, and concentrated in vacuo. The crude product was purified by column chromatography (5–40% EtOAc in n-hexane) to afford the product (777 mg, 2.97 mmol, 86%) as a colorless oil.

$^1$H NMR (400 MHz, $CDCl_3$) δ 7.72 – 7.64 (m, 2H), 7.37 – 7.29 (m, 2H), 3.62 (t, *J* = 6.4 Hz, 2H), 3.14 (t, *J* = 6.7 Hz, 2H), 2.75 (s, 3H), 2.44 (s, 3H).

$^{13}$C NMR (126 MHz, $CDCl_3$) δ 143.6, 134.3, 129.9, 127.6, 47.9, 42.0, 35.7, 31.2, 21.6.

HRMS (ESI) m/z calcd. for $C_{11}H_{16}ClNNaO_2S$ ($[M+Na]^+$): 284.0482, found: 284.0478.

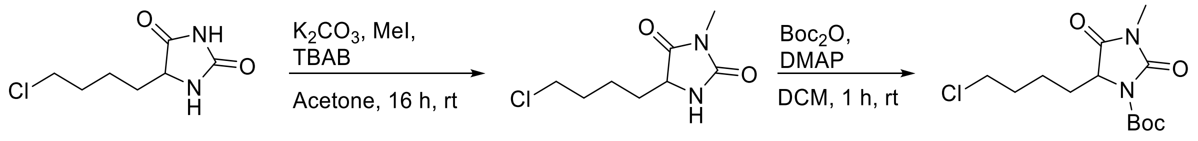


***Tert*-butyl 5-(4-chlorobutyl)-3-methyl-2,4-dioxoimidazolidine-1-carboxylate (B10):**

Methylation: 5-(4-Chlorobutyl)imidazolidine-2,4-dione (238 mg, 1.25 mmol, 1.0 equiv) was dissolved in acetone (5.00 mL). Potassium carbonate (259 mg, 1.88 mmol, 1.5 equiv) and iodomethane (266 mg, 117 µL, 1.88 mmol, 1.5 equiv) were added, followed by tetrabutylammonium bromide (10.1 mg, 31.3 µmol, 0.025 equiv). The mixture was stirred at rt until consumption of the starting material. The reaction mixture was diluted with acetone, filtered, and concentrated *in vacuo*. The residue was dissolved in EtOAc, washed

with water and brine, dried over $MgSO_4$, filtered, and concentrated *in vacuo*. Recrystallization (*n*-hexane/EtOAc) afforded the methylated intermediate (141 mg, 0.69 mmol, 55%) as an off-white solid.

Boc protection: To a suspension of 5-(4-chlorobutyl)-3-methylimidazolidine-2,4-dione (50.0 mg, 244 µmol, 1.0 equiv) and DMAP (1.49 mg, 12.2 µmol, 0.05 equiv) in DCM (0.80 mL) was added dropwise a solution of $Boc_2O$ (61.3 mg, 281 µmol, 1.15 equiv) in DCM (0.20 mL). The reaction mixture was stirred at rt for 1 h. The mixture was concentrated *in vacuo*, and the residue was purified by column chromatography (12–60% EtOAc in *n*-hexane) to afford the product (64.0 mg, 210 µmol, 86%) as a light-yellow viscous oil.

$^1$H NMR (400 MHz, $CDCl_3$) δ 4.42 (dd, *J* = 6.2, 3.5 Hz, 1H), 3.51 (t, *J* = 6.5 Hz, 2H), 3.05 (s, 3H), 2.14 – 1.98 (m, 2H), 1.86 – 1.71 (m, 2H), 1.56 (s, 10H), 1.41 – 1.28 (m, 1H).

$^{13}$C NMR (101 MHz, $CDCl_3$) δ 171.2, 152.6, 148.6, 84.6, 59.5, 44.4, 32.1, 29.3, 28.2, 25.1, 20.7.

HRMS (ESI) m/z calcd. for $C_{13}H_{21}ClN_2NaO_4$ ([M+Na]$^+$): 327.1082, found: 327.1091.

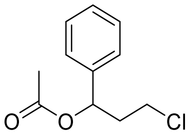


**3-Chloro-1-phenylpropyl acetate (B11):** To a solution of 3-chloro-1-phenylpropan-1-ol (450 mg, 2.64 mmol, 1.0 equiv) and DMAP (16.1 mg, 132 µmol, 0.05 equiv) in DCM (10.5 mL) was added acetyl chloride (269 mg, 0.24 mL, 3.43 mmol, 1.3 equiv) at rt, followed by triethylamine (347 mg, 478 µL, 3.43 mmol, 1.3 equiv). The reaction mixture was stirred at rt for 16 h, then a second portion of acetyl chloride (269 mg, 0.24 mL, 3.43 mmol, 1.3 equiv) was added and stirring continued for 2.5 h. The mixture was diluted with DCM (10 mL) and washed with 2 M HCl (10 mL). The aqueous phase was extracted with DCM (10 mL), and the combined organic layers were washed with water (10 mL) and brine (10 mL), dried over $MgSO_4$, filtered, and concentrated *in vacuo*. The crude product was purified by column chromatography (5–40% EtOAc in *n*-hexane) to afford 3-chloro-1-phenylpropyl acetate (296 mg, 1.39 mmol, 52.8%) as a colorless viscous oil. The NMR data are in accordance with those reported.[63]

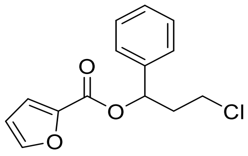


**3-Chloro-1-phenylpropyl furan-2-carboxylate (B12):** 3-Chloro-1-phenylpropan-1-ol (500 mg, 2.93 mmol, 1.0 equiv) was suspended in DCM (14.7 mL), and furan-2-carbonyl chloride (478 mg, 362 µL, 3.66 mmol, 1.25 equiv) was added at 0 °C. Triethylamine (445 mg, 613 µL, 4.40 mmol, 1.5 equiv) was then added slowly, and the mixture was stirred for 16 h while warming to rt. A second portion of furan-2-carbonyl chloride (191 mg, 145 µL, 1.47 mmol, 0.5 equiv) was then added at rt, and the mixture was stirred for an additional 3 h.

The reaction was quenched with 2 M HCl (15 mL), and the layers were separated. The aqueous phase was extracted with DCM (2 × 15 mL), and the combined organic layers were washed with water (15 mL) and brine (15 mL), dried over $MgSO_4$, filtered, and concentrated *in vacuo*. The crude product was purified by column chromatography (5–40% EtOAc in *n*-hexane) to afford the product (580 mg, 2.19 mmol, 75%) as a colorless oil.

$^1$H NMR (400 MHz, $CDCl_3$) δ 7.60 – 7.56 (m, 1H), 7.45 – 7.28 (m, 5H), 7.24 – 7.20 (m, 1H), 6.51 (dd, *J* = 3.5, 1.7 Hz, 1H), 6.20 – 6.13 (m, 1H), 3.69 – 3.58 (m, 1H), 3.58 – 3.46 (m, 1H), 2.61 – 2.48 (m, 1H), 2.40 – 2.25 (m, 1H).

$^{13}$C NMR (101 MHz, $CDCl_3$) δ 157.9, 146.62, 144.7, 139.3, 128.9, 128.6, 126.6, 118.4, 112.0, 73.8, 40.7, 39.3.

HRMS (ESI) m/z calcd. for $C_{14}H_{13}ClNaO_3$ ($[M+Na]^+$): 287.0445, found: 287.0448.

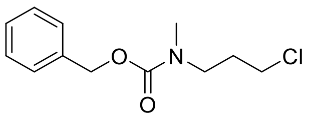


**Benzyl (3-chloropropyl)(methyl)carbamate (B13):** 3-Chloro-*N*-methylpropan-1-amine hydrochloride (500 mg, 3.47 mmol, 1.0 equiv) was suspended in DCM (17.4 mL), and benzyl carbonochloridate (681 mg, 570 µL, 3.99 mmol, 1.15 equiv) was added at 0 °C. Triethylamine (790 mg, 1.09 mL, 7.81 mmol, 2.25 equiv) was added slowly, and the mixture was stirred for 16 h while warming to rt. The reaction was quenched with 2 M HCl (15 mL) and diluted with DCM (10 mL). The layers were separated, and the aqueous phase was extracted with DCM (2 × 15 mL). The combined organic layers were washed with water (15 mL) and brine (15 mL), dried over $MgSO_4$, filtered, and concentrated *in vacuo*. The crude product was purified by column chromatography (5–40% EtOAc in *n*-hexane) to afford the product (689 mg, 2.85 mmol, 82.1%) as a colorless oil.

$^1$H NMR (400 MHz, $CDCl_3$) δ 7.40 – 7.27 (m, 5H), 5.13 (s, 2H), 3.61 – 3.48 (m, 2H), 3.48 – 3.40 (m, 2H), 2.96 (s, 3H), 2.02 (br s, 2H).

$^{13}$C NMR (126 MHz, $CDCl_3$) δ 156.4, 136.9, 128.6, 128.1, 128.0, 67.3 (rotamers), 67.2 (rotamers), 47.0 (rotamers), 46.3 (rotamers), 42.5 (rotamers), 42.3 (rotamers), 35.2 (rotamers), 34.8 (rotamers), 31.2 (rotamers), 30.8 (rotamers).

HRMS (ESI) m/z calcd. for $C_{12}H_{16}ClNNaO_2$ ($[M+Na]^+$): 264.0762, found: 264.0759.

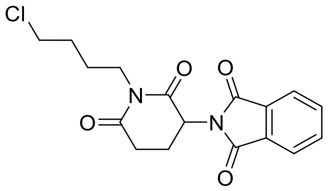

**2-(1-(4-chlorobutyl)-2,6-dioxopiperidin-3-yl)isoindoline-1,3-dione (B14):** 2-(2,6-Dioxopiperidin-3-yl)isoindoline-1,3-dione (350 mg, 1.36 mmol, 1.0 equiv) was suspended in acetone (5.42 mL), and potassium carbonate (281 mg, 2.03 mmol, 1.5 equiv) and tetrabutylammonium bromide (8.74 mg, 27.1 µmol, 0.02 equiv) were added. 1-Bromo-4-chlorobutane (349 mg, 234 µL, 2.03 mmol, 1.5 equiv) was then added, and the suspension was stirred for 48 h at rt. The mixture was diluted with acetone, filtered, and concentrated *in vacuo*. The crude solid was dissolved in DCM, washed with water and brine, and concentrated *in vacuo*. The residue was purified by column chromatography (12–100% EtOAc in *n*-hexane) to afford the product (320 mg, 0.92 mmol, 68%) as a white solid.

$^{1}$H NMR (400 MHz, $CDCl_3$) δ 7.93 – 7.84 (m, 2H), 7.83 – 7.72 (m, 2H), 5.04 – 4.93 (m, 1H), 3.92 – 3.77 (m, 2H), 3.55 (t, *J* = 6.4 Hz, 2H), 3.05 – 2.90 (m, 1H), 2.86 – 2.69 (m, 2H), 2.19 – 2.06 (m, 1H), 1.90 – 1.65 (m, 4H).

$^{13}$C NMR (126 MHz, $CDCl_3$) δ 171.0, 168.7, 167.5, 134.6, 131.9, 123.9, 50.2, 44.6, 39.9, 32.1, 29.9, 25.4, 22.2.

HRMS (ESI) m/z calcd. for $C_{17}H_{17}ClN_2NaO_4$ ($[M+Na]^+$): 371.0769, found: 371.0767.

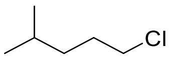

**1-Chloro-4-methylpentane (B15):** In a flame-dried vial under $N_2$, thionyl chloride (1.48 g, 907 µL, 12.4 mmol, 1.0 equiv) was added dropwise to a mixture of iso-hexanol (1.27 g, 1.50 mL, 12.4 mmol, 1.0 equiv) and pyridine (355 µL) at 0 °C. The reaction was stirred at 55 °C for 15 h. The mixture was cooled to 0 °C, quenched with water (10 mL), and extracted with $Et_2O$ (3×15 mL). The combined organic layers were washed with brine, dried over $Na_2SO_4$, filtered, and concentrated *in vacuo*. The crude product was purified by Kugelrohr distillation (50 mbar, 75 °C) to afford the product (800 mg, 6.63 mmol, 53%) as a colorless oil.

$^{1}$H NMR (400 MHz, $CDCl_3$) δ 3.52 (t, J = 6.8 Hz, 2H), 1.82 – 1.73 (m, 2H), 1.57 (dt, J = 13.2, 6.6 Hz, 1H), 1.35 – 1.25 (m, 2H), 0.90 (d, J = 6.7 Hz, 6H).

$^{13}$C NMR (101 MHz, $CDCl_3$) δ 45.6, 36.3, 30.8, 27.7, 22.6.

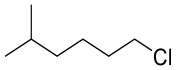

**1-Chloro-5-methylhexane (B16):** The product was prepared analogously to the procedure described above from 5-methylhexan-1-ol (4.65 g, 40.0 mmol, 5.65 mL, 1.0 equiv), thionyl chloride (5.23 g, 3.21 mL, 44.0

mmol, 1.1 equiv), and pyridine (1.14 mL). The crude product was purified by Kugelrohr distillation (30 mbar, 100 °C) to afford the product (3.89 g, 28.8 mmol, 72%) as a colorless oil.

$^1$H NMR (400 MHz, $CDCl_3$) δ 3.53 (t, J = 6.7 Hz, 2H), 1.80 – 1.69 (m, 2H), 1.62 – 1.48 (m, 1H), 1.47 – 1.37 (m, 2H), 1.25 – 1.14 (m, 2H), 0.88 (d, J = 6.6 Hz, 6H).

$^{13}$C NMR (101 MHz, $CDCl_3$) δ 45.3, 38.3, 33.0, 28.0, 24.9, 22.7.

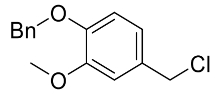


**1-(benzyloxy)-4-(chloromethyl)-2-methoxybenzene (B17):** The benzyl chloride was prepared according to a literature procedure. The NMR data are in accordance with those reported.[64]

### 3.3 Alternative *N*-carbamate succinimides

**General procedure for the preparation of *N*-carbamate substituted cyclic imides (GP2):**

To a solution of the free cyclic imide (1.0 equiv) and triethylamine (1.5 equiv) in DCM (0.40 M) was added dropwise a carbonochloridate (1.5 equiv) at 0 °C. After 1 h, the ice bath was removed and the mixture was stirred until completion was indicated by thin-layer-chromatography (typically < 1 h) at rt, then it was concentrated *in vacuo*. The residue was dissolved in DCM, washed with 2M aqueous HCl, water and brine, dried over $MgSO_4$, filtered, and concentrated *in vacuo*. The crude product was purified by column chromatography.

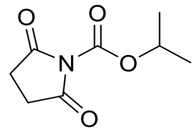


**Isopropyl 2,5-dioxopyrrolidine-1-carboxylate (A1a):** Was prepared from succinimide (495 mg, 5.00 mmol, 1.0 equiv), triethylamine (1.05 ml, 7.50 mmol, 1.50 equiv) and isopropyl carbonochloridate in toluene 1M (7.50 ml, 7.50 mmol, 1.50 equiv) according to GP2. Purification by column chromatography (12-80% EtOAc in *n*-hexane), afforded the product (831 mg, 4.49 mmol, 90%) as a colorless, waxy solid.

$^1$H NMR (400 MHz, $CDCl_3$) δ 5.18 (hept, *J* = 6.3 Hz, 1H), 2.79 (s, 4H), 1.39 (d, *J* = 6.3 Hz, 6H).

$^{13}$C NMR (101 MHz, $CDCl_3$) δ 172.76, 147.89, 73.79, 28.67, 21.61.

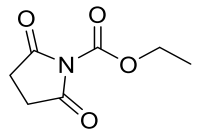

**Ethyl 2,5-dioxopyrrolidine-1-carboxylate (A1b):** Was prepared from succinimide (495 mg, 5.00 mmol, 1.0 equiv), triethylamine (1.05 ml, 7.50 mmol, 1.50 equiv) and ethyl carbonochloridate (0.72 ml, 7.50 mmol, 1.50 equiv) according to GP2. Purification by column chromatography (12-80% EtOAc in *n*-hexane), afforded the product (779 mg, 4.55 mmol, 90%) as a colorless, viscous liquid.

$^1$H NMR (400 MHz, $CDCl_3$) δ 4.44 (q, *J* = 7.1 Hz, 2H), 2.81 (s, 4H), 1.40 (t, *J* = 7.2 Hz, 3H).

$^{13}$C NMR (126 MHz, $CDCl_3$) δ 172.69, 148.37, 65.00, 28.66, 14.06.

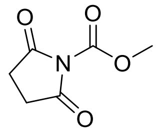

**Methyl 2,5-dioxopyrrolidine-1-carboxylate (A1c):** Was prepared from succinimide (495 mg, 5.00 mmol, 1.0 equiv), triethylamine (1.05 ml, 7.50 mmol, 1.50 equiv) and methyl carbonochloridate (0.58 ml, 7.50 mmol, 1.50 equiv) according to GP2. Purification by column chromatography (12-80% EtOAc in *n*-hexane), afforded the product (540 mg, 3.41 mmol, 68%) as a colorless solid.

$^1$H NMR (400 MHz, $CDCl_3$) δ 3.99 (s, 3H), 2.82 (s, 4H).

$^{13}$C NMR (126 MHz, $CDCl_3$) δ 172.58, 148.93, 55.15, 28.65.

## 4. Synthesis and characterization of cross-coupled products

**General procedure for the cross-electrophile coupling of *N*-Boc succinimides (GP-A):**

In an argon-filled glovebox, $Ni(COD)_2$ (55 mg, 0.20 mmol, 1.0 equiv) and 4,4'-di-*tert*-butyl-2,2'-bipyridyl (62 mg, 0.23 mmol, 1.15 equiv) were dissolved in NMP (0.40 mL). The solution was stirred at rt for 30 min, then zinc (39 mg, 0.60 mmol, 3.0 equiv) was added, followed by *N*-Boc succinimide (0.30 mmol, 1.5 equiv) and LiBr (26 mg, 0.30 mmol, 1.5 equiv). After stirring for 45 min at rt, the reaction mixture had changed color from deep purple to orange-red. The reaction was removed from the glovebox. The alkyl chloride substrate was then added, and the mixture was stirred at 60°C for 16 h. The reaction was cooled to rt, diluted with EtOAc, and quenched with 2 M HCl. The suspension was filtered through cotton, and the layers were separated. The aqueous layer was extracted with EtOAc (2 × 10 mL). The combined organic layers were washed with 1 M LiCl, dried over $MgSO_4$, filtered, and concentrated *in vacuo*. The crude residue was purified by column chromatography.

**Special case.** If the alkyl chloride or the *N*-Boc imide is a viscous liquid, it is dissolved in NMP (0.15 mL) prior to addition. In this case, the initial reaction solvent volume is reduced to 0.25 mL of NMP.

**General procedure for the cross-electrophile coupling of *N*-Boc glutarimides (GP-B):**

In an argon-filled glovebox, $Ni(COD)_2$ (55 mg, 0.20 mmol, 1.0 equiv) and 4,4'-di-*tert*-butyl-2,2'-bipyridyl (62 mg, 0.23 mmol, 1.15 equiv) were dissolved in NMP (0.40 mL). The solution was stirred at rt for 30 min, then zinc (39 mg, 0.60 mmol, 3.0 equiv) was added, followed by a *N*-Boc glutarimide (0.30 mmol, 1.5 equiv) and LiBr (26 mg, 0.30 mmol, 1.5 equiv). After stirring for 16 h at rt, the reaction mixture had changed color from deep purple to orange-red. The reaction was removed from the glovebox. The alkyl chloride substrate was then added, and the mixture was stirred at 60 °C for 9 h. The reaction was cooled to rt, diluted with EtOAc, and quenched with 2 M HCl. The suspension was filtered through cotton, and the layers were separated. The aqueous layer was extracted with EtOAc (2 × 10 mL). The combined organic layers were washed with 1 M LiCl, dried over $MgSO_4$, filtered, and concentrated *in vacuo*. The crude residue was purified by column chromatography.

**Special case.** If the alkyl chloride or the *N*-Boc imide is a viscous liquid, it is dissolved in NMP (0.15 mL) prior to addition. In this case, the initial reaction solvent volume is reduced to 0.25 mL of NMP.

**General procedure for the catalytic cross-electrophile coupling of *N*-Boc imides (GP-C):**

In an argon-filled glovebox, $Ni(COD)_2$ (14 mg, 0.05 mmol, 0.25 equiv) and 4,4'-di-*tert*-butyl-2,2'-bipyridyl (20 mg, 0.075 mmol, 0.375 equiv) were dissolved in NMP (0.40 mL). The solution was stirred at rt for 30 min, then zinc (39 mg, 0.60 mmol, 3.0 equiv) was added, followed by *N*-Boc succinimide (0.30 mmol, 1.5 equiv) and LiBr (26 mg, 0.30 mmol, 1.5 equiv). After stirring for 45 min at rt, the reaction mixture had changed color

from deep purple to orange-red. The reaction was removed from the glovebox. The alkyl chloride substrate was then added, and the mixture was stirred at 60 °C for 16 h. The reaction was cooled to rt, diluted with EtOAc, and quenched with 2 M HCl. The suspension was filtered through cotton, and the layers were separated. The aqueous layer was extracted with EtOAc (2 × 10 mL). The combined organic layers were washed with 1 M LiCl, dried over $MgSO_4$, filtered, and concentrated *in vacuo*. The crude residue was purified by column chromatography.

**Special case.** If the alkyl chloride or the *N*-Boc imide is a viscous liquid, it is dissolved in NMP (0.15 mL) prior to addition. In this case, the initial reaction solvent volume is reduced to 0.25 mL of NMP.

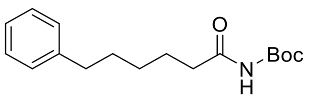


**C1:** Was prepared from *N*-Boc succinimide (60 mg, 0.30 mmol, 1.5 equiv) and (3-chloropropyl)benzene (31 mg, 29 µL, 0.20 mmol, 1 equiv) using GP-A. Purification by column chromatography (5-40% EtOAc in *n*-hexane) afforded the product (36 mg, 0.124 mmol, 62%) as a colorless, viscous oil.

**Catalytic:** Was prepared from *N*-Boc succinimide (60 mg, 0.30 mmol, 1.5 equiv) and (3-chloropropyl)benzene (31 mg, 29 µL, 0.20 mmol, 1 equiv) using GP-C. Purification by column chromatography (5-40% EtOAc in *n*-hexane) afforded the product (29 mg, 0.10 mmol, 50%) as a colorless, viscous oil.

$^1$H NMR (400 MHz, $CDCl_3$) δ 7.30 – 7.24 (m, 2H), 7.20 – 7.13 (m, 4H), 2.72 (t, *J* = 7.8 Hz, 2H), 2.61 (t, *J* = 8.1 Hz, 2H), 1.72 – 1.61 (m, 4H), 1.49 (s, 9H), 1.44 – 1.36 (m, 2H).

$^{13}$C NMR (101 MHz, $CDCl_3$) δ 174.9, 150.6, 142.7, 128.5, 128.4, 125.8, 82.5, 36.1, 35.9, 31.3, 28.9, 28.1, 24.2.

HRMS (ESI) m/z calcd. for $C_{17}H_{25}NNaO_3$ ($[M+Na]^+$): 314.1727, found: 314.1729.

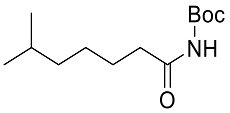


**C2:** Was prepared from *N*-Boc succinimide (60 mg, 0.30 mmol, 1.5 equiv) and 1-chloro-3-methylbutane (21 mg, 25 µL, 0.20 mmol, 1 equiv) using GP-A. Purification by column chromatography (5-40% EtOAc in *n*-hexane) afforded the product (26 mg, 0.11 mmol, 53%) as a colorless solid.

$^1$H NMR (400 MHz, $CDCl_3$) δ 7.25 (s, 1H), 2.72 (t, *J* = 7.6 Hz, 2H), 1.66 – 1.58 (m, 2H), 1.57 – 1.51 (m, 1H), 1.49 (s, 9H), 1.40 – 1.28 (m, 2H), 1.22 – 1.15 (m, 2H), 0.86 (d, *J* = 6.6 Hz, 6H).

$^{13}$C NMR (101 MHz, $CDCl_3$) δ 174.9, 150.6, 82.5, 38.8, 36.2, 28.2, 28.0, 27.1, 24.6, 22.7.

HRMS (ESI) m/z calcd. for $C_{13}H_{25}NNaO_3$ ($[M+Na]^+$): 266.1727, found: 266.1727.

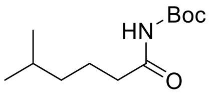


**C3:** Was prepared from *N*-Boc succinimide (60 mg, 0.30 mmol, 1.5 equiv) and 1-chloro-2-methylpropane (19 mg, 21 µL, 0.20 mmol, 1 equiv) using GP-A. Purification by column chromatography (5-40% EtOAc in *n*-hexane) afforded the product (14.0 mg, 0.06 mmol, 31%) as a colorless, viscous oil.

$^{1}$H NMR (400 MHz, $CDCl_3$) δ 7.20 (s, 1H), 2.70 (t, *J* = 7.4 Hz, 2H), 1.69 – 1.60 (m, 2H), 1.59 – 1.53 (m, 1H), 1.49 (s, 9H), 1.26 – 1.19 (m, 2H), 0.88 (d, *J* = 6.6 Hz, 6H).

$^{13}$C NMR (101 MHz, $CDCl_3$) δ 174.9, 150.6, 82.5, 38.5, 36.4, 28.2, 27.9, 22.6, 22.2.

HRMS (ESI) m/z calcd. for $C_{12}H_{23}NNaO_3$ ($[M+Na]^+$): 252.1570, found: 252.1568.

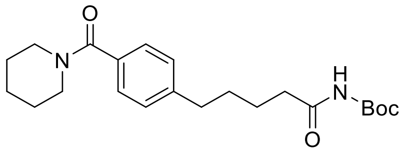


**C4:** Was prepared from *N*-Boc succinimide (60 mg, 0.30 mmol, 1.5 equiv) and (4-(2-chloroethyl)phenyl)(piperidin-1-yl)methanone (50 mg, 0.20 mmol, 1 equiv) using GP-A. Purification by column chromatography (12-100% EtOAc in *n*-hexane) afforded the product (48.0 mg, 0.12 mmol, 62%) as a colorless, viscous oil.

**Catalytic:** Was prepared from *N*-Boc succinimide (60 mg, 0.30 mmol, 1.5 equiv) and (4-(2-chloroethyl)phenyl)(piperidin-1-yl)methanone (50 mg, 0.20 mmol, 1 equiv) using GP-C. Purification by column chromatography (10-100% EtOAc in *n*-hexane), followed by extraction of the collected fractions with sat. $NaHCO_3$ (2×) and water, afforded the product (33 mg, 0.08 mmol, 42%) as a colorless, viscous oil.

$^{1}$H NMR (400 MHz, $CDCl_3$) δ 7.31 – 7.28 (m, 2H), 7.26 (s, 1H), 7.21 – 7.17 (m, 2H), 3.69 (br s, 2H), 3.36 (br s, 2H), 2.78 – 2.62 (m, 4H), 1.72 – 1.51 (m, 10H), 1.48 (s, 9H).

$^{13}$C NMR (101 MHz, $CDCl_3$) δ 174.6, 170.6, 150.6, 143.9, 134.1, 128.5, 127.1, 82.6, 49.0, 43.3, 36.0, 35.6, 30.8, 28.2, 26.7, 25.8, 24.8, 23.9.

HRMS (ESI) m/z calcd. for $C_{22}H_{32}N_2NaO_4$ ($[M+Na]^+$): 411.2254, found: 411.2256.

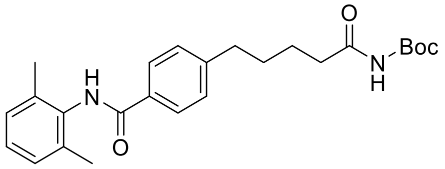


**C5:** Was prepared from *N*-Boc succinimide (60 mg, 0.30 mmol, 1.5 equiv) and 4-(2-chloroethyl)-N-(2,6-dimethylphenyl)benzamide (58 mg, 0.20 mmol, 1 equiv) using GP-A. Purification by two consecutive column chromatographies (12-100% EtOAc in *n*-hexane) and (0-5% MeOH in DCM) afforded the product (70.0 mg, 0.16 mmol, 82%) as a colorless, viscous oil.

**Catalytic:** Was prepared from *N*-Boc succinimide (60 mg, 0.30 mmol, 1.5 equiv) and 4-(2-chloroethyl)-N-(2,6-dimethylphenyl)benzamide (58 mg, 0.20 mmol, 1 equiv) using GP-C. Purification by two consecutive column chromatographies (12-100% EtOAc in *n*-hexane) and (0-5% MeOH in DCM) afforded the product (53.0 mg, 0.116 mmol, 58%, 90% pure as a mixture with *N*-Boc succinimide, yield is purity adjusted) as a colorless, viscous oil.

$^{1}$H NMR (400 MHz, $CDCl_3$) δ 7.88 – 7.80 (m, 2H), 7.38 (s, 1H), 7.34 – 7.27 (m, 2H), 7.17 – 7.07 (m, 3H), 2.80 – 2.69 (m, 4H), 2.28 (s, 6H), 1.76 – 1.66 (m, 4H), 1.49 (s, 9H).

$^{13}$C NMR (101 MHz, $CDCl_3$) δ 174.6, 165.9, 150.6, 146.7, 135.7, 134.1, 132.1, 128.9, 128.40, 127.5, 127.5, 82.7, 35.9, 35.7, 30.7, 28.1, 23.8, 18.7.

HRMS (ESI) m/z calcd. for $C_{25}H_{32}N_2NaO_4$ ($[M+Na]^+$): 447.2254, found: 447.2254.

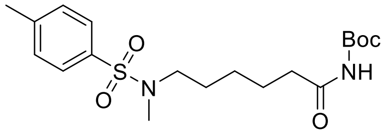


**C6:** Was prepared from *N*-Boc succinimide (60 mg, 0.30 mmol, 1.5 equiv) and *N*-(3-chloropropyl)-*N*,4-dimethylbenzenesulfonamide (52 mg, 0.20 mmol, 1 equiv) using GP-A. Purification by column chromatography (12-100% EtOAc in *n*-hexane) afforded the product (39.0 mg, 0.10 mmol, 49%) as a colorless, viscous oil.

$^{1}$H NMR (400 MHz, $CDCl_3$) δ 7.68 – 7.63 (m, 2H), 7.33 – 7.28 (m, 2H), 7.23 (s, 1H), 2.97 (t, *J* = 7.2 Hz, 2H), 2.76 – 2.67 (m, 5H), 2.45 – 2.40 (m, 3H), 1.70 – 1.61 (m, 2H), 1.60 – 1.51 (m, 2H), 1.49 (s, 9H), 1.43 – 1.32 (m, 2H).

$^{13}$C NMR (101 MHz, $CDCl_3$) δ 174.6, 150.6, 143.3, 134.8, 129.8, 127.6, 82.6, 50.1, 36.0, 34.8, 28.2, 27.5, 26.2, 23.9, 21.6.

HRMS (ESI) m/z calcd. for $C_{19}H_{30}N_2NaO_5S$ ($[M+Na]^+$): 421.1768, found: 421.1769.

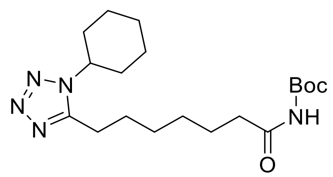


**C7:** Was prepared from *N*-Boc succinimide (60 mg, 0.30 mmol, 1.5 equiv) and 5-(4-chlorobutyl)-1-cyclohexyl-1H-tetrazole (49 mg, 0.20 mmol, 1 equiv) using GP-A. Purification by column chromatography (10-100% EtOAc in *n*-hexane) afforded the product (40.0 mg, 0.11 mmol, 53%) as a colorless solid.

$^{1}$H NMR (400 MHz, $CDCl_3$) δ 7.25 (s, 1H), 4.17 – 4.04 (m, 1H), 2.78 (t, *J* = 7.7 Hz, 2H), 2.73 (t, *J* = 7.4 Hz, 2H), 2.06 – 1.93 (m, 6H), 1.88 – 1.74 (m, 3H), 1.72 – 1.62 (m, 2H), 1.48 (s, 9H), 1.46 – 1.29 (m, 7H).

$^{13}$C NMR (101 MHz, $CDCl_3$) δ 174.7, 154.0, 150.6, 82.6, 57.7, 36.0, 33.1, 29.0, 28.8, 28.2, 27.3, 25.5, 25.0, 24.0, 23.4.

HRMS (ESI) m/z calcd. for $C_{19}H_{33}N_5NaO_3$ ($[M+Na]^+$): 402.2476, found: 402.2476

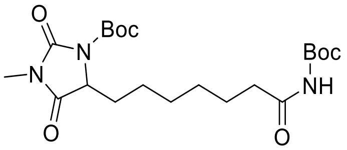


**C8:** Was prepared from *N*-Boc succinimide (60 mg, 0.30 mmol, 1.5 equiv) and tert-butyl 5-(4-chlorobutyl)-3-methyl-2,4-dioxoimidazolidine-1-carboxylate (61 mg, 0.20 mmol, 1 equiv) using GP-A. Purification by two consecutive column chromatographies (10-100% EtOAc in *n*-hexane) and (0-5% MeOH in DCM) afforded the product (45.0 mg, 0.10 mmol, 51%) as a colorless, viscous oil.

$^{1}$H NMR (500 MHz, $CDCl_3$) δ 7.21 (s, 1H), 4.41 – 4.38 (m, 1H), 3.03 (s, 3H), 2.70 (t, *J* = 7.5 Hz, 2H), 2.07 – 1.96 (m, 2H), 1.65 – 1.59 (m, 2H), 1.55 (s, 9H), 1.48 (s, 9H), 1.37 – 1.30 (m, 4H), 1.30 – 1.10 (m, 2H).

$^{13}$C NMR (126 MHz, $CDCl_3$) δ 174.7, 171.4, 152.8, 150.6, 148.6, 84.4, 82.6, 59.8, 36.0, 29.9, 29.1, 29.0, 28.2, 28.2, 25.0, 24.1, 23.1.

HRMS (ESI) m/z calcd. for $C_{21}H_{35}N_3NaO_7$ ($[M+Na]^+$): 464.2367, found: 464.2367.

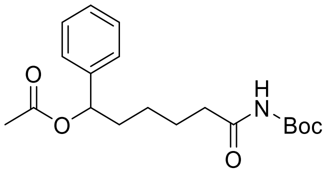


**C9:** Was prepared from *N*-Boc succinimide (60 mg, 0.30 mmol, 1.5 equiv) and 3-chloro-1-phenylpropyl acetate (43 mg, 0.20 mmol, 1 equiv) using GP-A. Purification by column chromatography (5-40% EtOAc in *n*-hexane) afforded the product (49.0 mg, 0.14 mmol, 70%) as a colorless, viscous oil.

$^{1}$H NMR (400 MHz, $CDCl_3$) δ 7.35 – 7.27 (m, 5H), 5.75 – 5.69 (m, 1H), 2.71 (t, *J* = 7.4 Hz, 2H), 2.06 (s, 3H), 1.99 – 1.88 (m, 1H), 1.86 – 1.73 (m, 1H), 1.70 – 1.61 (m, 2H), 1.48 (s, 9H), 1.44 – 1.27 (m, 2H).

$^{13}$C NMR (101 MHz, $CDCl_3$) δ 174.6, 170.5, 150.6, 140.8, 128.6, 128.0, 126.6, 82.6, 76.0, 36.2, 36.0, 28.1, 25.2, 24.0, 21.4.

HRMS (ESI) m/z calcd. for $C_{19}H_{27}NNaO_5$ ($[M+Na]^+$): 372.1781, found: 372.1

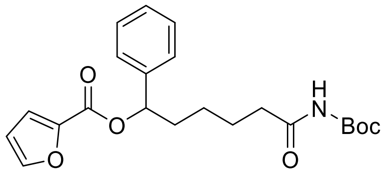


**C10:** Was prepared from *N*-Boc succinimide (60 mg, 0.30 mmol, 1.5 equiv) and 3-chloro-1-phenylpropyl furan-2-carboxylate (48 mg, 0.20 mmol, 1 equiv) using GP-A. Purification by column chromatography (5-40% EtOAc in *n*-hexane) afforded the product (48.0 mg, 0.12 mmol, 60%) as a colorless, viscous oil.

$^{1}$H NMR (400 MHz, $CDCl_3$) δ 7.60 – 7.54 (m, 1H), 7.41 – 7.27 (m, 5H), 7.23 (s, 1H), 7.21 – 7.19 (m, 1H), 6.50 (dd, *J* = 3.5, 1.7 Hz, 1H), 5.99 – 5.93 (m, 1H), 2.71 (t, *J* = 7.5 Hz, 2H), 2.13 – 2.02 (m, 1H), 1.98 – 1.85 (m, 1H), 1.74 – 1.64 (m, 2H), 1.47 (s, 9H), 1.46 – 1.30 (m, 2H).

$^{13}$C NMR (101 MHz, $CDCl_3$) δ 174.6, 158.2, 150.6, 146.4, 145.0, 140.4, 128.6, 128.2, 126.7, 118.1, 111.9, 82.6, 76.6, 36.3, 36.0, 28.1, 25.2, 24.0.

HRMS (ESI) m/z calcd. for $C_{22}H_{27}NNaO_6$ ($[M+Na]^+$): 424.1731, found: 424.1733.

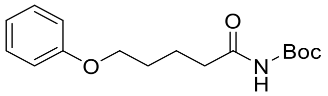

**C11:** Was prepared from *N*-Boc succinimide (60 mg, 0.30 mmol, 1.5 equiv) and (2-chloroethoxy)benzene (31 mg, 0.20 mmol, 1 equiv) using GP-A. Purification by column chromatography (5-40% EtOAc in *n*-hexane) afforded the product (22.0 mg, 0.08 mmol, 37%) as a colorless, viscous oil.

$^1$H NMR (400 MHz, $CDCl_3$) δ 7.37 (s, 1H), 7.30 – 7.23 (m, 2H), 6.97 – 6.84 (m, 3H), 4.01 – 3.95 (m, 2H), 2.85 – 2.79 (m, 2H), 1.90 – 1.80 (m, 4H), 1.49 (s, 9H).

$^{13}$C NMR (101 MHz, $CDCl_3$) δ 174.6, 159.1, 150.6, 129.5, 120.7, 114.6, 82.6, 67.5, 35.8, 28.8, 28.1, 21.0.

HRMS (ESI) m/z calcd. for $C_{16}H_{23}NNaO_4$ ([M+Na]$^+$): 316.1519, found: 316.1518.

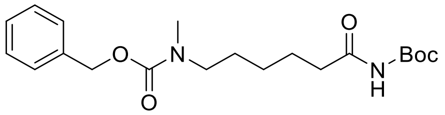

**C12:** Was prepared from *N*-Boc succinimide (60 mg, 0.30 mmol, 1.5 equiv) and benzyl (3-chloropropyl)(methyl)carbamate (48 mg, 0.20 mmol, 1 equiv) using GP-A. Purification by two consecutive column chromatographies (12-100% EtOAc in *n*-hexane) and (25-80% MTBE in *n*-hexane) afforded the product (51.0 mg, 0.14 mmol, 67%) as a colorless, viscous oil.

$^1$H NMR (400 MHz, $CDCl_3$) δ 7.37 – 7.27 (m, 5H), 7.25 (s, 1H), 5.12 (s, 2H), 3.32 – 3.21 (m, 2H), 2.90 (s, 3H), 2.75 – 2.63 (m, 2H), 1.64 (s, 2H), 1.60 – 1.52 (m, 2H), 1.49 (s, 9H), 1.40 – 1.28 (m, 2H).

$^{13}$C NMR (101 MHz, $CDCl_3$) δ 174.6, 156.4, 150.6, 137.2, 128.6, 128.0, 128.0, 82.6, 67.1, 49.1 (rotamers), 48.6 (rotamers), 36.0 (d, rotamers), 34.8 (rotamers), 34.1 (rotamers), 28.2, 27.9 (rotamers), 27.4 (rotamers), 26.3, 24.1 (d, rotamers).

HRMS (ESI) m/z calcd. for $C_{20}H_{30}N_2NaO_5$ ([M+Na]$^+$): 401.2047, found: 401.2048.

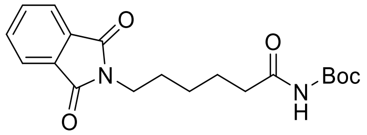

**C13:** Was prepared from *N*-Boc succinimide (60 mg, 0.30 mmol, 1.5 equiv) and 2-(3-chloropropyl)isoindoline-1,3-dione (45 mg, 0.20 mmol, 1 equiv) using GP-A. Purification by column

chromatography (12-100% EtOAc in *n*-hexane) afforded the product (39.0 mg, 0.10 mmol, 52%) as a colorless, viscous oil.

$^{1}$H NMR (400 MHz, $CDCl_3$) δ 7.87 – 7.79 (m, 2H), 7.73 – 7.67 (m, 2H), 7.26 (s, 1H), 3.68 (t, *J* = 7.2 Hz, 2H), 2.72 (t, *J* = 7.5 Hz, 2H), 1.77 – 1.63 (m, 4H), 1.55 – 1.35 (m, 11H).

$^{13}$C NMR (101 MHz, $CDCl_3$) δ 174.6, 168.6, 150.6, 134.0, 132.3, 123.3, 82.6, 38.0, 36.0, 28.5, 28.2, 26.5, 23.9.

HRMS (ESI) m/z calcd. for $C_{19}H_{24}N_2NaO_5$ ($[M+Na]^+$): 383.1577, found: 383.1577.

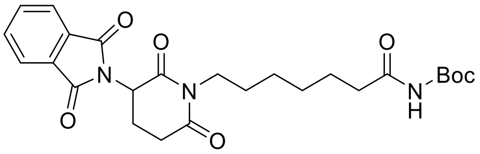


**C14:** Was prepared from *N*-Boc succinimide (60 mg, 0.30 mmol, 1.5 equiv) and 2-(1-(4-chlorobutyl)-2,6-dioxopiperidin-3-yl)isoindoline-1,3-dione (70 mg, 0.20 mmol, 1 equiv) using GP-A. Purification by column chromatography (12-100% EtOAc in *n*-hexane) afforded the product (58.0 mg, 0.12 mmol, 60%) as a colorless, viscous oil.

**Catalytic:** Was prepared from *N*-Boc succinimide (60 mg, 0.30 mmol, 1.5 equiv) and 2-(1-(4-chlorobutyl)-2,6-dioxopiperidin-3-yl)isoindoline-1,3-dione (70 mg, 0.20 mmol, 1 equiv) using GP-C. Purification by column chromatography (10-100% EtOAc in *n*-hexane) afforded the product (42.0 mg, 0.09 mmol, 43%) as a colorless, viscous oil.

$^{1}$H NMR (400 MHz, $CDCl_3$) δ 7.93 – 7.84 (m, 2H), 7.81 – 7.71 (m, 2H), 7.23 (s, 1H), 5.02 – 4.93 (m, 1H), 3.86 – 3.74 (m, 2H), 3.01 – 2.90 (m, 1H), 2.87 – 2.65 (m, 4H), 2.16 – 2.07 (m, 1H), 1.69 – 1.52 (m, 4H), 1.48 (s, 9H), 1.42 – 1.28 (m, 4H).

$^{13}$C NMR (101 MHz, $CDCl_3$) δ 174.7, 170.9, 168.6, 167.6, 150.5, 134.5, 132.0, 123.9, 82.5, 50.3, 40.8, 36.1, 32.2, 28.8, 28.2, 27.8, 26.7, 24.2, 22.2.

HRMS (ESI) m/z calcd. for $C_{25}H_{31}N_3NaO_7$ ($[M+Na]^+$): 508.2054, found: 508.2050.

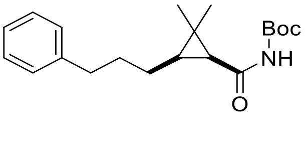


**C15:** Was prepared from tert-butyl 6,6-dimethyl-2,4-dioxo-3-azabicyclo[3.1.0]hexane-3-carboxylate (72 mg, 0.30 mmol, 1.5 equiv) and (3-chloropropyl)benzene (31 mg, 29 uL, 0.20 mmol, 1 equiv) using GP-A.

Purification by column chromatography (1-15% EtOAc in *n*-hexane) afforded the product (26 mg, 0.08 mmol, 39%) as a colorless, viscous oil.

$^{1}$H NMR (500 MHz, $CDCl_3$) δ 7.29 – 7.24 (m, 2H, Ph), 7.20 – 7.13 (m, 4H, Ph), 2.67 – 2.57 (m, 2H, α-Ph $CH_2$), 2.26 (d, *J* = 8.7 Hz, 1H, α-amide CH), 1.76 – 1.71 (m, 2H, γ-Ph $CH_2$), 1.67 – 1.60 (m, 2H, β-Ph $CH_2$), 1.49 (s, 9H, 3 x Boc-$CH_3$), 1.27 – 1.14 (m, 7H, 2 x $CH_3$ + β-amide CH).

$^{13}$C NMR (126 MHz, $CDCl_3$) δ 172.1, 150.7, 142.9, 128.5, 128.4, 125.8, 82.1, 36.0, 35.9, 31.8, 29.5, 29.4, 28.2, 27.9, 23.4, 14.6.

A 2D-NOESY NMR and 1D-NOESY cross-peak between δ 2.26 ppm and 1.21 ppm was observed, supporting the indicated cis-configuration (NOESY spectra shown in Supplementary Figure 72 and 73).

HRMS (ESI) m/z calcd. for $C_{20}H_{29}NNaO_3$ ($[M+Na]^+$): 354.2040, found: 354.2038.

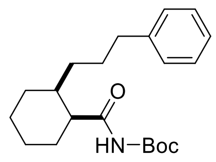


**C16:** Was prepared from tert-butyl 1,3-dioxooctahydro-2H-isoindole-2-carboxylate (76 mg, 0.30 mmol, 1.5 equiv) and (3-chloropropyl)benzene (31 mg, 29 uL, 0.20 mmol, 1 equiv) using GP-A. Purification by column chromatography (1-20% EtOAc in *n*-hexane) afforded the product (16 mg, 0.05 mmol, 23%) as a colorless, viscous oil.

$^{1}$H NMR (400 MHz, $CDCl_3$) δ 7.31 – 7.26 (m, 2H), 7.23 – 7.14 (m, 4H), 3.24 – 3.14 (m, 1H, α-amide CH), 2.69 – 2.50 (m, 2H), 1.96 – 1.88 (m, 1H, β-amide CH), 1.88 – 1.56 (m, 6H), 1.52 (s, 12H), 1.42 – 1.27 (m, 3H).

$^{13}$C NMR (101 MHz, $CDCl_3$) δ 175.8, 150.2, 142.8, 128.5, 128.4, 125.8, 82.4, 45.8, 36.9, 36.2, 29.8, 29.0, 28.5, 28.2, 25.1, 24.0, 22.3.

A 2D-NOESY NMR cross-peak between δ 3.24 – 3.14 ppm and 1.96 – 1.88 ppm was observed, supporting the indicated cis-configuration (NOESY spectra shown in Supplementary Figure 78).

HRMS (ESI) m/z calcd. for $C_{21}H_{31}NNaO_3$ ($[M+Na]^+$): 368.2196, found: 368.2196.

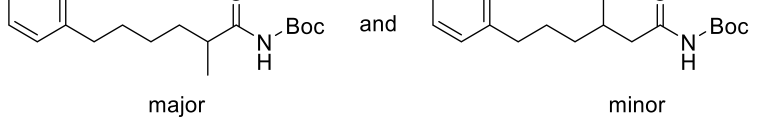


**C17:** Was prepared from tert-butyl 3-methyl-2,5-dioxopyrrolidine-1-carboxylate (64 mg, 0.30 mmol, 1.5 equiv) and (3-chloropropyl)benzene (31 mg, 29 uL, 0.20 mmol, 1 equiv) using GP-A. Purification by column chromatography (5-40% EtOAc in *n*-hexane) afforded the product as a mixture of regioisomers (α-Me/β-

Me = 1.7:1.0, 27 mg, 0.09 mmol, 44%) as a light-yellow, viscous oil. LC–MS analysis confirmed a purity >95% (UV and ELSD), consistent with the presence of only the two regioisomers reported.

$^{1}H$ NMR (400 MHz, $CDCl_3$) δ 7.30 – 7.24 (m, 5H), 7.19 – 7.15 (m, 6H), 3.08 (major, s, 1H), 2.76 – 2.64 (m, 1H), 2.63 – 2.50 (m, 4H), 2.12 – 2.02 (minor, m, 1H), 1.81 – 1.70 (m, 1H), 1.67 – 1.56 (m, 5H), 1.50 (major, s, 9H), 1.49 (minor, s, 5H), 1.44 – 1.32 (m, 4H), 1.30 – 1.22 (m, 1H), 1.16 (major, d, *J* = 6.9 Hz, 3H), 0.96 (minor, d, *J* = 6.6 Hz, 2H).

$^{13}C$ NMR (126 MHz, $CDCl_3$) δ 177.7, 174.2, 150.6, 150.2, 142.8, 142.7, 128.52, 128.51, 128.4, 125.8, 82.54, 82.52, 43.3, 39.6, 36.6, 36.2, 35.9, 33.4, 31.6, 29.5, 29.0, 28.1, 26.9, 19.8, 17.2.

HRMS (ESI) m/z calcd. for $C_{18}H_{27}NNaO_3$ ([M+Na]$^+$): 328.1883, found: 328.1880.

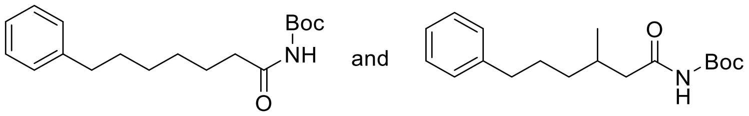


linear (major) branched (minor)

**E1:** Was prepared from *N*-Boc glutarimide (64 mg, 0.30 mmol, 1.5 equiv) and (3-chloropropyl)benzene (31 mg, 29 uL, 0.20 mmol, 1 equiv) using GP-B. Purification by column chromatography (5-40% EtOAc in *n*-hexane) afforded a mixture of the linear and branched product (28 mg, 0.09 mmol, 46%) as a colorless, viscous oil.

$^{1}H$ NMR (400 MHz, $CDCl_3$) δ 7.29 – 7.24 (m, 2H), 7.20 (s, 1H), 7.19 – 7.14 (m, 3H), 2.71 (t, *J* = 7.5 Hz, 2H), 2.60 (t, *J* = 7.5 Hz, 2H), 2.07 (minor, m, 0.24H), 1.73 – 1.58 (m, 4H), 1.49 (t, *J* = 0.8 Hz, 9H), 1.46 – 1.23 (m, 4H), 0.96 (minor, d, *J* = 6.7 Hz, 0.72H).

$^{13}C$ NMR (126 MHz, $CDCl_3$) δ 174.9 (major), 150.6 (major), 150.5 (minor), 142.9 (major), 142.8 (minor), 128.5 (major), 128.40 (minor), 128.37, 125.78 (minor), 125.7 (major), 82.6, 43.4 (minor), 36.6 (minor), 36.18 (minor), 36.17 (major), 36.0 (major), 31.5 (major), 29.5 (minor), 29.2 (major), 29.1 (major), 29.0 (minor), 28.2, 24.3 (major), 19.8 (minor).

The branched product was identified by comparison of the $^{1}H$ and $^{13}C$ NMR spectra of mixtures **E1** and **C17** (above). Matching chemical shifts in both spectra confirm that the minor products formed in these reactions are identical and correspond to the same branched structure (see Supplementary Figure 83 and 84).

HRMS (ESI) m/z calcd. for $C_{18}H_{27}NNaO_3$ ([M+Na]$^+$): 328.1883, found: 328.1882.

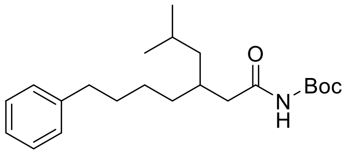


**E2:** Was prepared from tert-butyl 4-isobutyl-2,6-dioxopiperidine-1-carboxylate (81 mg, 0.30 mmol, 1.5 equiv) and (3-chloropropyl)benzene (31 mg, 29 uL, 0.20 mmol, 1 equiv) using GP-B. Purification by column chromatography (1-20% EtOAc in *n*-hexane) afforded the product (38 mg, 0.11 mmol, 53%) as a colorless, viscous oil.

$^{1}$H NMR (400 MHz, $CDCl_3$) δ 7.30 – 7.23 (m, 2H), 7.21 – 7.11 (m, 4H), 2.71 – 2.54 (m, 4H), 2.09 – 1.96 (m, 1H), 1.66 – 1.55 (m, 3H), 1.49 (s, 9H), 1.40 – 1.27 (m, 4H), 1.22 – 1.06 (m, 2H), 0.89 – 0.84 (m, 6H).

$^{13}$C NMR (101 MHz, $CDCl_3$) δ 174.4, 150.6, 142.9, 128.5, 128.4, 125.7, 82.5, 43.8, 41.1, 36.1, 34.1, 31.9, 31.9, 28.2, 26.2, 25.4, 23.0, 22.9.

HRMS (ESI) m/z calcd. for $C_{22}H_{35}NNaO_3$ ($[M+Na]^+$): 384.2509, found: 384.2508.

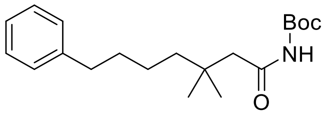


**E3:** Was prepared from tert-butyl 4,4-dimethyl-2,6-dioxopiperidine-1-carboxylate (72 mg, 0.30 mmol, 1.5 equiv) and (3-chloropropyl)benzene (31 mg, 29 uL, 0.20 mmol, 1 equiv) using GP-B. Purification by column chromatography (5-40% EtOAc in *n*-hexane) afforded the product (23 mg, 0.07 mmol, 34%) as a colorless, viscous oil.

$^{1}$H NMR (400 MHz, $CDCl_3$) δ 7.30 – 7.24 (m, 2H), 7.22 – 7.14 (m, 3H), 2.65 – 2.57 (m, 4H), 1.65 – 1.55 (m, 2H), 1.49 (s, 9H), 1.44 – 1.25 (m, 4H), 1.06 (s, 3H), 1.01 (s, 3H).

$^{13}$C NMR (126 MHz, $CDCl_3$) δ 172.9, 150.4, 142.9, 128.5, 128.4, 125.7, 82.5, 48.0 (α-$CH_2$, rotamer), 46.1 (α-$CH_2$, rotamer), 42.3, 36.1, 33.9, 32.4, 29.7 ($CH_3$, rotamer), 28.2, 27.2 ($CH_3$, rotamer), 24.0.

The methyl groups appear as two signals in the $^{1}$H NMR spectrum, and the α-amide $CH_2$ carbon shows two resonances at δ 46.14 and 47.98 ppm in the $^{13}$C NMR spectrum, possibly reflecting restricted rotation or conformational effects. The structural assignment is further supported by 2D NMR data (Supplementary Figure 86 and 87).

HRMS (ESI) m/z calcd. for $C_{20}H_{31}NNaO_3$ ($[M+Na]^+$): 356.2196, found: 356.2196.

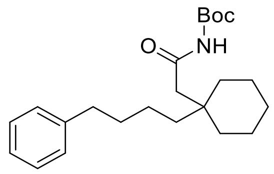


**E4:** Was prepared from tert-butyl 2,4-dioxo-3-azaspiro[5.5]undecane-3-carboxylate (84 mg, 0.30 mmol, 1.5 equiv) and (3-chloropropyl)benzene (31 mg, 29 uL, 0.20 mmol, 1 equiv) using GP-B. Purification by column chromatography (5-40% EtOAc in *n*-hexane) afforded the product (18 mg, 0.05 mmol, 24%) as a colorless, viscous oil.

$^{1}$H NMR (400 MHz, $CDCl_3$) δ 7.30 – 7.24 (m, 2H), 7.20 (s, 1H), 7.19 – 7.13 (m, 3H), 2.65 – 2.58 (m, 4H), 1.65 – 1.51 (m, 4H), 1.49 (s, 9H), 1.47 – 1.38 (m, 9H), 1.36 – 1.22 (m, 3H).

$^{13}$C NMR (101 MHz, $CDCl_3$) δ 172.8, 150.4, 143.0, 128.5, 128.4, 125.7, 82.4, 42.7, 38.0, 36.6, 36.1, 35.7, 32.4, 28.2, 26.3, 23.1, 21.8.

HRMS (ESI) m/z calcd. for $C_{23}H_{35}NNaO_3$ ($[M+Na]^+$): 396.2509, found: 396.2509.

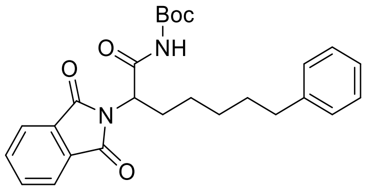


**E5:** Was prepared from *N*-Boc Thalidomide (108 mg, 0.30 mmol, 1.5 equiv) and (3-chloropropyl)benzene (31 mg, 29 uL, 0.20 mmol, 1 equiv) using GP-B. Purification by column chromatography (5-50% EtOAc in *n*-hexane) afforded the product (13 mg, 0.03 mmol, 14%) as a colorless, waxy solid.

$^{1}$H NMR (500 MHz, $CDCl_3$) δ 7.88 (dd, *J* = 5.5, 3.0 Hz, 2H), 7.80 – 7.78 (m, 1H), 7.75 (dd, *J* = 5.5, 3.0 Hz, 2H), 7.25 – 7.21 (m, 2H), 7.16 – 7.11 (m, 3H), 5.32 – 5.22 (m, 1H), 2.56 (t, *J* = 7.7 Hz, 2H), 2.48 – 2.36 (m, 1H), 2.16 – 2.05 (m, 1H), 1.65 – 1.54 (m, 3H), 1.47 (s, 9H), 1.40 – 1.33 (m, 3H).

$^{13}$C NMR (126 MHz, $CDCl_3$) δ 169.4, 168.3, 149.8, 142.6, 134.5, 131.8, 128.5, 128.4, 125.7, 123.8, 83.3, 55.5, 35.9, 31.2, 28.6, 28.4, 28.1, 26.4.

HRMS (ESI) m/z calcd. for $C_{26}H_{30}N_2NaO_5$ ($[M+Na]^+$): 473.2047, found: 473.2046.

### 4.1 Additional Substrates

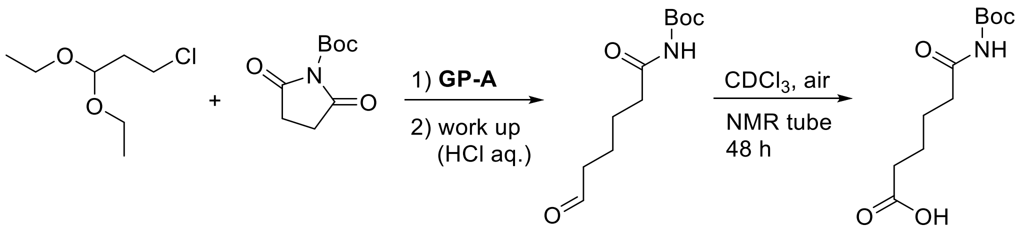


**AS1:** Was prepared from *N*-Boc succinimide (60 mg, 0.30 mmol, 1.5 equiv) and 3-Chloro-1,1-diethoxypropane (33 mg, 0.20 mmol, 1 equiv) using GP-A (including aqueous work up with 2M HCl). Purification by column chromatography (20-100% EtOAc in *n*-hexane) afforded the deprotected aldehyde product. The sample was dissolved in $CDCl_3$ (1 ml) and after 6 h under air, $^1H$ and $^{13}C$ NMR were measured. $^{13}C$ NMR indicated a mixture of two compounds consisting of the aldehyde and carboxylic acid cross-coupled product. Remeasuring of the $^1H$ and $^{13}C$ NMR confirmed full conversion to the carboxylic acid, which was obtained as a colorless solid (27.0 mg, 0.11 mmol, 55%).

$^1H$ NMR (400 MHz, $CDCl_3$) δ 7.78 (s, 1H), 2.79 – 2.73 (m, 2H), 2.41 – 2.34 (m, 2H), 1.73 – 1.66 (m, 4H), 1.48 (s, 9H).

$^{13}C$ NMR (101 MHz, $CDCl_3$) δ 178.86, 174.98, 150.66, 82.59, 35.61, 33.69, 27.99, 24.08, 23.52.

HRMS (ESI) m/z calcd. for $C_{11}H_{19}N_1NaO_5$ ($[M+Na]^+$): 268.1155, found: 268.1152.

### 4.2 Failed Substrates

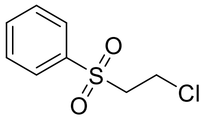

**FS1**

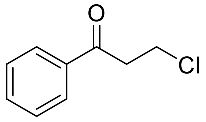

**FS2**

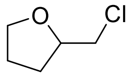

**FS3**

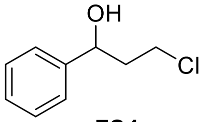

**FS4**

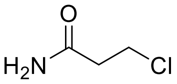

**FS5**

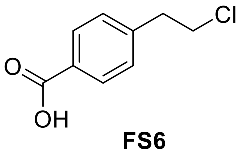

**FS6**

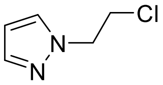

**FS7**

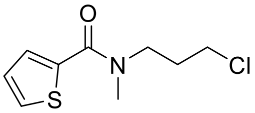

**FS8**

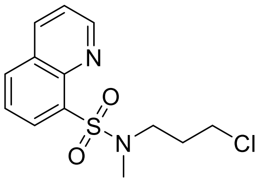

**FS9**

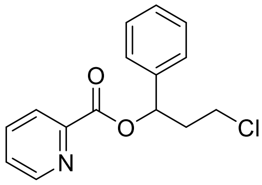

**FS10**

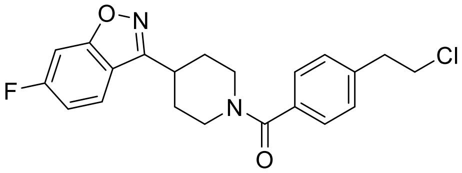

**FS11**

Observation for Nucleophiles

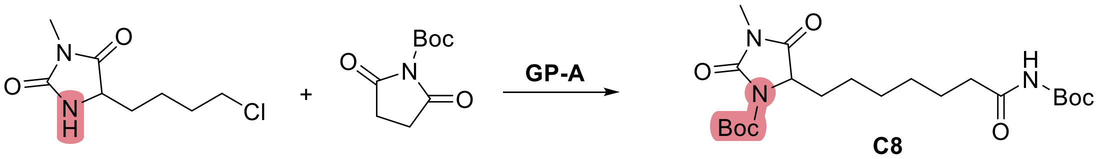


**FS12**

**C8**
isolated in low yield

Observation for Aryl halides

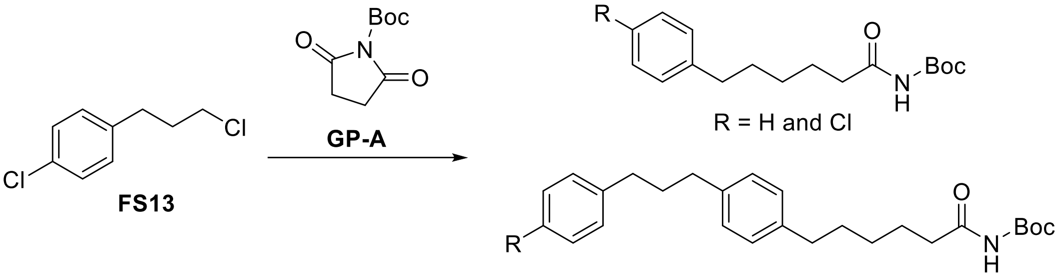


**FS13**

R = H and Cl

R = H and Cl

Alkyl chlorides with nucleophilic groups (**FS4**, **FS5**, **FS6**, **FS12**) proved to be incompatible with the developed cross-coupling conditions (**GP-A**). For the reaction of **FS12** with *N*-Boc succinimide the double Boc-protected product (**C8**) was isolated in low yield. The incompatibility of nucleophiles might therefore be explained by competition of the nucleophile against Ni for reaction with the imide substrate, the *N*-Boc imide also being able to react as a Boc transfer reagent.

## 5. Case studies

### 5.1 Maleimide diversification: Synthesis and characterization of cyclic imides

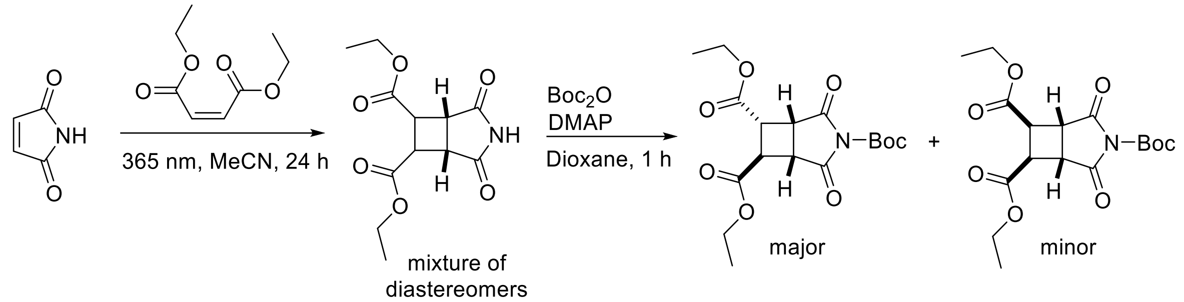


**3-(*tert*-butyl) 6,7-diethyl 2,4-dioxo-3-azabicyclo[3.2.0]heptane-3,6,7-tricarboxylate (F2):**

[2+2] cycloaddition: Maleimide (450 mg, 4.64 mmol, 1.0 equiv) and diethyl maleate (798 mg, 753 µL, 4.64 mmol, 1.0 equiv) were dissolved in MeCN (27.0 mL) and irradiated with 365 nm light (30 W) at rt for 24 h. The resulting cloudy solution was filtered and concentrated *in vacuo* to afford a colorless viscous oil. The residue was purified by column chromatography (5–100% EtOAc in *n*-hexane) to afford the free imide (527 mg, 1.96 mmol, 42%) as a colorless oil.

Boc protection: The title compound was prepared from the free imide (450 mg, 1.67 mmol, 1.0 equiv), DMAP (10.2 mg, 0.08 mmol, 0.05 equiv), and $Boc_2O$ (474 mg, 2.17 mmol, 1.3 equiv) according to GP1. Purification by column chromatography (5–80% MTBE in *n*-hexane) afforded the two diastereomers: major (400 mg, 1.10 mmol, 65%) as a colorless oil, and minor (72.0 mg, 0.19 mmol, 12%, 90% of minor enantiomer) as a colorless solid.

Major:

$^{1}$H NMR (500 MHz, $CDCl_3$) δ 4.26 – 4.16 (m, 4H), 3.82 – 3.76 (m, 1H), 3.72 – 3.67 (m, 1H), 3.59 – 3.50 (m, 2H), 1.58 (s, 9H), 1.32 – 1.26 (m, 6H).

$^{13}$C NMR (126 MHz, $CDCl_3$) δ 172.2, 171.3, 170.8, 169.2, 146.3, 86.7, 62.30, 62.29, 41.6, 41.2, 39.2, 39.0, 27.9, 14.3, 14.1.

HRMS (ESI) m/z calcd. for $C_{17}H_{23}NNaO_8$ ($[M+Na]^+$): 392.1316, found: 392.1313.

Minor:

$^{1}$H NMR (500 MHz, $CDCl_3$) δ 4.24 – 4.11 (m, 4H), 3.69 – 3.66 (m, 2H), 3.59 – 3.56 (m, 2H), 1.56 (s, 9H), 1.28 – 1.24 (m, 6H).

$^{13}$C NMR (126 MHz, $CDCl_3$) δ 172.5, 169.7, 146.4, 86.9, 62.1, 42.1, 39.7, 27.9, 14.1.

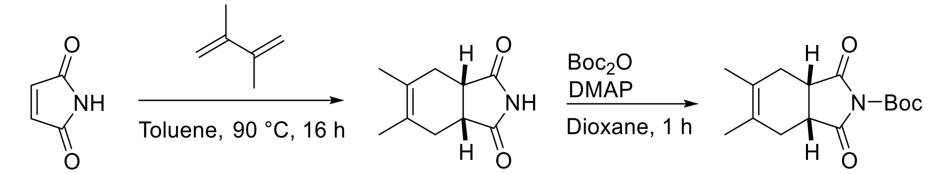


***Tert*-butyl 5,6-dimethyl-1,3-dioxo-1,3,3a,4,7,7a-hexahydro-2H-isoindole-2-carboxylate (F3):**

The free imide was prepared according to a literature procedure.[65] The title compound was prepared from the free imide (1.79 g, 10.0 mmol, 1.0 equiv), DMAP (61.0 mg, 0.50 mmol, 0.05 equiv), and $Boc_2O$ (2.83 g, 13.0 mmol, 1.3 equiv) according to GP1. Purification by two consecutive column chromatographies (10-100% EtOAc in *n*-hexane) and (40-100% DCM in *n*-hexane) afforded the product (894 mg, 3.20 mmol, 32%) as a colorless solid.

$^1$H NMR (400 MHz, $CDCl_3$) δ 3.12 – 3.01 (m, 2H), 2.48 – 2.38 (m, 2H), 2.30 – 2.20 (m, 2H), 1.69 (d, *J* = 1.1 Hz, 6H), 1.55 (s, 9H).

$^{13}$C NMR (101 MHz, $CDCl_3$) δ 176.3, 146.6, 126.9, 86.0, 40.3, 30.5, 27.9, 19.4.

HRMS (ESI) m/z calcd. for $C_{15}H_{21}NNaO_4$ ([M+Na]$^+$): 302.1363, found: 302.1360.

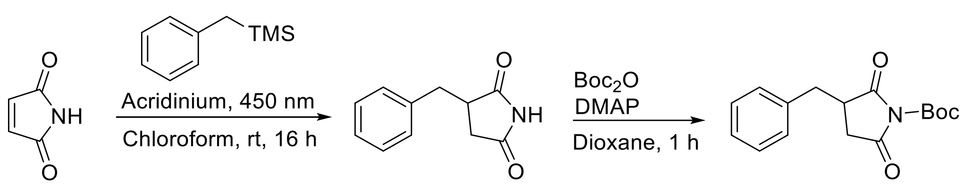


***Tert*-butyl 3-benzyl-2,5-dioxopyrrolidine-1-carboxylate (F4):**

The free imide was prepared according to a literature procedure.[66] The title compound was prepared from the free imide (243 mg, 1.28 mmol, 1.0 equiv), DMAP (7.85 mg, 0.06 mmol, 0.05 equiv), and $Boc_2O$ (364 mg, 1.67 mmol, 1.3 equiv) according to GP1. Purification by column chromatography (10-40% EtOAc in *n*-hexane) afforded the product (296 mg, 1.02 mmol, 80%) as a light-yellow, viscous oil.

$^1$H NMR (400 MHz, $CDCl_3$) δ 7.35 – 7.23 (m, 3H), 7.20 – 7.14 (m, 2H), 3.31 – 3.24 (m, 1H), 3.22 – 3.12 (m, 1H), 2.94 – 2.83 (m, 1H), 2.73 (dd, *J* = 18.5, 9.3 Hz, 1H), 2.50 (dd, *J* = 18.5, 5.6 Hz, 1H), 1.57 (s, 9H).

$^{13}$C NMR (101 MHz, $CDCl_3$) δ 175.2, 172.0, 136.9, 129.12, 129.10, 127.4, 86.4, 41.9, 36.6, 33.9, 27.9.

HRMS (ESI) m/z calcd. for $C_{16}H_{19}NNaO_4$ ([M+Na]$^+$): 312.1206, found: 312.1203.

### 5.2 Maleimide diversification: Synthesis and characterization of cross-coupled products

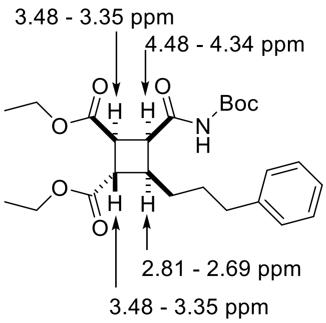


**C18:** Was prepared from 3-(*tert*-butyl) 6,7-diethyl-2,4-dioxo-3-azabicyclo[3.2.0]heptane-3,6,7-tricarboxylate (111 mg, 0.30 mmol, 1.5 equiv, trans-ester) and (3-chloropropyl)benzene (31 mg, 29 uL, 0.20 mmol, 1 equiv) using GP-A. Purification by column chromatography (5-40% EtOAc in *n*-hexane) afforded the product (25 mg, 0.05 mmol, 27%) as a colorless, waxy solid.

$^{1}$H NMR (500 MHz, $CDCl_3$) δ 7.32 (s, 1H), 7.25 – 7.21 (m, 2H), 7.18 – 7.09 (m, 3H), 4.48 – 4.34 (br s, 1H), 4.17 – 4.10 (m, 4H), 3.48 – 3.35 (m, 2H), 2.81 – 2.69 (m, 1H), 2.61 – 2.48 (m, 2H), 1.71 – 1.62 (m, 1H), 1.50 – 1.47 (m, 3H), 1.45 (s, 9H), 1.22 (t, $J$ = 7.2 Hz, 6H).

$^{13}$C NMR (126 MHz, $CDCl_3$) δ 173.6, 171.5, 150.3, 142.2, 128.4, 128.4, 125.9, 83.1, 60.9, 60.8, 45.2, 43.4, 38.8, 38.6, 35.7, 30.5, 28.2, 28.1, 14.27, 14.26.

Amide carbonyl not detected in $^{13}$C NMR under standard acquisition conditions; structure confirmed by $^{1}$H NMR, 2D NMR and HRMS. Multiple 2D-NOESY NMR cross-peaks between δ 2.75 ppm and 3.44 ppm as well as 4.40 ppm were observed, supporting the indicated relative configuration (2D-NMR spectra shown in Supplementary Figures 107-109).

HRMS (ESI) m/z calcd. for $C_{25}H_{35}NNaO_7$ ($[M+Na]^+$): 484.2306, found: 484.2304.

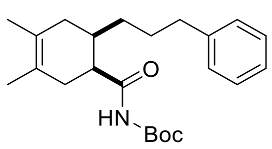

**C19:** Was prepared from *tert*-butyl-5,6-dimethyl-1,3-dioxo-1,3,3a,4,7,7a-hexahydro-2H-isoindole-2-carboxylate (84.0 mg, 0.30 mmol, 1.5 equiv) and (3-chloropropyl)benzene (31 mg, 29 uL, 0.20 mmol, 1 equiv) using GP-A. Purification by column chromatography (5-40% EtOAc in *n*-hexane) afforded the product (22 mg, 0.06 mmol, 30%) as a colorless, viscous oil.

$^{1}$H NMR (400 MHz, $CDCl_3$) δ 7.32 (s, 1H), 7.29 – 7.23 (m, 2H), 7.19 – 7.13 (m, 3H), 3.23 – 3.13 (m, 1H, α-amide CH), 2.66 – 2.47 (m, 2H), 2.37 – 2.25 (m, 1H), 2.25 – 2.15 (m, 1H), 2.14 – 1.86 (m, 3H, β-amide CH + cyclohexene $CH_2$), 1.77 – 1.57 (m, 8H), 1.49 (s, 9H), 1.43 – 1.24 (m, 2H).

$^{13}$C NMR (126 MHz, $CDCl_3$) δ 174.9, 150.0, 142.8, 128.44, 128.37, 125.8, 124.8, 123.4, 82.4, 44.3, 36.2, 35.8, 35.1, 30.9, 29.9, 29.5, 28.2, 19.2, 19.0.

A 1D-NOESY NMR cross-peak between δ 3.18 ppm and 2.00 ppm was observed, supporting the indicated cis-configuration (2D-NMR spectra shown in Supplementary Figure 112-114).

HRMS (ESI) m/z calcd. for $C_{23}H_{33}NNaO_3$ ($[M+Na]^+$): 394.2353, found: 394.2349.

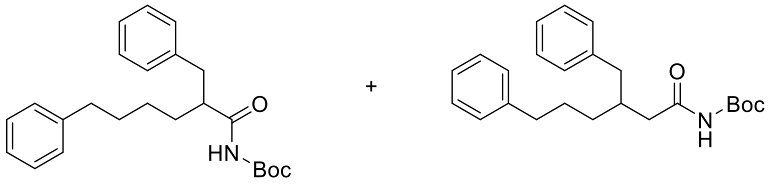


**C20:** Was prepared from *tert*-butyl 3-benzyl-2,5-dioxopyrrolidine-1-carboxylate (87 mg, 0.30 mmol, 1.5 equiv) and (3-chloropropyl)benzene (31 mg, 29 uL, 0.20 mmol, 1 equiv) using GP-A. Purification by column chromatography (5-40% EtOAc in *n*-hexane) afforded the product as a mixture of regioisomers (α-Bn/β-Bn = 1.7:1.0, 42 mg, 0.11 mmol, 56%) as a colorless, viscous oil. LC–MS analysis confirmed a purity >95% (ELSD and UV), consistent with the presence of only the two regioisomers reported.

$^{1}$H NMR (500 MHz, $CDCl_3$) δ 7.30 – 7.26 (m, 5H), 7.25 – 7.24 (m, 1H), 7.22 – 7.13 (m, 11H), 2.99 (dd, $J$ = 13.5, 8.0 Hz, 1H), 2.78 – 2.52 (m, 7H), 2.35 (p, $J$ = 6.7 Hz, 1H, minor), 1.83 – 1.73 (m, 1H), 1.73 – 1.63 (m, 2H), 1.63 – 1.56 (m, 2H), 1.55 – 1.50 (m, 1H), 1.49 (s, 5H, minor), 1.46 (s, 9H, major), 1.44 – 1.29 (m, 3H).

$^{13}$C NMR (126 MHz, $CDCl_3$) δ 176.5, 174.1, 150.5, 149.9, 142.64, 142.61, 140.5, 139.4, 129.4, 129.2, 128.51, 128.49, 128.4, 126.4, 126.1, 125.77, 125.76, 82.51, 82.48, 40.4, 40.0, 38.7, 36.1, 36.0, 35.8, 33.4, 31.6, 31.5, 28.8, 28.13, 28.07, 26.9.

HRMS (ESI) m/z calcd. for $C_{24}H_{31}NNaO_3$ ($[M+Na]^+$): 404.2196, found: 404.2193.

**5.3 Capsaicin precursors: Synthesis and characterization of cross-coupled products**

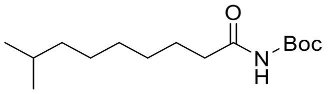

**C21:** Was prepared from *N*-Boc succinimide (299 mg, 1.50 mmol, 1.5 equiv) and (3-chloropropyl)benzene (135 mg, 145 uL, 1.00 mmol, 1 equiv) using GP-A. Purification by column chromatography (5-20% EtOAc in *n*-hexane) afforded the product (163 mg, 0.59 mmol, 59%) as a colorless, viscous oil.

$^{1}$H NMR (400 MHz, $CDCl_3$) δ 7.24 (s, 1H), 2.76 – 2.66 (m, 2H), 1.69 – 1.57 (m, 2H), 1.56 – 1.44 (m, 10H), 1.38 – 1.20 (m, 6H), 1.19 – 1.09 (m, 2H), 0.85 (d, *J* = 6.6 Hz, 6H).

$^{13}$C NMR (101 MHz, $CDCl_3$) δ 175.0, 150.6, 82.5, 39.1, 36.2, 29.8, 29.3, 28.2, 28.1, 27.4, 24.4, 22.8.

HRMS (ESI) m/z calcd. for $C_{15}H_{29}NNaO_3$ ($[M+Na]^+$): 294.2040, found: 294.2038.

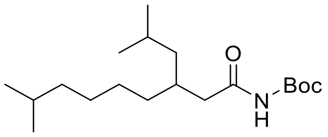

**E6:** Was prepared from *tert*-butyl 4-isobutyl-2,6-dioxopiperidine-1-carboxylate (81.0 mg, 0.30 mmol, 1.5 equiv) and 1-chloro-4-methylpentane (24.0 mg, 0.20 mmol, 1 equiv) using GP-B. Purification by column chromatography (2-15% EtOAc in *n*-hexane) afforded the product (33 mg, 0.10 mmol, 50%) as a colorless, viscous oil.

$^{1}$H NMR (400 MHz, $CDCl_3$) δ 7.17 (s, 1H), 2.72 – 2.53 (m, 2H), 2.06 – 1.96 (m, 1H), 1.66 – 1.46 (m, 12H), 1.32 – 1.07 (m, 9H), 0.92 – 0.81 (m, 12H).

$^{13}$C NMR (101 MHz, $CDCl_3$) δ 174.5, 150.5, 82.5, 43.9, 41.1, 39.2, 34.3, 32.0, 28.2, 28.1, 27.9, 26.8, 25.4, 23.1, 22.9, 22.8.

HRMS (ESI) m/z calcd. for $C_{19}H_{37}NNaO_3$ ($[M+Na]^+$): 350.2666, found: 350.2664.

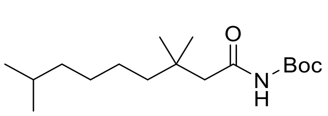

**E7:** Was prepared from *tert*-butyl 4,4-dimethyl-2,6-dioxopiperidine-1-carboxylate (72.0 mg, 0.30 mmol, 1.5 equiv) and 1-chloro-4-methylpentane (24.0 mg, 0.20 mmol, 1 equiv) using GP-B. Purification by column

chromatography (2-15% EtOAc in *n*-hexane) afforded the product (22 mg, 0.07 mmol, 36%) as a colorless, viscous oil.

$^{1}$H NMR (500 MHz, $CDCl_3$) δ 7.16 (s, 1H), 2.59 (s, 2H), 1.49 (s, 10H), 1.37 – 1.33 (m, 2H), 1.28 – 1.20 (m, 4H), 1.20 – 1.13 (m, 2H), 1.01 (s, 6H), 0.86 (d, J = 6.6 Hz, 6H).

$^{13}$C NMR (126 MHz, CDCl3) δ 173.0, 150.4, 82.4, 46.2, 42.6, 39.2, 33.9, 28.3, 28.2 (2C), 27.2, 24.5, 22.8.

HRMS (ESI) m/z calcd. for $C_{17}H_{33}NNaO_3$ ([M+Na]$^+$): 322.2353, found: 322.2351.

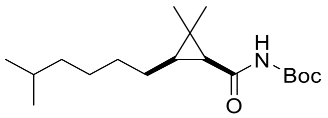


**C22:** Was prepared from *tert*-butyl 6,6-dimethyl-2,4-dioxo-3-azabicyclo[3.1.0]hexane-3-carboxylate (72.0 mg, 0.30 mmol, 1.5 equiv) and 1-chloro-5-methylhexane (27.0 mg, 0.20 mmol, 1 equiv) using GP-A. Purification by column chromatography (2-15% EtOAc in *n*-hexane) afforded the product (19 mg, 0.06 mmol, 30%) as a colorless, viscous oil.

$^{1}$H NMR (400 MHz, $CDCl_3$) δ 7.15 (s, 1H), 2.24 (d, J = 8.7 Hz, 1H), 1.49 (s, 12H), 1.31 – 1.12 (m, 13H), 0.85 (d, J = 6.6 Hz, 6H).

$^{13}$C NMR (101 MHz, $CDCl_3$) δ 172.1, 150.6, 81.9, 39.0, 36.1, 30.1, 29.4, 29.3, 28.1, 28.0, 27.7, 27.2, 23.4, 22.7, 14.5.

A 2D-NOESY NMR cross-peak between δ 2.23 ppm and 1.20 ppm was observed, supporting the indicated cis-configuration (NOESY spectrum shown in Supplementary Figure 125).

HRMS (ESI) m/z calcd. for $C_{18}H_{33}NNaO_3$ ([M+Na]$^+$): 334.2353, found: 334.2351.

**5.4 Capsaicin: Synthesis and characterization of OBn-dihydrocapsaicin**

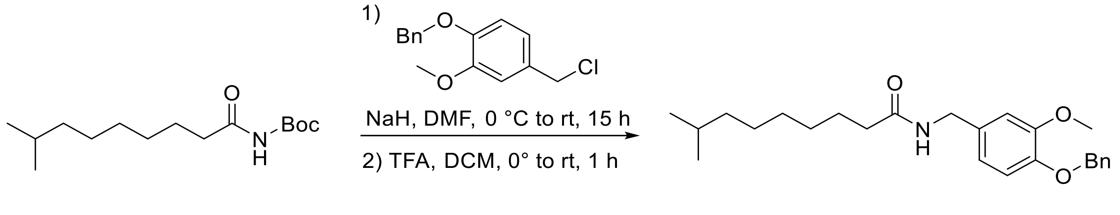


**OBn-Dihydrocapsaicin (C21):** In a flame-dried vial under $N_2$, *tert*-butyl (8-methylnonanoyl)carbamate (60.0 mg, 221 μmol, 1.0 equiv) was added portionwise to a suspension of NaH (10.6 mg, 60% w/w, 265 μmol, 1.2 equiv) in dry DMF (1.11 mL) at 0 °C. The mixture was stirred for 30 min at 0 °C before addition of 1-(benzyloxy)-4-(chloromethyl)-2-methoxybenzene (63.9 mg, 243 μmol, 1.1 equiv). The reaction was stirred at rt for 15 h, poured onto water, and extracted with EtOAc (3×). The combined organic layers were washed with water, brine, and 5% aq. LiCl, dried over $Na_2SO_4$, filtered, and concentrated *in vacuo*. The crude

residue was dissolved in DCM (1.11 mL), cooled to 0 °C, and treated dropwise with TFA (252 mg, 170 µL, 2.21 mmol, 10 equiv). After stirring at 0 °C for 1 h, the reaction mixture was concentrated *in vacuo*. The residue was resuspended in EtOAc, washed with sat. $NaHCO_3$ and brine, dried over $Na_2SO_4$, filtered, and concentrated *in vacuo*. Purification by flash column chromatography (20–40% EtOAc in *n*-hexane), followed by preparative TLC (10% EtOAc in DCM), afforded the product (26 mg, 0.06 mmol, 29%) as a white solid.

$^1$H NMR (400 MHz, $CDCl_3$) δ 7.46 – 7.39 (m, 2H), 7.39 – 7.33 (m, 2H), 7.33 – 7.27 (m, 1H), 6.86 – 6.80 (m, 2H), 6.74 (dd, J = 8.2, 2.0 Hz, 1H), 5.14 (s, 2H), 4.36 (d, J = 5.6 Hz, 2H), 3.88 (s, 3H), 2.22 – 2.16 (m, 2H), 1.65 (p, J = 7.5 Hz, 2H), 1.55 – 1.44 (m, 1H), 1.36 – 1.09 (m, 8H), 0.85 (d, J = 6.6 Hz, 6H).

$^{13}$C NMR (101 MHz, $CDCl_3$) δ 173.0, 150.0, 147.7, 137.3, 131.8, 128.7, 128.0, 127.4, 120.2, 114.2, 111.9, 71.2, 56.2, 43.6, 39.1, 37.0, 29.8, 29.5, 28.1, 27.4, 26.0, 22.8.

HRMS (ESI) m/z calcd. for $C_{25}H_{35}NNaO_3$ ($[M+Na]^+$): 420.2509, found: 420.2505.

## 6. Mechanistic studies

### 6.1 Synthesis and characterization of nickel complexes

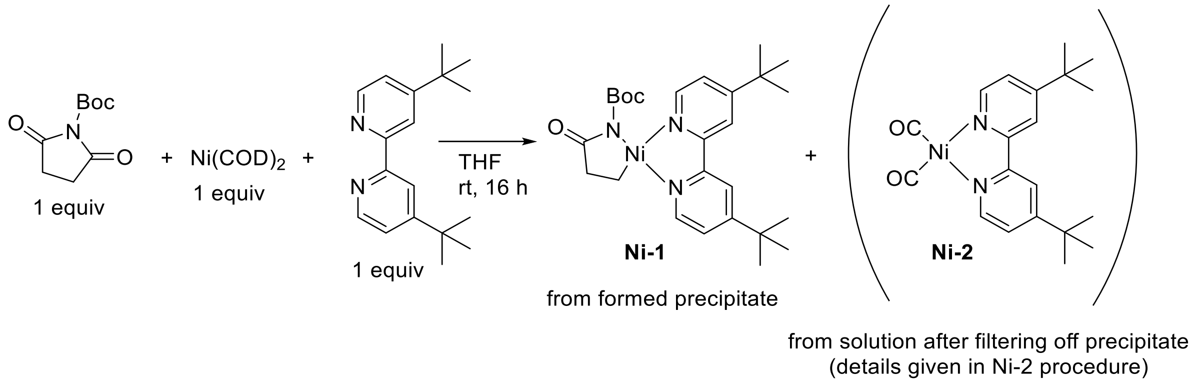


**Ni-1:** In an argon-filled glovebox, a vial equipped with a stir bar was charged with $Ni(COD)_2$ (30.0 mg, 109 µmol, 1 equiv), 4,4'-di-tert-butyl-2,2'-bipyridine (29.3 mg, 109 µmol, 1 equiv), and 1 mL THF, affording a dark purple solution. The mixture was stirred at room temperature for 45 min, after which tert-butyl 2,5-dioxopyrrolidine-1-carboxylate (21.7 mg, 109 µmol, 1 equiv) was added. Stirring was continued at room temperature for 16 h, yielding a bright red-orange precipitate suspended in a red solution. The solids were collected by filtration, washed with $Et_2O$ (4 ×), and dried *in vacuo*. The nickel product **Ni-1** (34 mg, 67 µmol, 62%) was obtained as a bright orange solid. Slow evaporation of the concentrated solution in 1,2-DCE afforded bright orange crystals suitable for single-crystal X-ray diffraction.

$^1$H NMR (400 MHz, $CD_2Cl_2$) δ 8.37 (d, *J* = 5.9 Hz, 1H), 8.16 (d, *J* = 6.1 Hz, 1H), 7.82 (dd, *J* = 13.7, 2.0 Hz, 2H), 7.46 (dd, *J* = 6.0, 2.1 Hz, 1H), 7.37 (dd, *J* = 6.0, 2.1 Hz, 1H), 2.01 (t, *J* = 7.2 Hz, 2H), 1.40 (d, *J* = 4.5 Hz, 18H), 1.26 (s, 9H), 1.24 – 1.08 (m, 2H).

$^{13}$C NMR (126 MHz, $CD_2Cl_2$) δ 163.1, 162.5, 159.1, 156.0, 152.4, 150.0, 149.6, 123.8, 123.6, 118.2, 117.2, 78.3, 40.6, 35.8 (d, *J* = 4.7 Hz), 30.4, 30.3, 28.3, 27.8.

Note: **Ni-2** was recrystallized from the filtered THF solution using this preparation, however, these crystals were not suitable for X-ray diffraction.

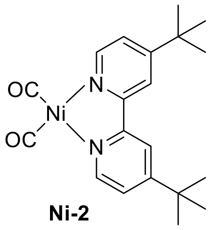


**Ni-2**

**Ni-2:** In an argon-filled glovebox, a vial equipped with a stir bar was charged with $Ni(COD)_2$ (30.0 mg, 109 µmol, 1 equiv), 4,4'-di-tert-butyl-2,2'-bipyridine (29.3 mg, 109 µmol, 1 equiv), and 1 mL THF, affording a dark purple solution. The mixture was stirred at room temperature for 45 min, after which tert-butyl 2,5-dioxopyrrolidine-1-carboxylate (21.7 mg, 109 µmol, 1 equiv) was added. Stirring was continued at room temperature for 16 h, yielding a dark red-purple solution. The solution was filtered and the filtrate was collected and layering with hexanes afforded bright red crystals of **Ni-2**, suitable for single-crystal X-ray diffraction. The $^1$H NMR spectrum of the filtrate displayed resonances consistent with literature values for **Ni-2**[67], in addition to additional resonances corresponding to an unidentified nickel species.

$^1$H NMR (400 MHz, $C_6D_6$) δ 8.98 (d, *J* = 5.5 Hz, 2H), 7.66 (s, 2H), 6.69 (d, *J* = 5.0 Hz, 2H), 1.00 (s, 18H).

$^{13}$C NMR (126 MHz, $C_6D_6$) δ 197.5 (s, CO).

### 6.2 Halide exchange studies

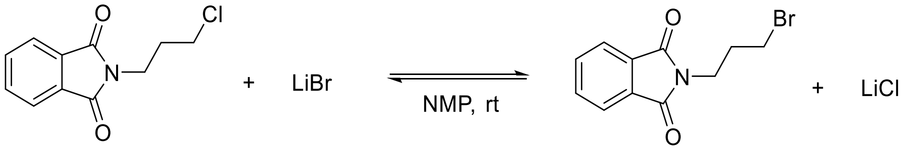


To a solution of LiBr (26 mg, 0.30 mmol, 1.5 equiv) in NMP (0.25 mL) were added 2-(3-chloropropyl)isoindoline-1,3-dione (45 mg, 0.20 mmol, 1.0 equiv) and internal standard (6-phenyl-1-(piperidin-1-yl)hexan-1-one, 10 mg) in NMP (0.15 mL). The mixture was stirred at rt for either 10 min or 8 h. Aliquots (100 µL) were withdrawn, diluted with MeCN (1 mL), and analyzed by LC–MS. All measurements were performed in triplicate.

***Supplementary Table 7. Alkyl halide exchange data points measured by calibrated LC-MS after 10 min and 8 h.***

| time [min] | AlkCl [%] | AlkBr [%] |
|---|---|---|
| 0 | 100 | 0 |
| 10 | 95 | 5 |
| 10 | 88 | 4 |
| 10 | 94 | 5 |
| 10 (avg.) | 92 | 5 |
| 480 | 95 | 8 |
| 480 | 91 | 8 |
| 480 | 89 | 8 |
| 480 (avg.) | 92 | 8 |

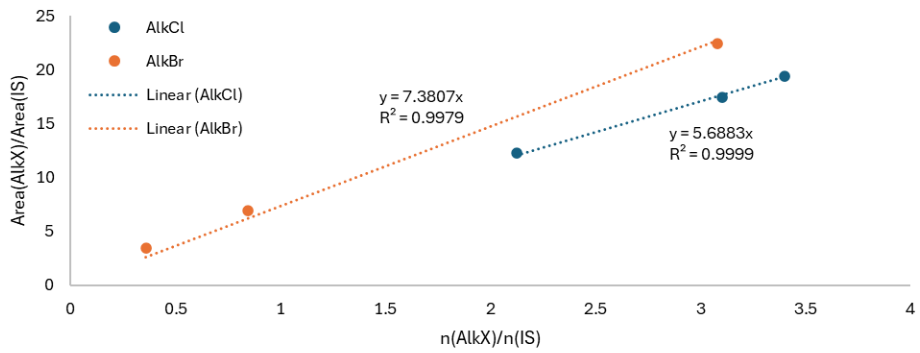


***Supplementary Figure 5. Calibration curve for LC-MS quantification of alkyl halides, 6-phenyl-1-(piperidin-1-yl)hexan-1-one was used as an internal standard.***

### 6.3 Radical clock studies

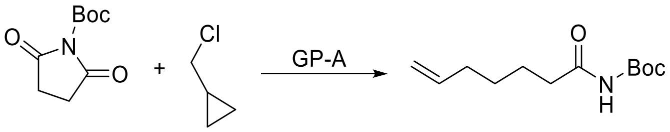


**C23:** Was obtained from *N*-Boc succinimide (60 mg, 0.30 mmol, 1.5 equiv) and (chloromethyl)cyclopropane (18.0 mg, 18 uL, 0.20 mmol, 1 equiv) using GP-A. Purification by column chromatography (5-40% EtOAc in *n*-hexane) afforded the ring-opened product (16 mg, 0.07 mmol, 35%) as a colorless oil. Alternatively, the yield was determined by $^{1}$H-NMR using 1,3,5-trimethoxybenzene as an internal standard, added prior to aqueous work-up. The ring-opened product was observed in 71%.

$^{1}$H NMR (400 MHz, $CDCl_3$) δ 7.27 (s, 1H), 5.87 – 5.72 (m, 1H), 5.05 – 4.90 (m, 2H), 2.73 (t, *J* = 7.4 Hz, 2H), 2.13 – 2.02 (m, 2H), 1.70 – 1.62 (m, 2H), 1.49 (s, 9H), 1.48 – 1.41 (m, 2H).

$^{13}$C NMR (101 MHz, $CDCl_3$) δ 174.8, 150.6, 138.7, 114.8, 82.6, 36.0, 33.6, 28.5, 28.2, 23.8.

HRMS (ESI) m/z calcd. for $C_{12}H_{21}NNaO_3$ ($[M+Na]^+$): 250.1414, found: 250.1411.

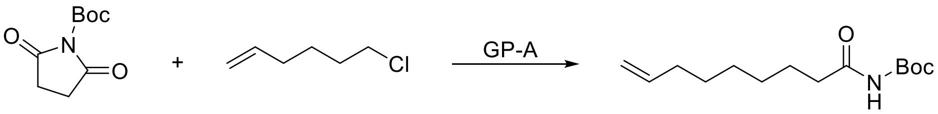


**C24:** Was obtained from *N*-Boc succinimide (60 mg, 0.30 mmol, 1.5 equiv) and 6-chlorohex-1-ene (24.0 mg, 26 uL, 0.20 mmol, 1 equiv) using GP-A. Purification by column chromatography (5-40% EtOAc in *n*-hexane) afforded the non-cyclized product (16 mg, 0.07 mmol, 33%) as a colorless oil. Alternatively, the yield was

determined by $^{1}$H NMR using 1,3,5-trimethoxybenzene as an internal standard, added prior to aqueous work-up. The non-cyclized product was observed in 70%.

$^{1}$H NMR (400 MHz, $CDCl_3$) δ 7.28 (s, 1H), 5.85 – 5.73 (m, 1H), 5.01 – 4.89 (m, 2H), 2.71 (t, *J* = 7.3 Hz, 2H), 2.07 – 1.99 (m, 2H), 1.68 – 1.60 (m, 2H), 1.48 (s, 9H), 1.41 – 1.31 (m, 6H).

$^{13}$C NMR (101 MHz, $CDCl_3$) δ 174.9, 150.6, 139.2, 114.4, 82.5, 36.2, 33.8, 29.1, 29.0, 28.9, 28.2, 24.3.

HRMS (ESI) m/z calcd. for $C_{14}H_{25}NNaO_3$ ($[M+Na]^+$): 278.1712, found: 278.1712.

Based on these data and reported rate constants, we conclude that radical capture occurs on a time scale between cyclopropylmethyl radical ring opening ($k_{RO} = 10^8$ $s^{-1}$)[68] and hexenyl radical cyclization ($k_{cyc} = 10^5$ $s^{-1}$)[69].

### 6.4 Mechanistic Proposal

Based on the above performed mechanistic experiments and literature precedent[6,32,34] we propose the depicted "radical chain" cross-electrophile coupling mechanism.

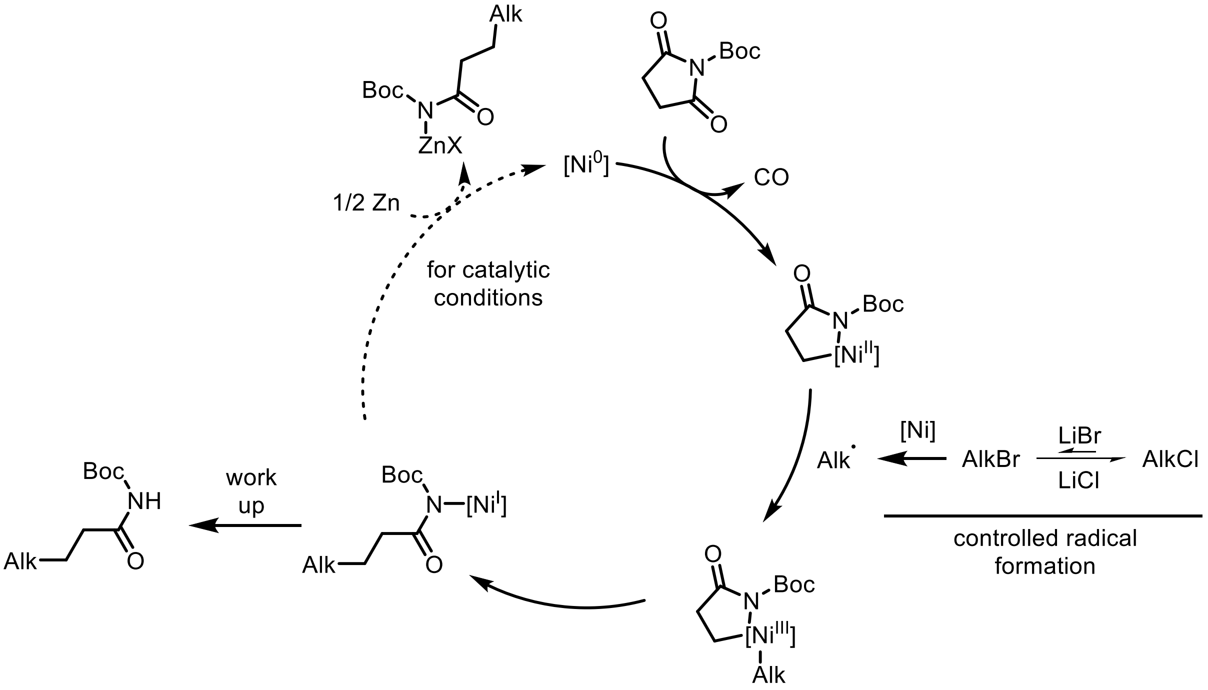


***Supplementary Scheme 1. Possible mechanism for the decarbonylative Csp$^3$-Csp$^3$ cross-electrophile coupling of N-Boc cyclic imides and alkyl chlorides.***

## 7. NMR spectra

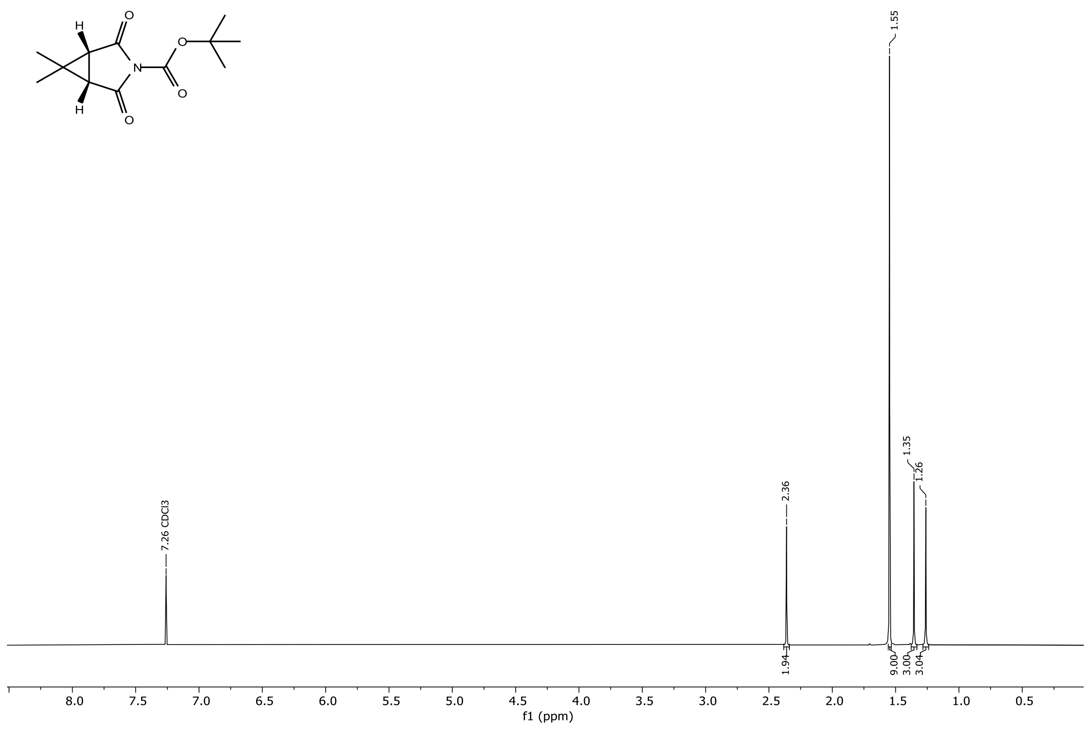

*Supplementary Figure 6. ¹H NMR spectrum of N-Boc 6,6-dimethyl-3-azabicyclo[3.1.0]hexane-2,4-dione (A3).*

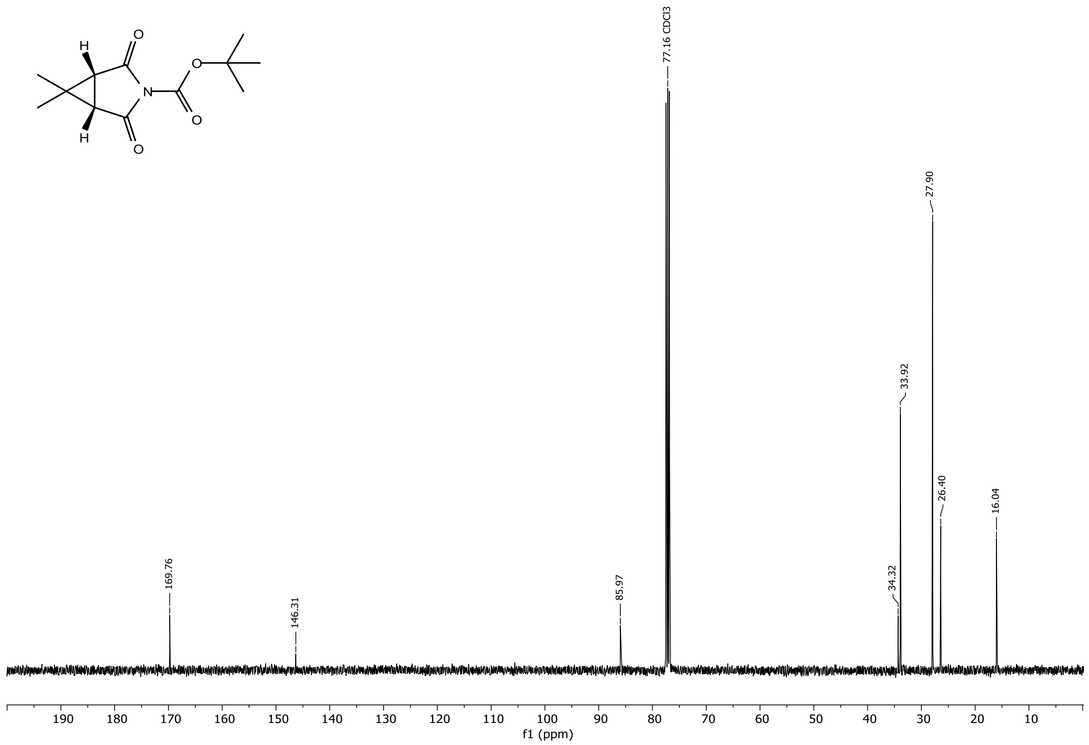

*Supplementary Figure 7. ¹³C NMR spectrum of N-Boc 6,6-dimethyl-3-azabicyclo[3.1.0]hexane-2,4-dione (A3).*

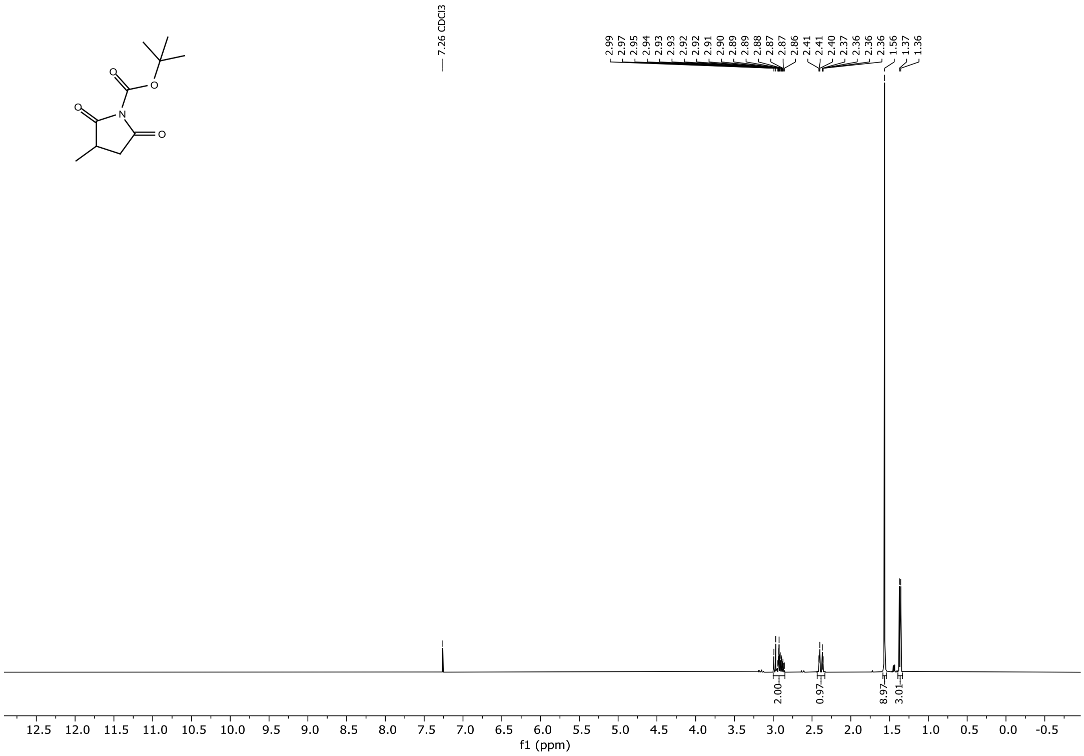


***Supplementary Figure 8. $^{1}H$ NMR spectrum of N-Boc 3-methylpyrrolidine-2,5-dione (A4).***

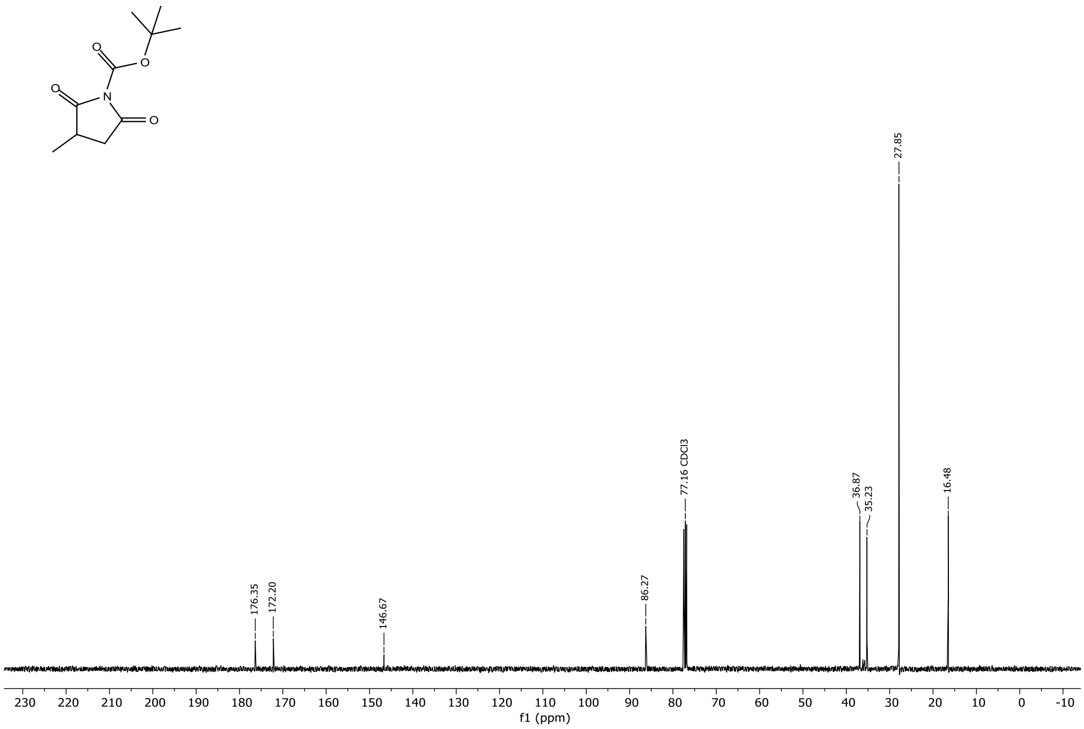


***Supplementary Figure 9. $^{13}C$ NMR spectrum of N-Boc 3-methylpyrrolidine-2,5-dione (A4).***

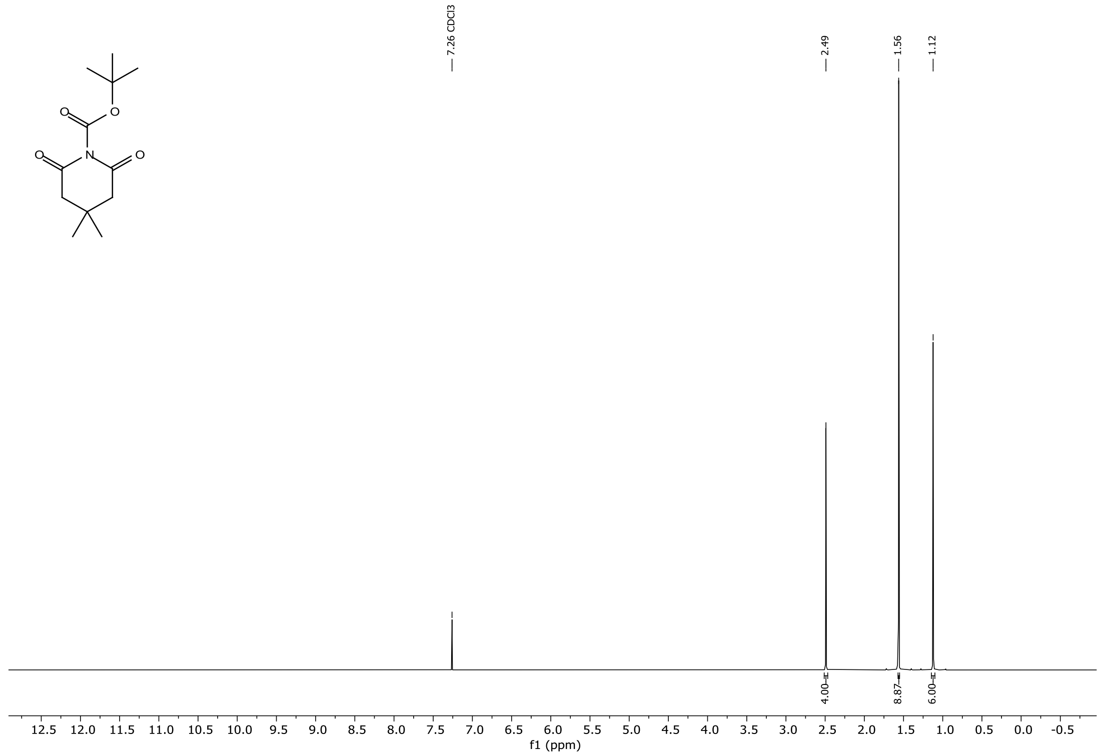


***Supplementary Figure 10. ¹H NMR spectrum of N-Boc 4,4-dimethylpiperidine-2,6-dione (D2).***

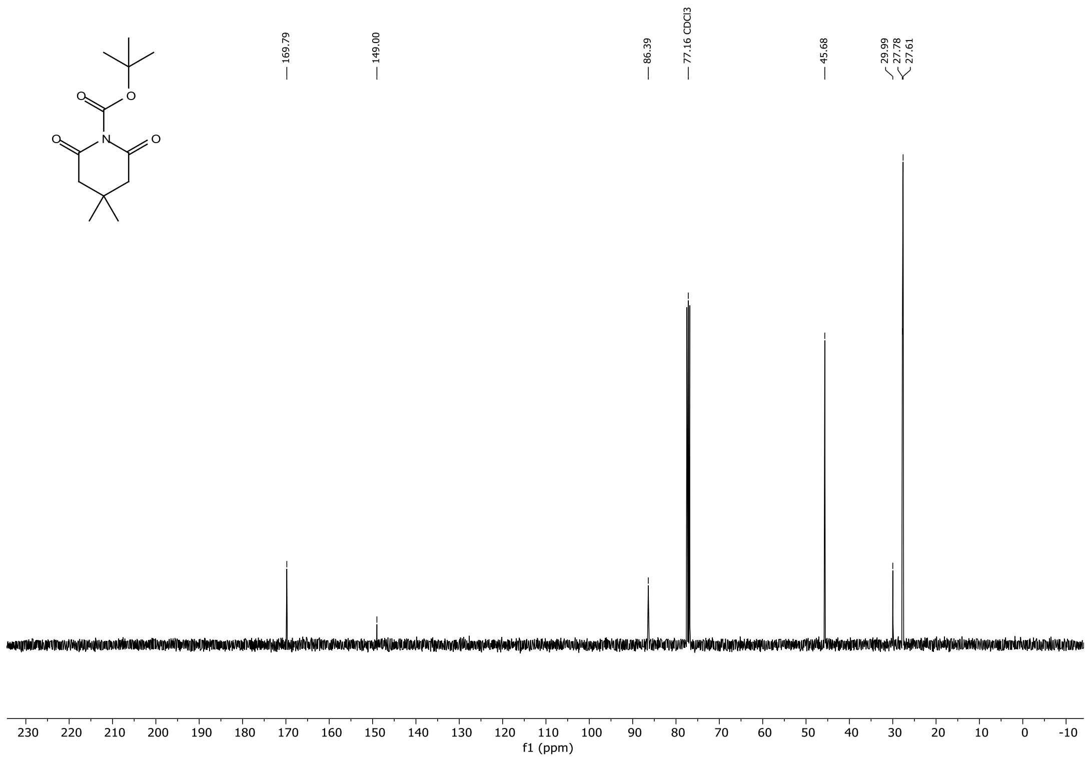


***Supplementary Figure 11. ¹³C NMR spectrum of N-Boc 4,4-dimethylpiperidine-2,6-dione (D2).***

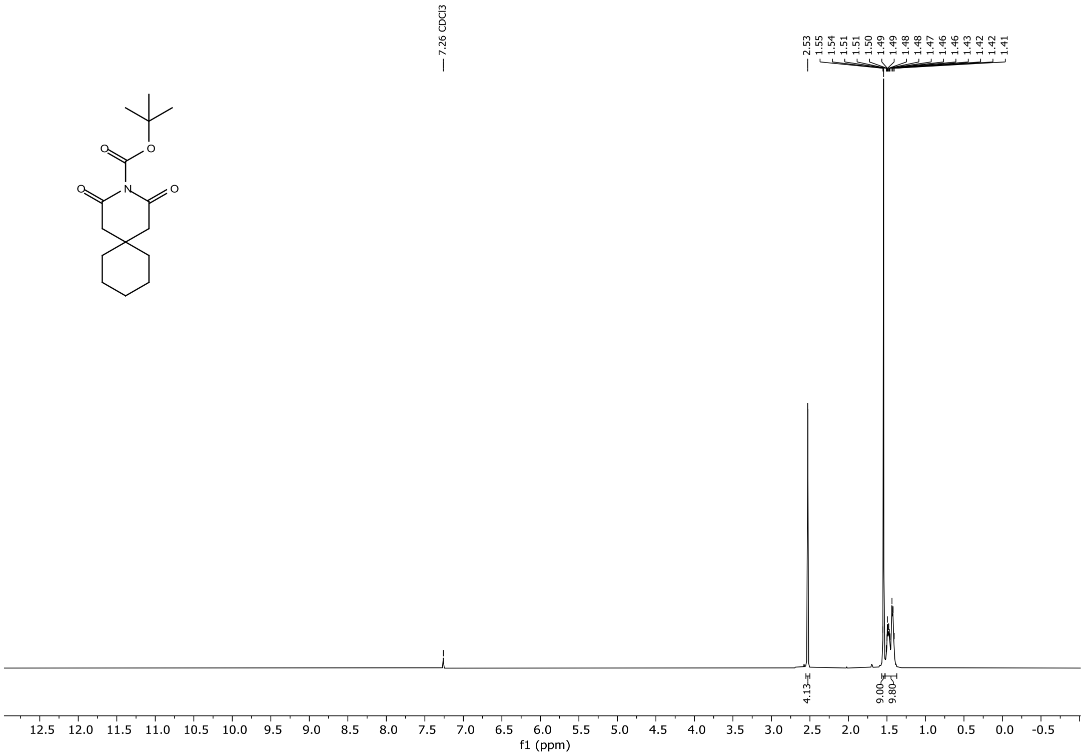


***Supplementary Figure 12. $^{1}$H NMR spectrum of N-Boc 3-azaspiro[5.5]undecane-2,4-dione (D3).***

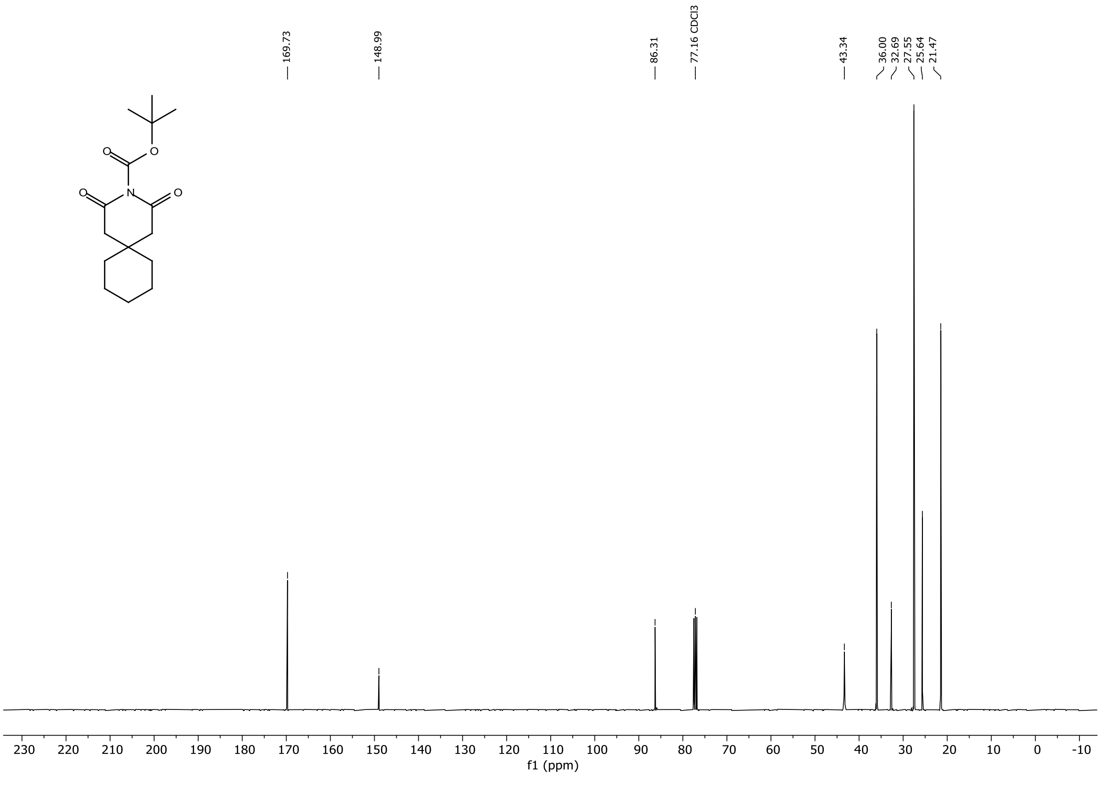


***Supplementary Figure 13. $^{13}$C NMR spectrum of N-Boc 3-azaspiro[5.5]undecane-2,4-dione (D3).***

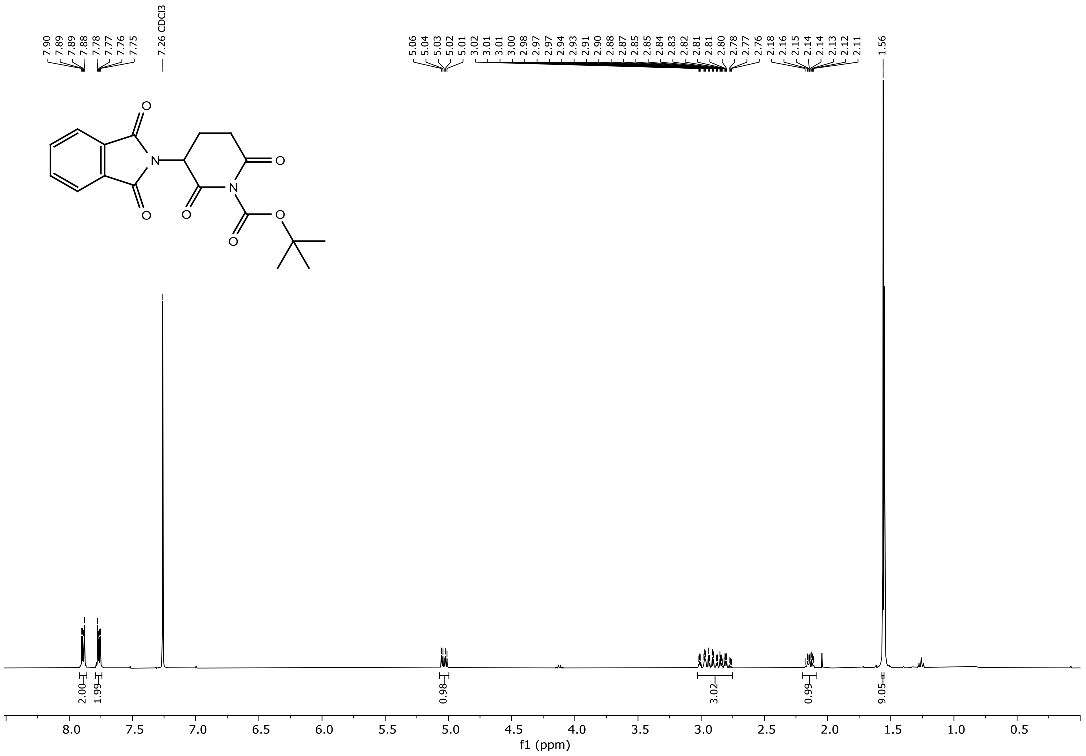

***Supplementary Figure 14. ¹H NMR spectrum of N-Boc Thalidomide (D4).***

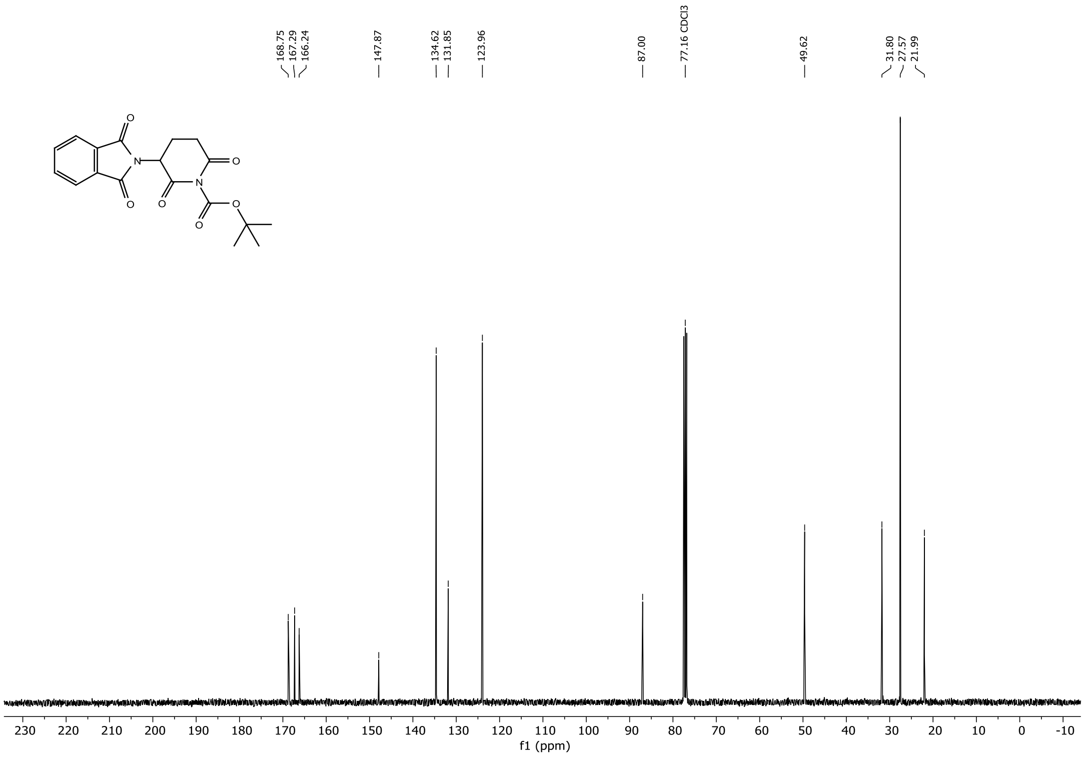

***Supplementary Figure 15. ¹³C NMR spectrum of N-Boc Thalidomide (D4).***

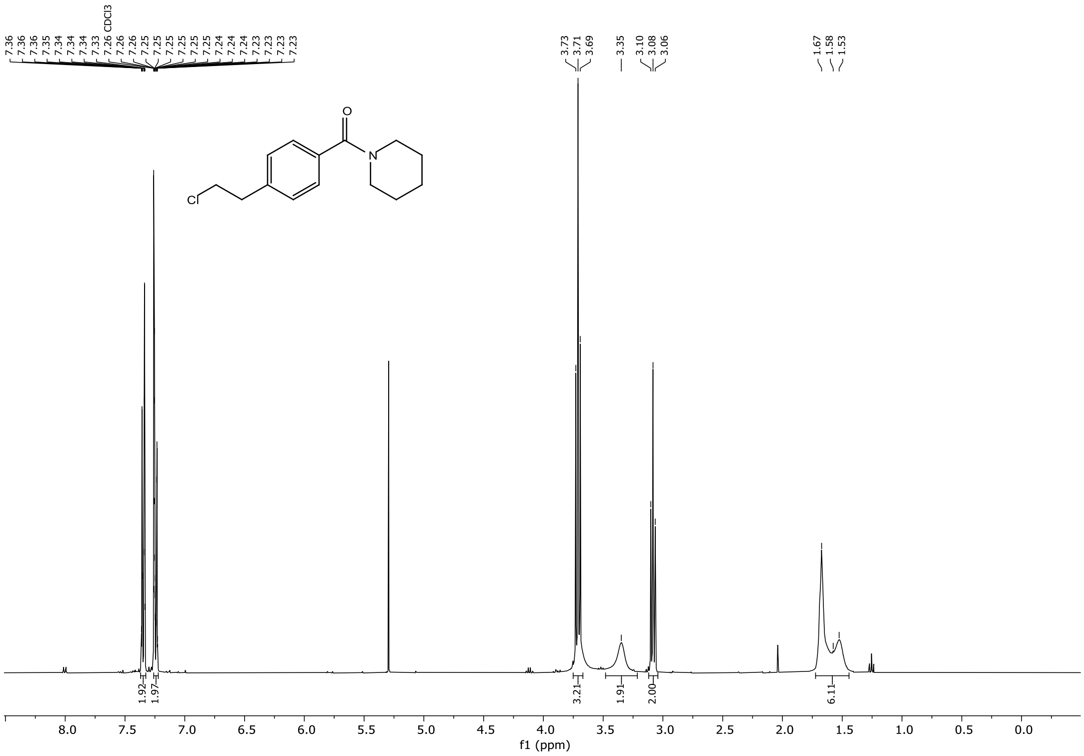


***Supplementary Figure 16. <sup></sup>***

***Supplementary Figure 16. $^{1}$H NMR spectrum of (4-(2-chloroethyl)phenyl)(piperidin-1-yl)methanone (B7).***

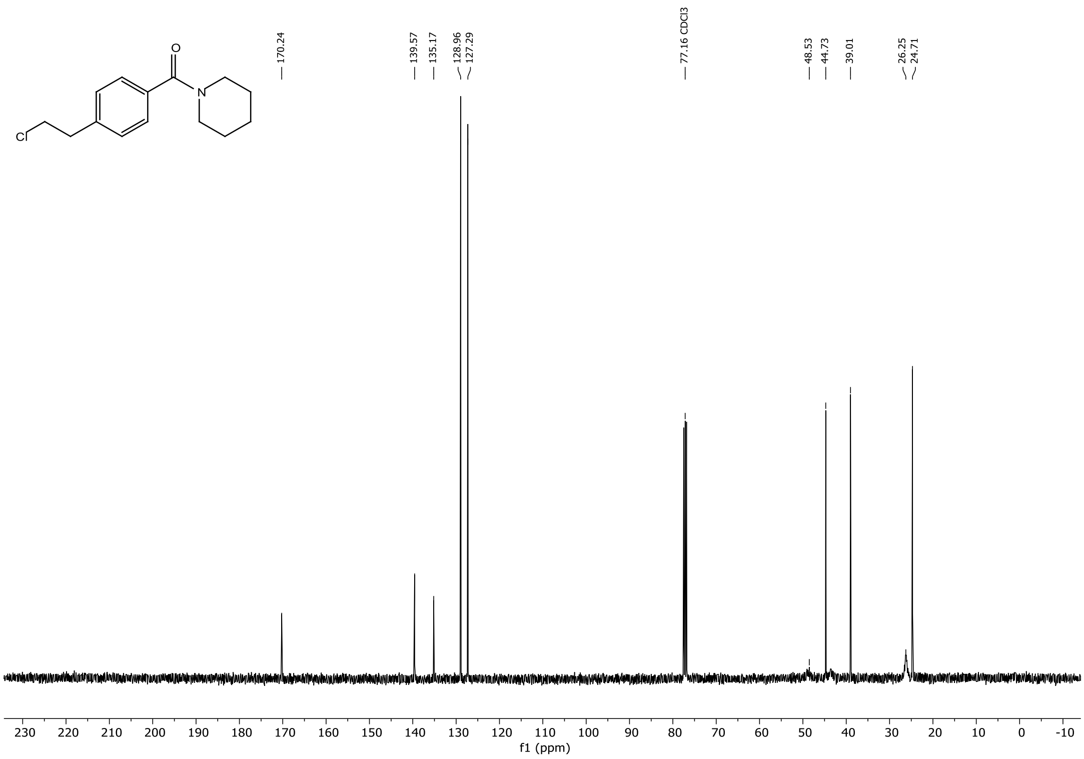


***Supplementary Figure 17. $^{13}$C NMR spectrum of (4-(2-chloroethyl)phenyl)(piperidin-1-yl)methanone (B7).***

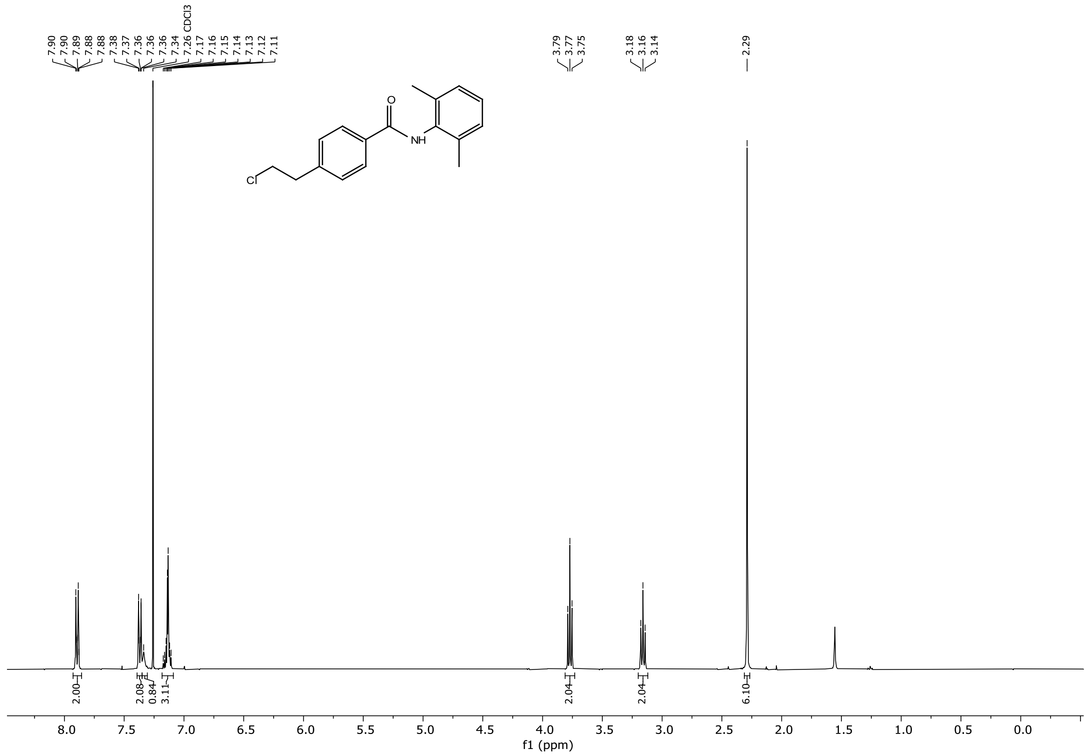

***Supplementary Figure 18. ¹H NMR spectrum of 4-(2-chloroethyl)-N-(2,6-dimethylphenyl)benzamide (B8).***

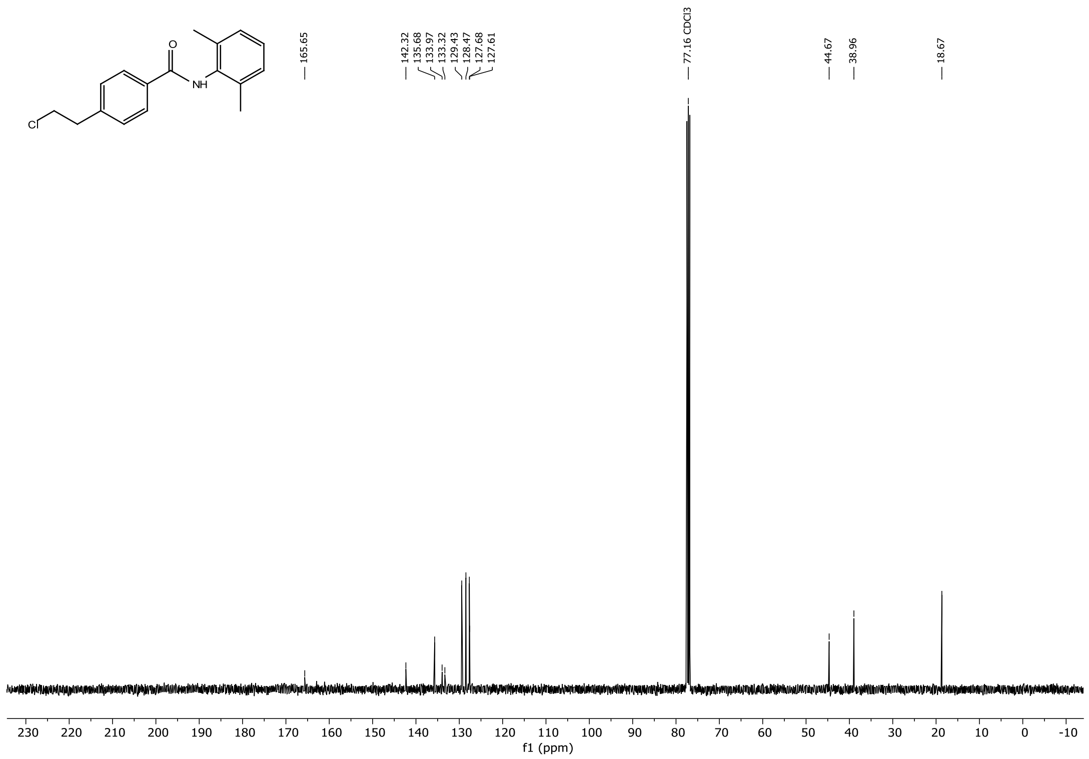

***Supplementary Figure 19. ¹³C NMR spectrum of 4-(2-chloroethyl)-N-(2,6-dimethylphenyl)benzamide (B8).***

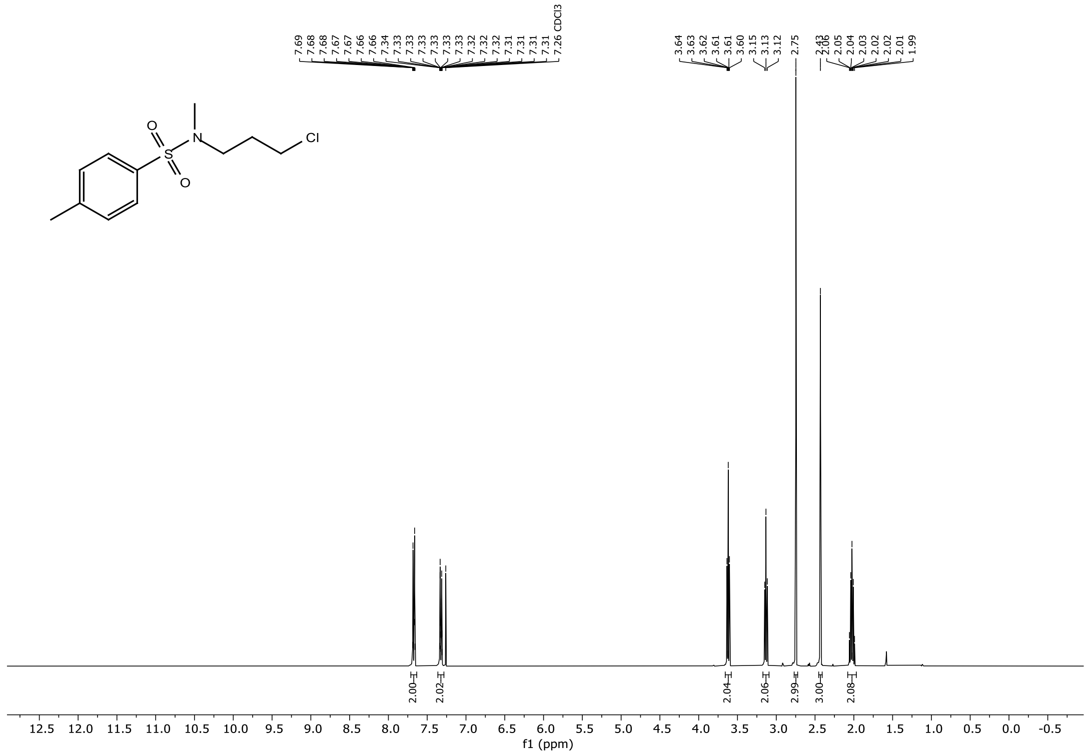

***Supplementary Figure 20. $^{1}H$ NMR spectrum of N-(3-chloropropyl)-N,4-dimethylbenzenesulfonamide (B9).***

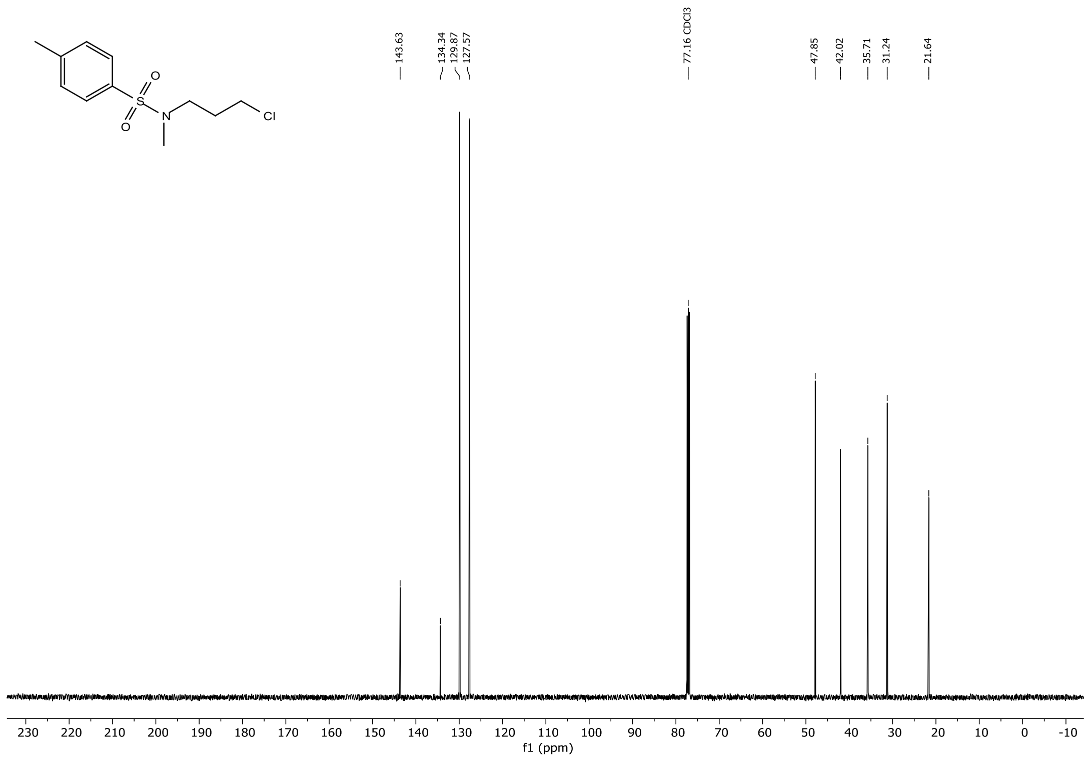

***Supplementary Figure 21. $^{13}C$ NMR spectrum of N-(3-chloropropyl)-N,4-dimethylbenzenesulfonamide (B9).***

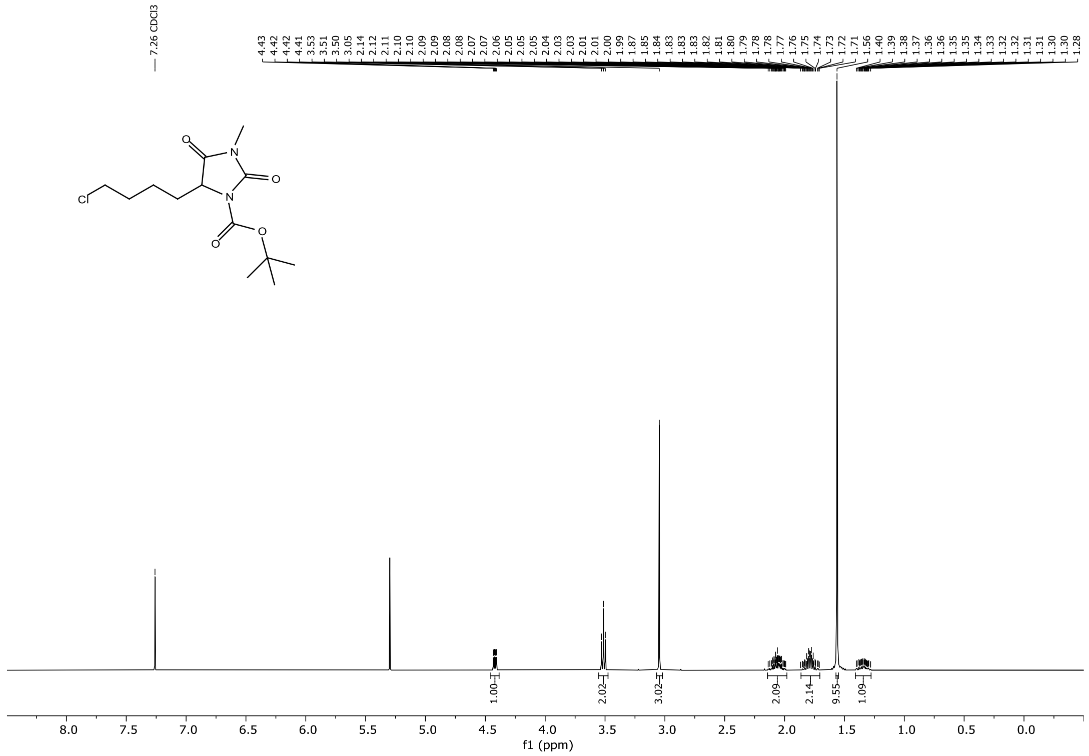


***Supplementary Figure 22. ¹H NMR spectrum of tert-butyl 5-(4-chlorobutyl)-3-methyl-2,4-dioxoimidazolidine-1-carboxylate (B10).***

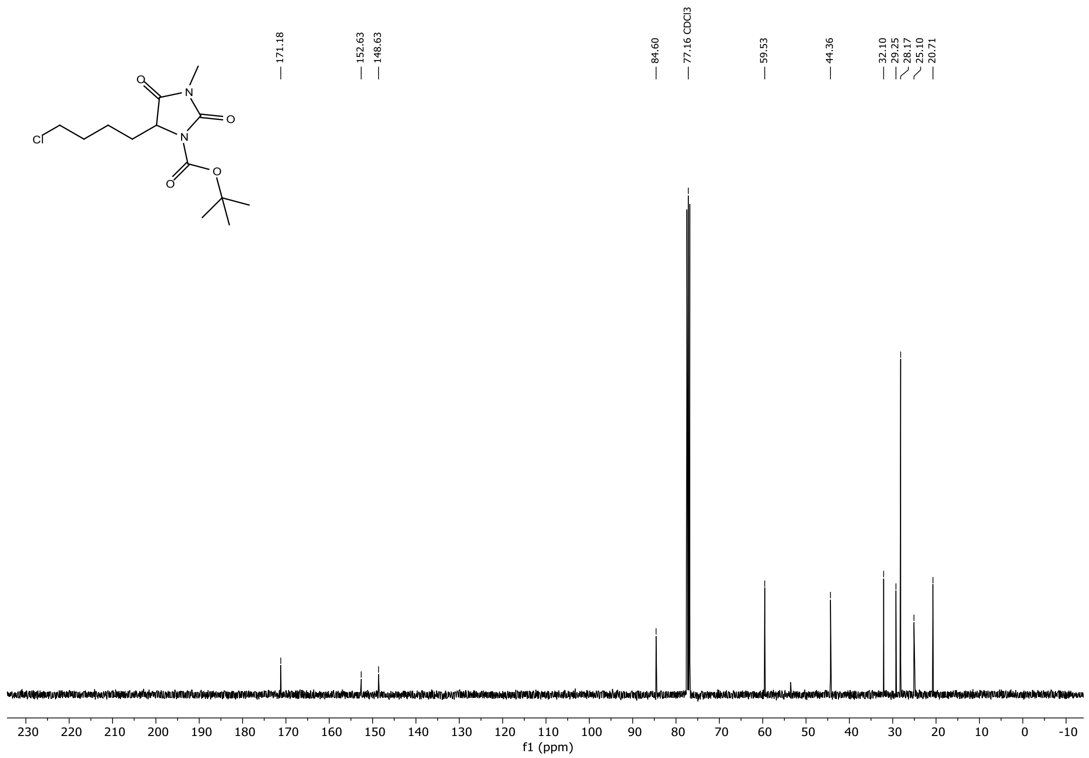


***Supplementary Figure 23. ¹³C NMR spectrum of tert-butyl 5-(4-chlorobutyl)-3-methyl-2,4-dioxoimidazolidine-1-carboxylate (B10).***

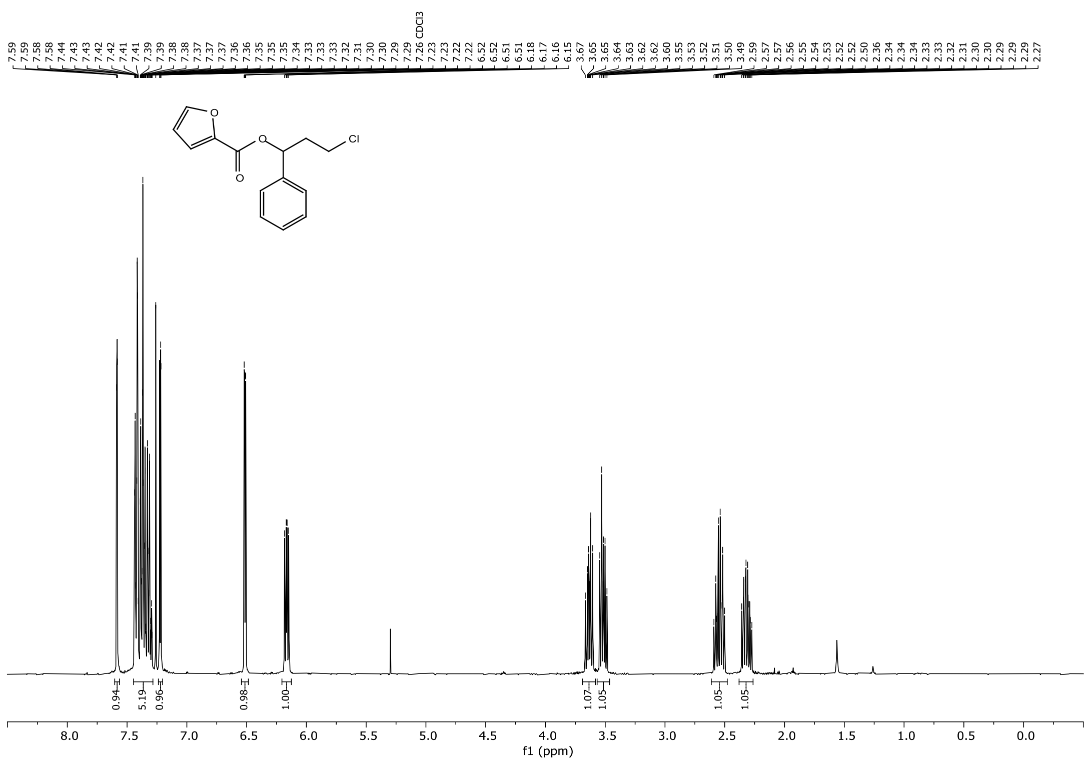


***Supplementary Figure 24. $^{1}$H NMR spectrum of 3-chloro-1-phenylpropyl furan-2-carboxylate (B12).***

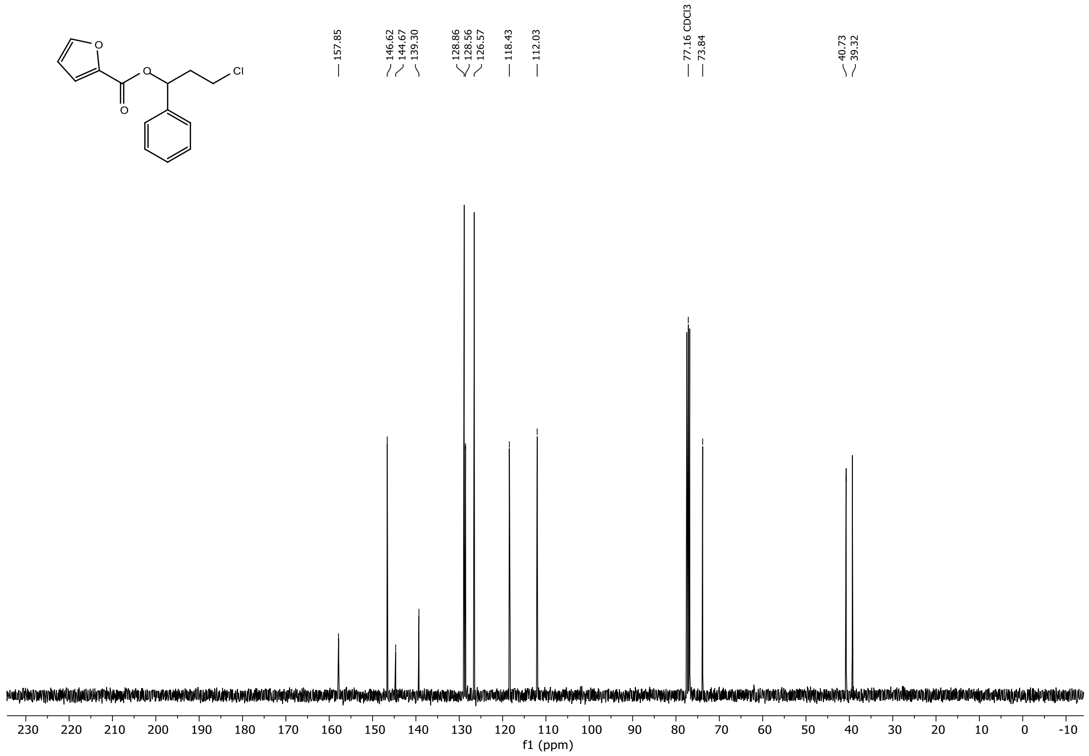


***Supplementary Figure 25. $^{13}$C NMR spectrum of 3-chloro-1-phenylpropyl furan-2-carboxylate (B12).***

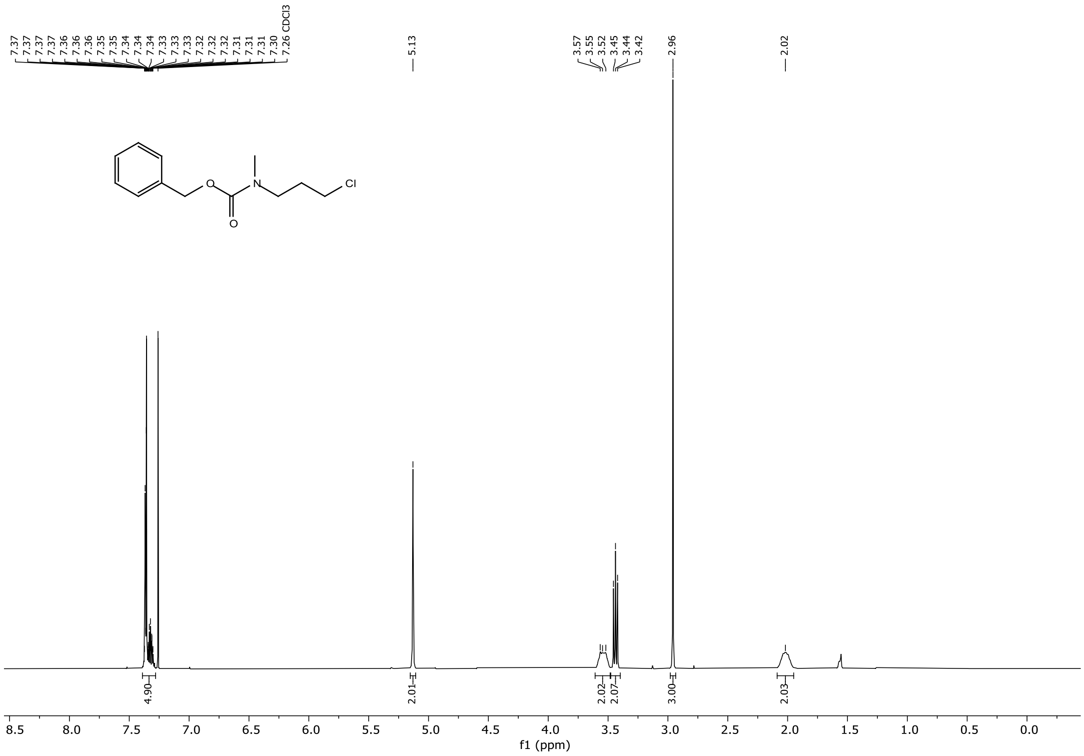


***Supplementary Figure 26. $^{1}$H NMR spectrum of benzyl (3-chloropropyl)(methyl)carbamate (B13).***

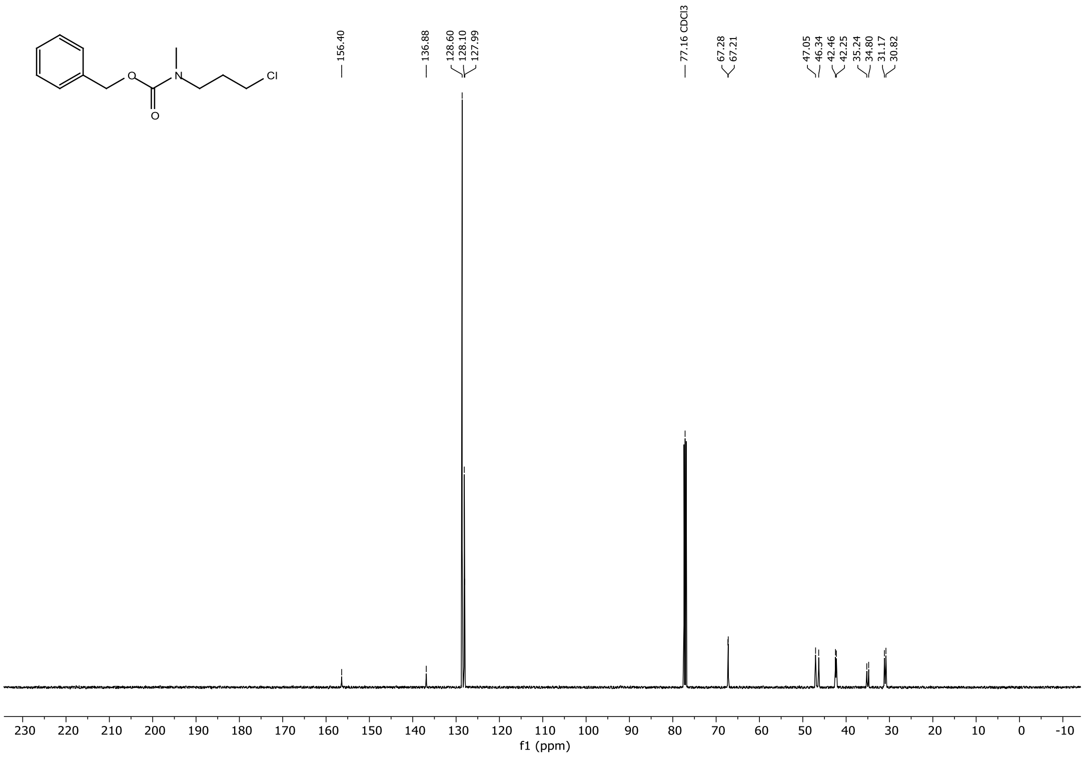


***Supplementary Figure 27. $^{13}$C NMR spectrum of benzyl (3-chloropropyl)(methyl)carbamate (B13).***

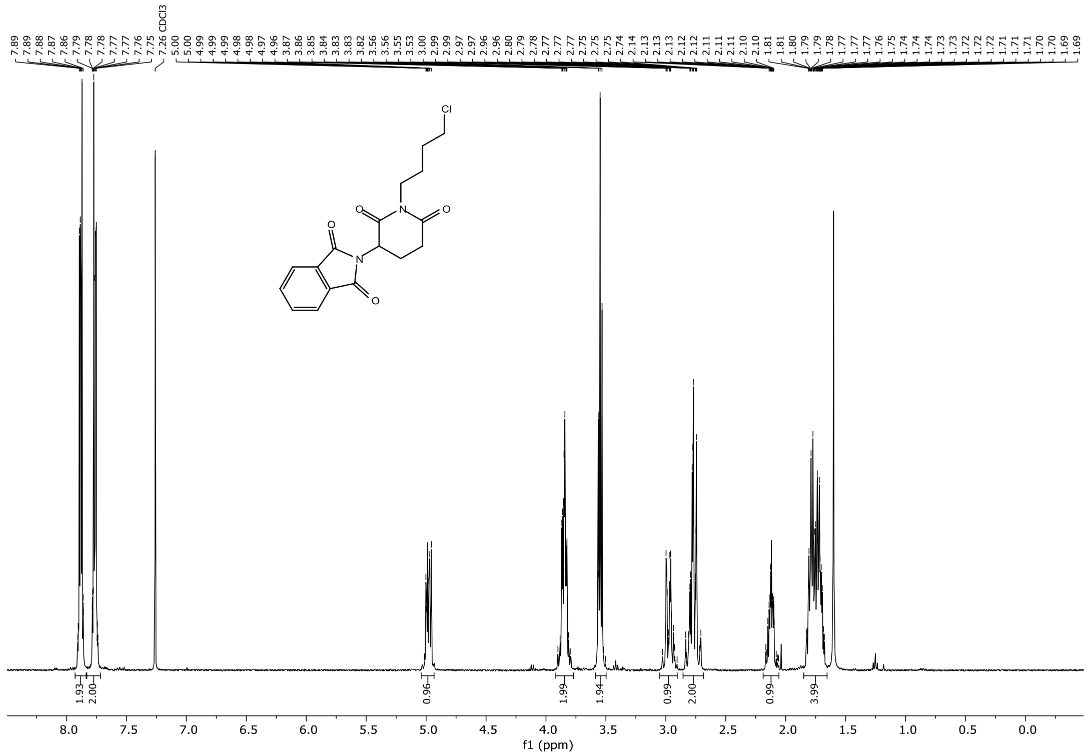


***Supplementary Figure 28. $^{1}H$ NMR spectrum of 2-(1-(4-chlorobutyl)-2,6-dioxopiperidin-3-yl)isoindoline-1,3-dione (B14).***

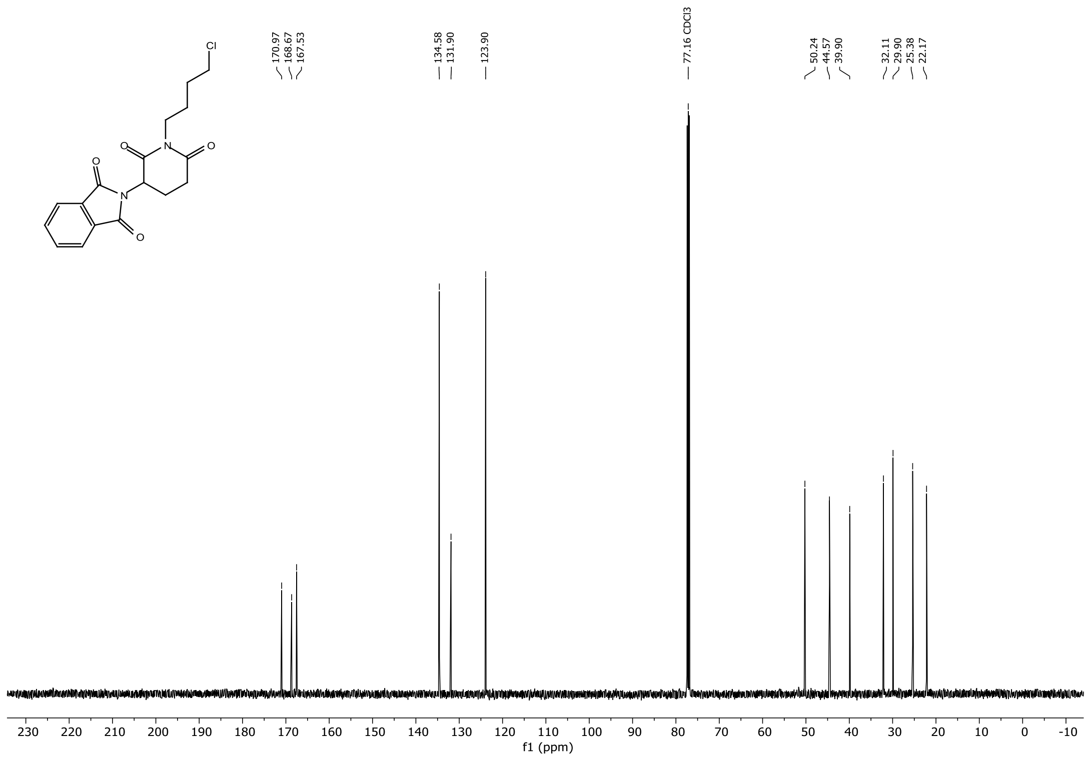


***Supplementary Figure 29. $^{13}C$ NMR spectrum of 2-(1-(4-chlorobutyl)-2,6-dioxopiperidin-3-yl)isoindoline-1,3-dione (B14).***

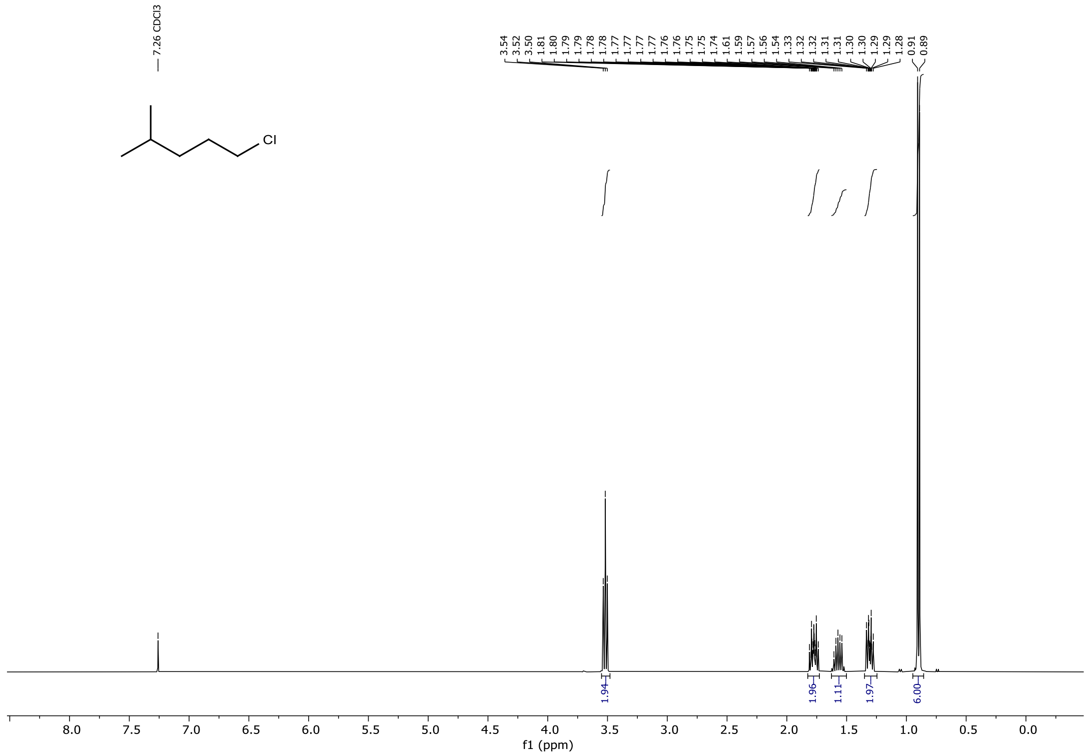


***Supplementary Figure 30. $^{1}H$ NMR spectrum of 1-chloro-4-methylpentane (B15).***

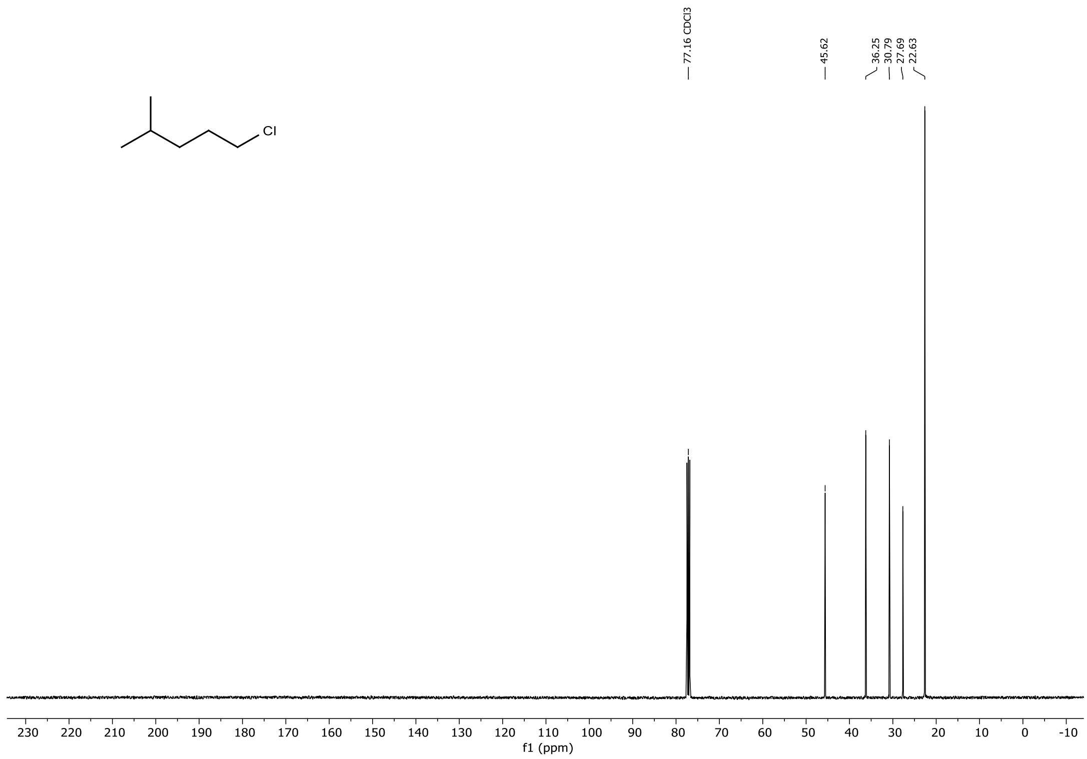


***Supplementary Figure 31. $^{13}C$ NMR spectrum of 1-chloro-4-methylpentane (B15).***

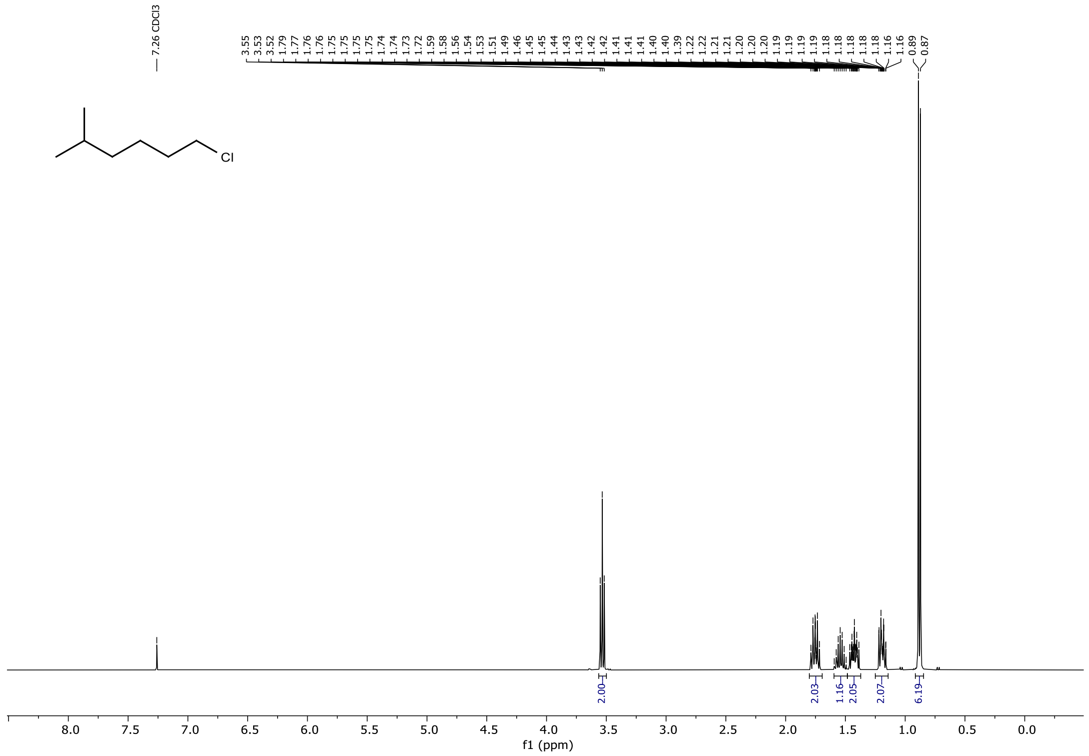


***Supplementary Figure 32. $^{1}$H NMR spectrum of 1-chloro-5-methylhexane (B16).***

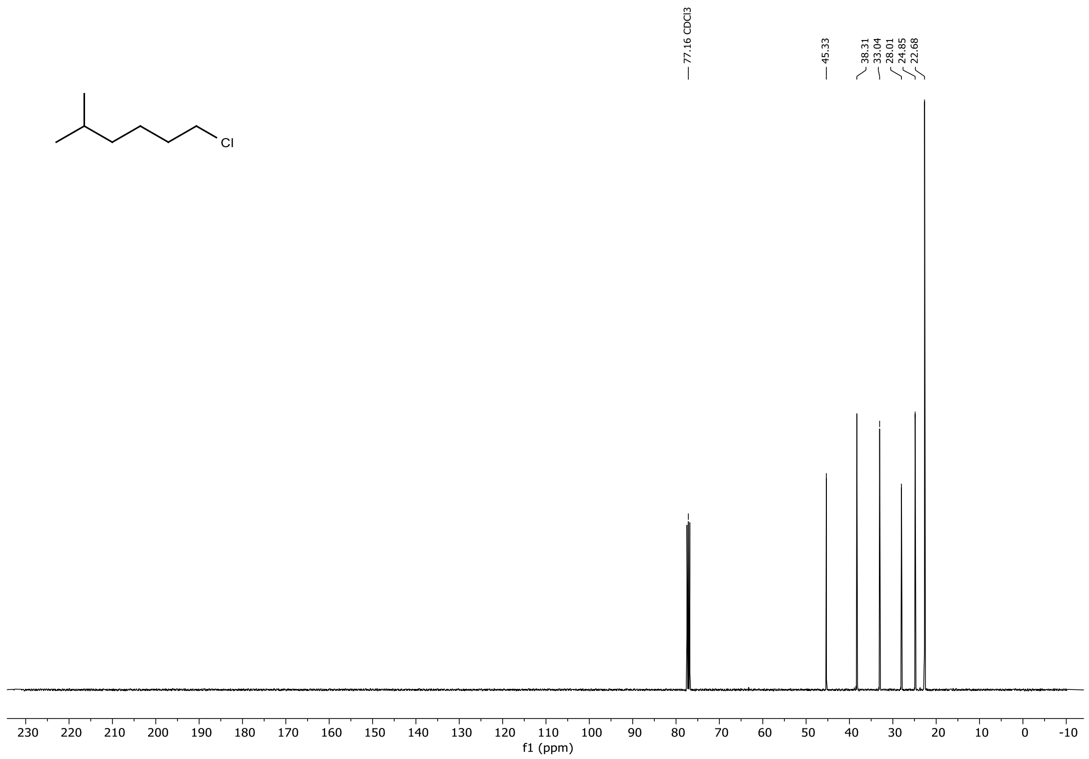


***Supplementary Figure 33. $^{13}$C NMR spectrum of 1-chloro-5-methylhexane (B16).***

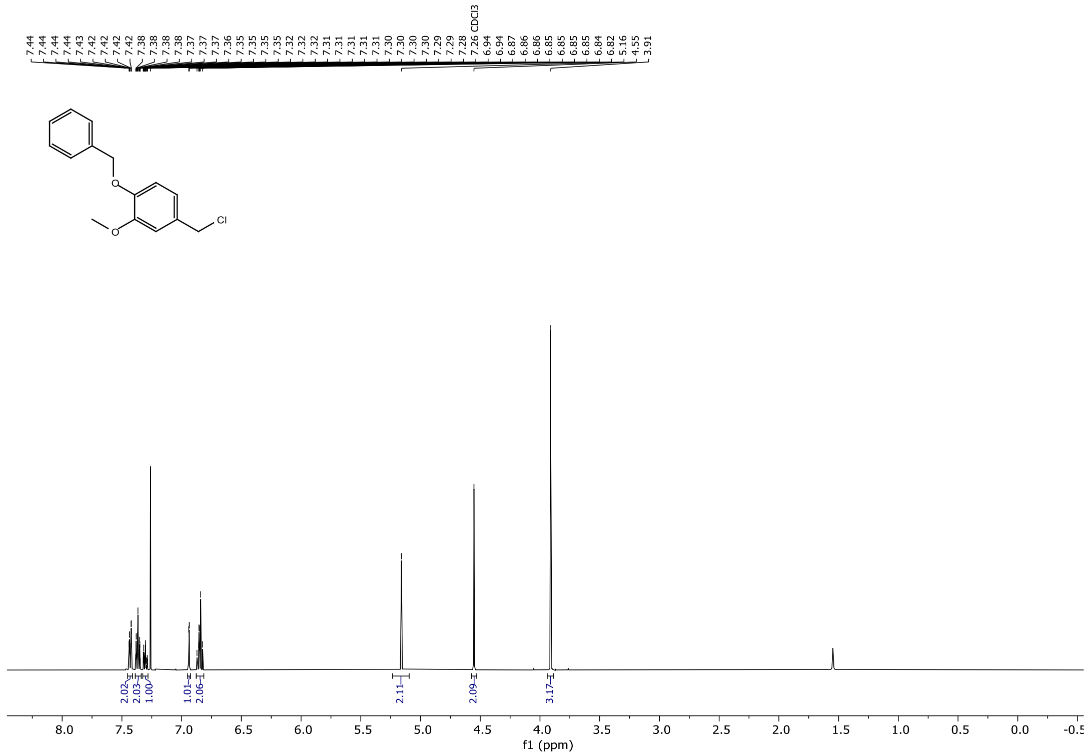


***Supplementary Figure 34. ¹H NMR spectrum of 1-(benzyloxy)-4-(chloromethyl)-2-methoxybenzene (B17).***

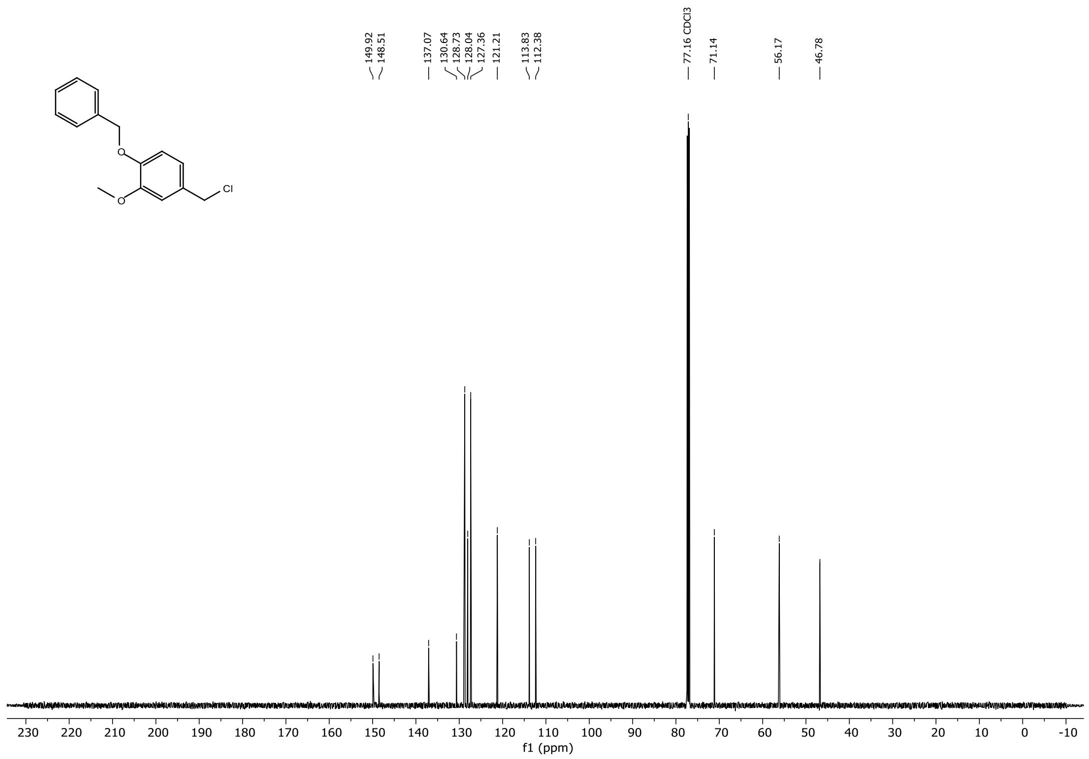


***Supplementary Figure 35. ¹³C NMR spectrum of 1-(benzyloxy)-4-(chloromethyl)-2-methoxybenzene (B17).***

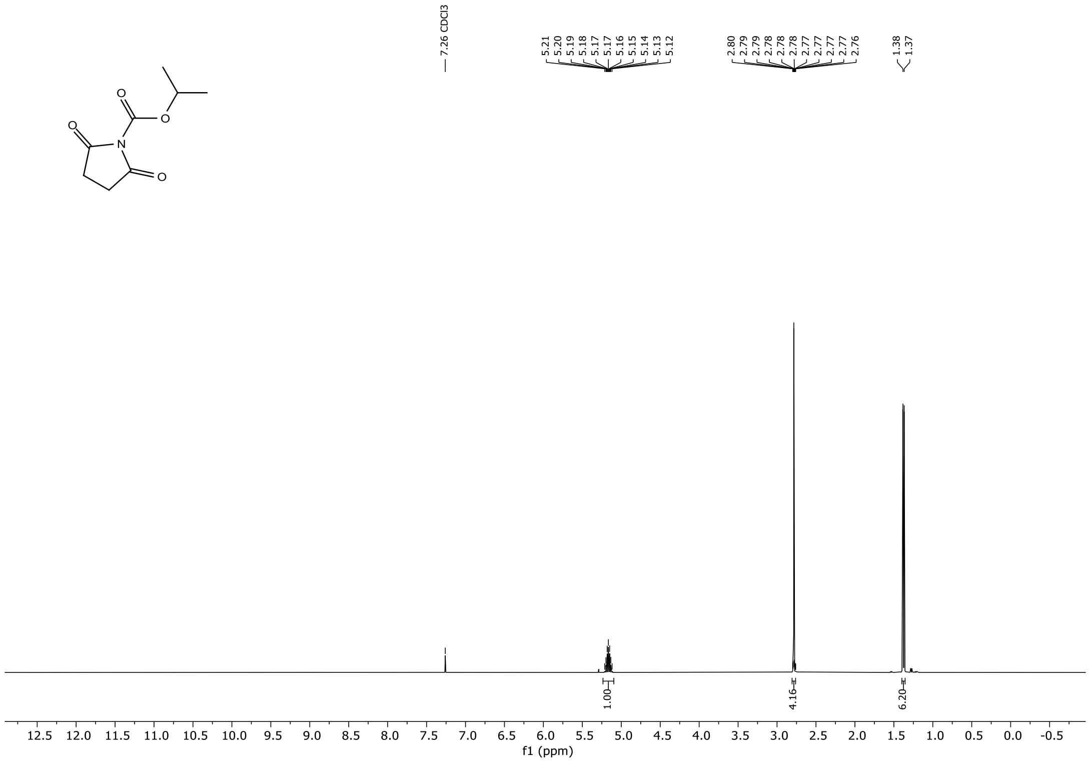


***Supplementary Figure 36. $^1$H NMR spectrum of isopropyl 2,5-dioxopyrrolidine-1-carboxylate (A1a).***

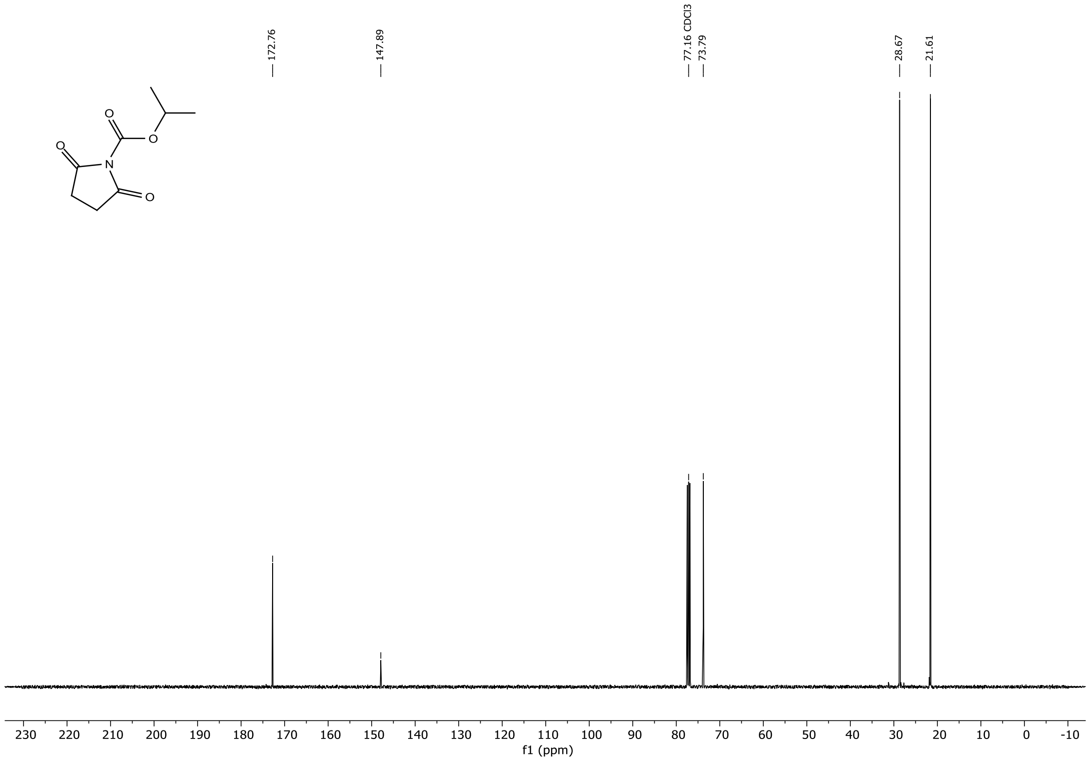


***Supplementary Figure 37. $^{13}$C NMR spectrum of isopropyl 2,5-dioxopyrrolidine-1-carboxylate (A1a).***

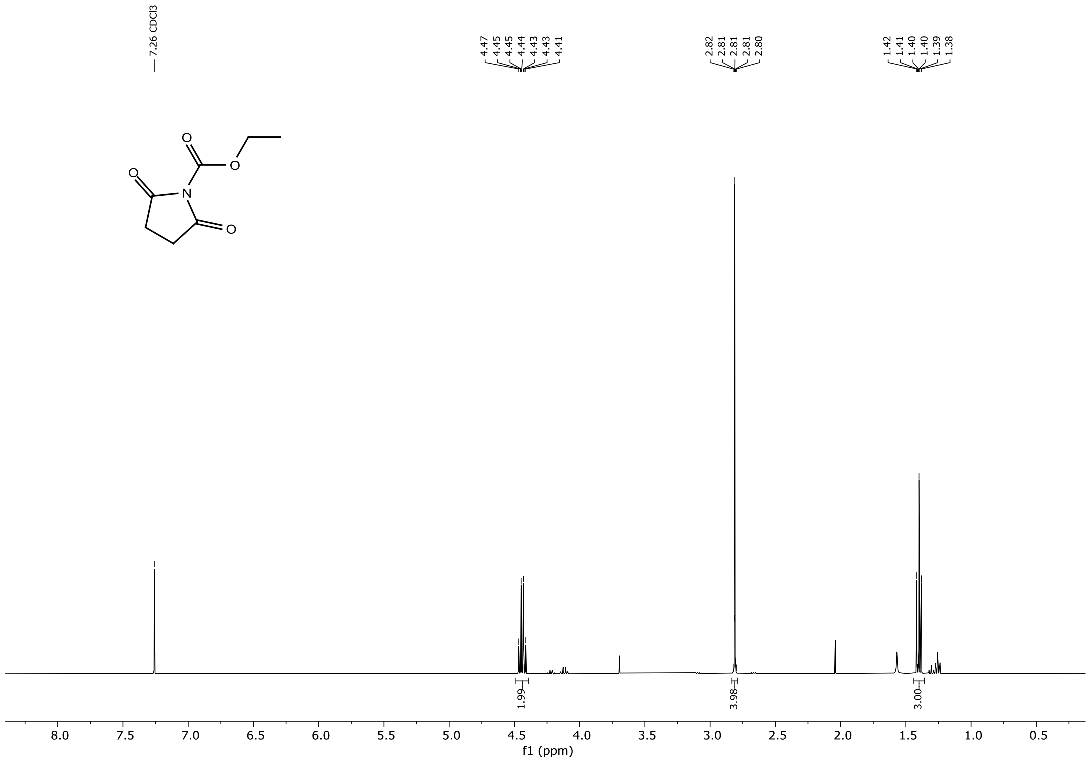


***Supplementary Figure 38. ¹H NMR spectrum of ethyl 2,5-dioxopyrrolidine-1-carboxylate (A1b).***

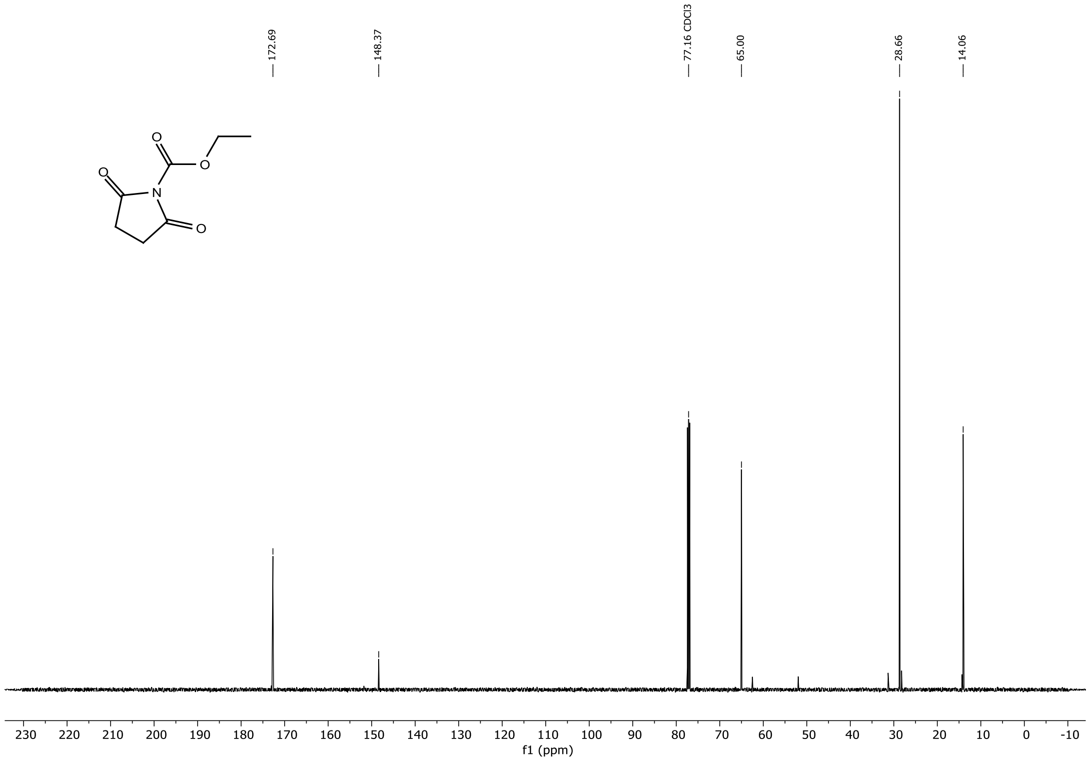


***Supplementary Figure 39. ¹³C NMR spectrum of ethyl 2,5-dioxopyrrolidine-1-carboxylate (A1b).***

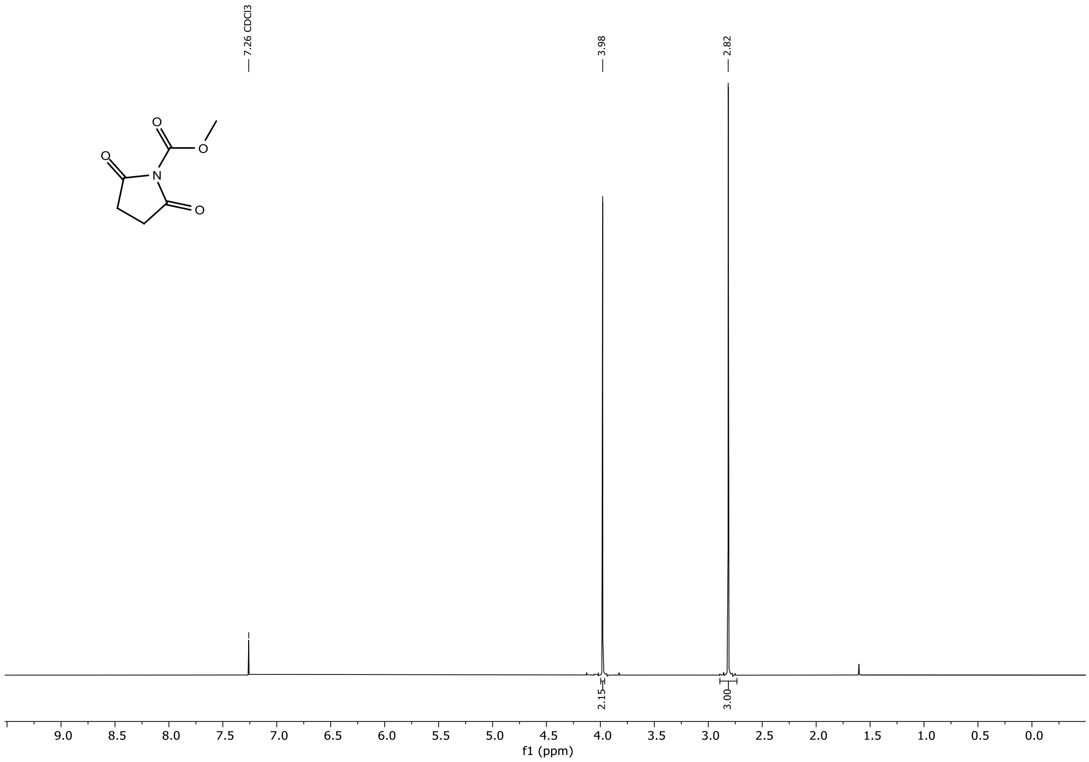


***Supplementary Figure 40.*** ***$^{1}$H NMR spectrum of methyl 2,5-dioxopyrrolidine-1-carboxylate (A1c).***

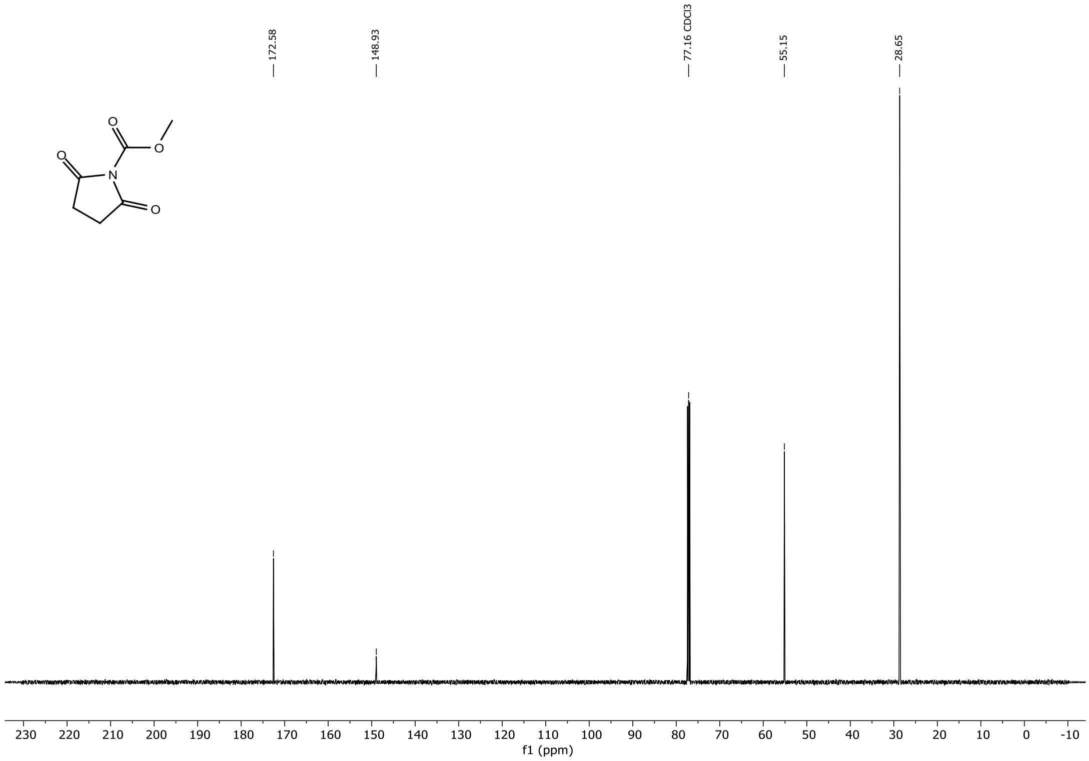


***Supplementary Figure 41.*** ***$^{13}$C NMR spectrum of methyl 2,5-dioxopyrrolidine-1-carboxylate (A1c).***

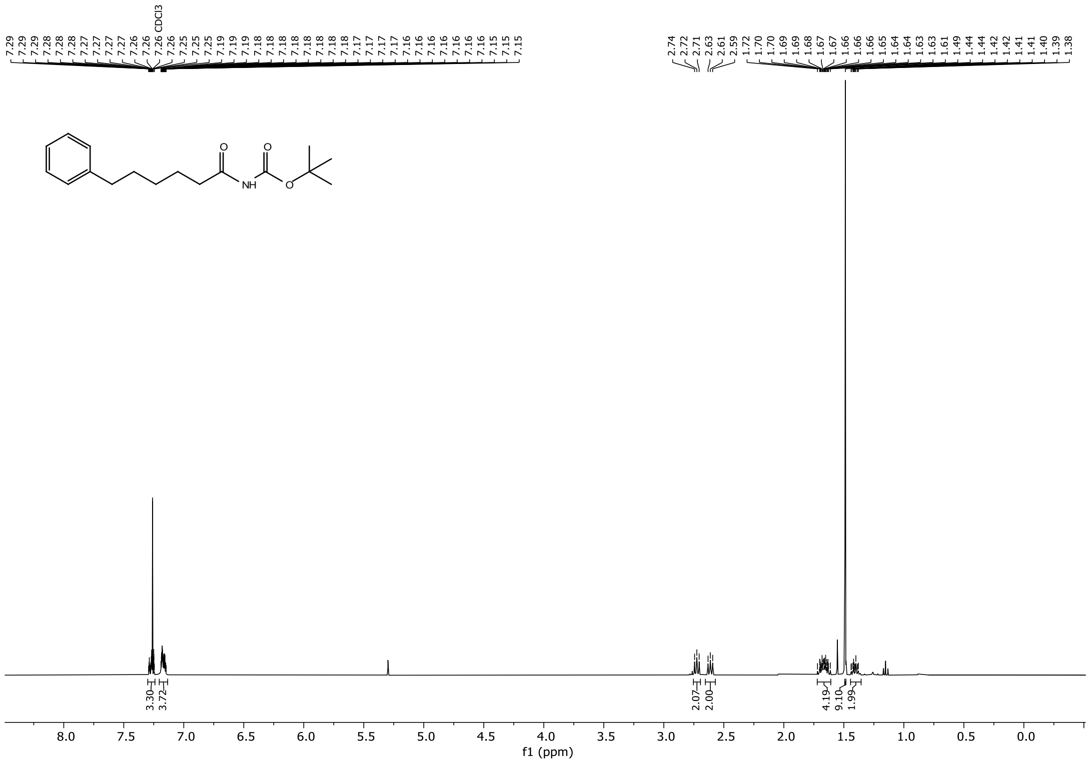

***Supplementary Figure 42. ¹H NMR spectrum of C1.***

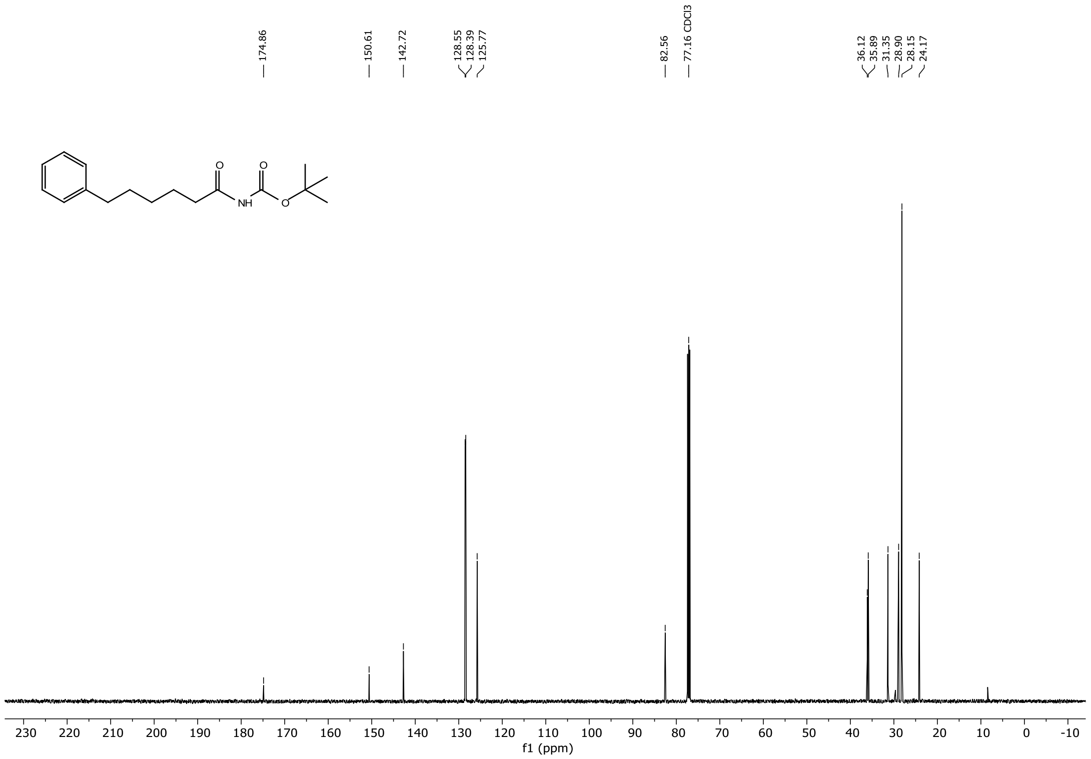

***Supplementary Figure 43. ¹³C NMR spectrum of C1.***

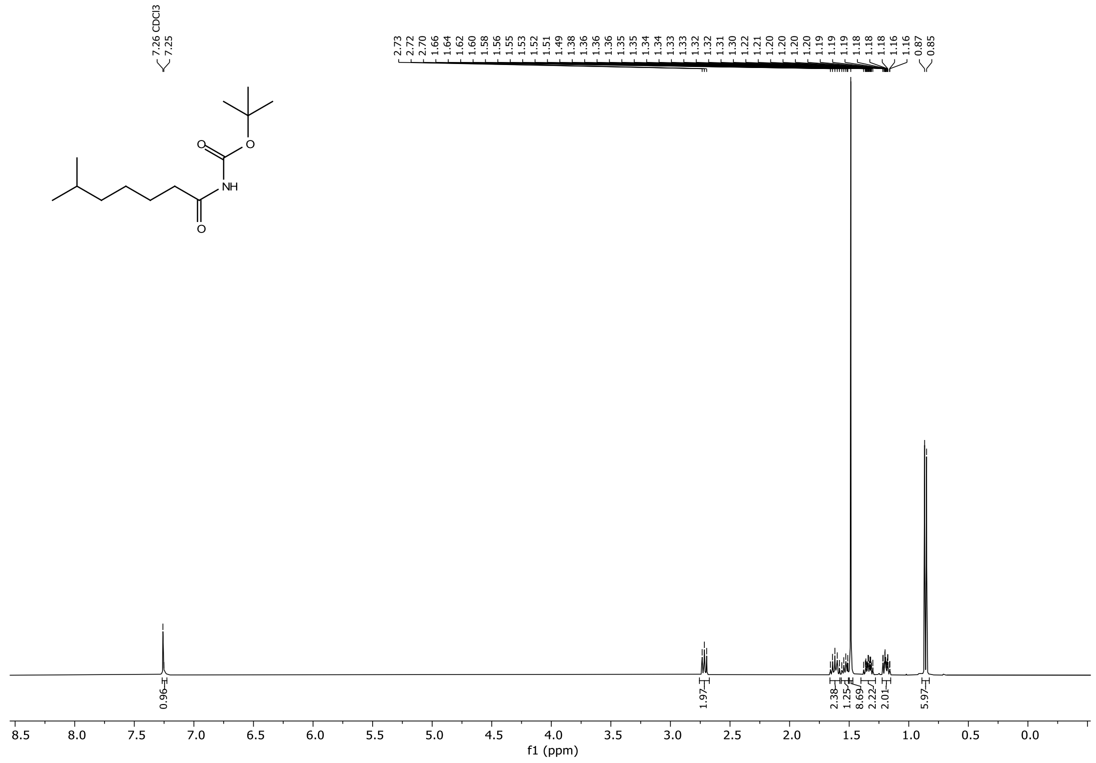


***Supplementary Figure 44.*** ***$^{1}$H NMR spectrum of C2.***

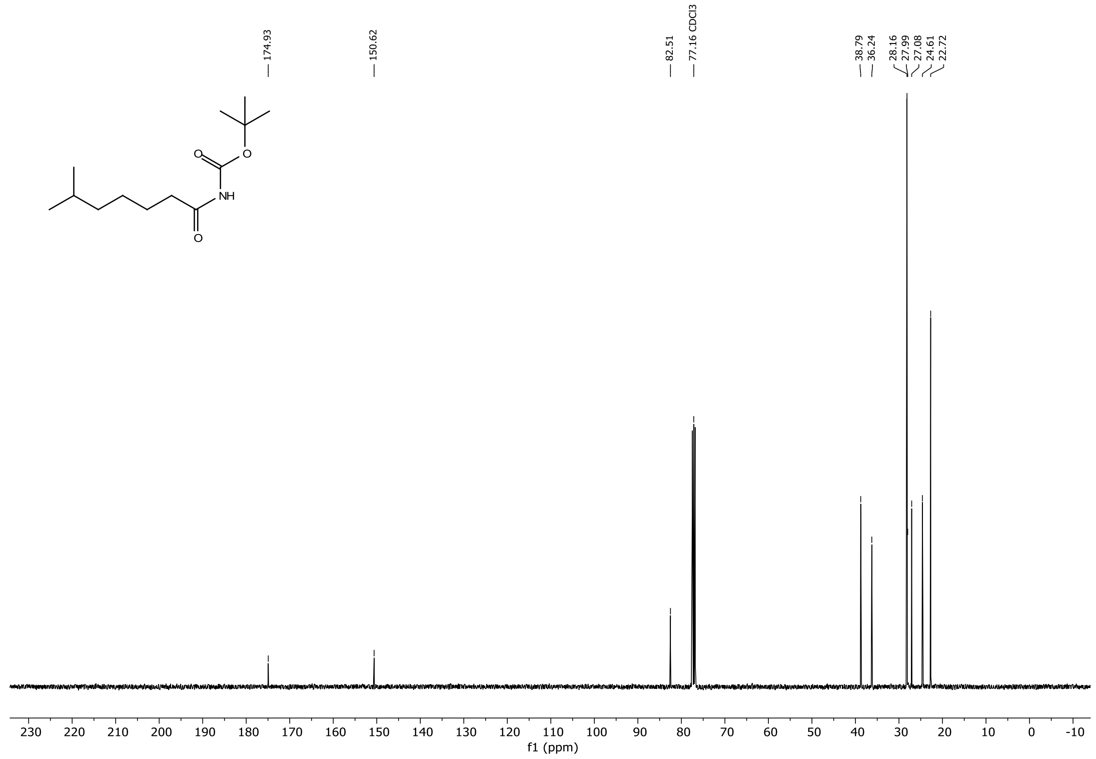


***Supplementary Figure 45.*** ***$^{13}$C NMR spectrum of C2.***

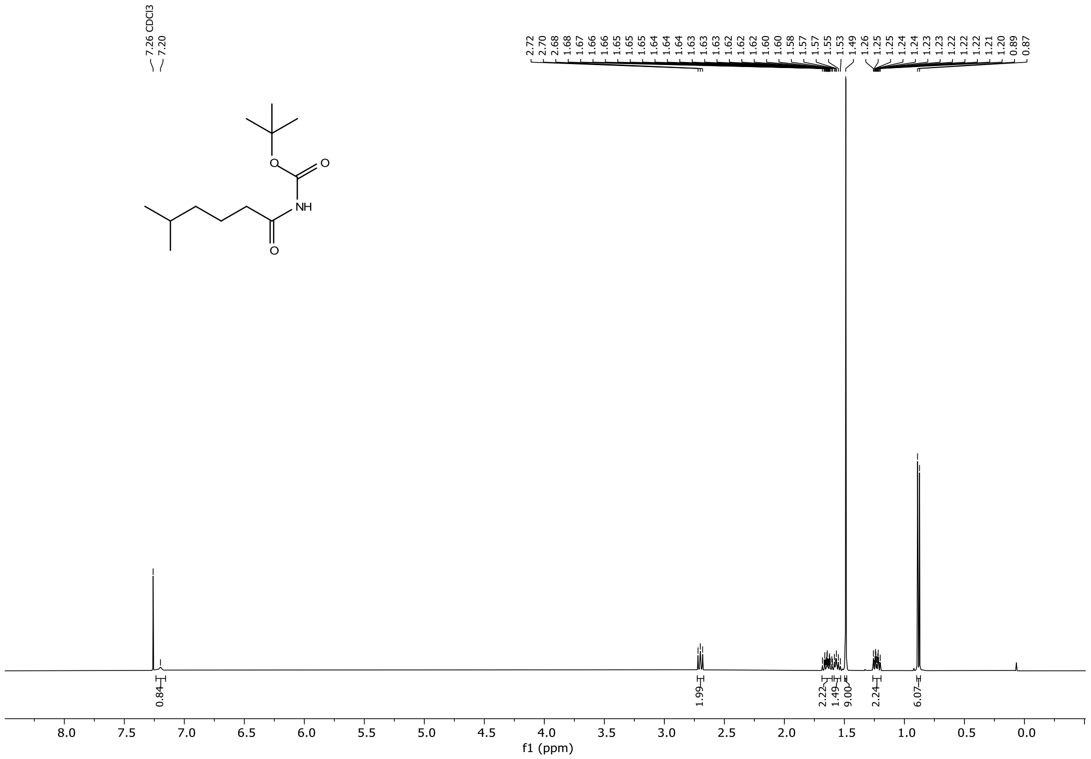


***Supplementary Figure 46. $^{1}H$ NMR spectrum of C3.***

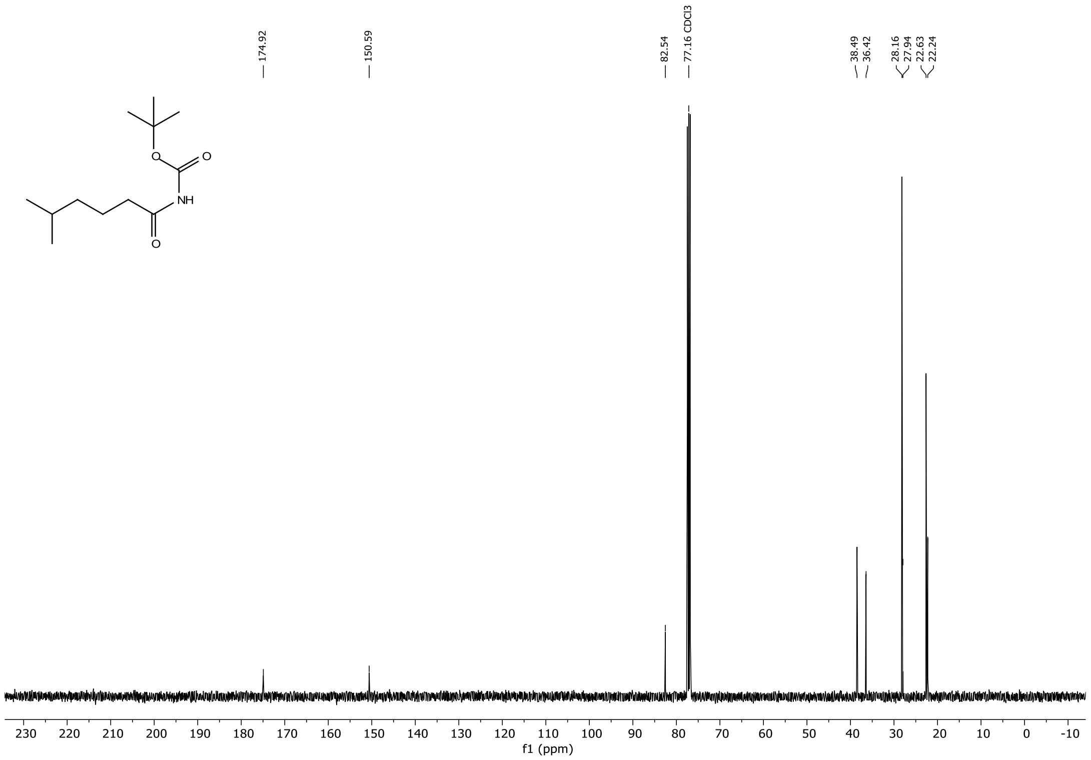


***Supplementary Figure 47. $^{13}C$ NMR spectrum of C3.***

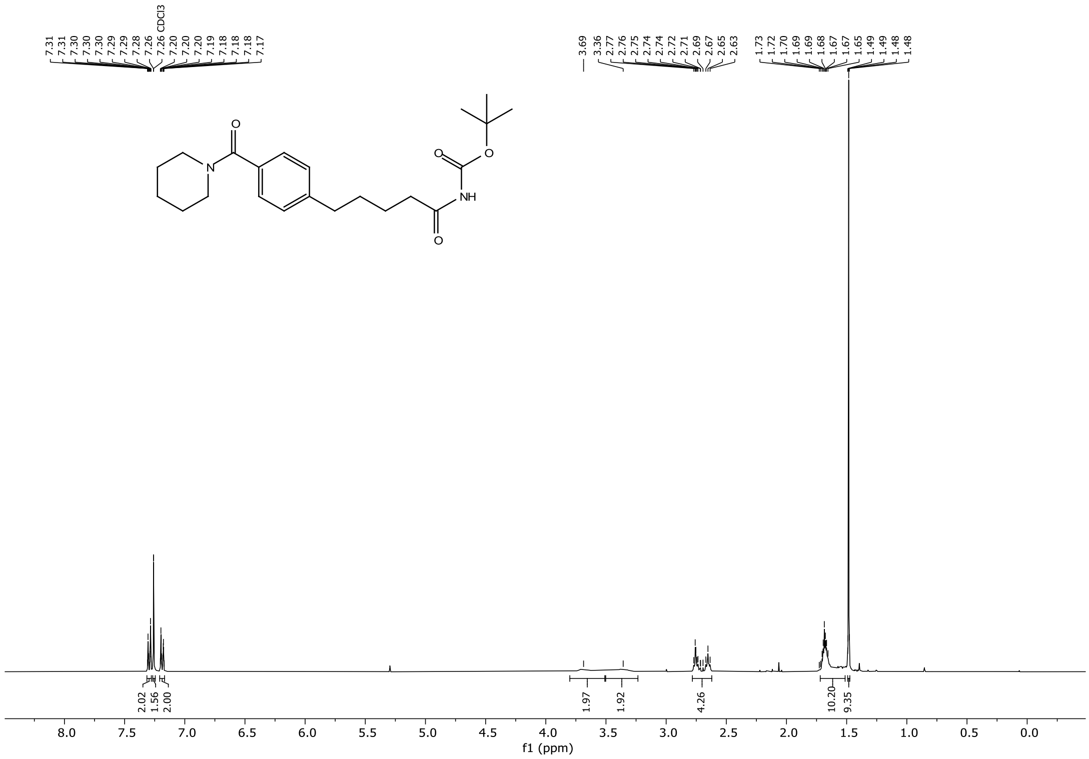

***Supplementary Figure 48. ¹H NMR spectrum of C4.***

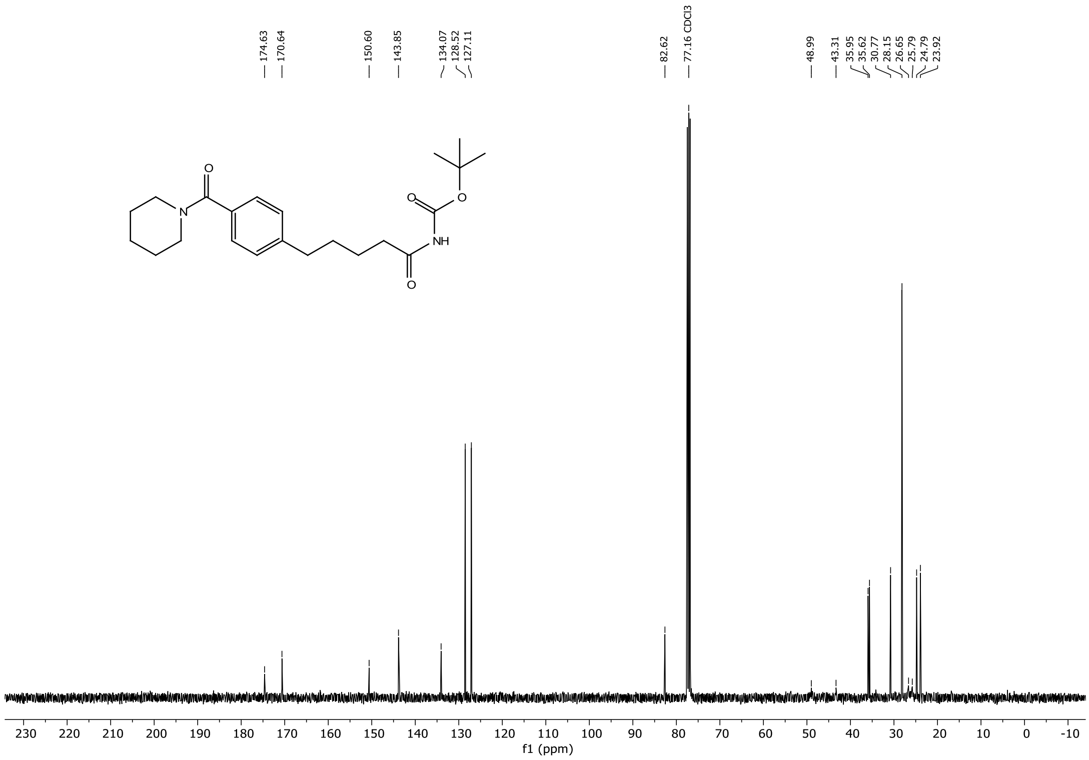

***Supplementary Figure 49. ¹³C NMR spectrum of C4.***

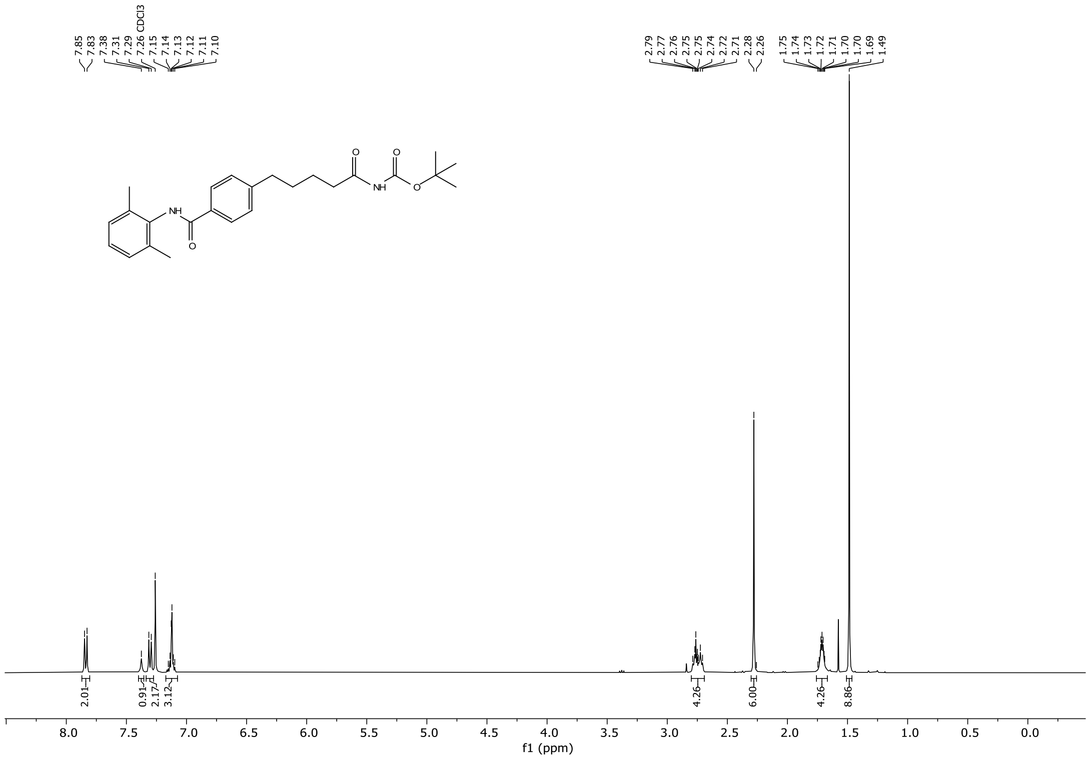

***Supplementary Figure 50. ¹H NMR spectrum of C5.***

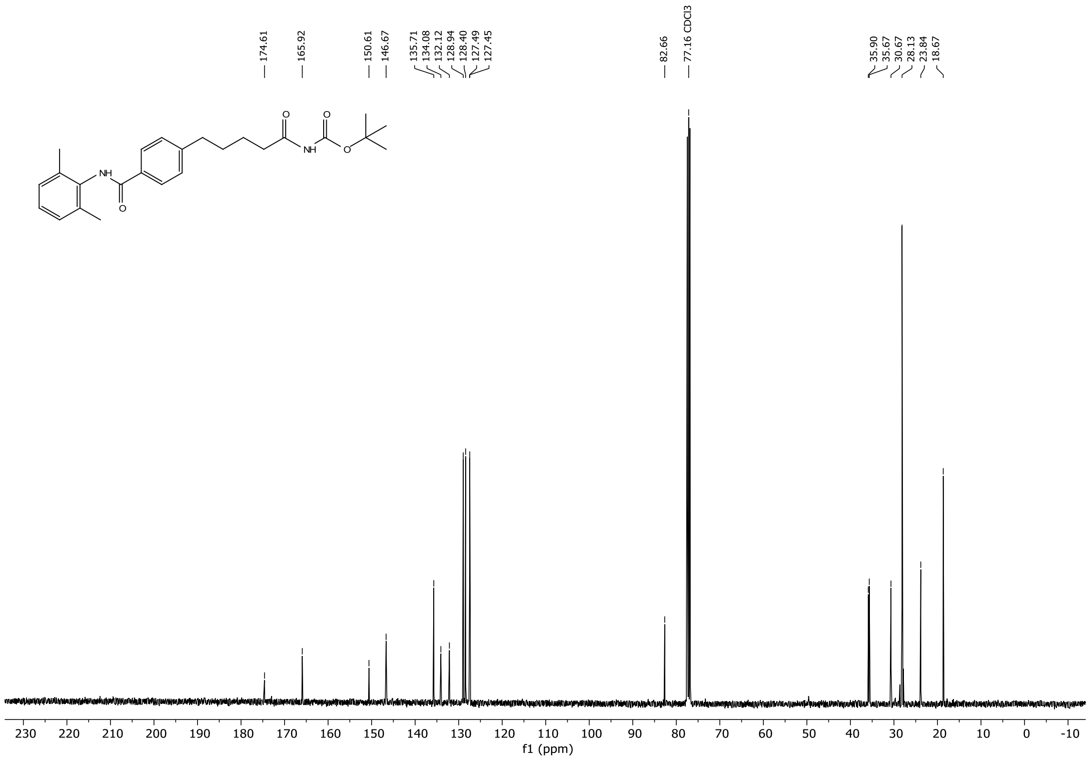

***Supplementary Figure 51. ¹³C NMR spectrum of C5.***

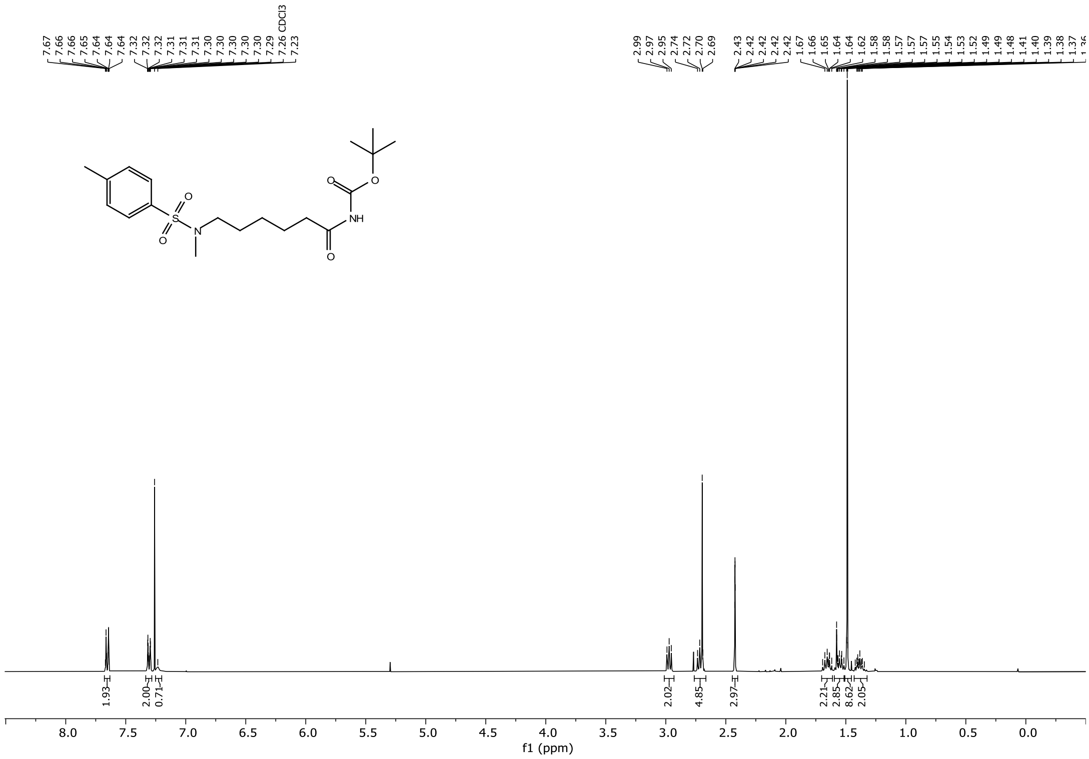

***Supplementary Figure 52. ¹H NMR spectrum of C6.***

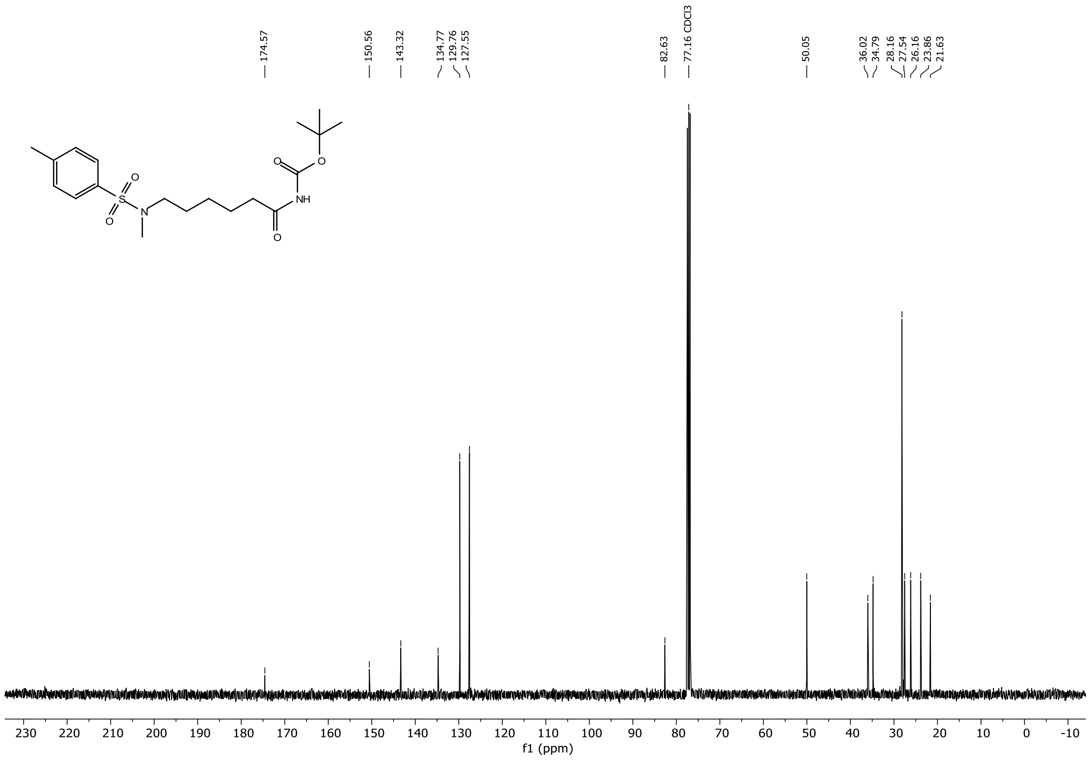

***Supplementary Figure 53. ¹³C NMR spectrum of C6.***

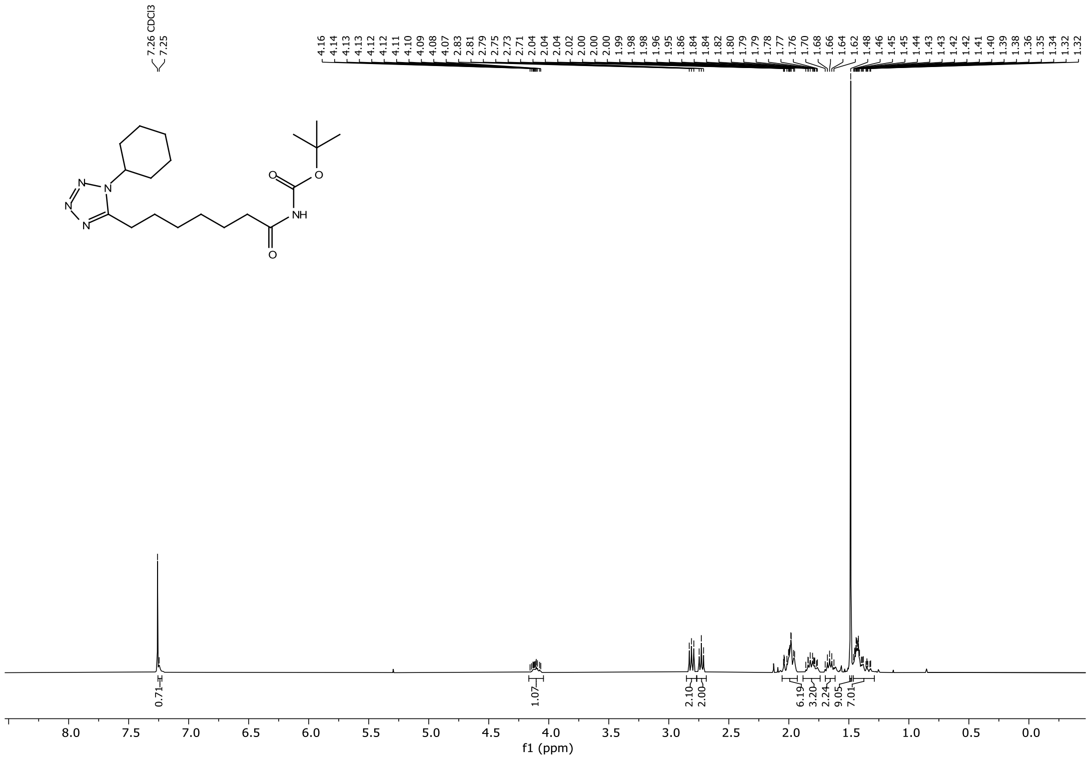


***Supplementary Figure 54. $^{1}H$ NMR spectrum of C7.***

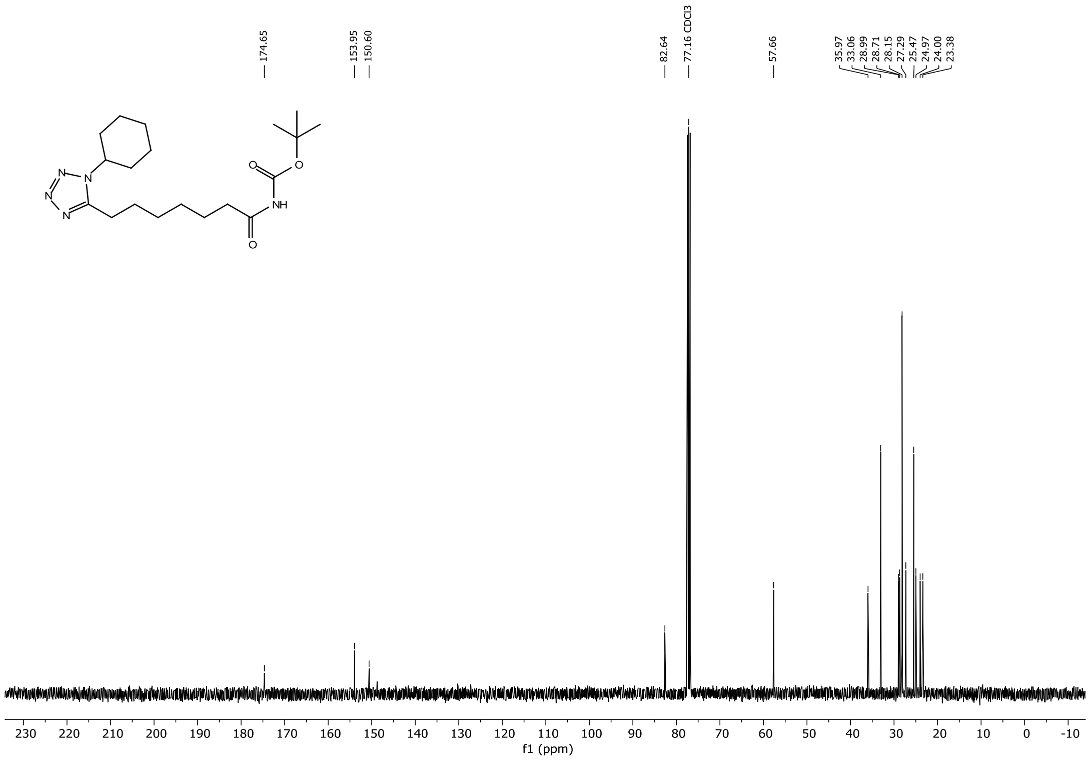


***Supplementary Figure 55. $^{13}C$ NMR spectrum of C7.***

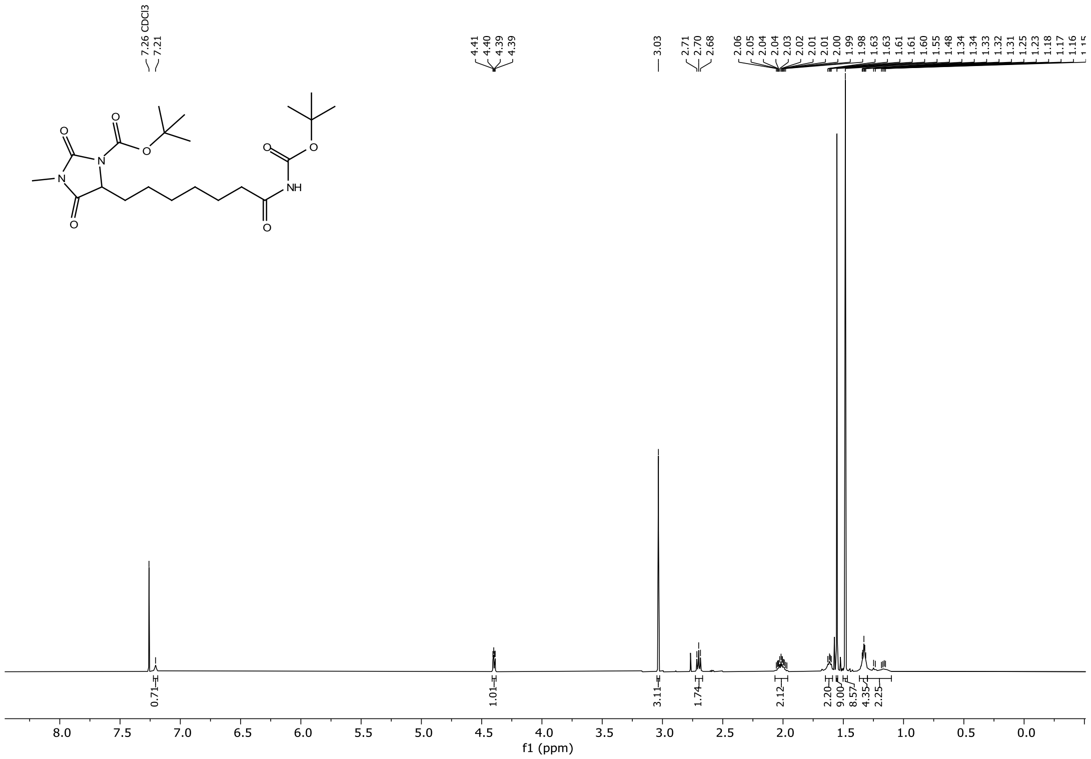


***Supplementary Figure 56. $^{1}H$ NMR spectrum of C8.***

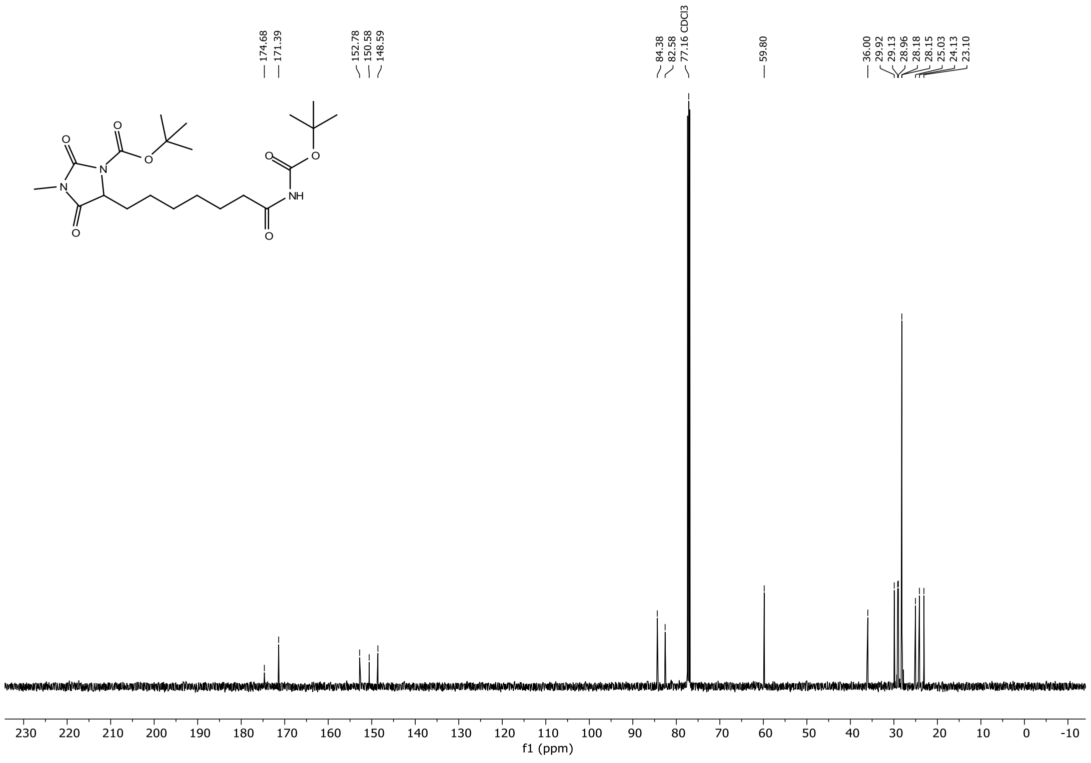


***Supplementary Figure 57. $^{13}C$ NMR spectrum of C8.***

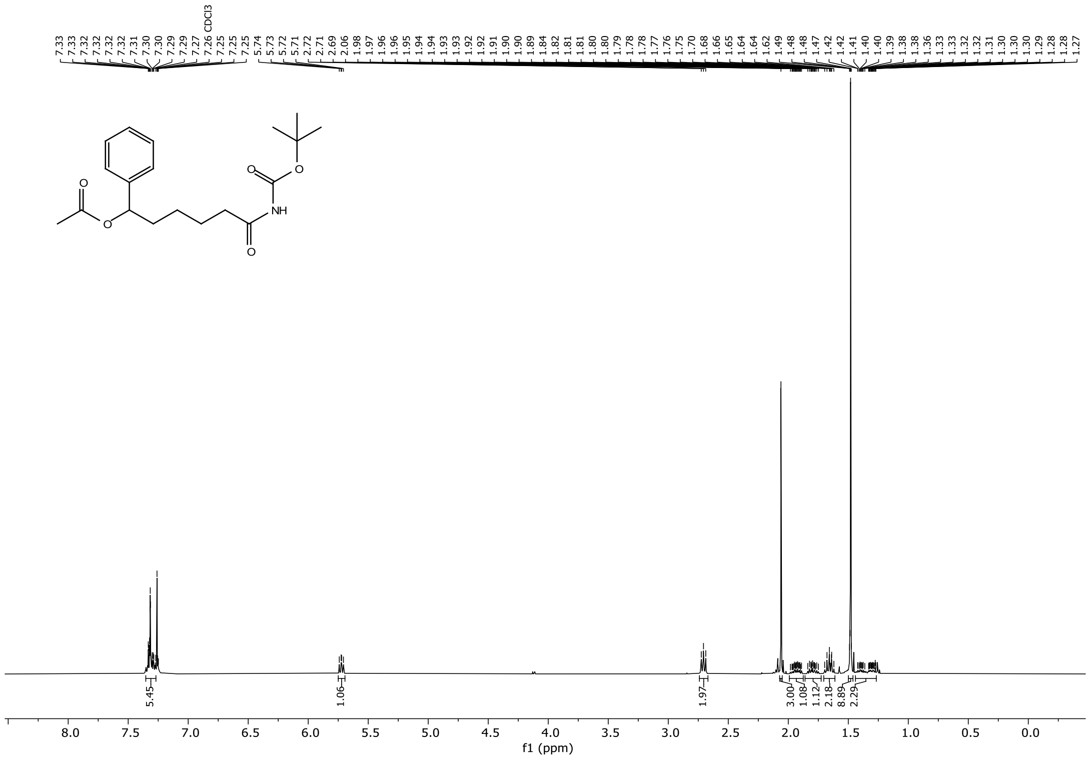

***Supplementary Figure 58. ¹H NMR spectrum of C9.***

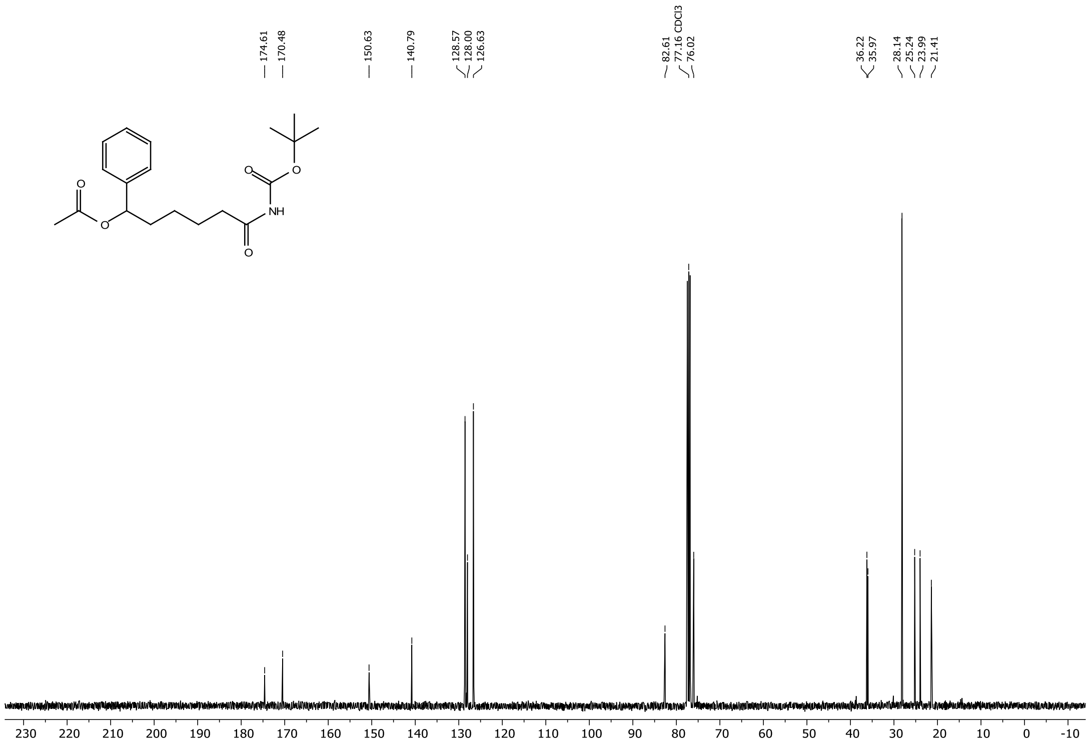

***Supplementary Figure 59. ¹³C NMR spectrum of C9.***

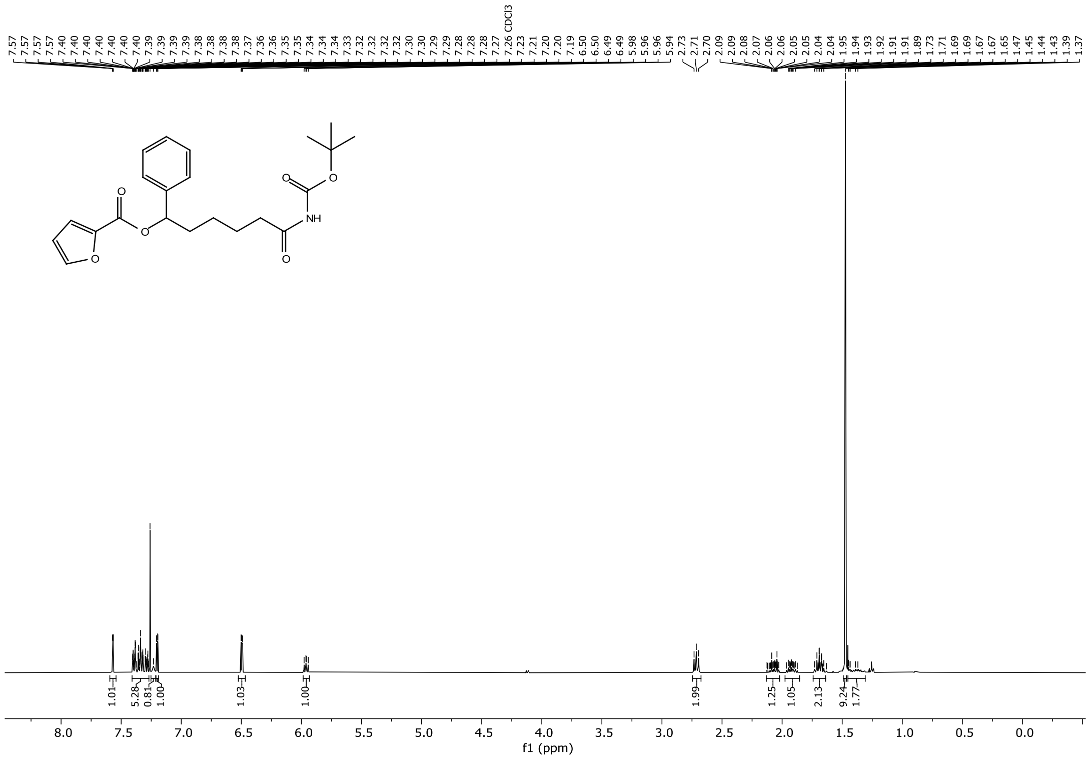

***Supplementary Figure 60. ¹H NMR spectrum of C10.***

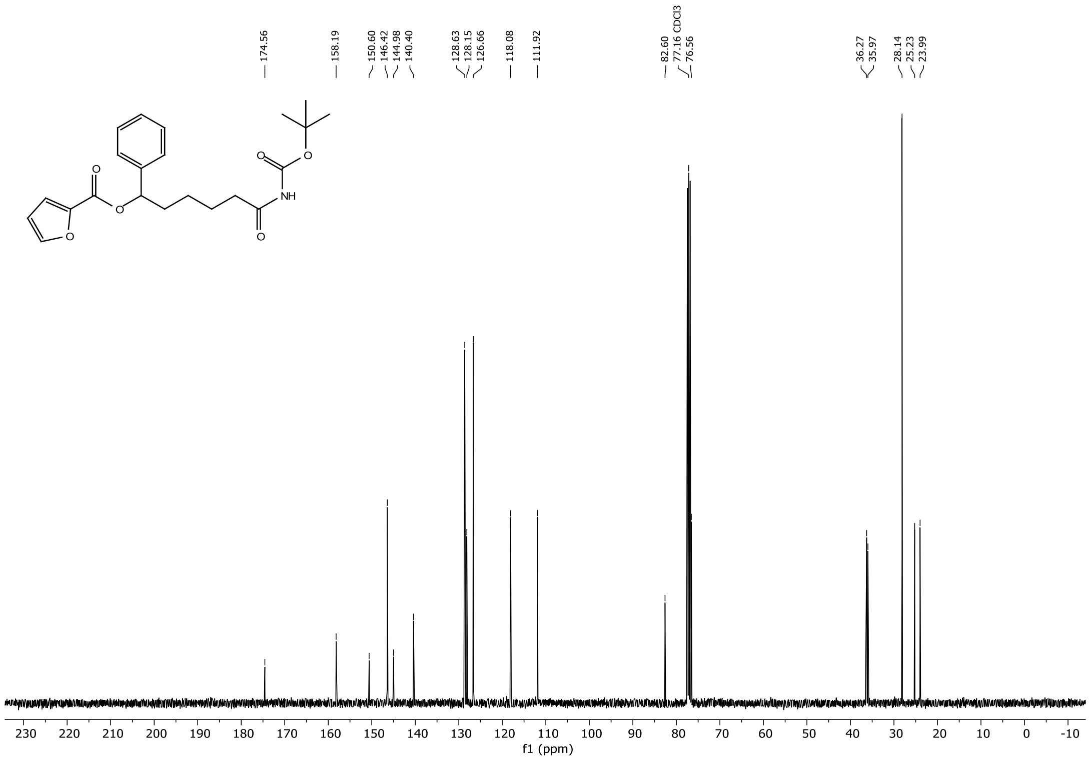

***Supplementary Figure 61. ¹³C NMR spectrum of C10.***

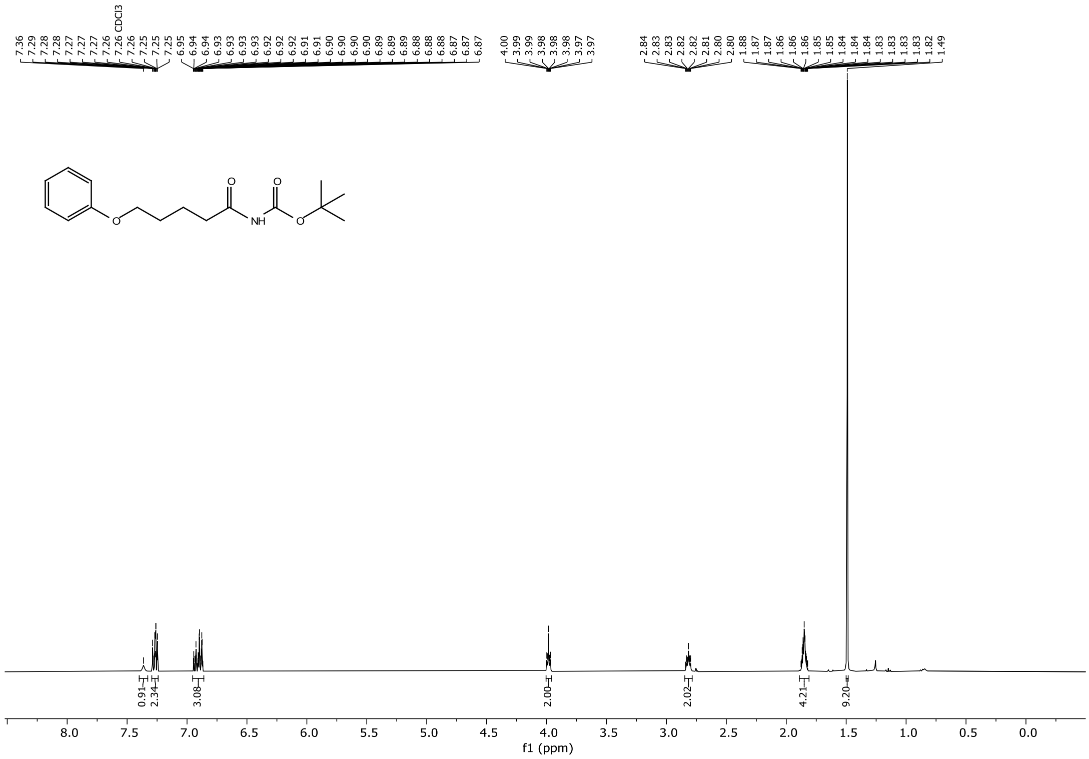

***Supplementary Figure 62. ¹H NMR spectrum of C11.***

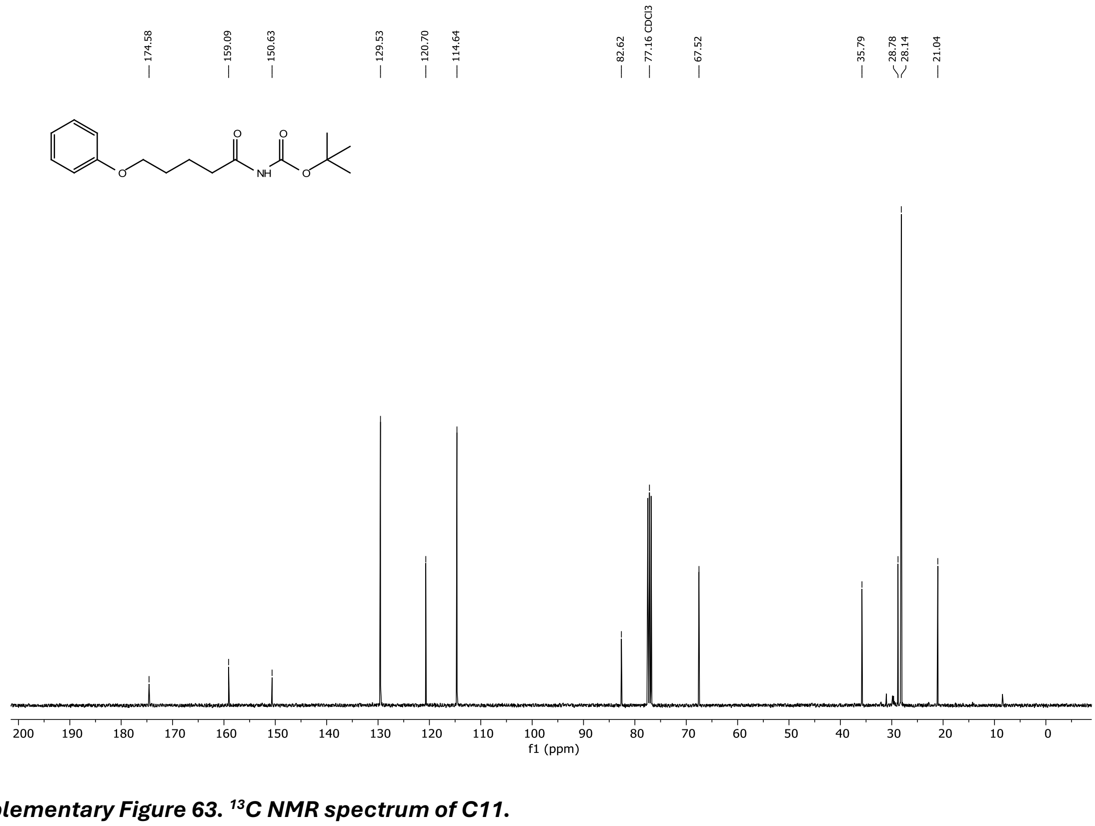

***Supplementary Figure 63. ¹³C NMR spectrum of C11.***

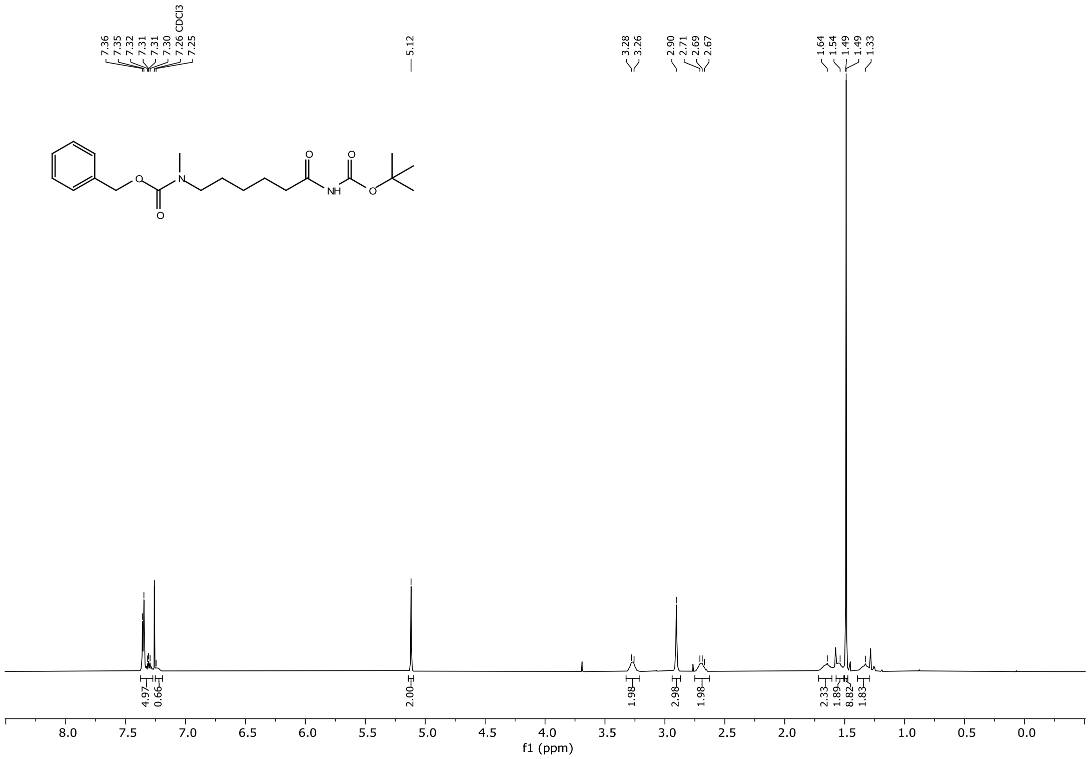


***Supplementary Figure 64. $^{1}H$ NMR spectrum of C12.***

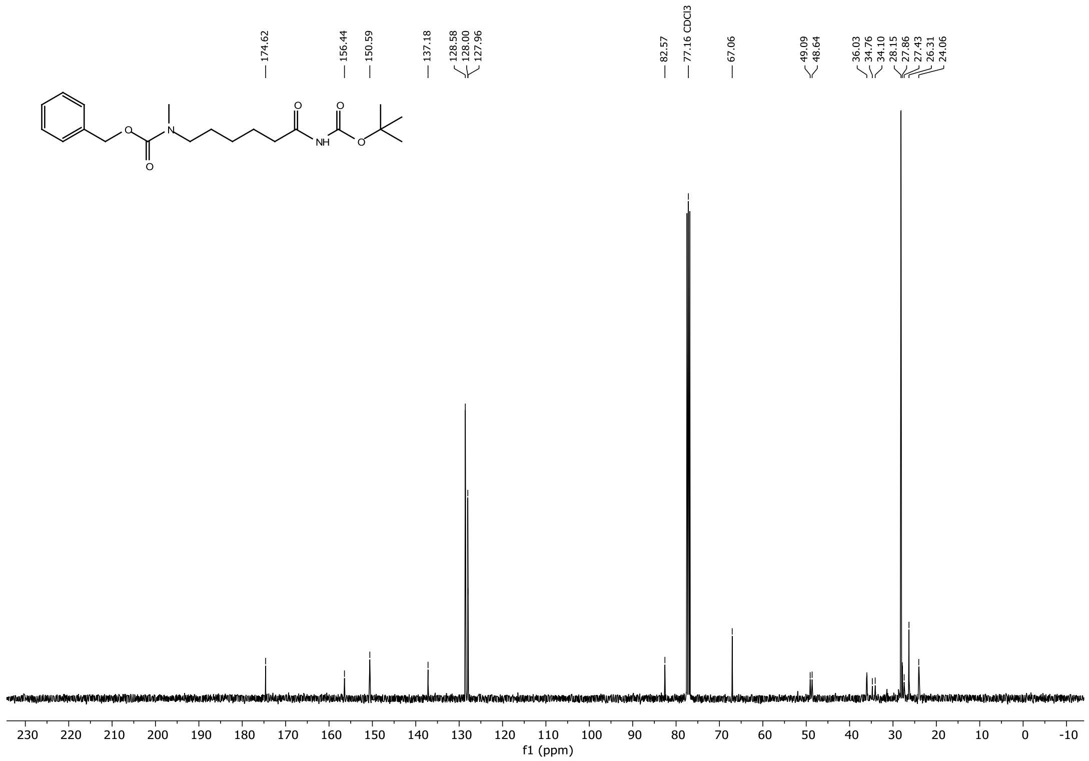


***Supplementary Figure 65. $^{13}C$ NMR spectrum of C12.***

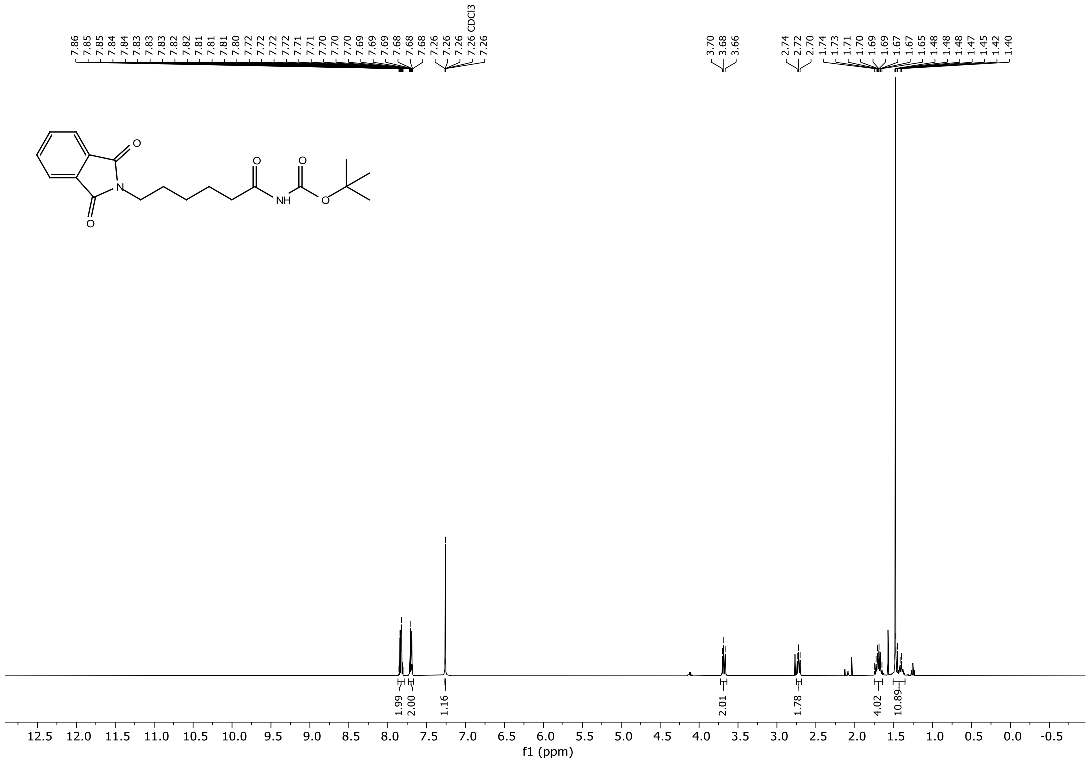

***Supplementary Figure 66. $^{1}H$ NMR spectrum of C13.***

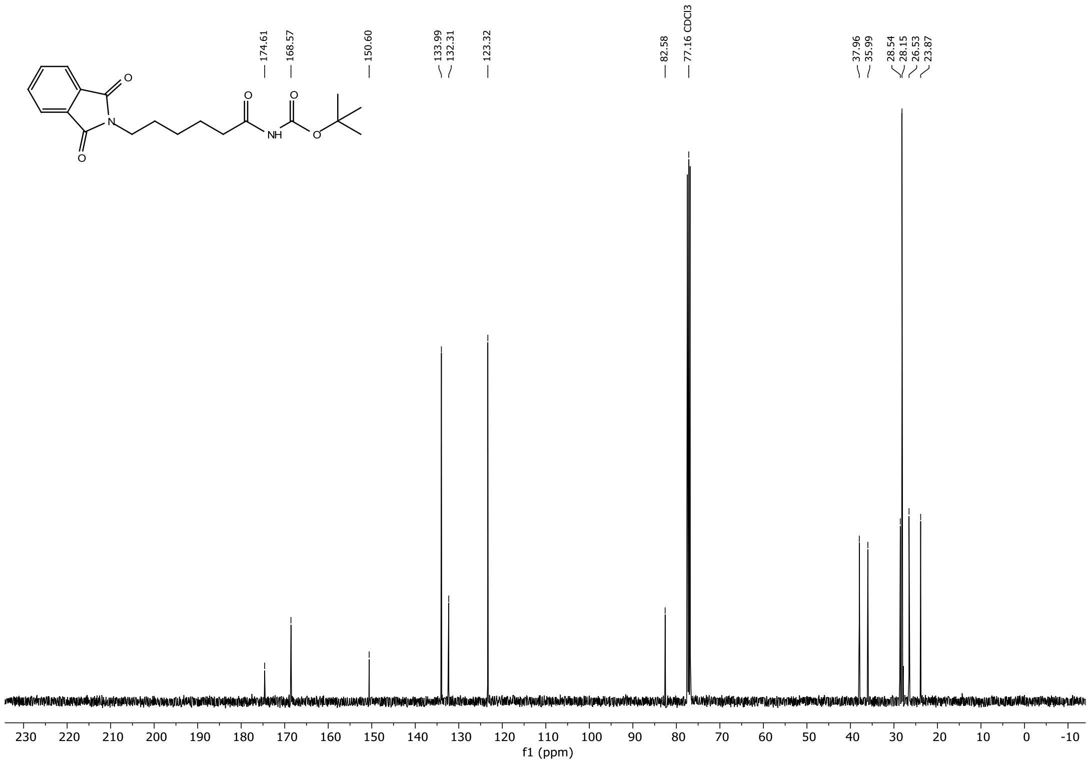

***Supplementary Figure 67. $^{13}C$ NMR spectrum of C13.***

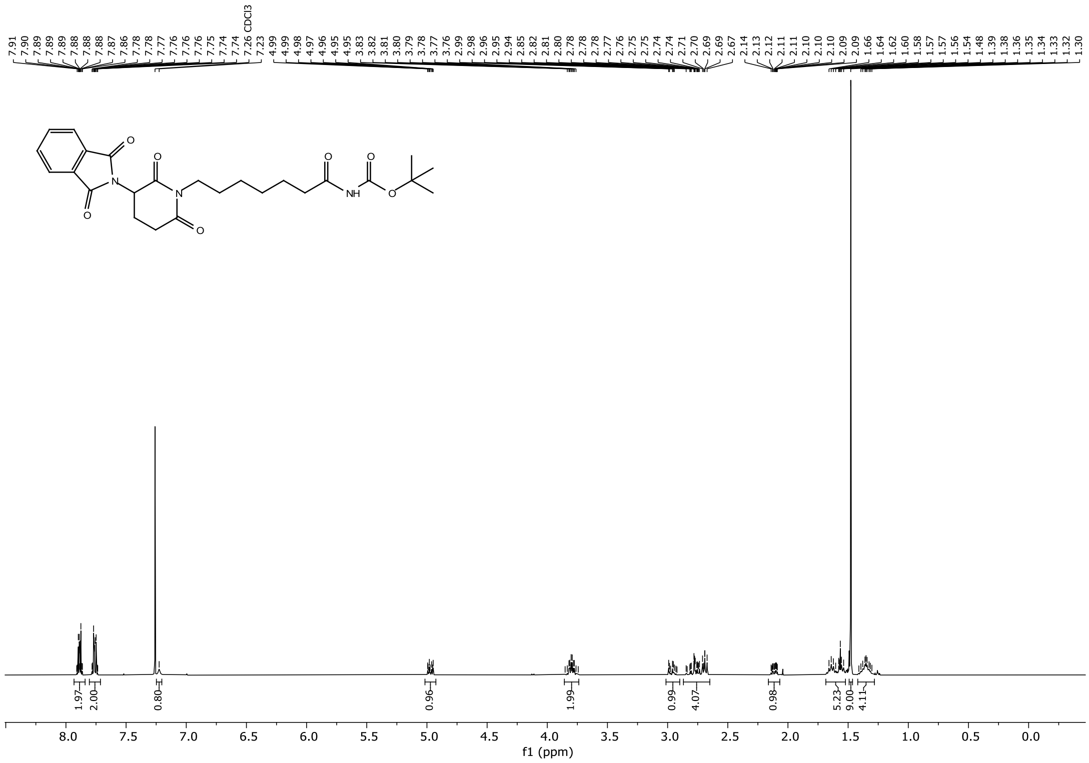


***Supplementary Figure 68. $^{1}$H NMR spectrum of C14.***

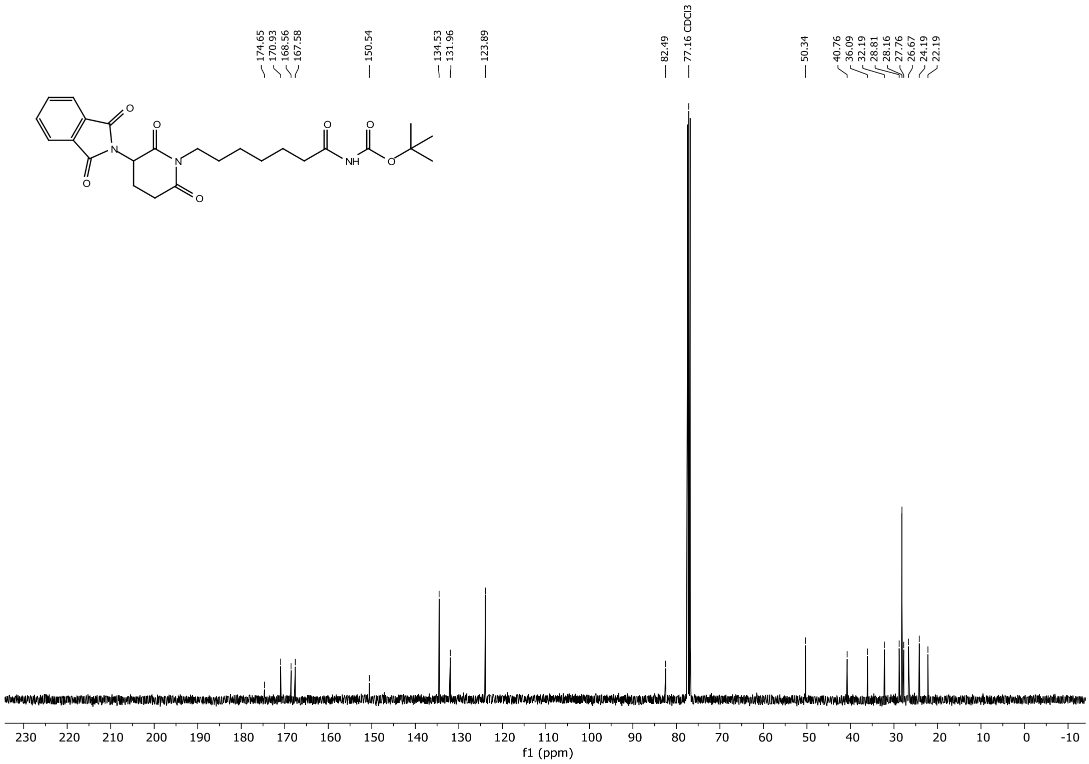


***Supplementary Figure 69. $^{13}$C NMR spectrum of C14.***

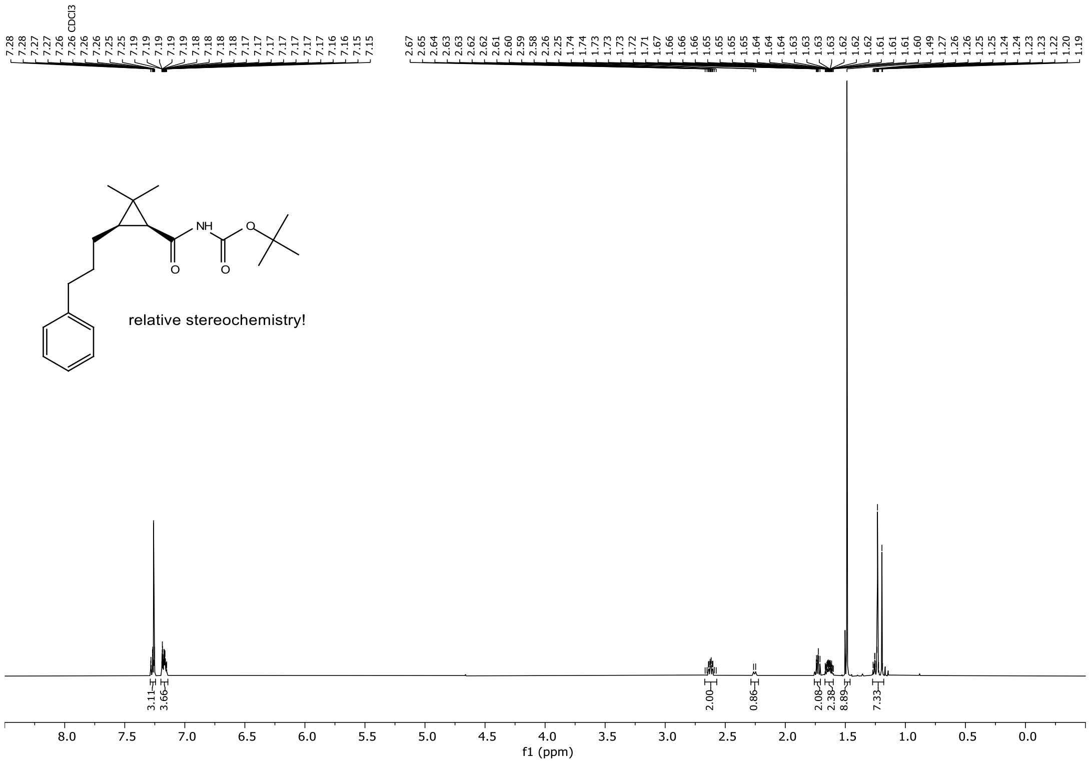


***Supplementary Figure 70.*** ***$^1$H NMR spectrum of C15.***

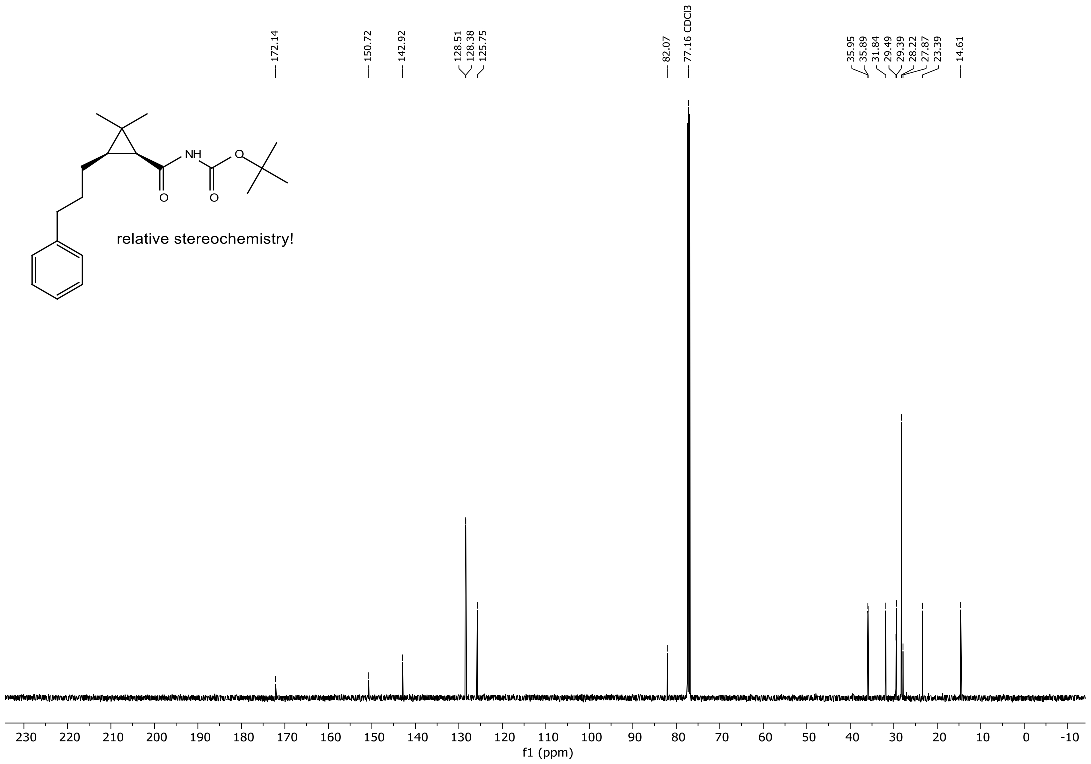


***Supplementary Figure 71.*** ***$^{13}$C NMR spectrum of C15.***

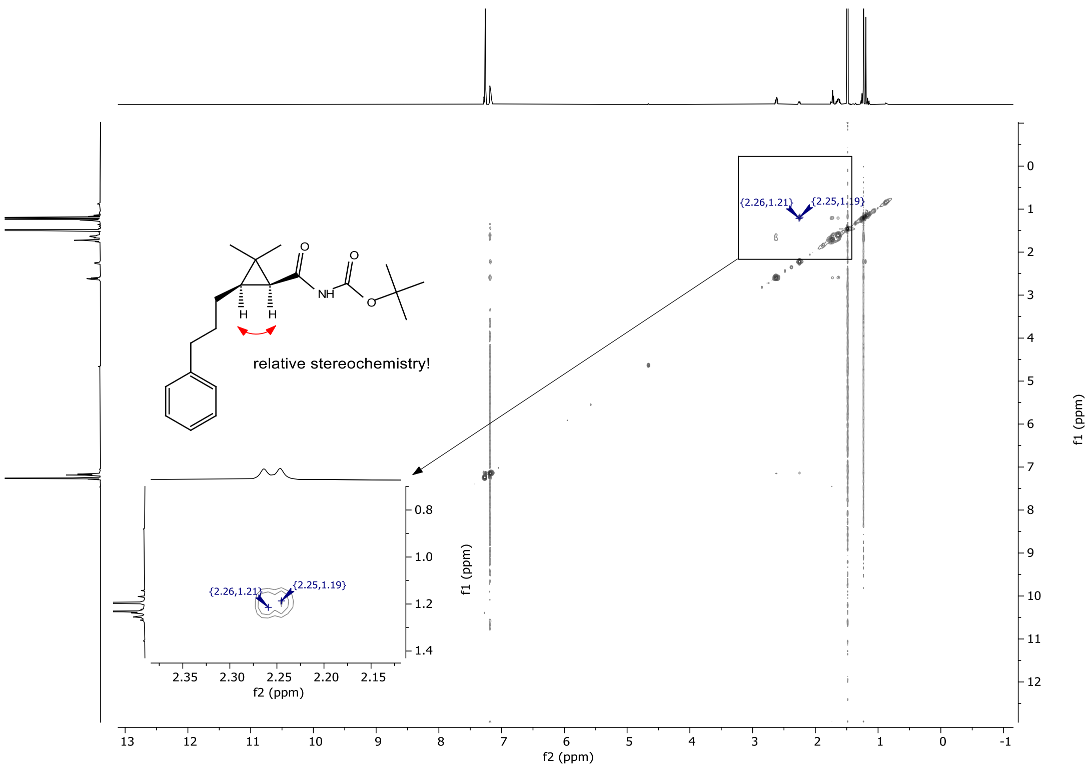


***Supplementary Figure 72. 2D-NOESY NMR spectrum of C15.***

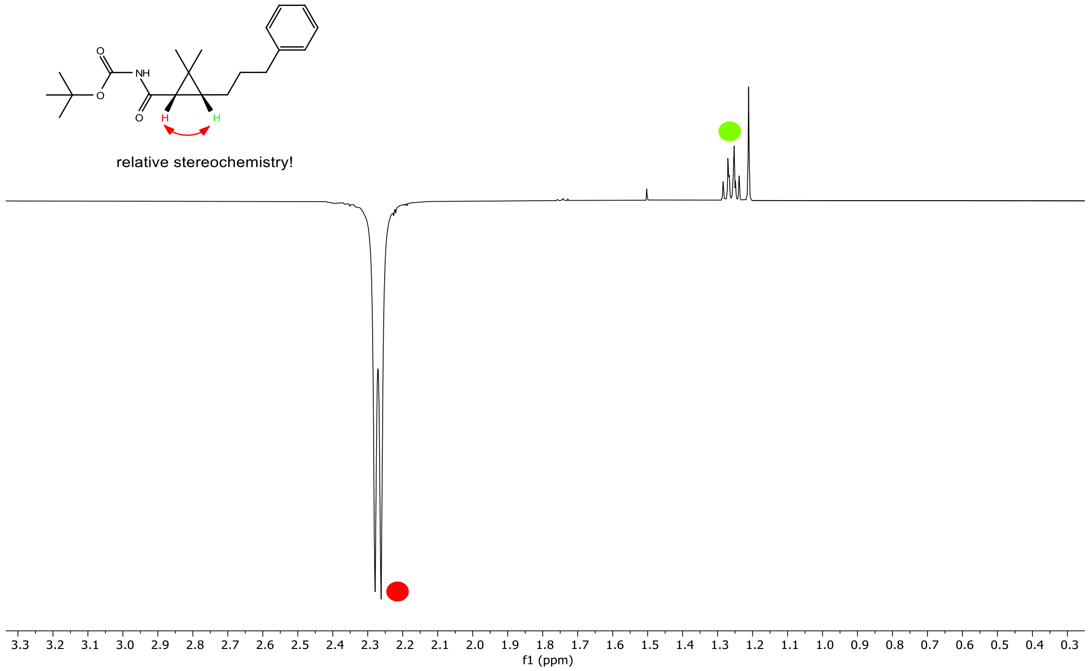


***Supplementary Figure 73. 1D-NOESY NMR spectrum of C15 (doublet at 2.25 ppm is irradiated).***

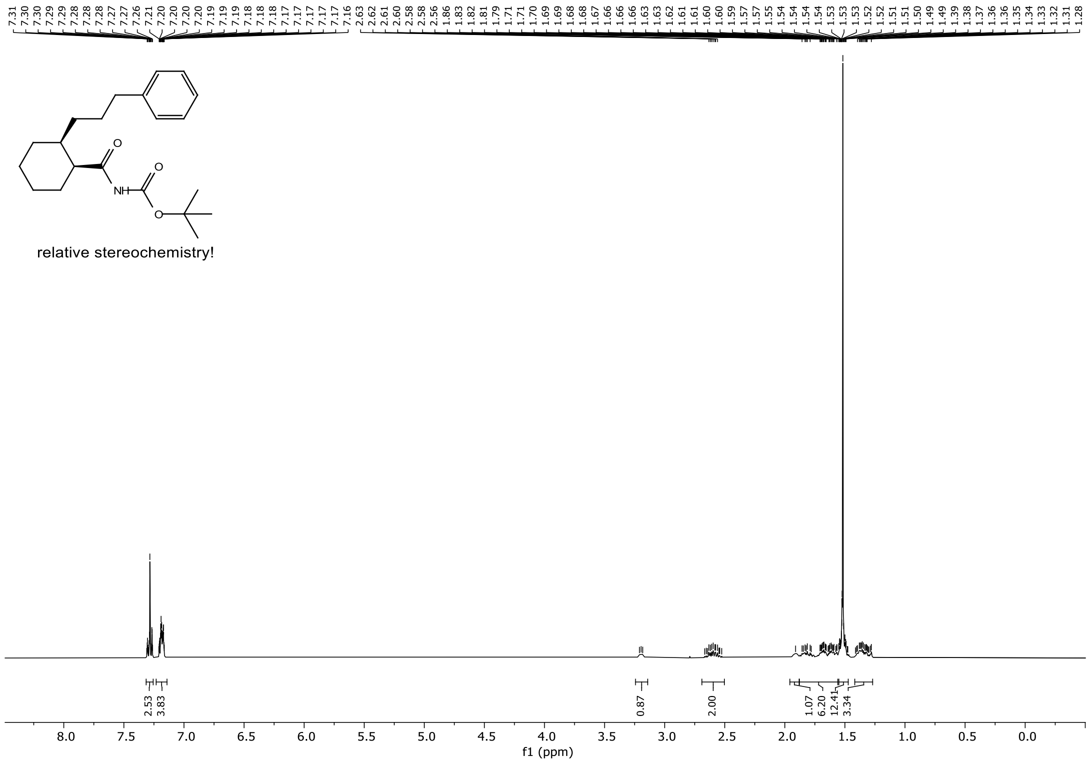

***Supplementary Figure 74.** $^{1}$**H NMR spectrum of C16.***

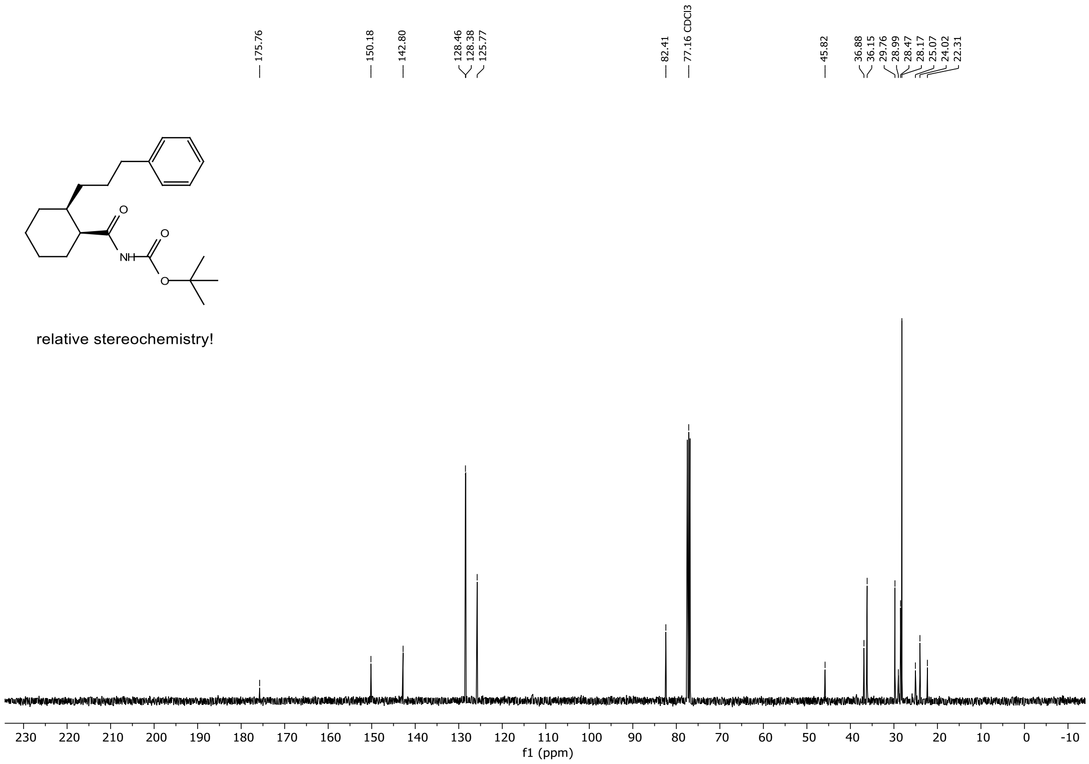

***Supplementary Figure 75.** $^{13}$**C NMR spectrum of C16.***

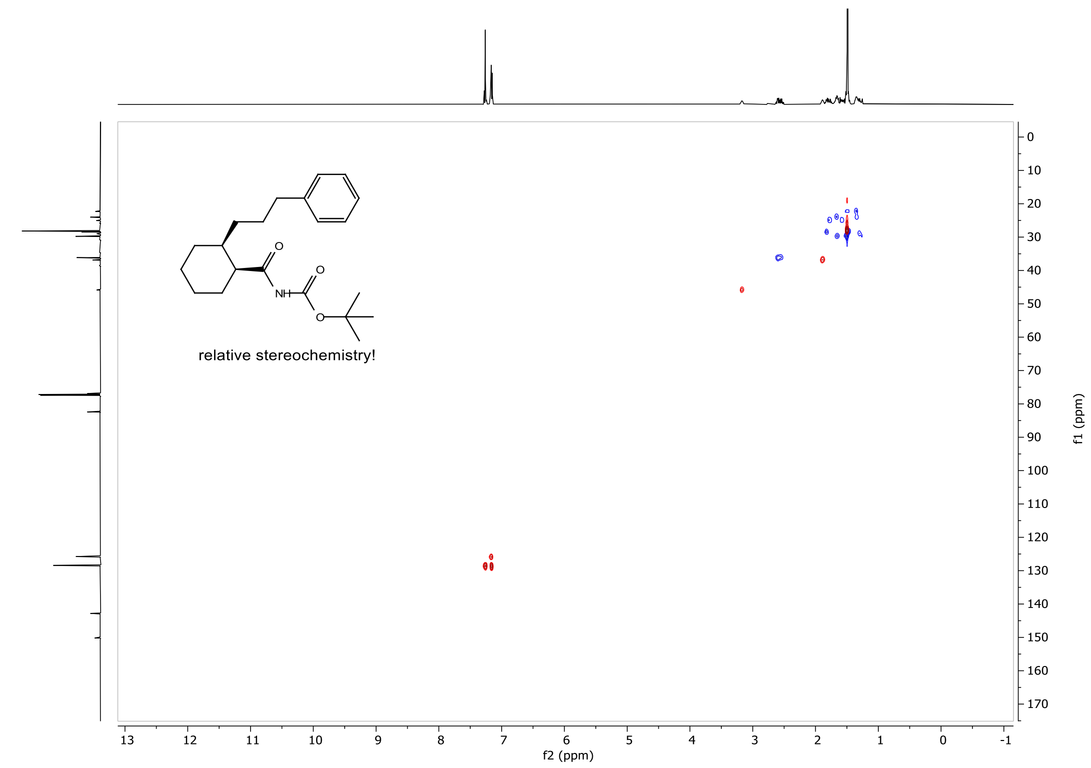


***Supplementary Figure 76. Phase edited HSQC NMR spectrum of C16.***

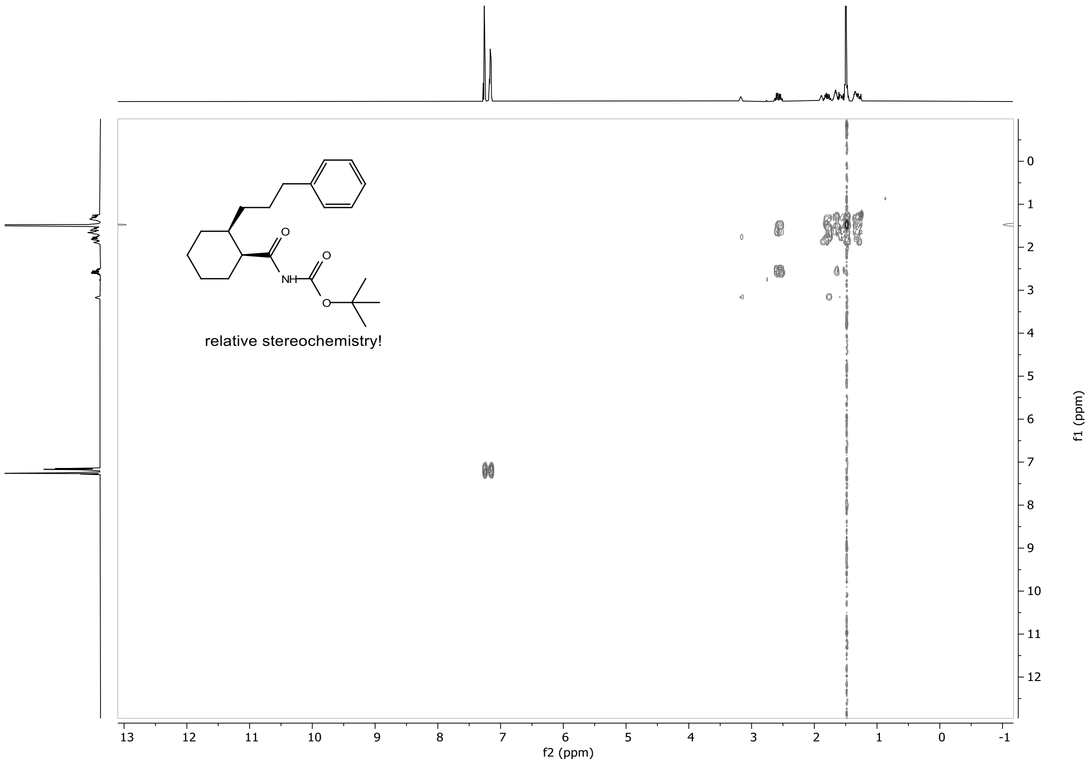


***Supplementary Figure 77. COSY NMR spectrum of C16.***

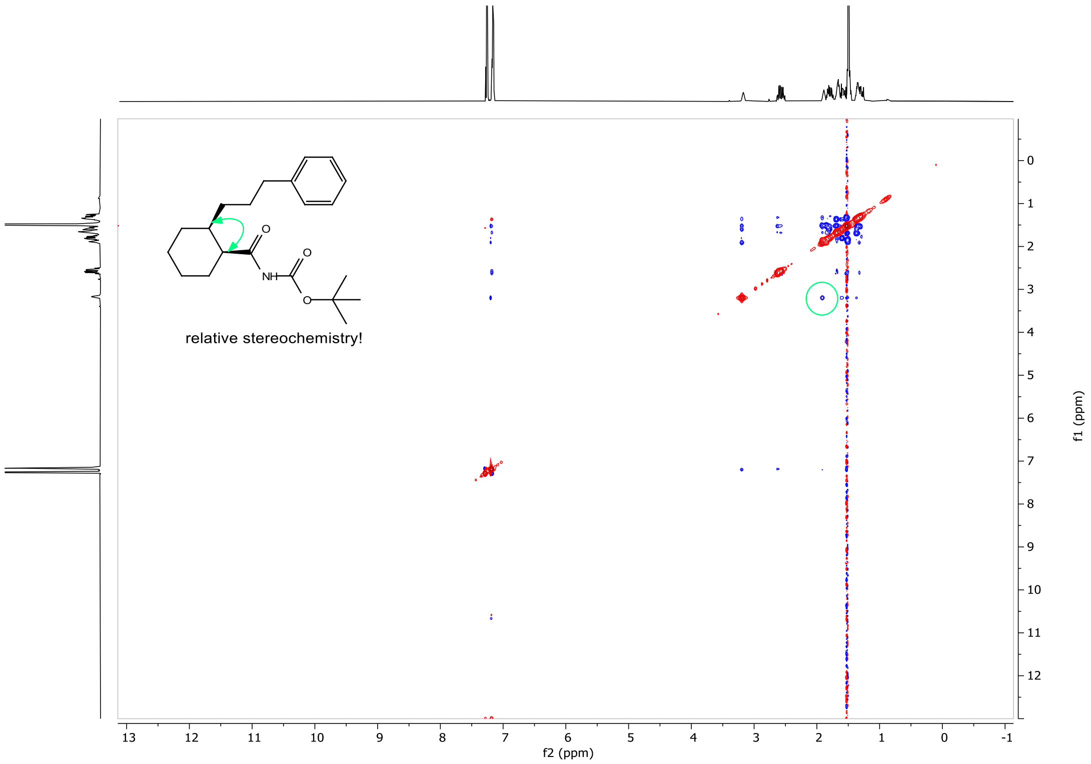


***Supplementary Figure 77. 2D-NOESY NMR spectrum of C16.***

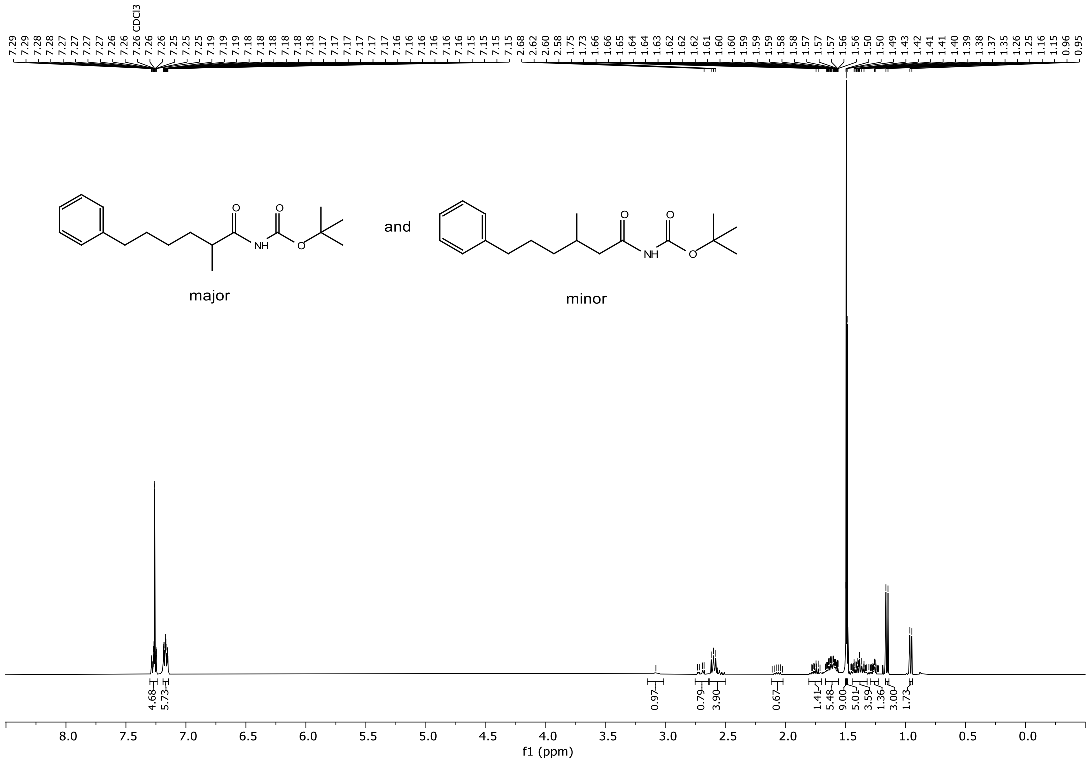


***Supplementary Figure 79. $^{1}$H NMR spectrum of regioisomers mixture of C17.***

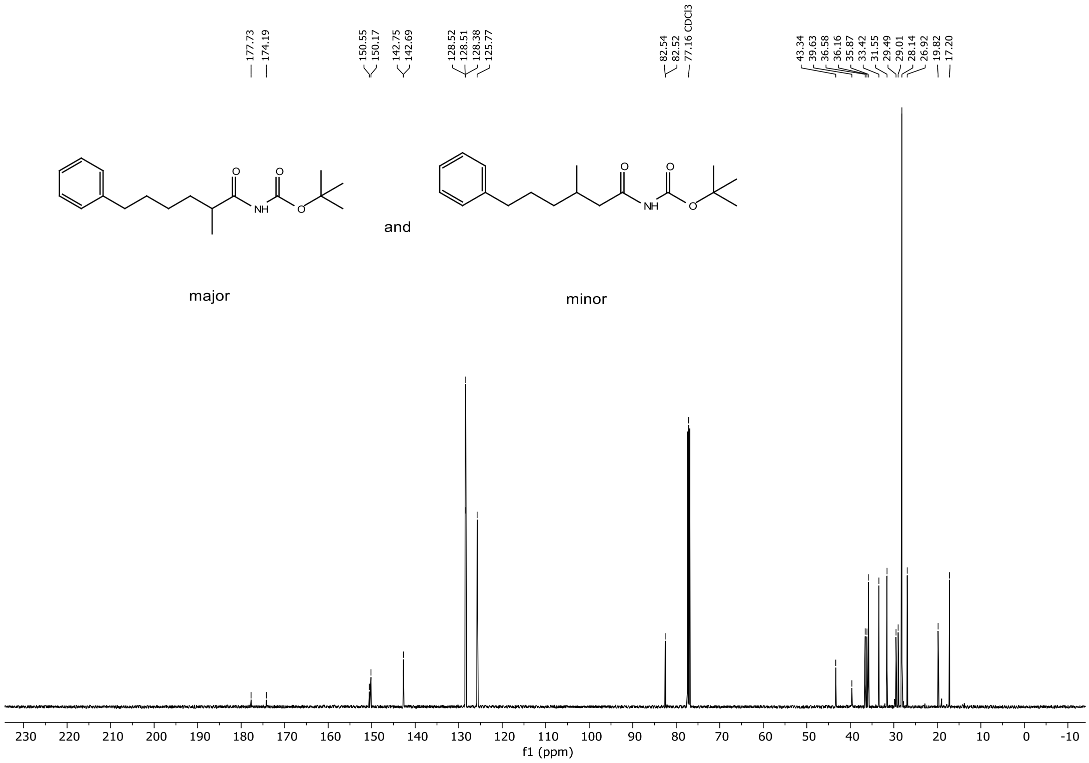


***Supplementary Figure 80. $^{13}$C NMR spectrum of regioisomers mixture of C17.***

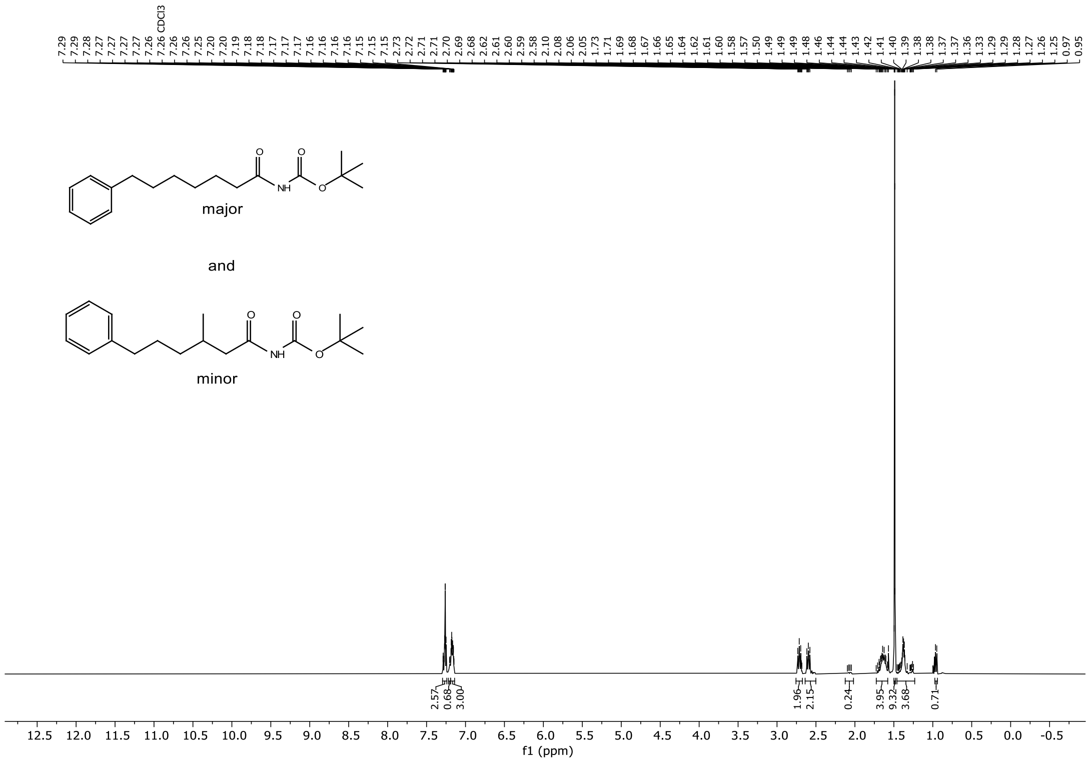


***Supplementary Figure 81. $^{1}$H NMR spectrum of linear and branched amide mixture of E1.***

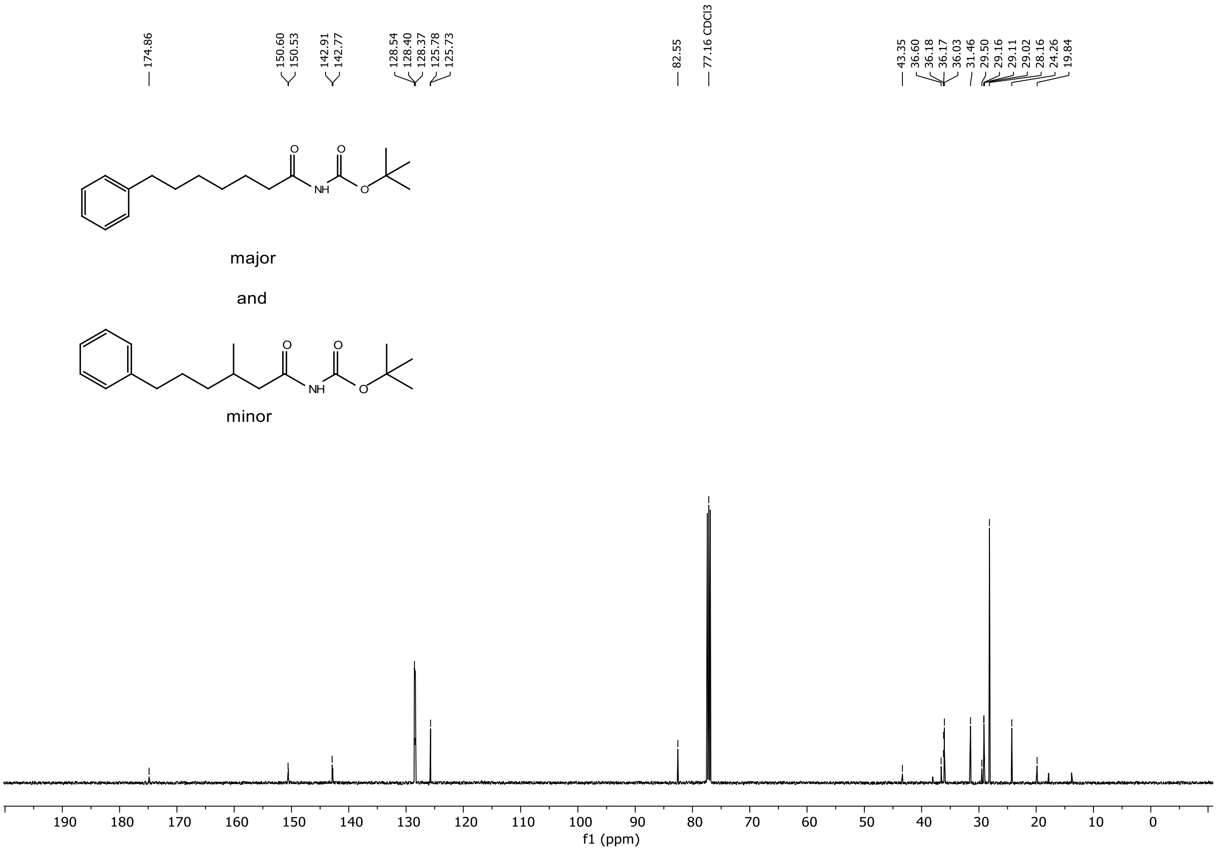


***Supplementary Figure 82. $^{13}$C NMR spectrum of linear and branched amide mixture of E1.***

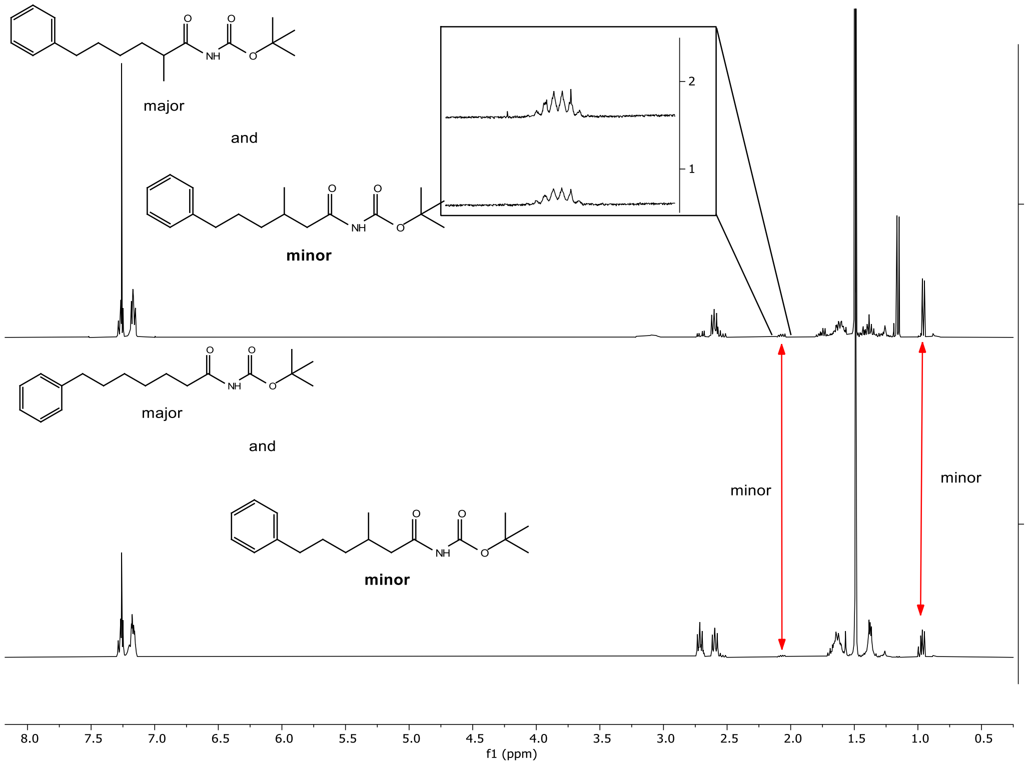


***Supplementary Figure 83. Comparison between $^{1}$H NMR spectra of E1 and C17.***

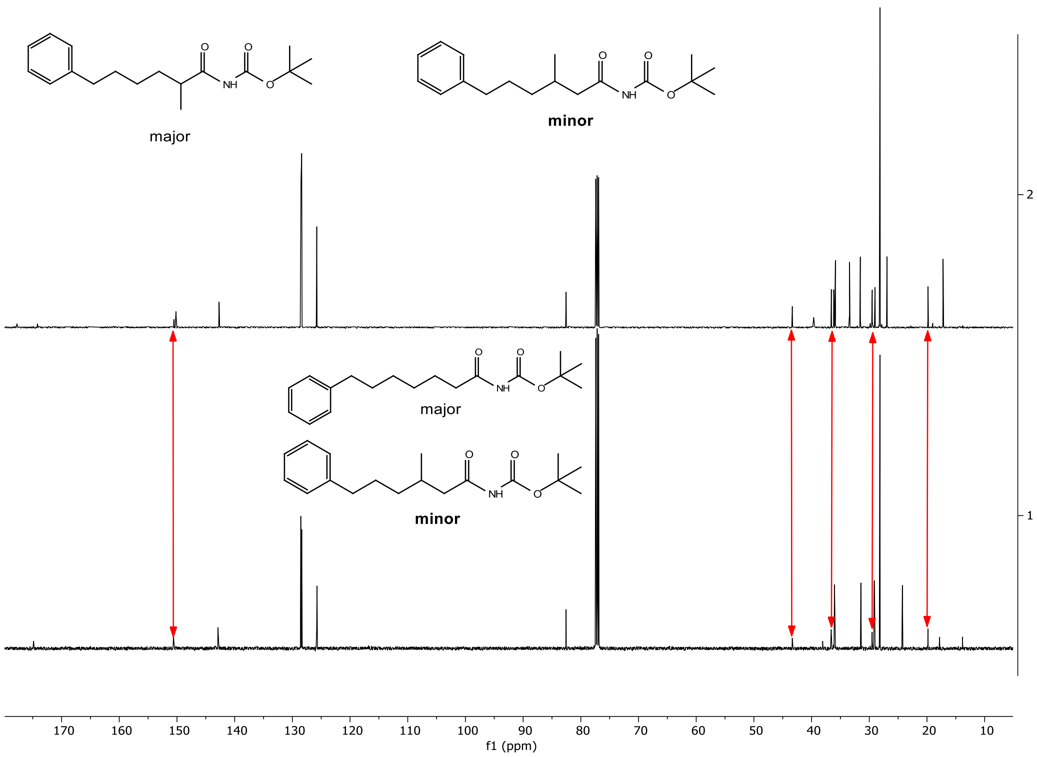


***Supplementary Figure 84. Comparison between $^{13}$C NMR spectra of E1 and C17.***

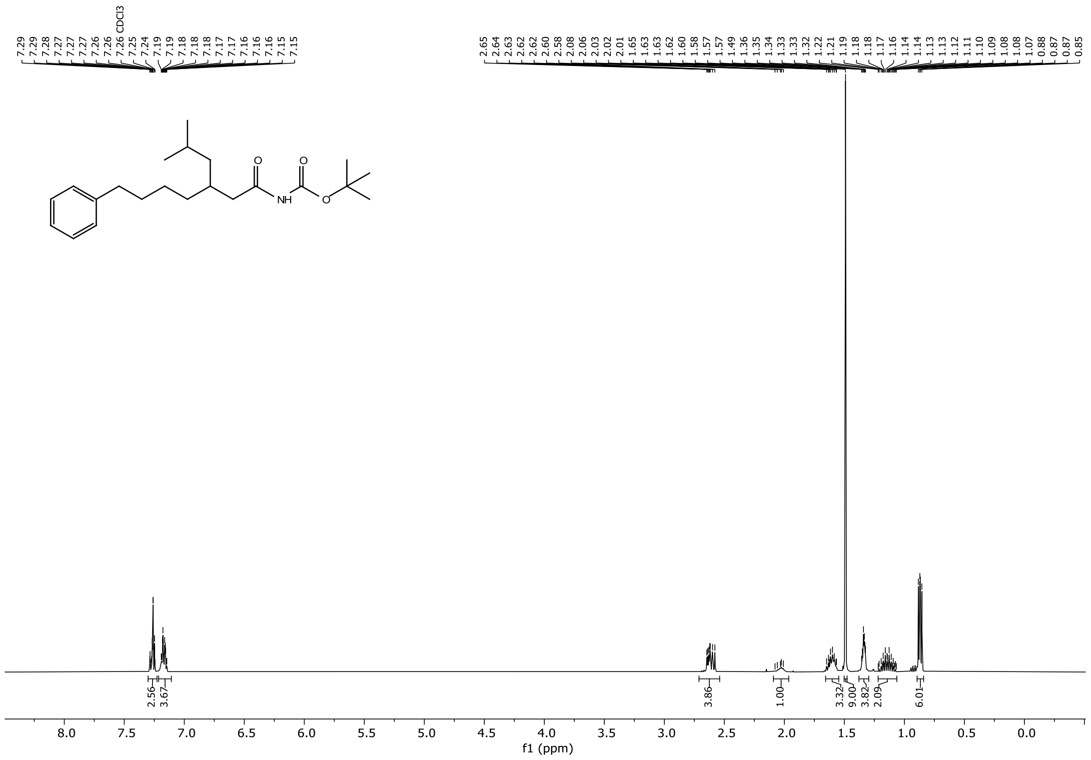


***Supplementary Figure 85. $^{1}$H NMR spectrum of E2.***

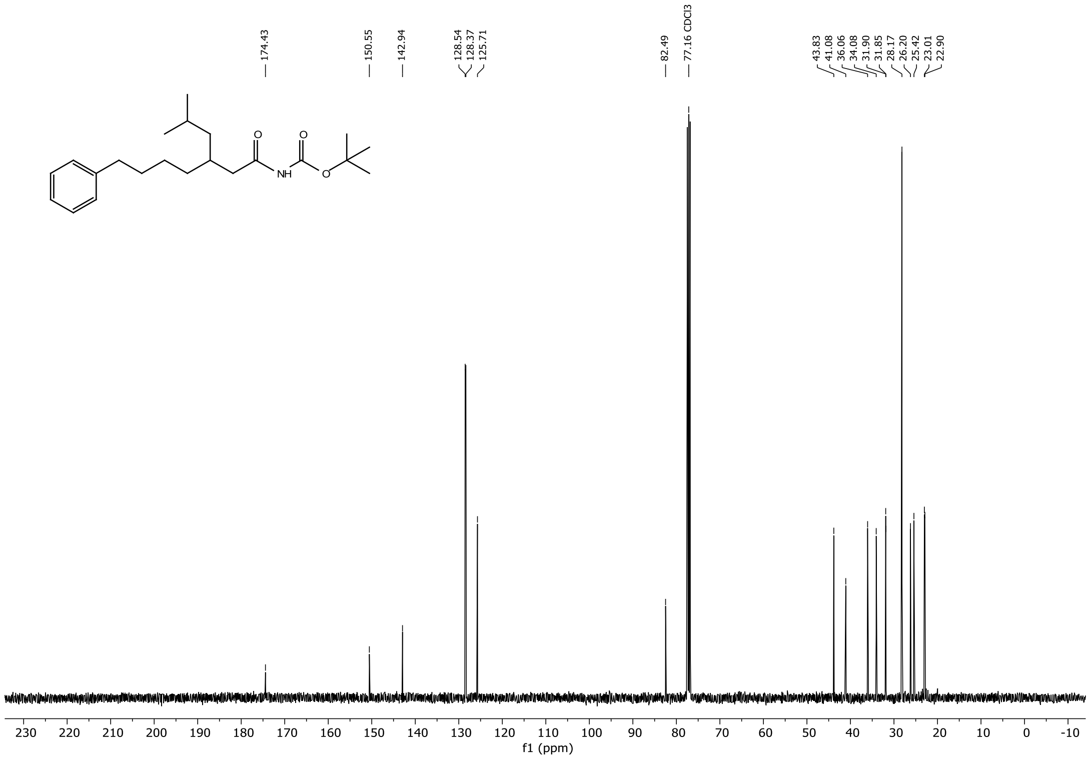


***Supplementary Figure 86. $^{13}$C NMR spectrum of E2.***

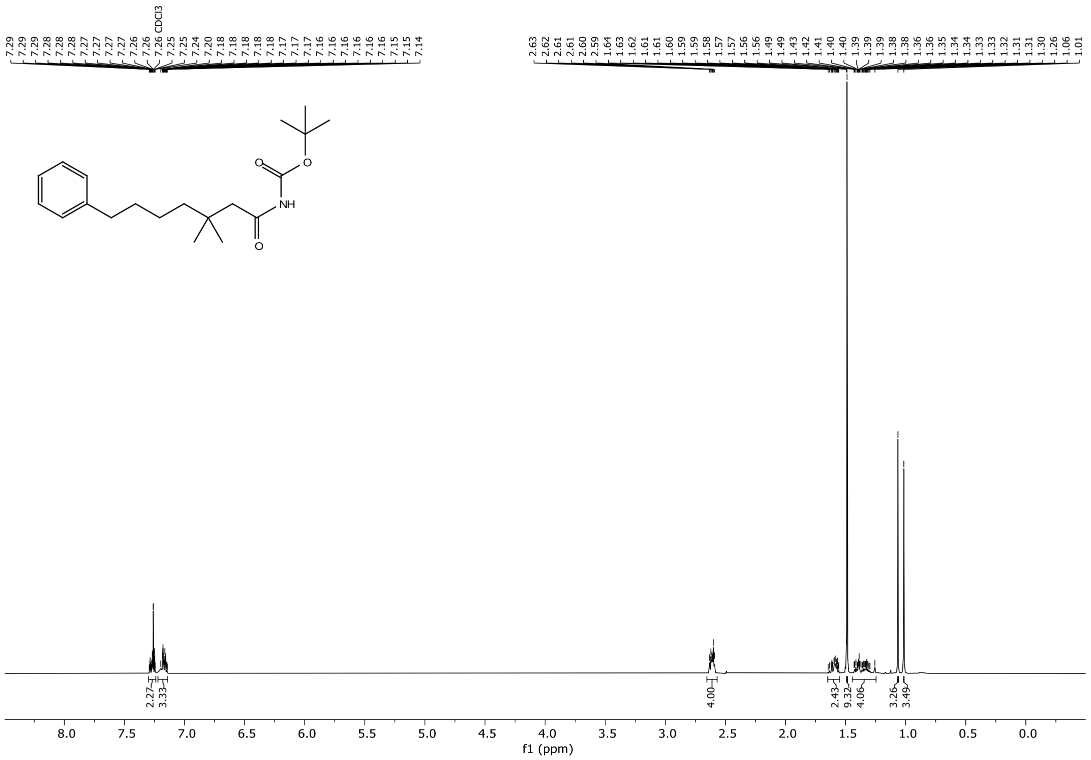


***Supplementary Figure 87. $^{1}$H NMR spectrum of E3.***

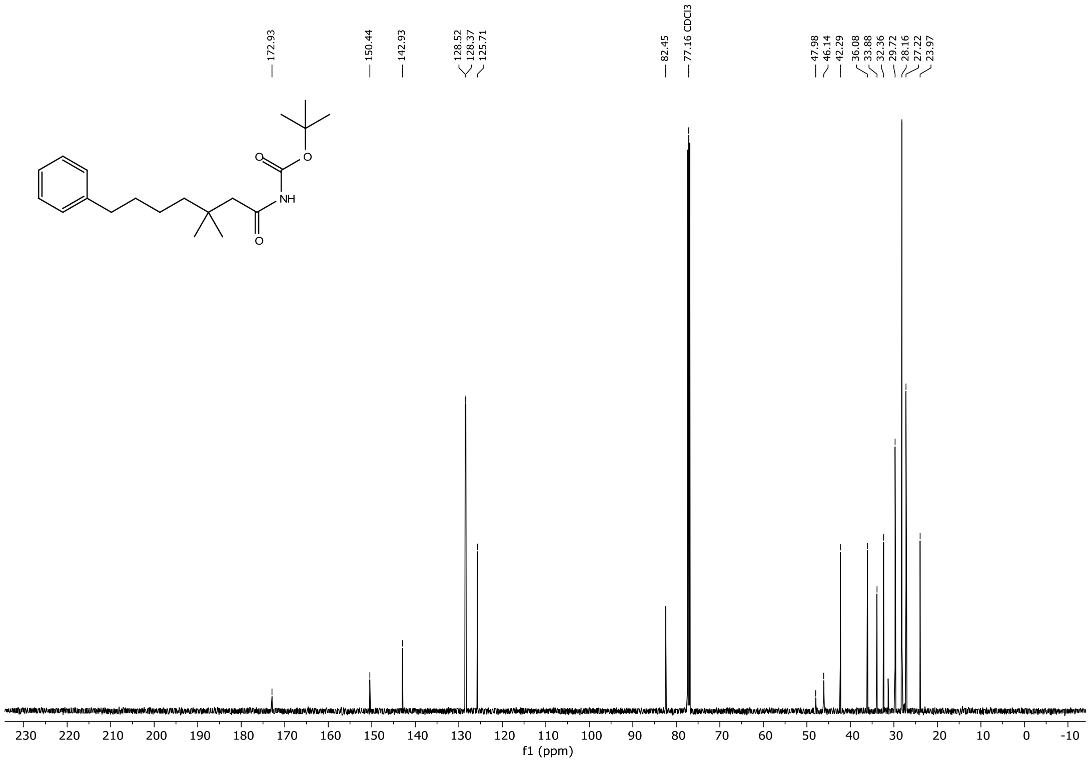


***Supplementary Figure 88. $^{13}$C NMR spectrum of E3.***

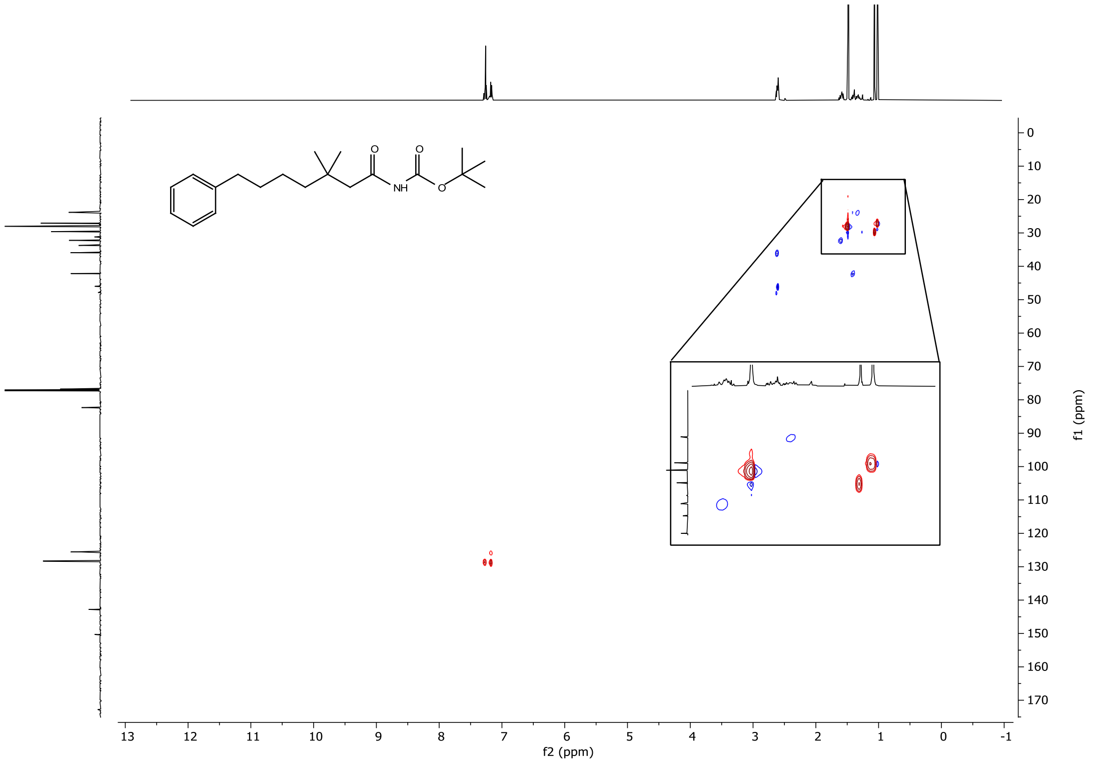


***Supplementary Figure 89. Phase edited HSQC NMR spectrum of E3.***

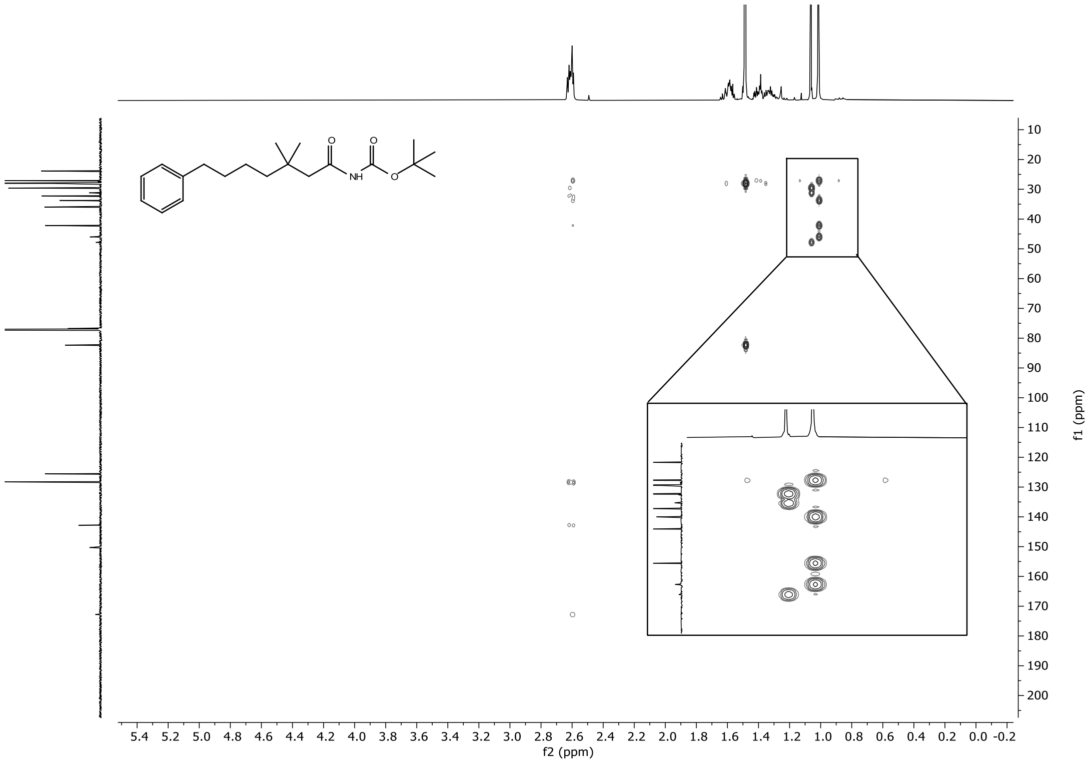

**_Supplementary Figure 90. HMBC NMR spectrum of E3._**

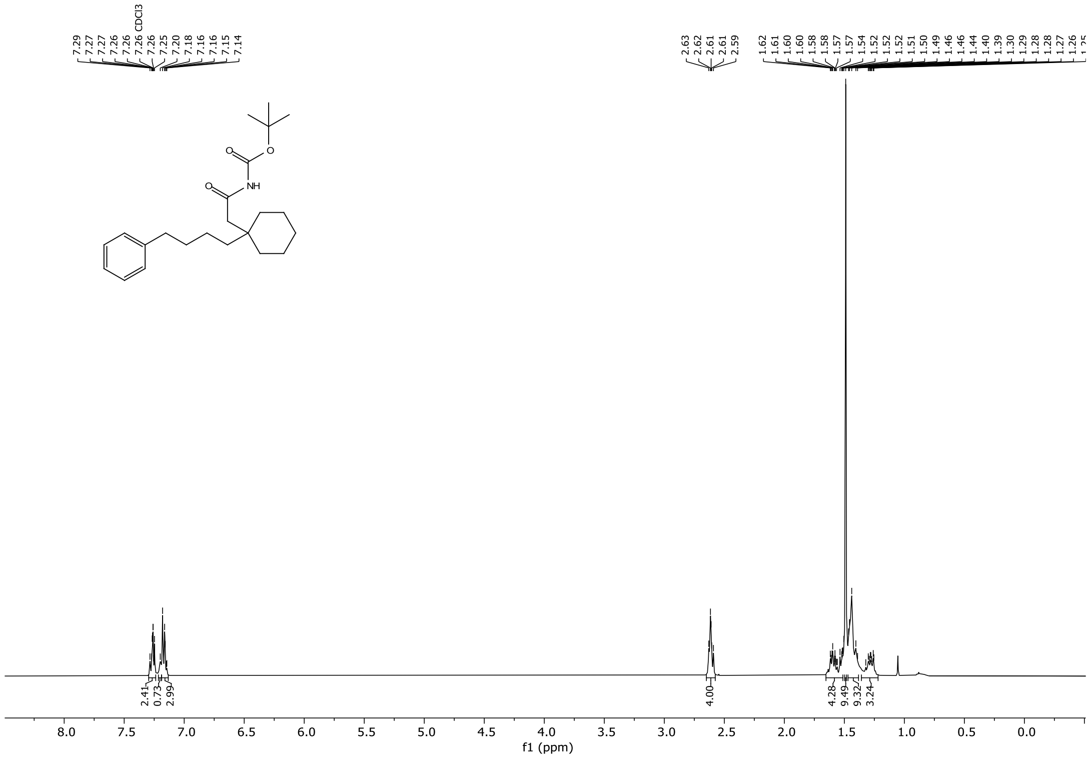

**_Supplementary Figure 91. ¹H NMR spectrum of E4._**

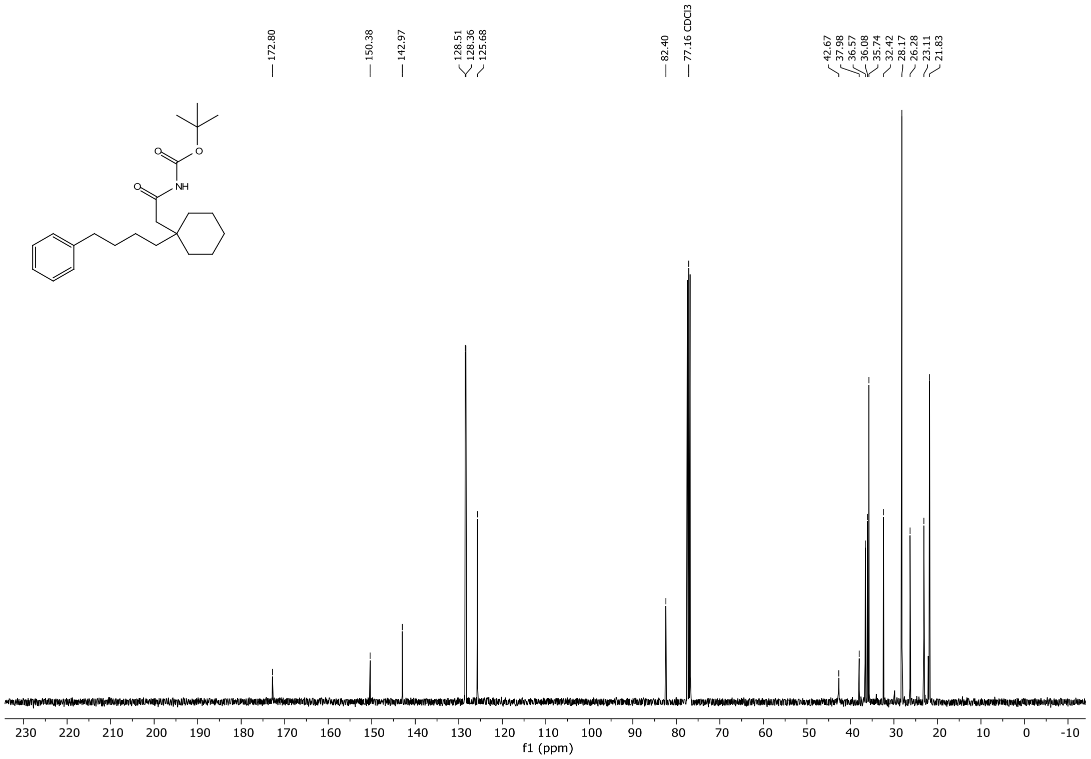


***Supplementary Figure 92. $^{13}$C NMR spectrum of E4.***

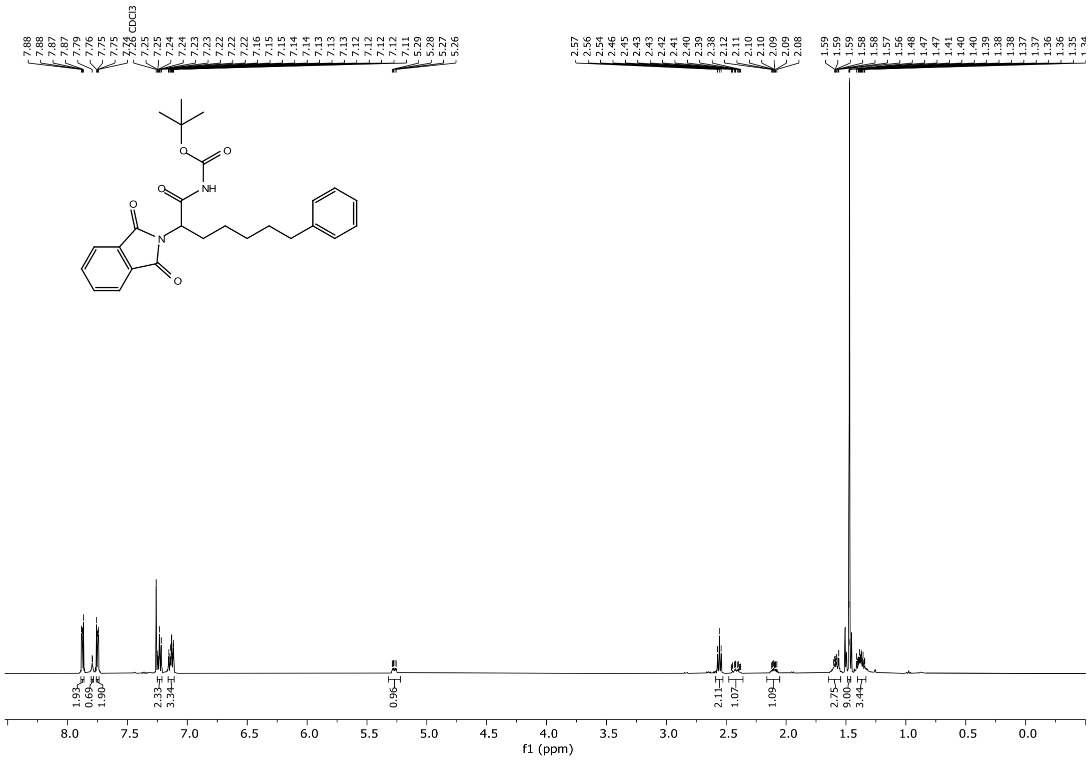


***Supplementary Figure 93. $^{1}$H NMR spectrum of E5.***

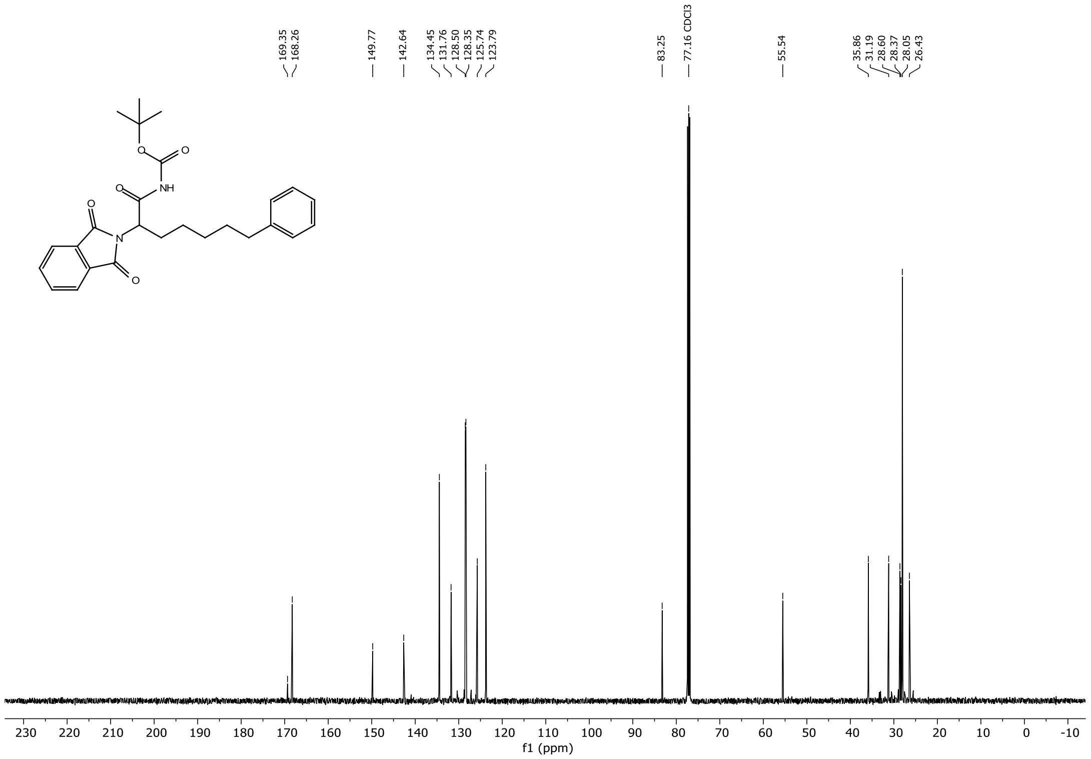


***Supplementary Figure 94. $^{13}$C NMR spectrum of E5.***

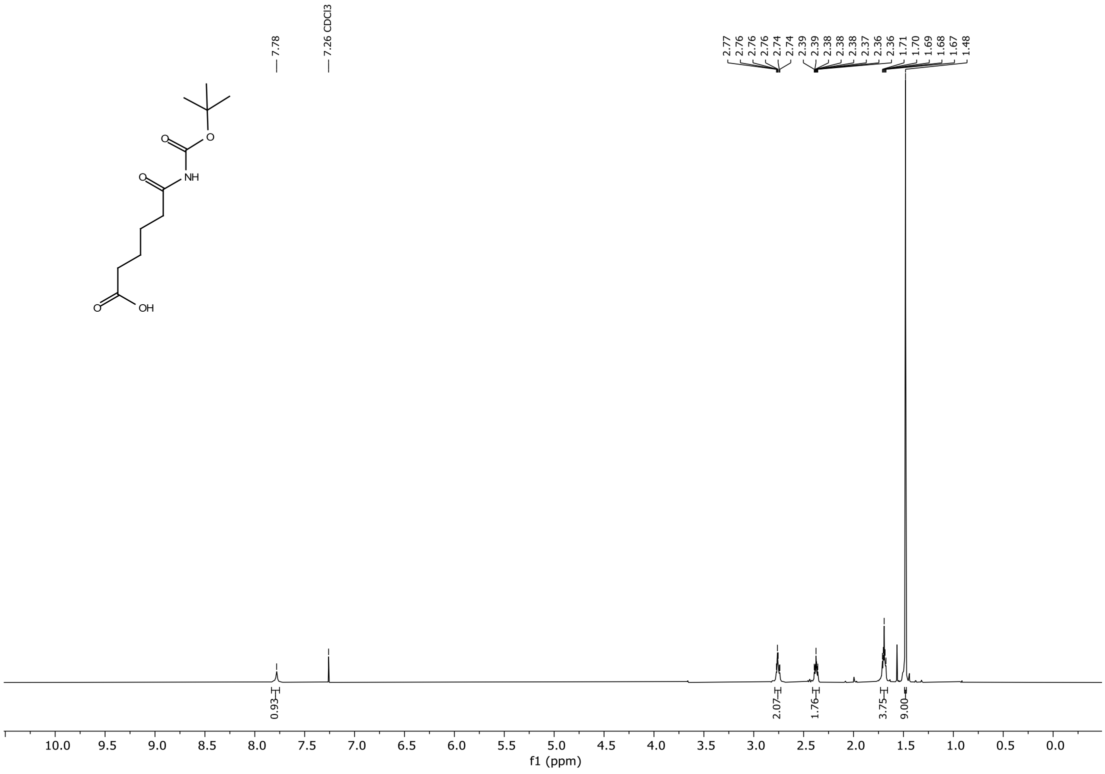


***Supplementary Figure 95. $^{1}$H NMR spectrum of AS1.***

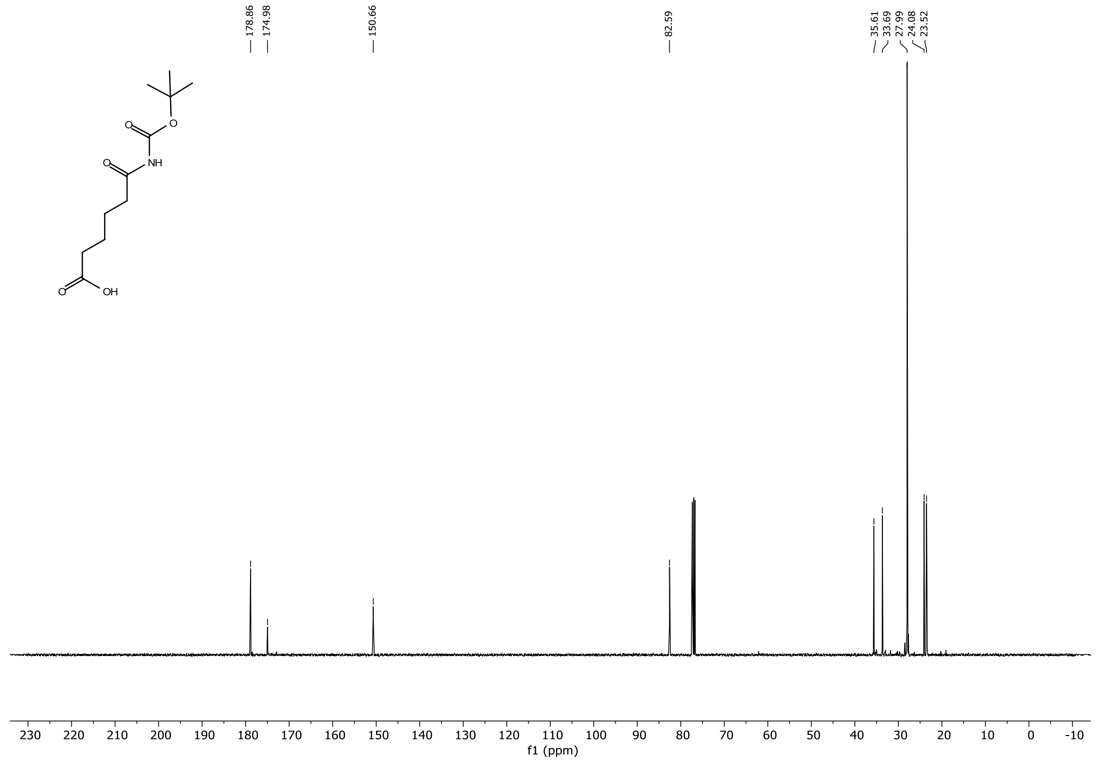


***Supplementary Figure 96.*** ***$^{13}$C NMR spectrum of AS1.***

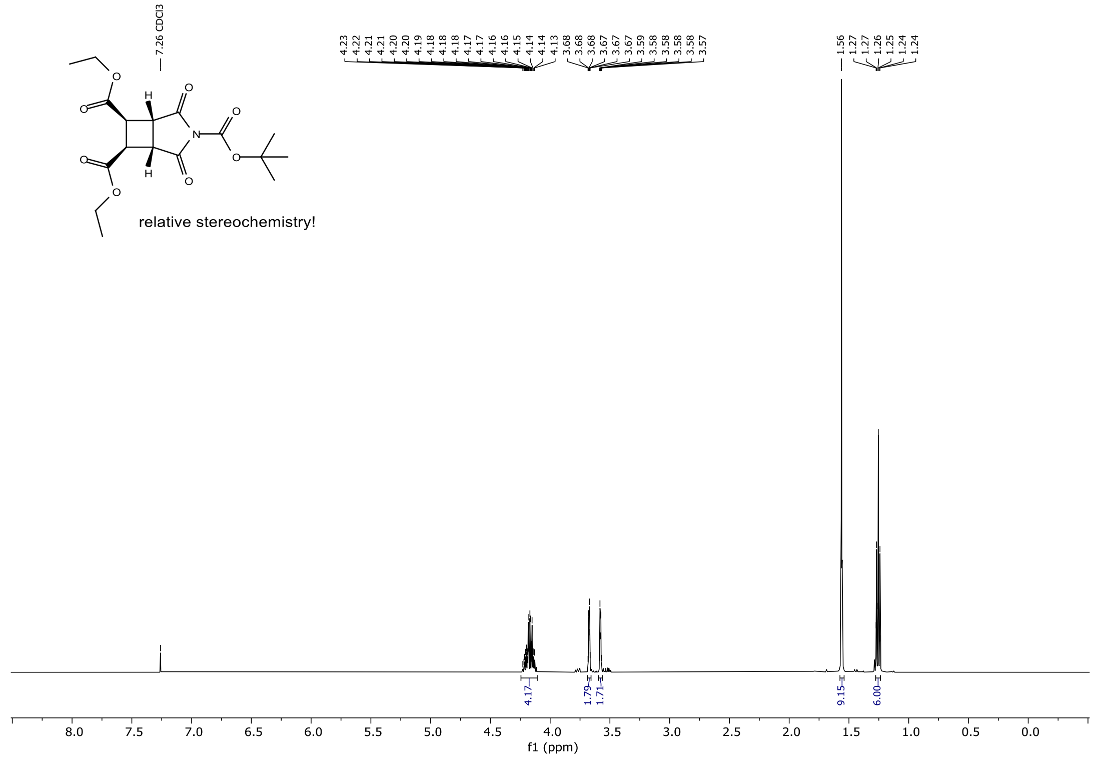


***Supplementary Figure 97. $^{1}$H NMR spectrum of 3-(tert-butyl) 6,7-diethyl 2,4-dioxo-3-azabicyclo[3.2.0]heptane-3,6,7-tricarboxylate (F2, minor cis-ester diastereomer).***

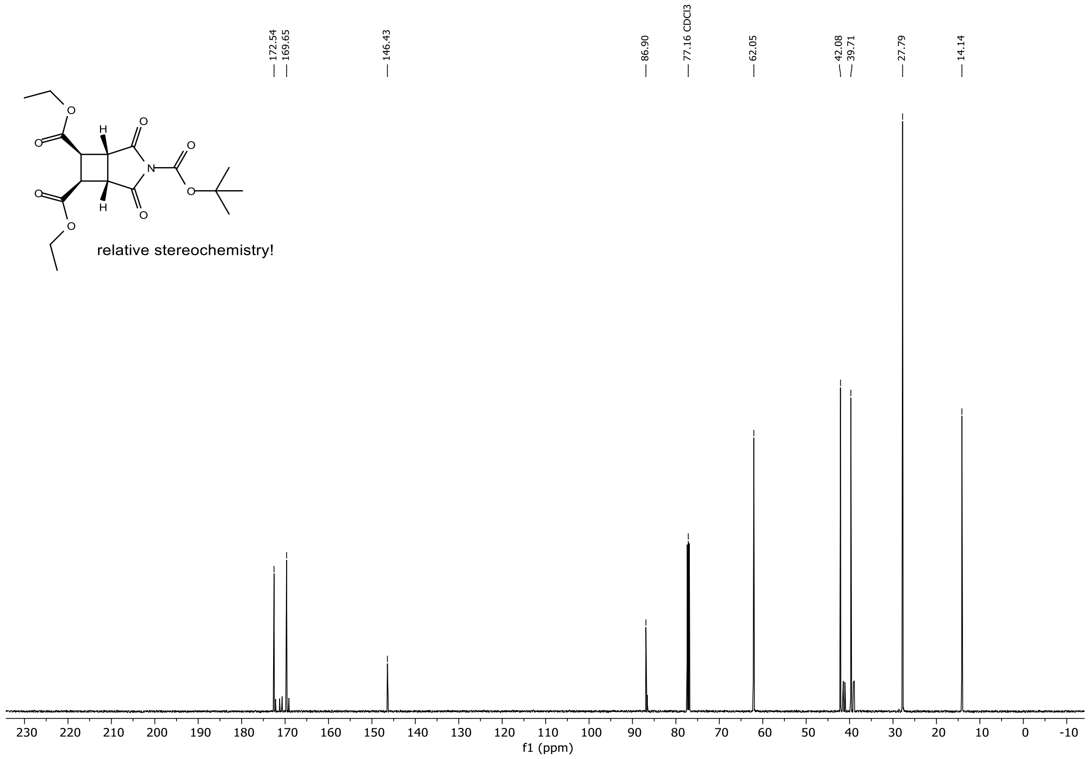


***Supplementary Figure 98. $^{13}$C NMR spectrum of 3-(tert-butyl) 6,7-diethyl 2,4-dioxo-3-azabicyclo[3.2.0]heptane-3,6,7-tricarboxylate (F2, minor cis-ester diastereomer).***

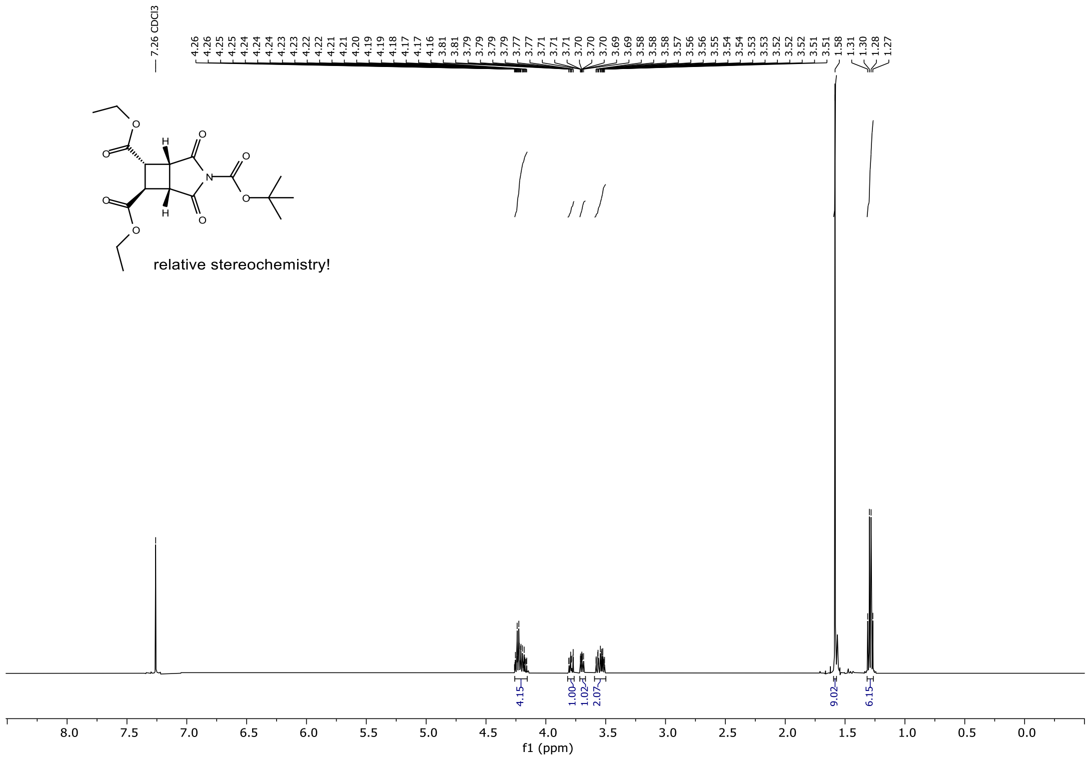


***Supplementary Figure 99. $^{1}$H NMR spectrum of 3-(tert-butyl) 6,7-diethyl 2,4-dioxo-3-azabicyclo[3.2.0]heptane-3,6,7-tricarboxylate (F2, major trans-ester diastereomer).***

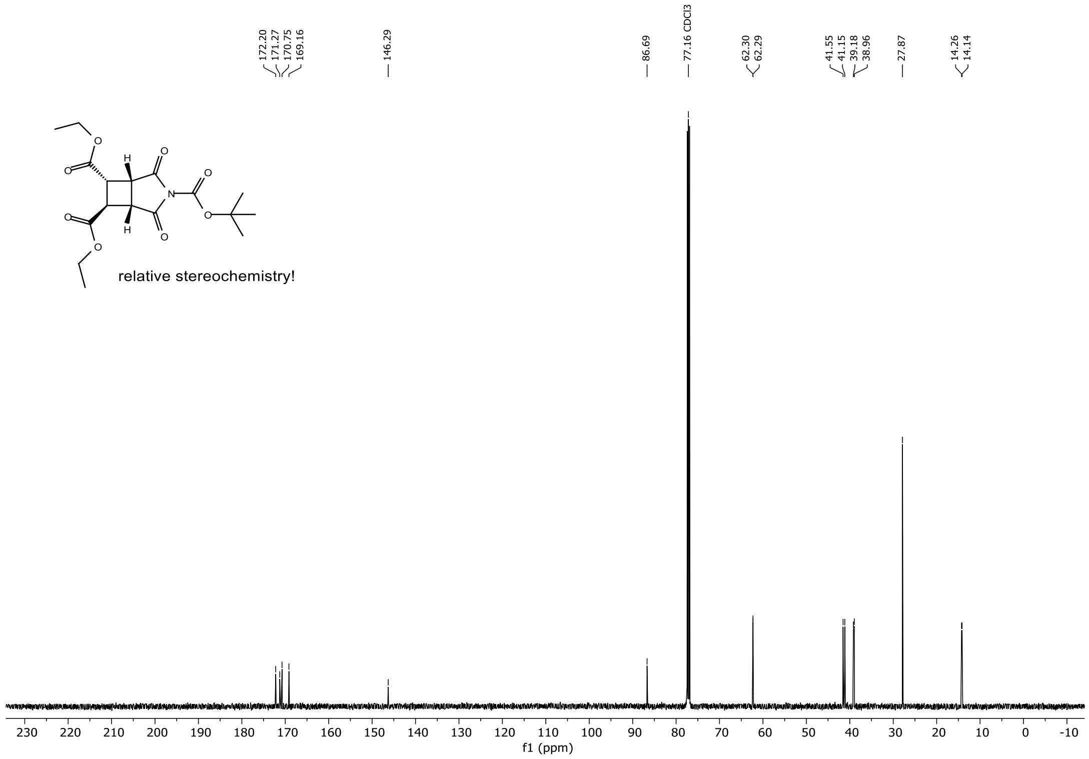


***Supplementary Figure 100. $^{13}C$ NMR spectrum of 3-(tert-butyl) 6,7-diethyl 2,4-dioxo-3-azabicyclo[3.2.0]heptane-3,6,7-tricarboxylate (F2, major trans-ester diastereomer).***

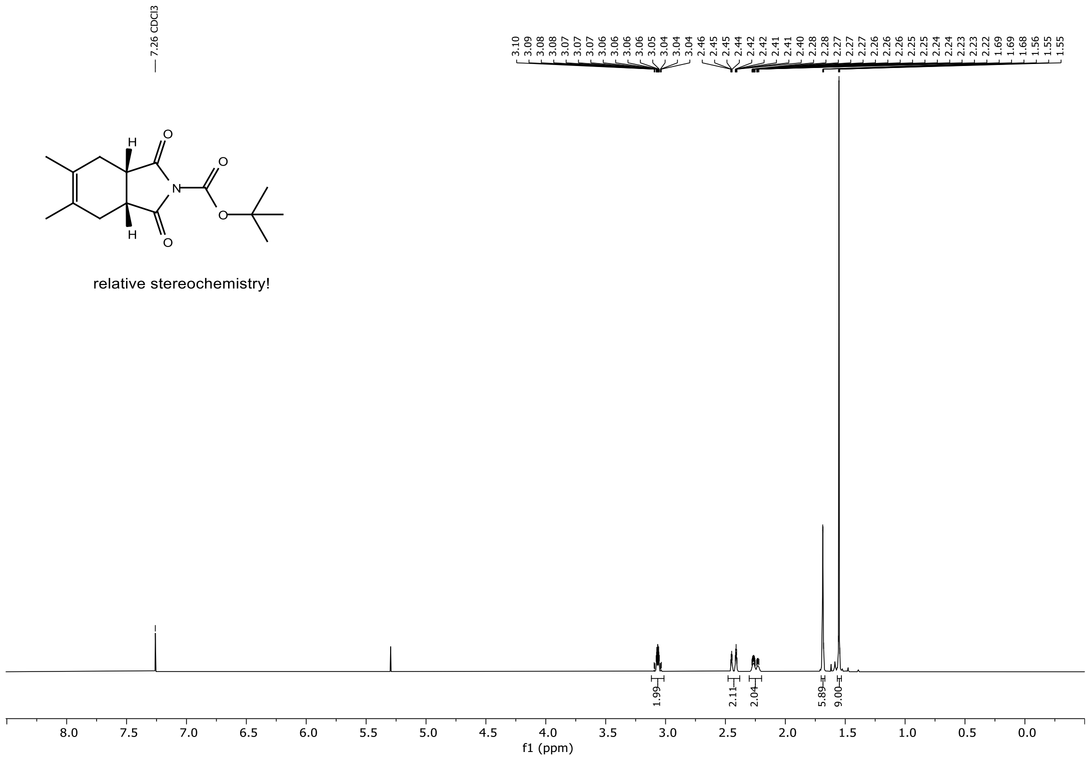


***Supplementary Figure 101. $^{1}H$ NMR spectrum of tert-butyl 5,6-dimethyl-1,3-dioxo-1,3,3a,4,7,7a-hexahydro-2H-isoindole-2-carboxylate (F3).***

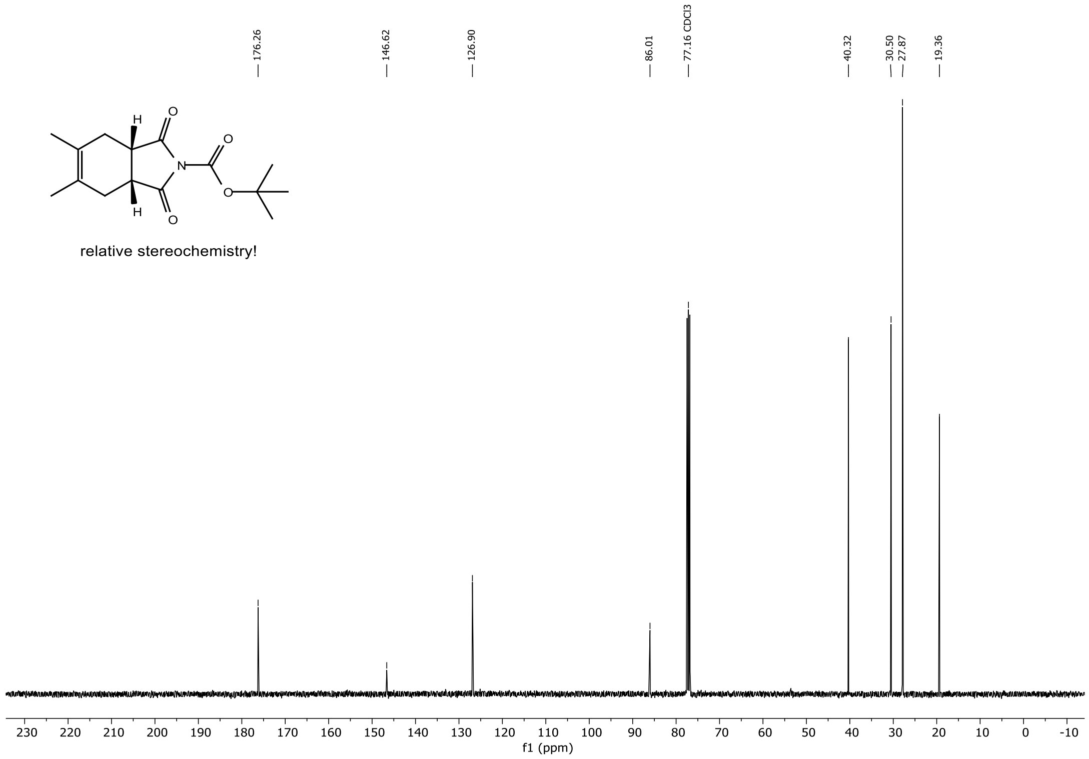


***Supplementary Figure 102. $^{13}$C NMR spectrum of tert-butyl 5,6-dimethyl-1,3-dioxo-1,3,3a,4,7,7a-hexahydro-2H-isoindole-2-carboxylate (F3).***

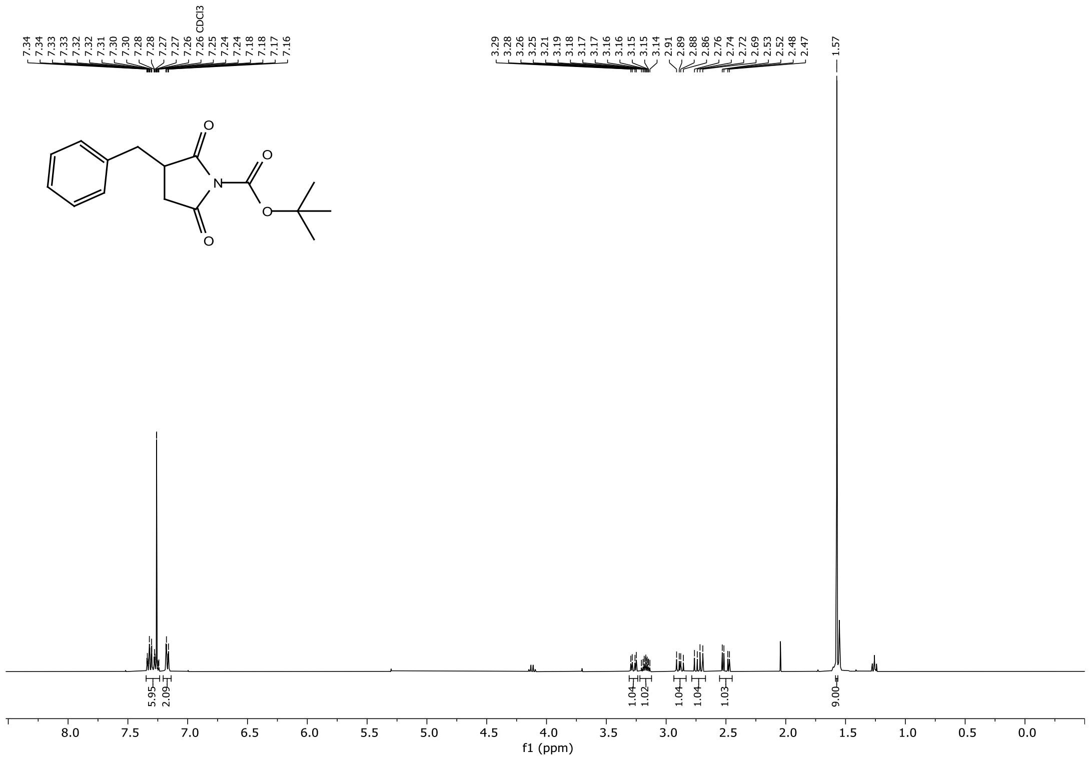


***Supplementary Figure 103. $^{1}$H NMR spectrum of tert-butyl 3-benzyl-2,5-dioxopyrrolidine-1-carboxylate (F4).***

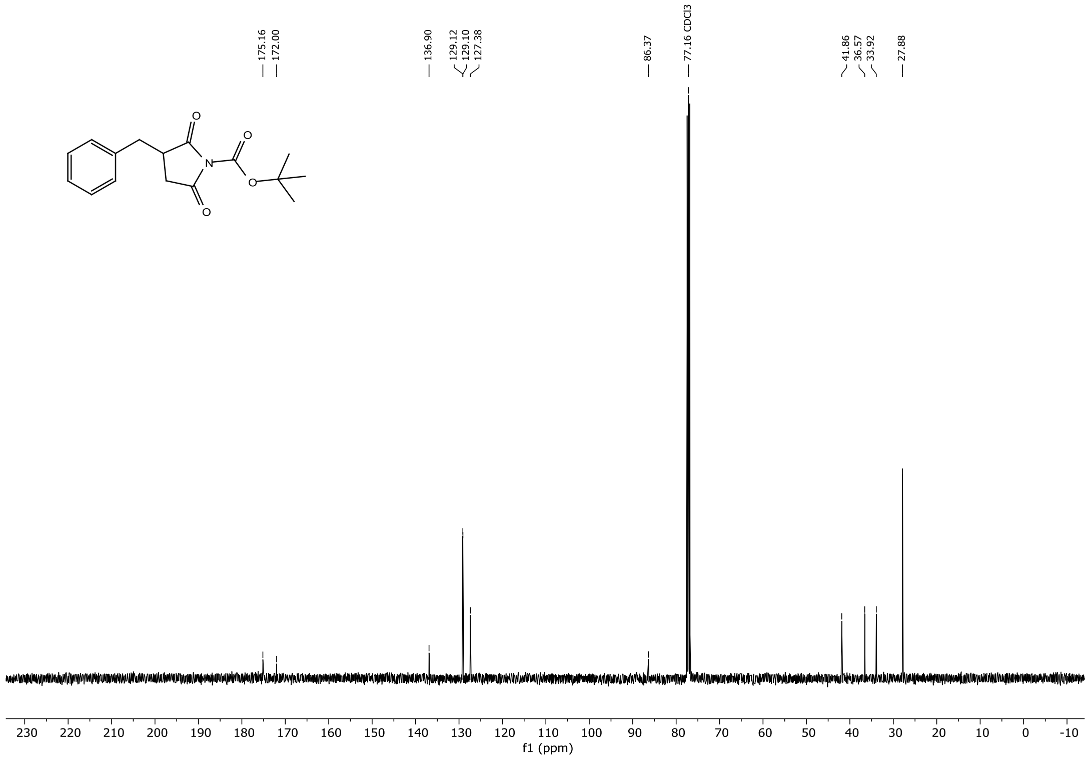

***Supplementary Figure 104. $^{13}C$ NMR spectrum of tert-butyl 3-benzyl-2,5-dioxopyrrolidine-1-carboxylate (F4).***

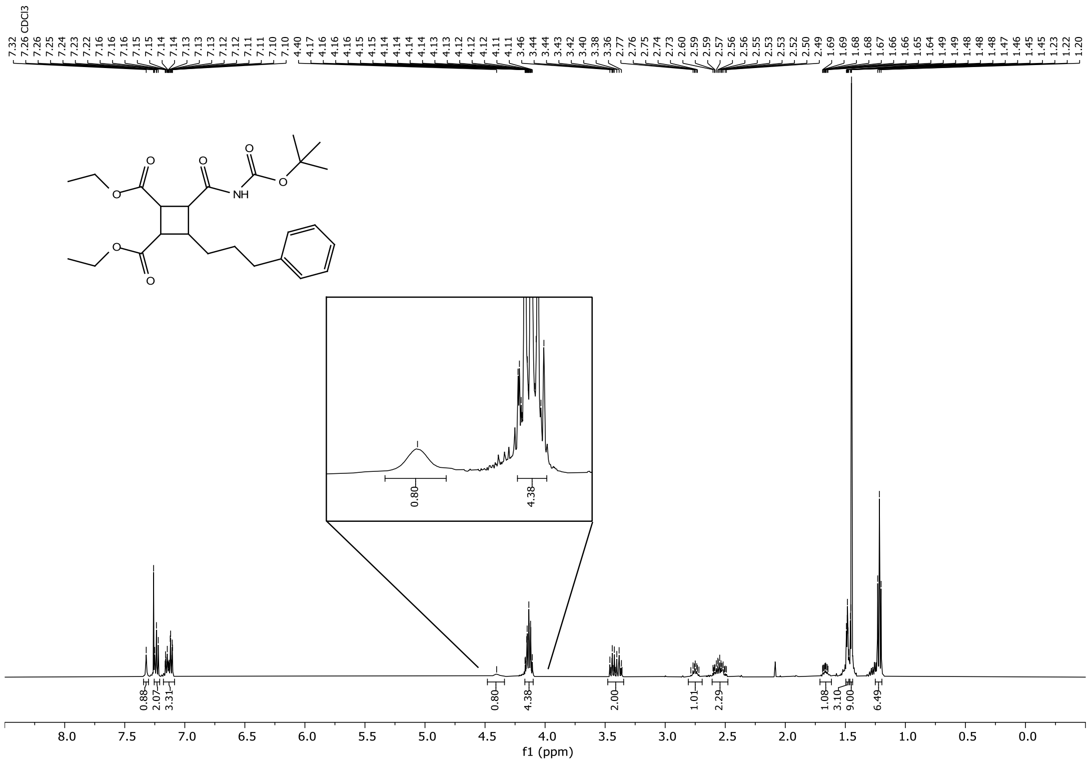

***Supplementary Figure 105. $^{1}H$ NMR spectrum of C18.***

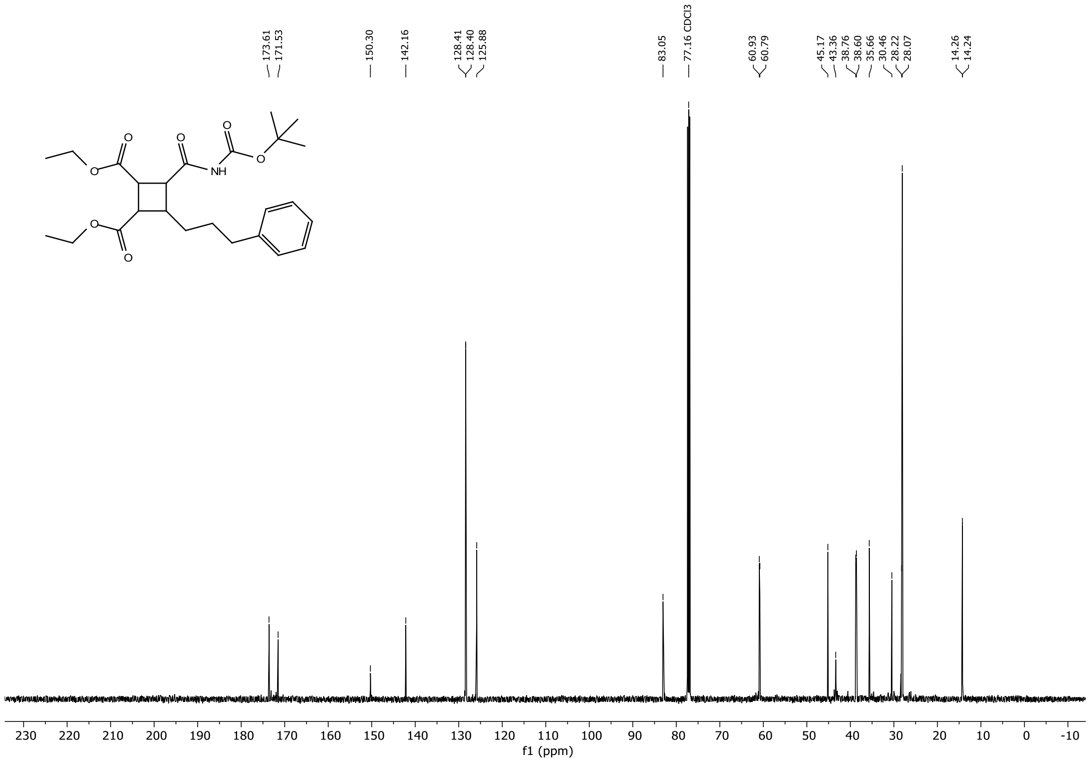


*Supplementary Figure 106.* ***$^{13}$C NMR spectrum of C18.***

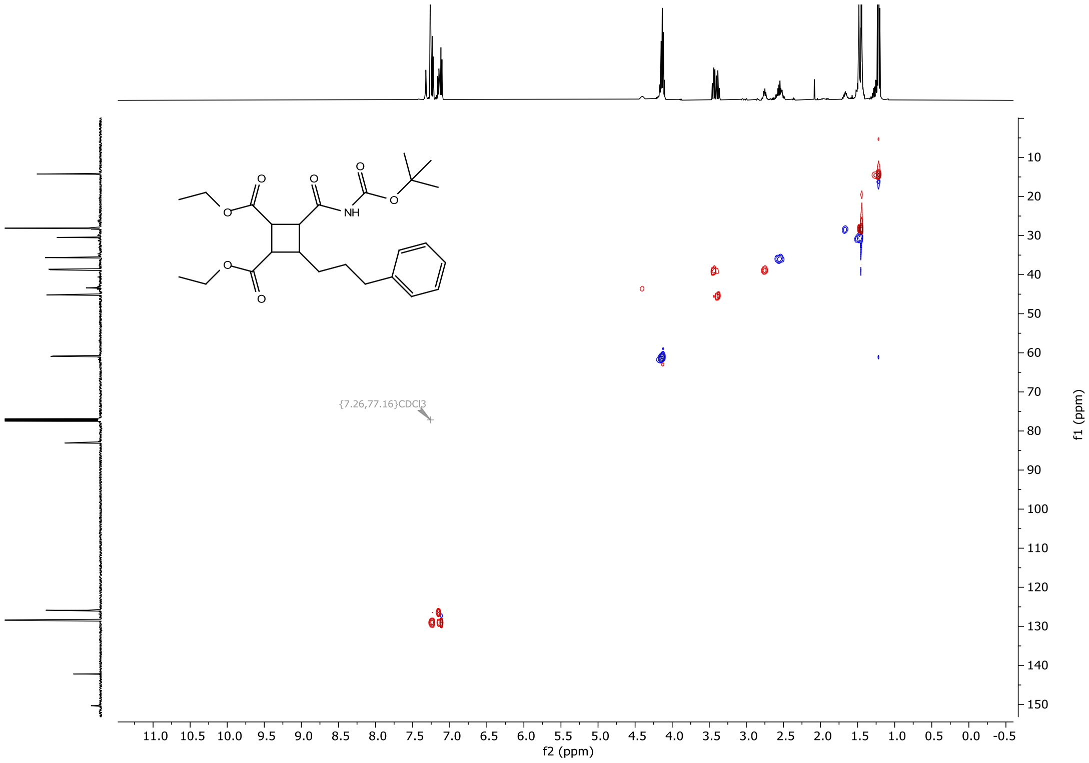


***Supplementary Figure 107. Phase edited HSQC NMR spectrum of C18.***

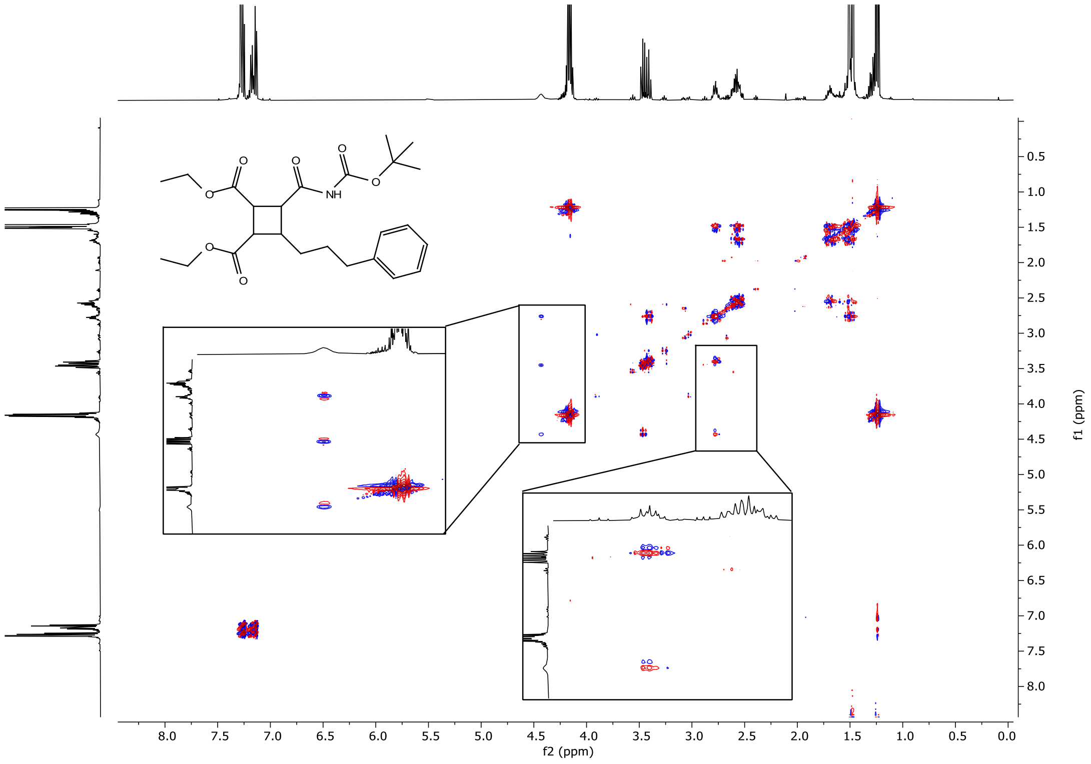


***Supplementary Figure 108. COSY NMR spectrum of C18.***

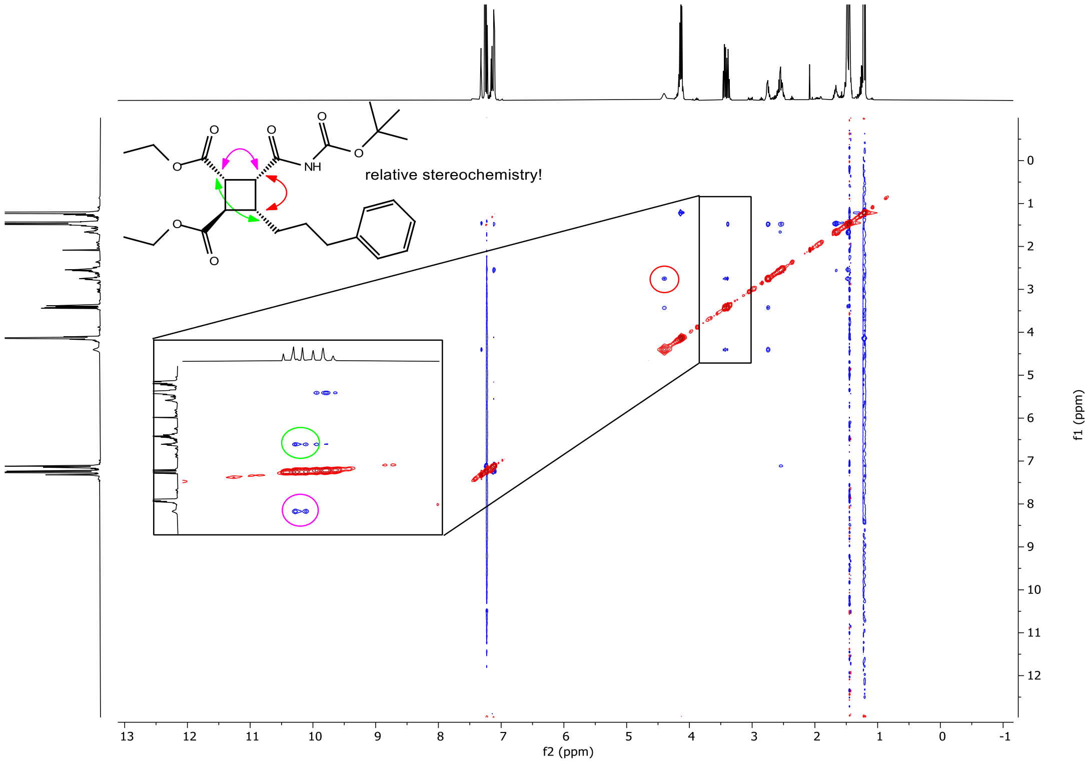


***Supplementary Figure 109. 2D-NOESY NMR spectrum of C18.***

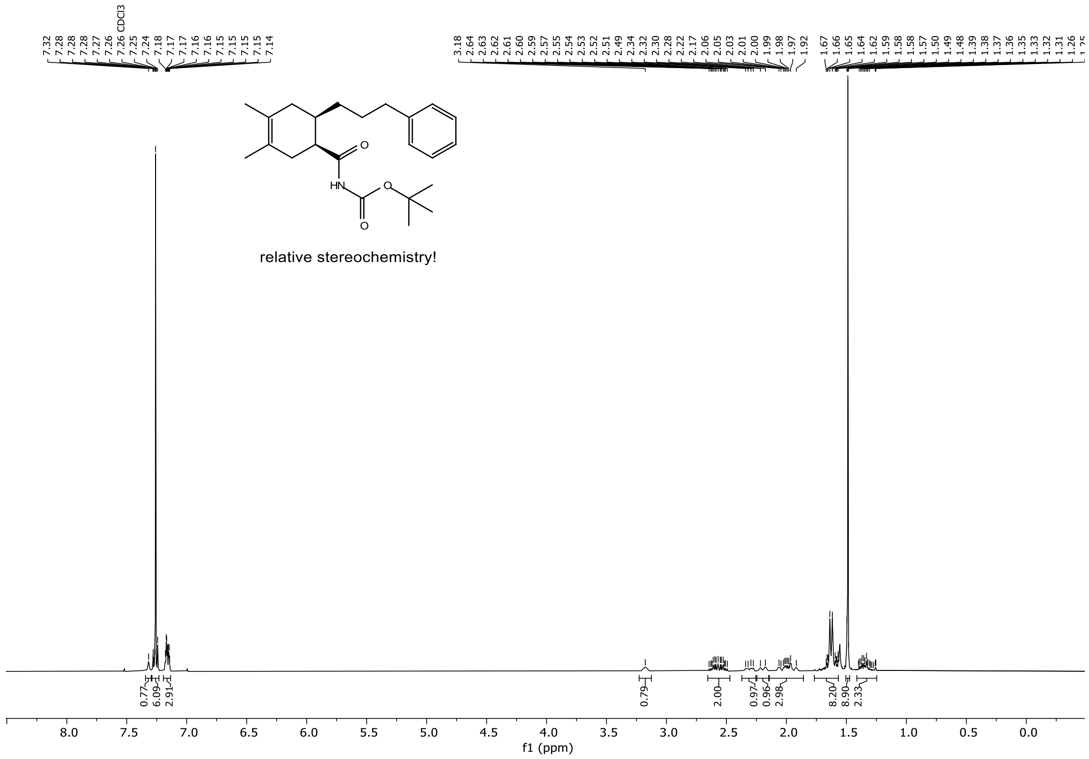


***Supplementary Figure 110. $^{1}$H NMR spectrum of C19.***

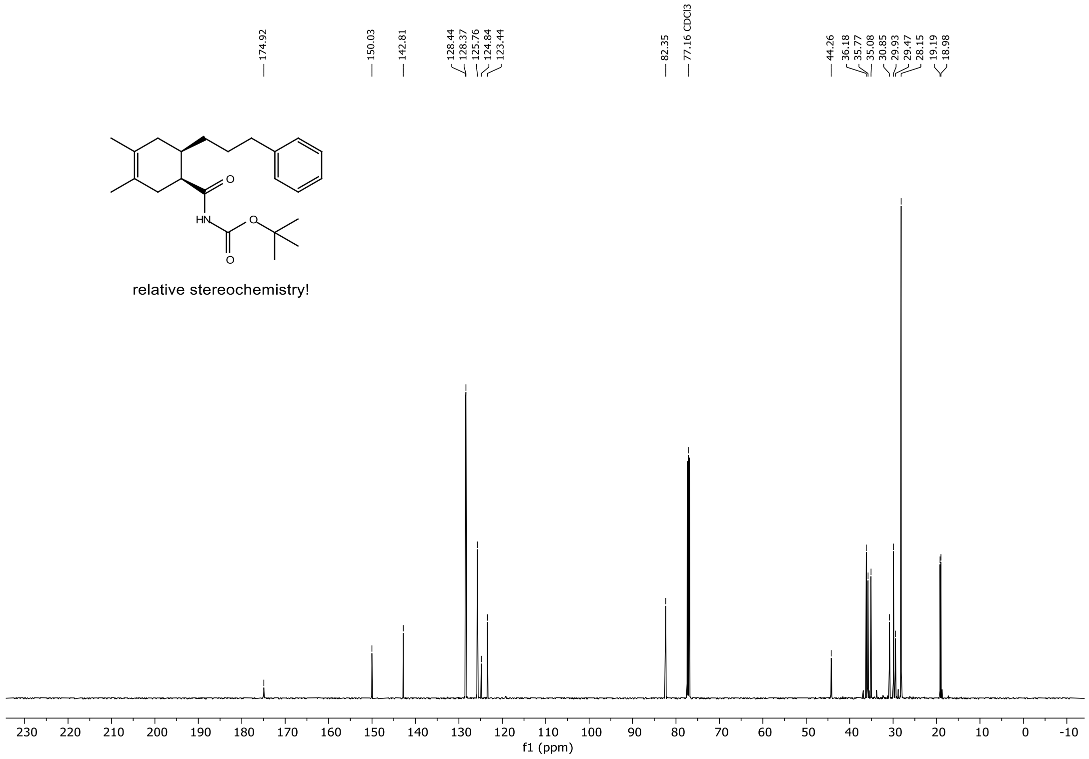


***Supplementary Figure 111. $^{13}$C NMR spectrum of C19.***

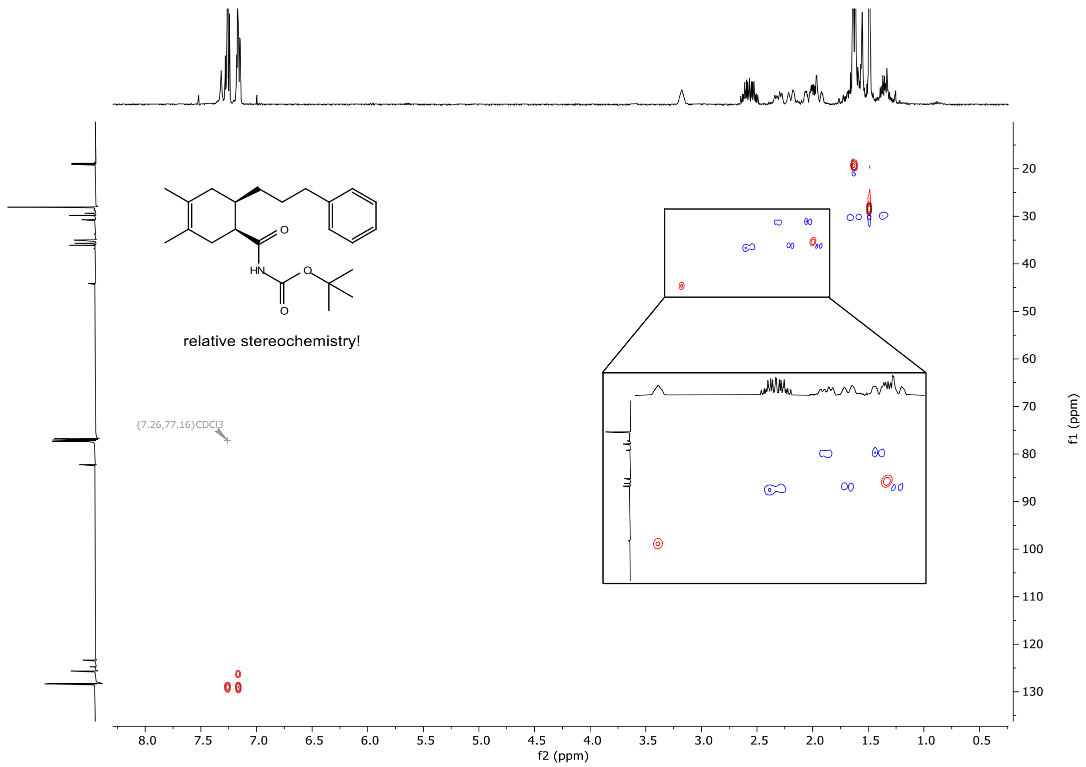


***Supplementary Figure 112. Phase edited HSQC NMR spectrum of C19.***

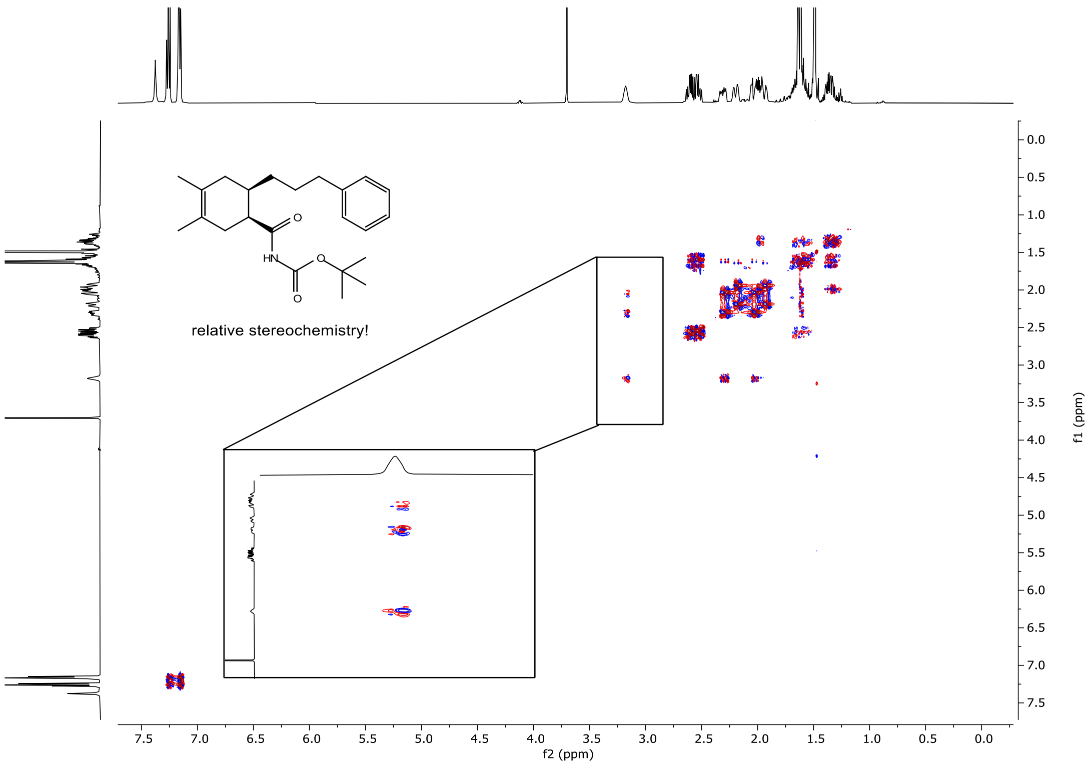


***Supplementary Figure 113. COSY NMR spectrum of C19.***

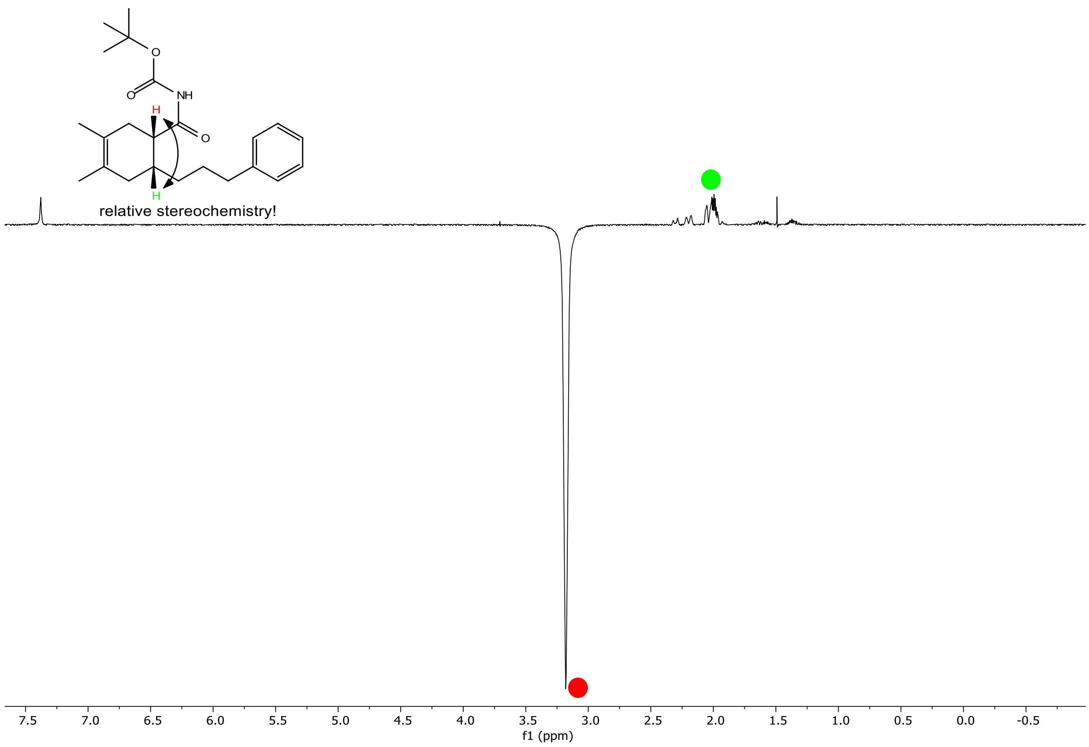


***Supplementary Figure 114. 1D-NOESY NMR spectrum of C19.***

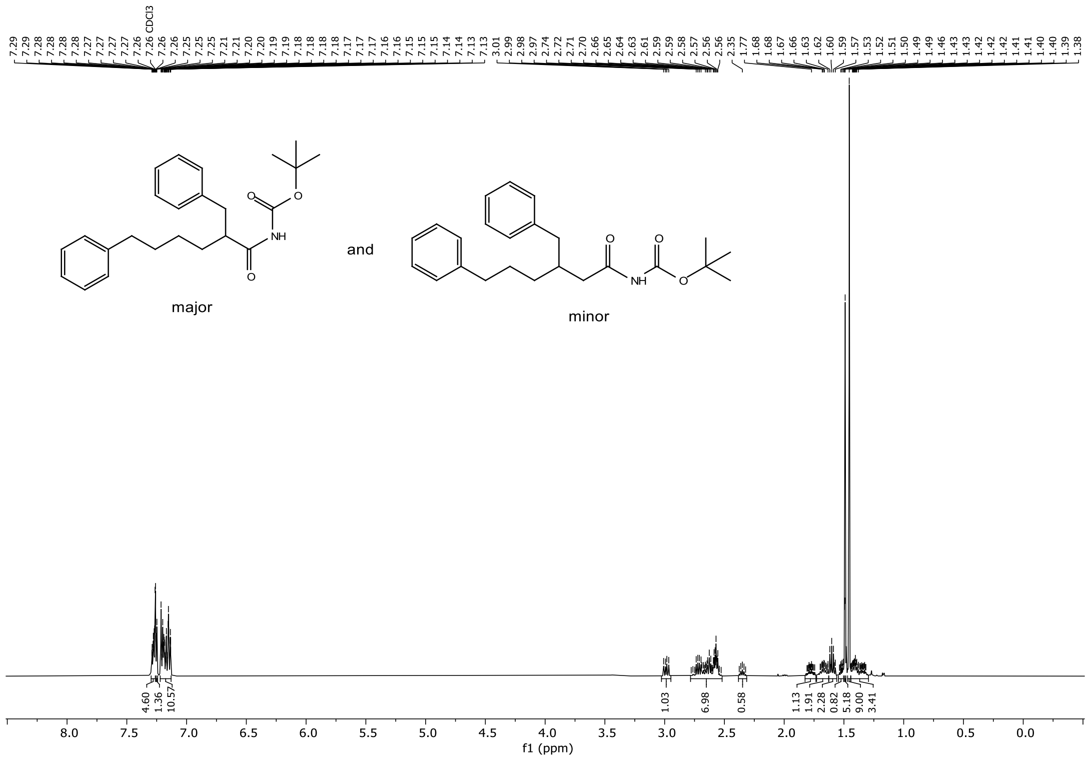


***Supplementary Figure 115. $^{1}$H NMR spectrum of regiosisomeric mixture of C20.***

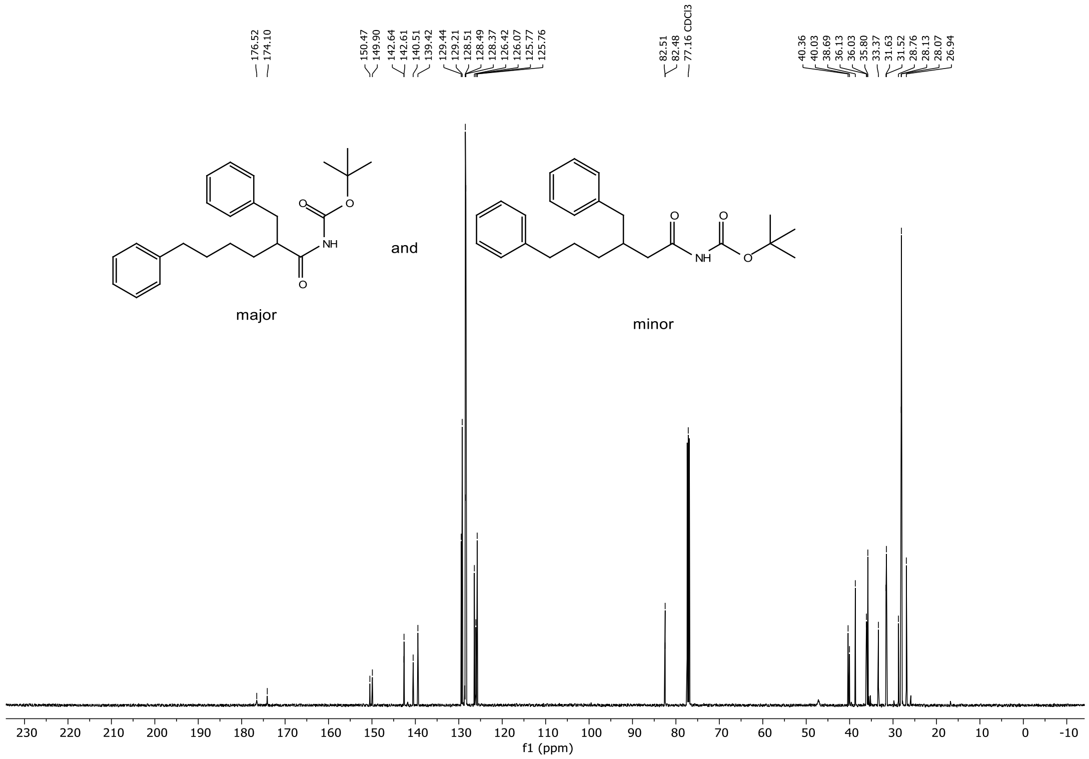


***Supplementary Figure 116. ¹³C NMR spectrum of regiosisomeric mixture of C20.***

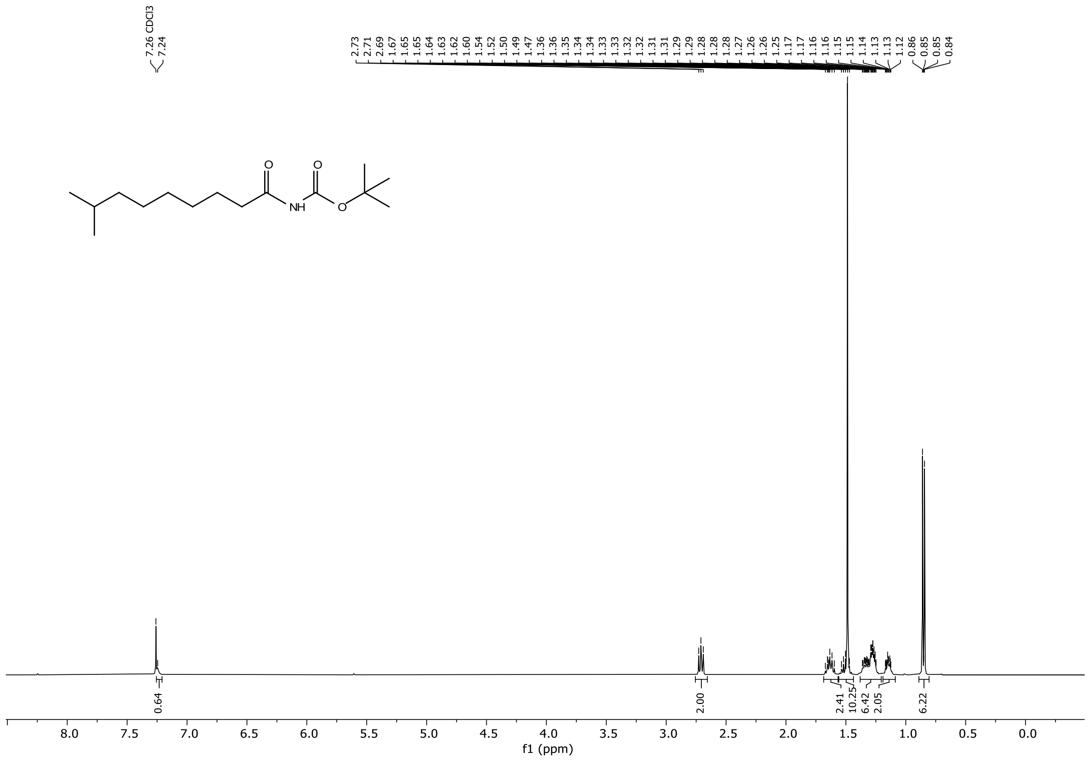


***Supplementary Figure 117. ¹H NMR spectrum of C21.***

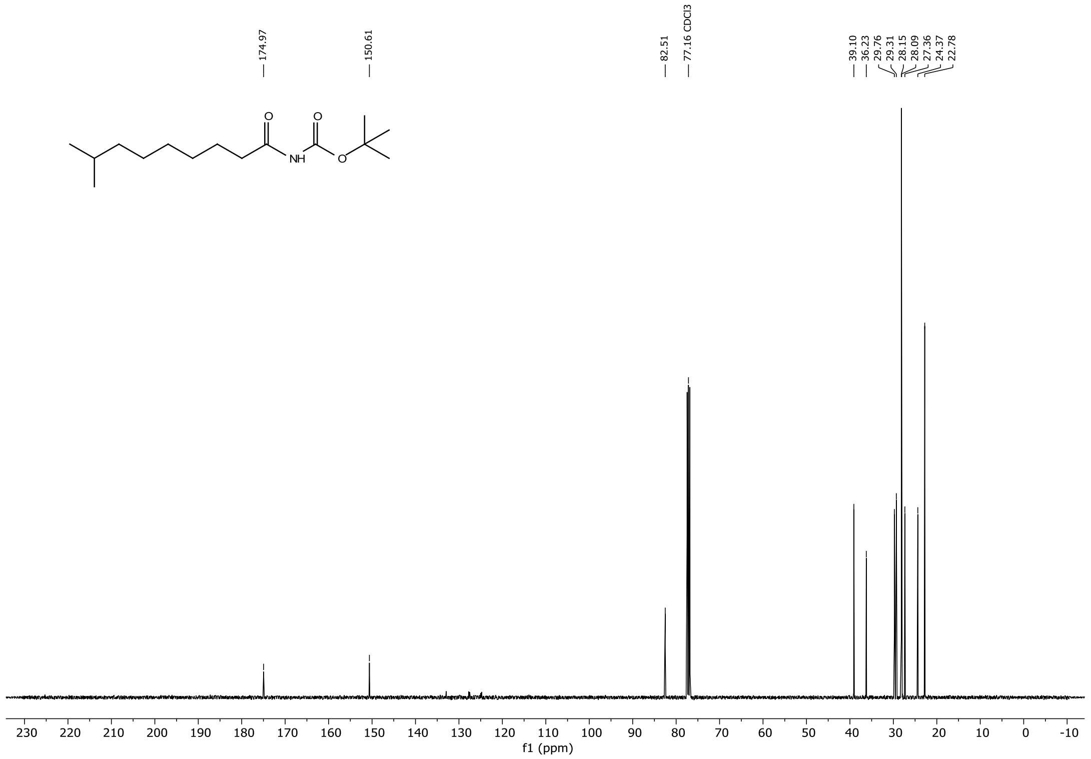

***Supplementary Figure 118. ^13^C NMR spectrum of C21.***

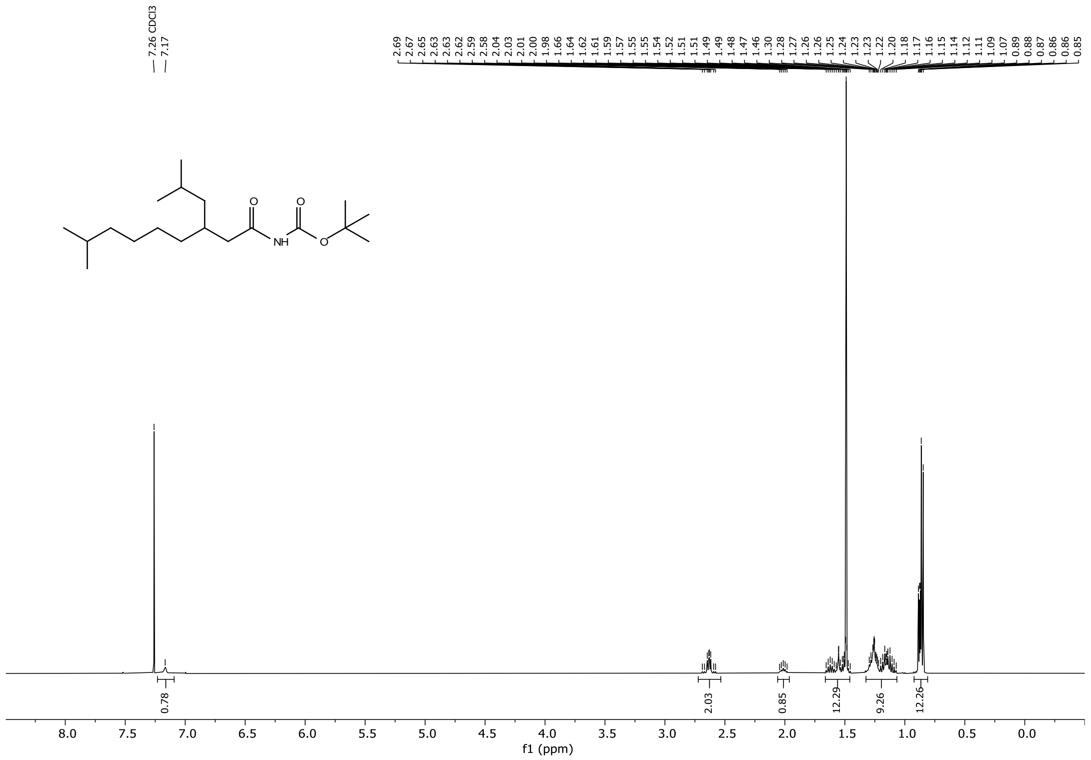

***Supplementary Figure 119. ^1^H NMR spectrum of E6.***

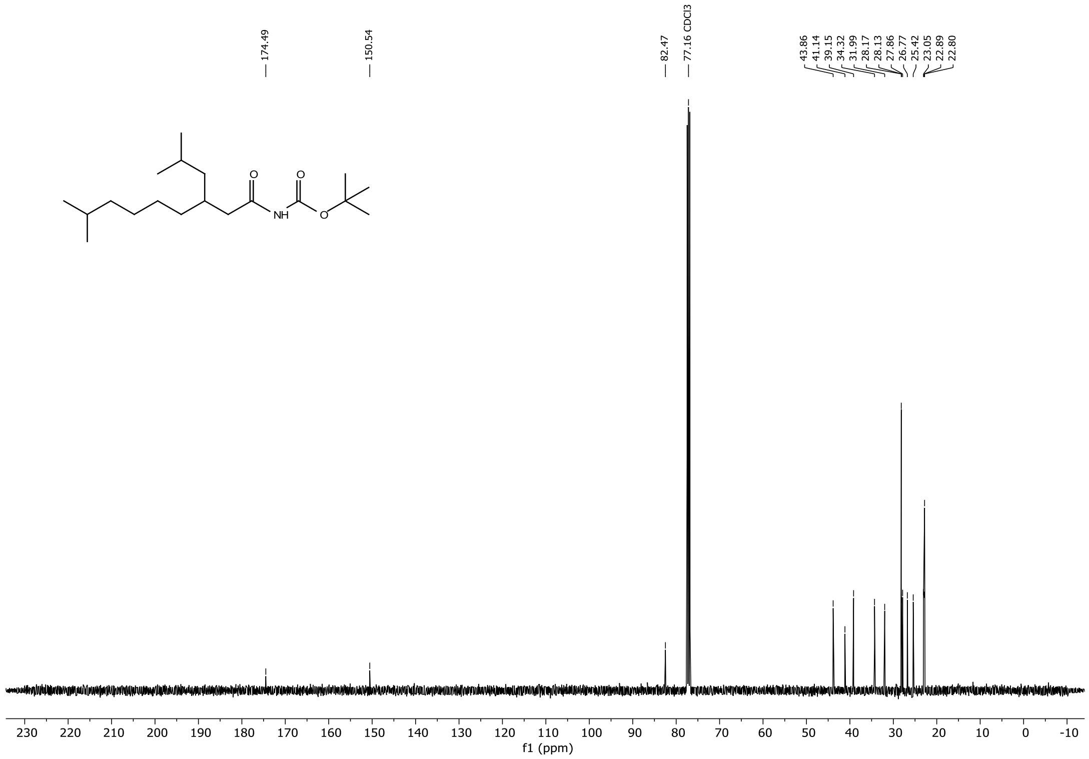

***Supplementary Figure 120. $^{13}C$ NMR spectrum of E6.***

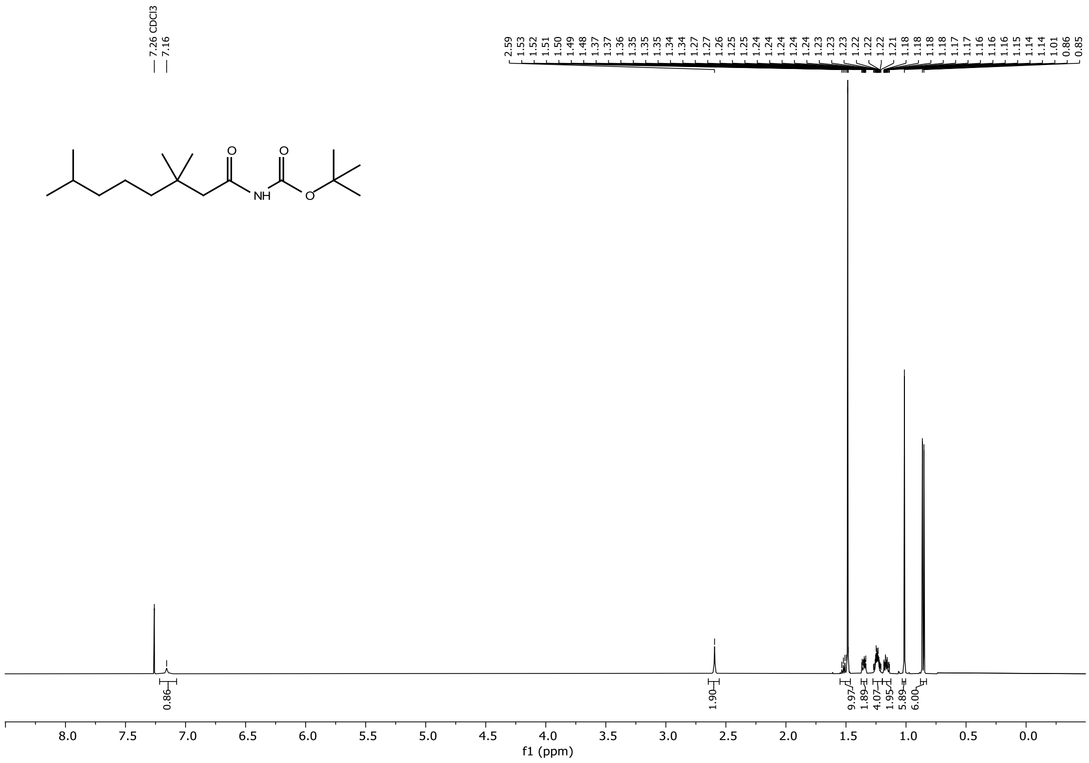

***Supplementary Figure 121. $^{1}H$ NMR spectrum of E7.***

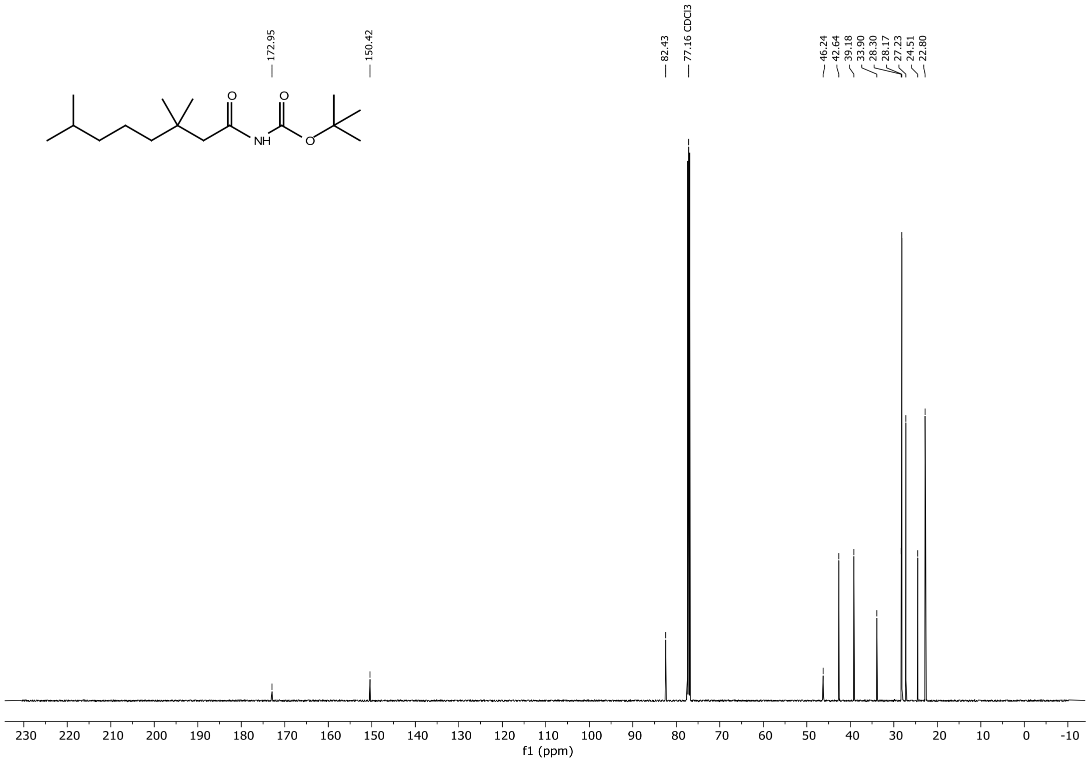


***Supplementary Figure 122. $^{13}$C NMR spectrum of E7.***

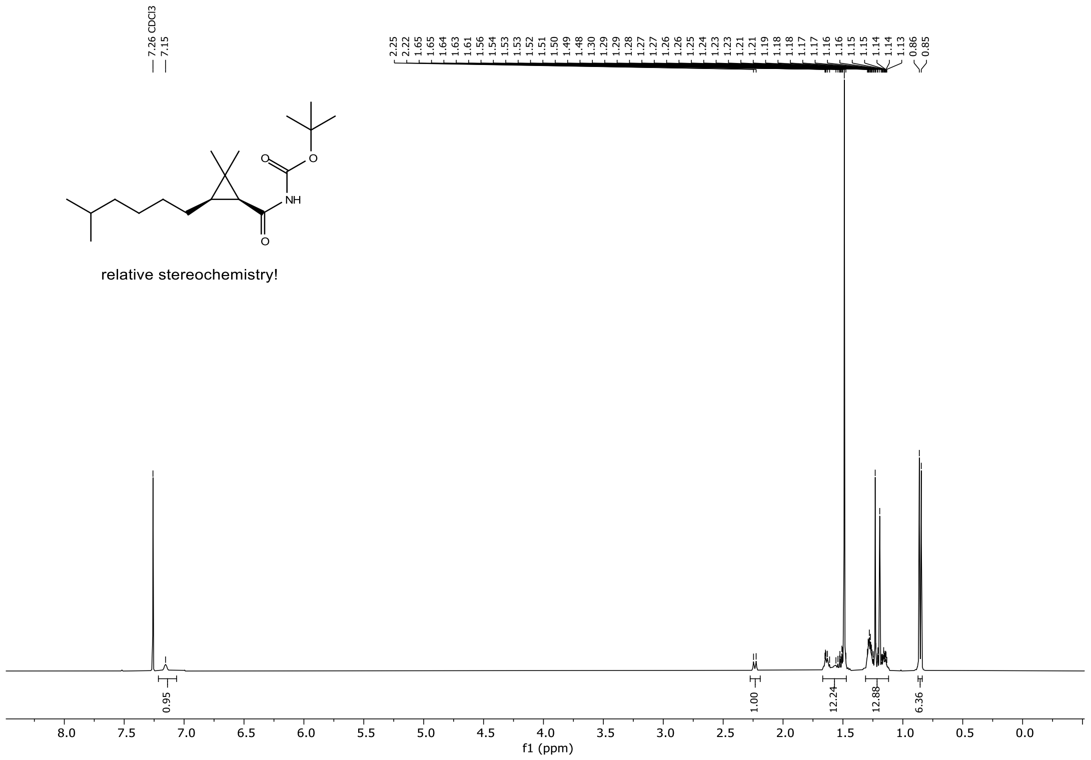


***Supplementary Figure 123. $^{1}$H NMR spectrum of C22.***

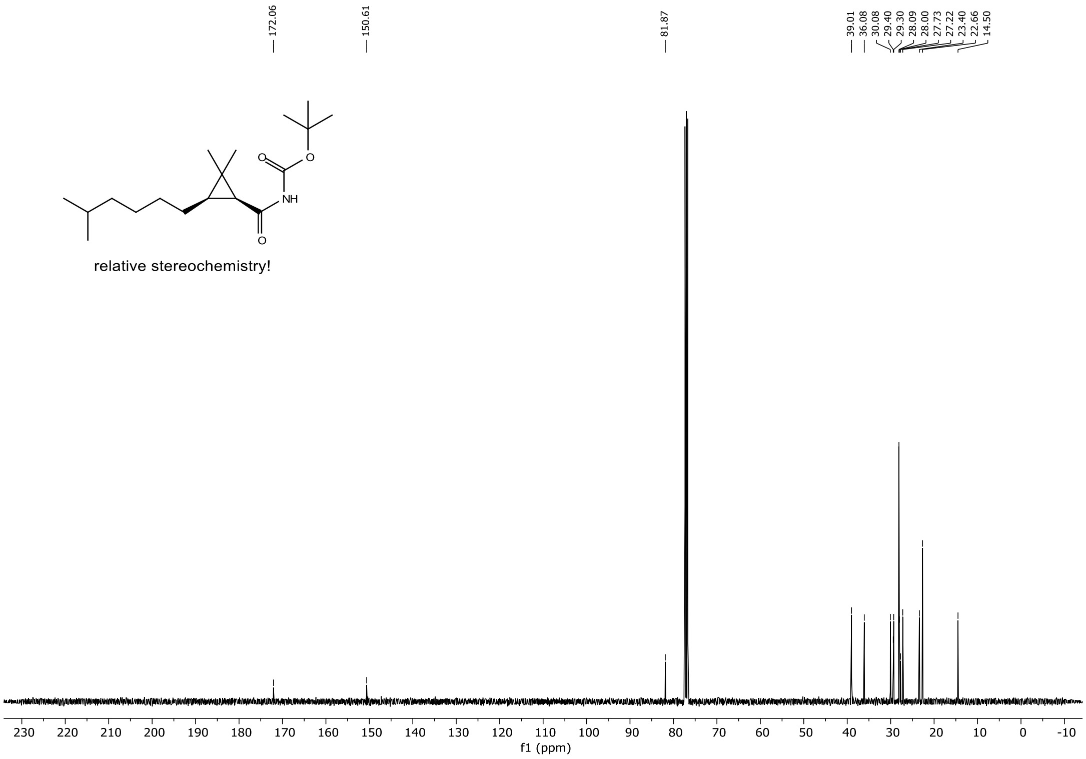


***Supplementary Figure 124. $^{13}$C NMR spectrum of C22.***

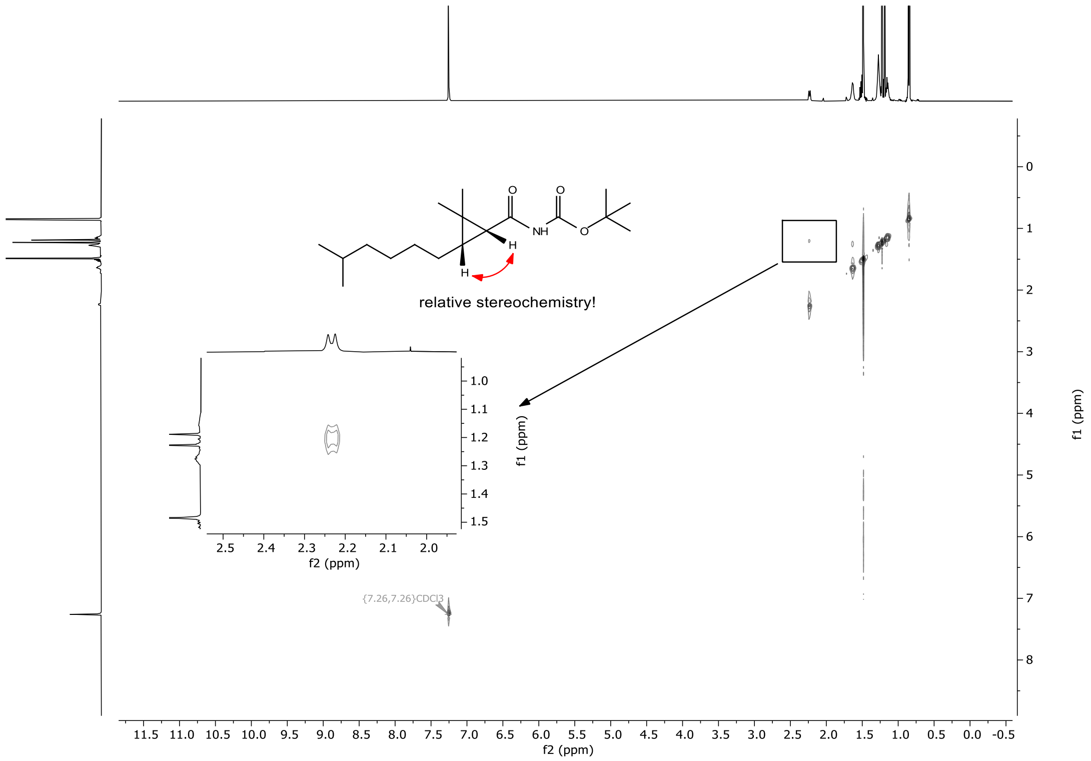


***Supplementary Figure 125. 2D-NOESY NMR spectrum of C22.***

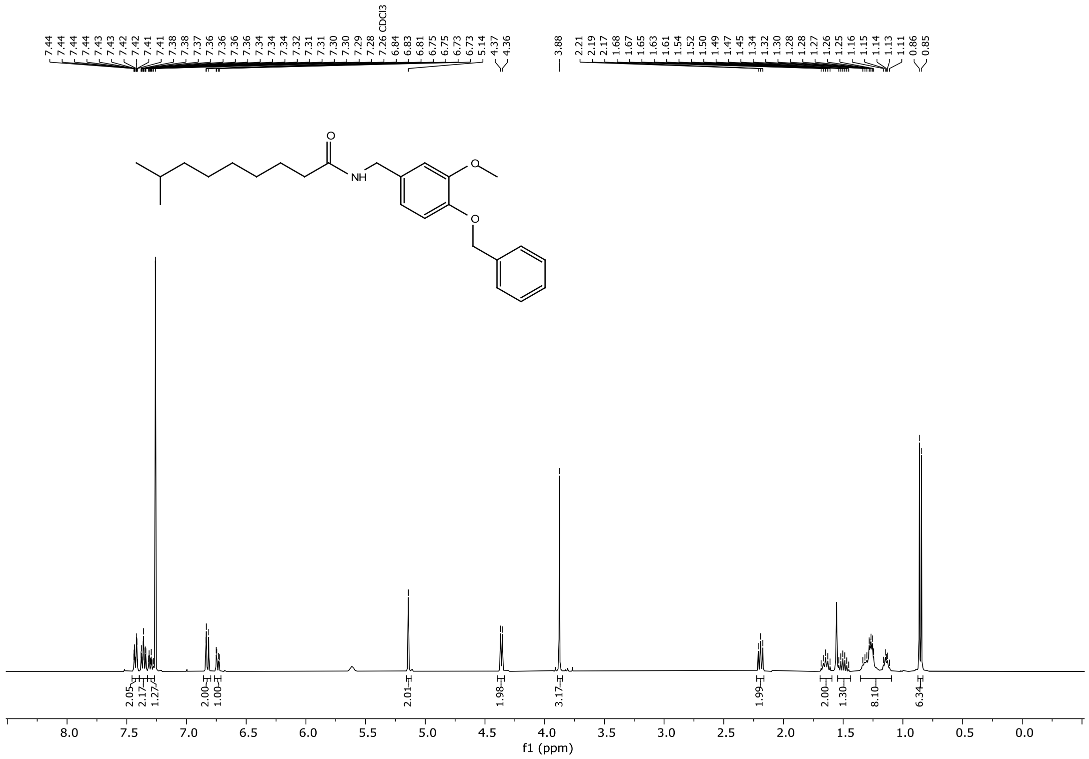

***Supplementary Figure 126. ¹H NMR spectrum of OBn-Dihydrocapsaicin (C21).***

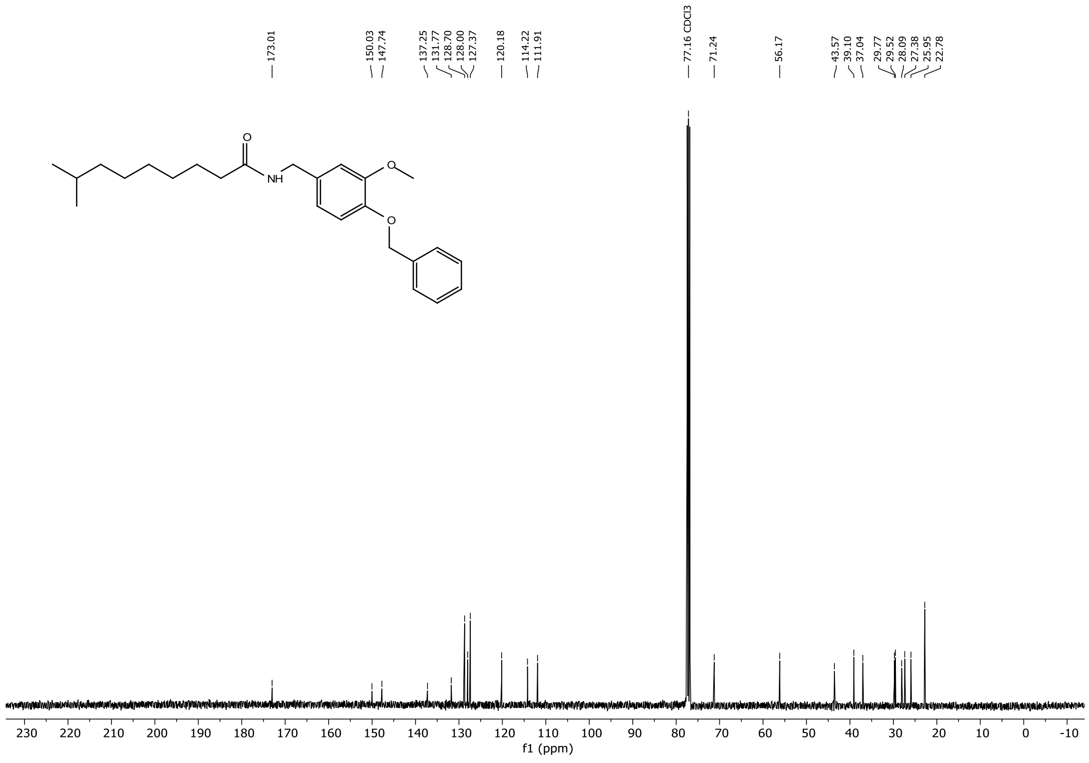

***Supplementary Figure 127. ¹³C NMR spectrum of OBn-Dihydrocapsaicin (C21).***

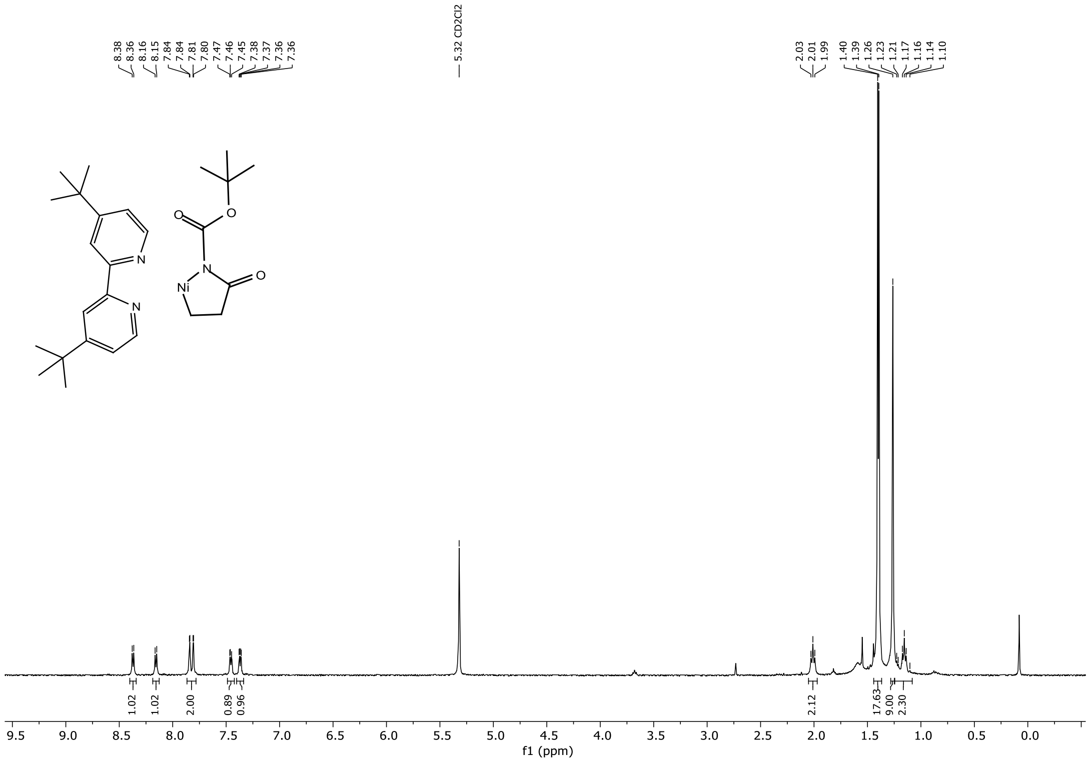


***Supplementary Figure 128. $^{1}$H NMR spectrum of Ni-1.***

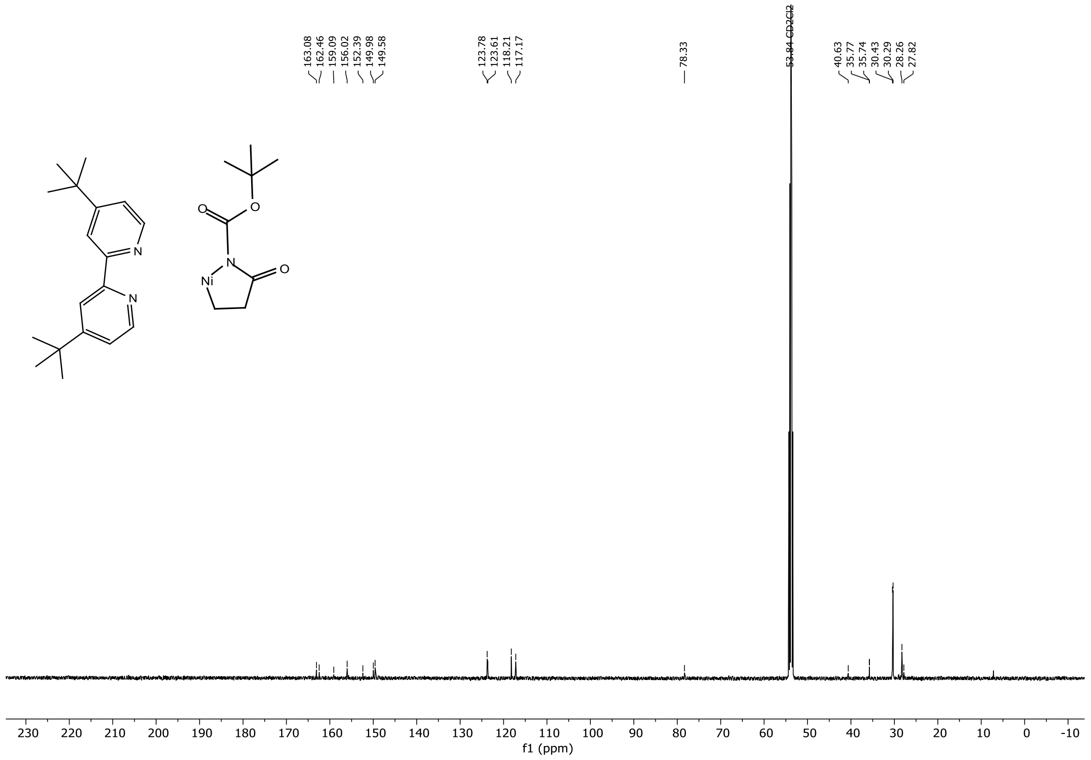


***Supplementary Figure 129. $^{13}$C NMR spectrum of Ni-1.***

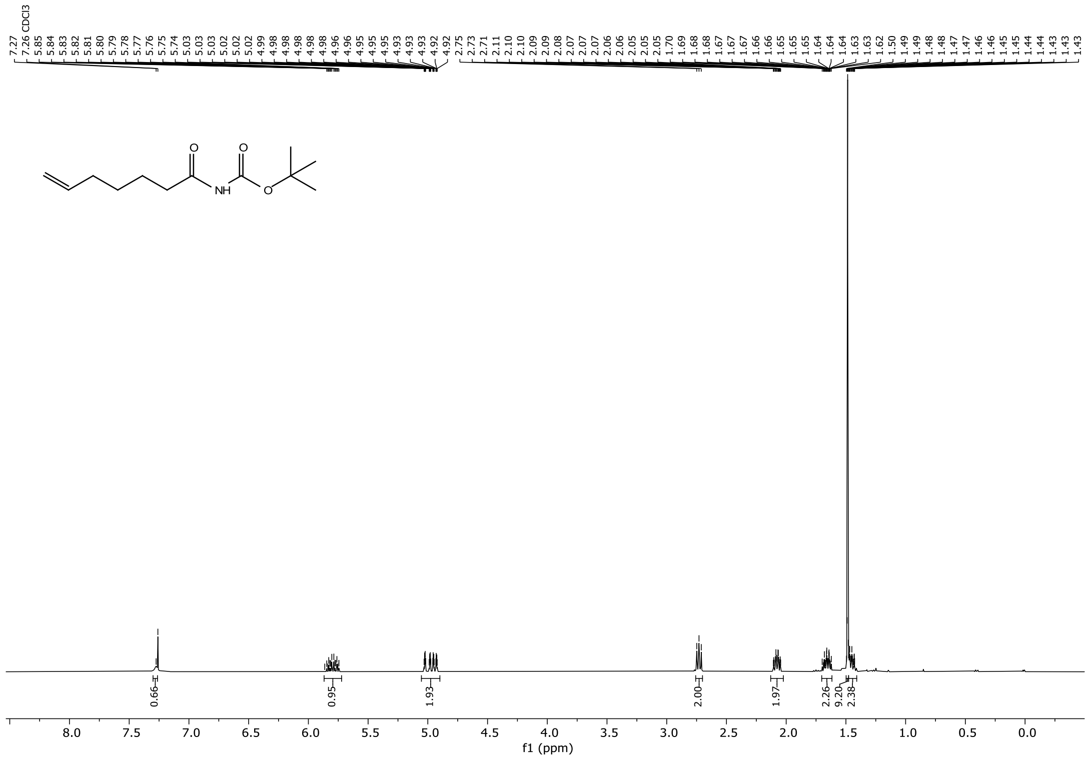


***Supplementary Figure 130. ¹H NMR spectrum of C23.***

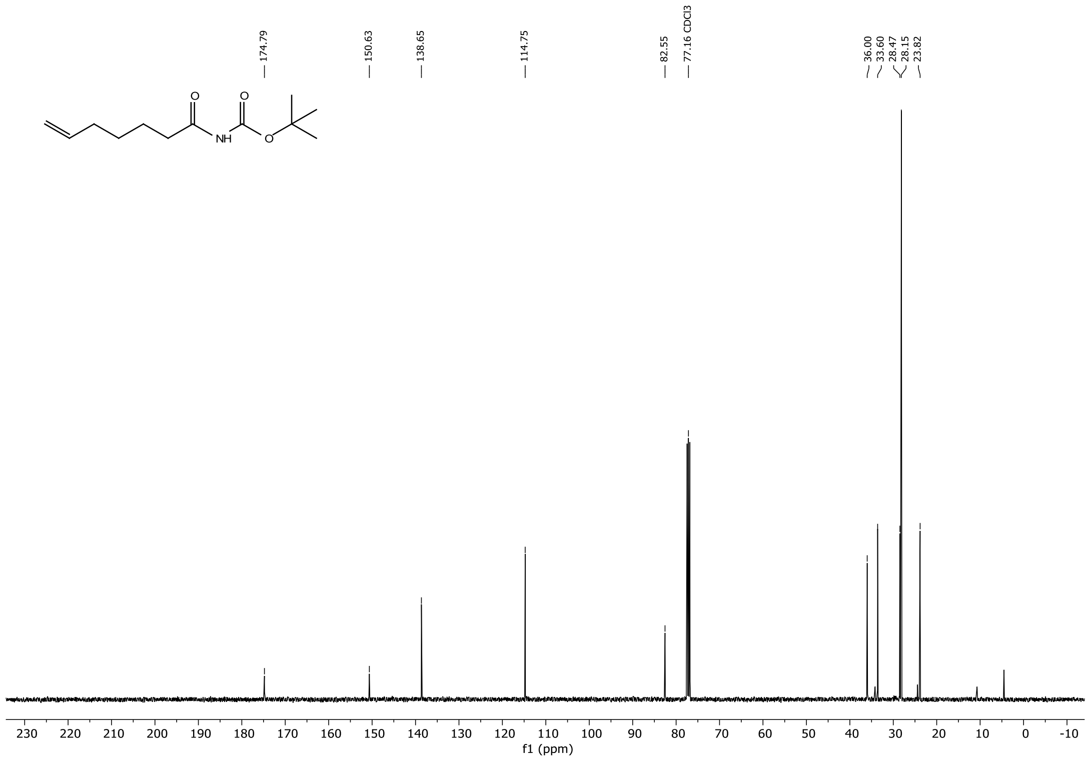


***Supplementary Figure 131. ¹³C NMR spectrum of C23.***

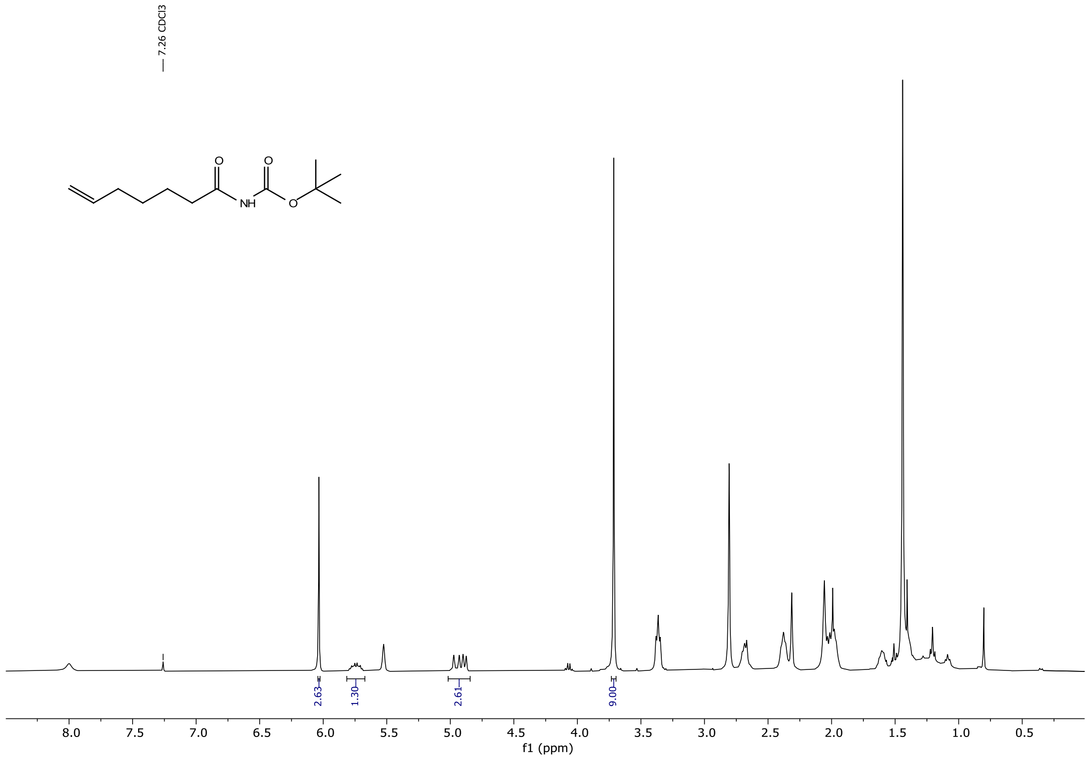


***Supplementary Figure 132. Crude ¹H NMR spectrum of C23 with 1,3,5-trimethoxybenzene as internal standard for yield determination.***

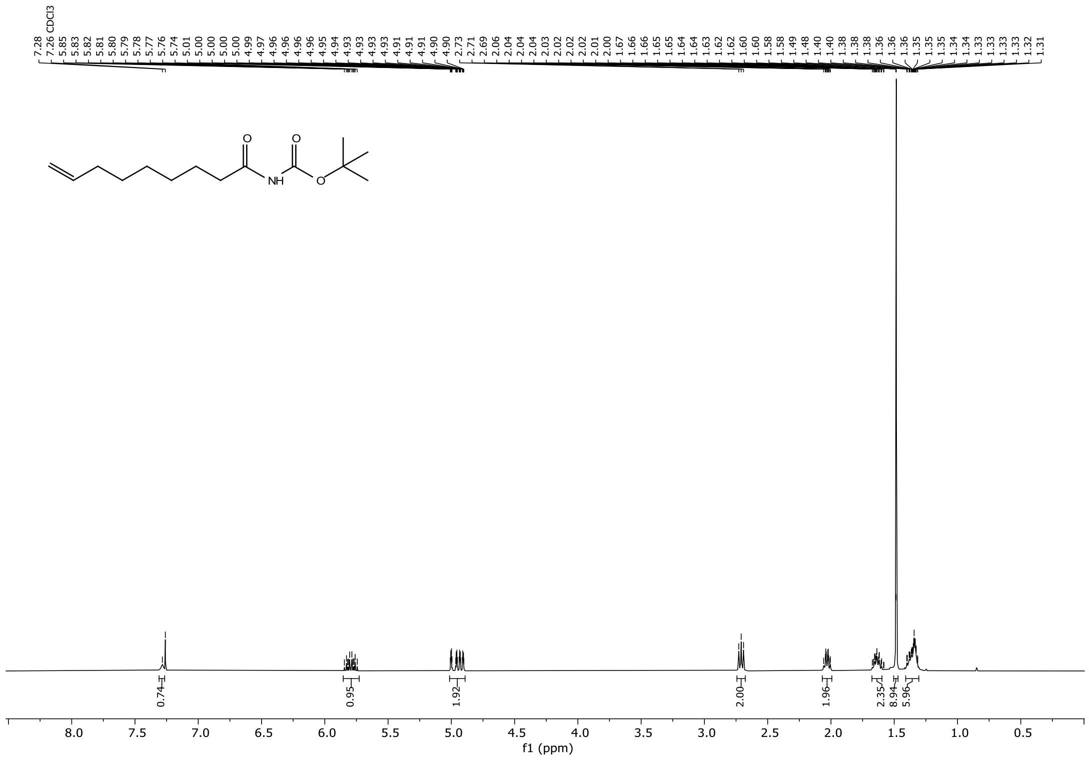


***Supplementary Figure 133. ¹H NMR spectrum of C22.***

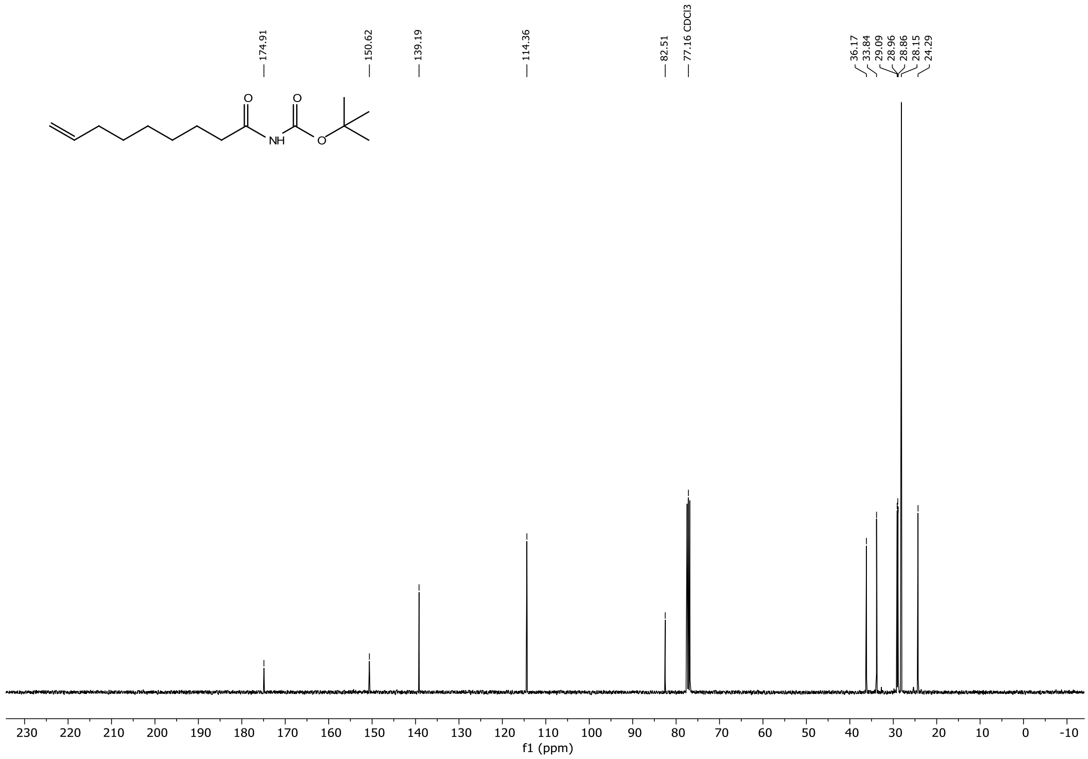


***Supplementary Figure 134. $^{13}$C NMR spectrum of C22.***

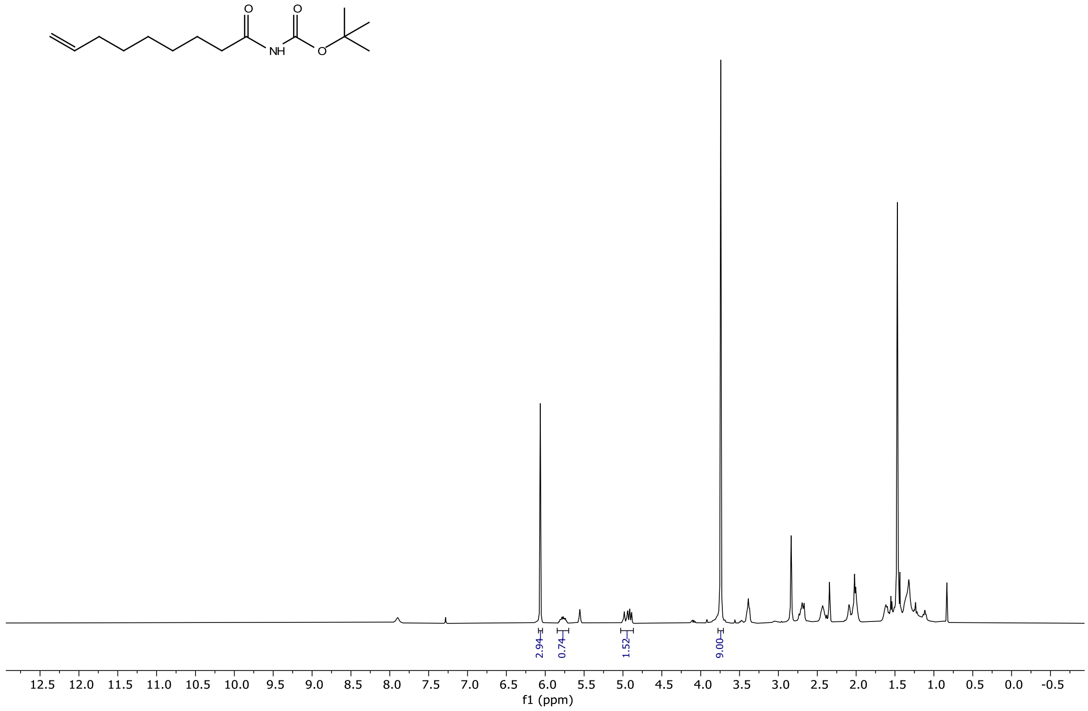


***Supplementary Figure 135. Crude $^{1}$H NMR spectrum of C22 with 1,3,5-trimethoxybenzene as internal standard for yield determination.***

# 8. XRD Data

## 8.1 X-ray structure parameters of nickel complexes

***Supplementary table 8. X-ray structure parameters of Ni-1 and Ni-2.***

| | **Ni-1** | **Ni-2** |
|---|---|---|
| CCDC # | 2495713 | 2495712 |
| Empirical formula | $C_{26}H_{37}N_3NiO_3$ | $C_{20}H_{24}N_2NiO_2$ |
| Formula weight | 498.29 | 383.12 |
| Temperature/K | 100.0(1) | 100.0(1) |
| Crystal system | monoclinic | orthorhombic |
| Space group | $P2_1/c$ | Fdd2 |
| a/Å | 10.3948(2) | 22.0314(3) |
| b/Å | 12.1968(2) | 20.8413(3) |
| c/Å | 20.7449(4) | 8.44160(10) |
| α/° | 90 | 90 |
| β/° | 99.606(2) | 90 |
| γ/° | 90 | 90 |
| Volume/Å$^3$ | 2593.23(8) | 3876.07(9) |
| Z | 4 | 8 |
| $\rho_{calc}$g/cm$^3$ | 1.276 | 1.313 |
| μ/mm$^{-1}$ | 1.319 | 1.554 |
| F(000) | 1064.0 | 1616.0 |
| Crystal size/mm$^3$ | 0.14 × 0.046 × 0.024 | 0.278 × 0.061 × 0.038 |
| Crystal colour | Clear orange | Clear red |
| Radiation | Cu Kα (λ = 1.54184) | Cu Kα (λ = 1.54184) |
| 2Θ range /° | 8.44 to 149.138 | 11.69 to 148.896 |
| Index ranges | -12 ≤ h ≤ 12<br>-12 ≤ k ≤ 14<br>-25 ≤ l ≤ 24 | -25 ≤ h ≤ 27<br>-25 ≤ k ≤ 25<br>-8 ≤ l ≤ 9 |
| Reflections collected | 22020 | 15704 |
| Independent reflections | 5043<br>$R_{int}$ = 0.0498<br>$R_{sigma}$ = 0.0406 | 1832<br>$R_{int}$ = 0.0454,<br>$R_{sigma}$ = 0.0240 |
| Data/restraints/parameters | 5043/0/307 | 1832/1/117 |
| Goodness-of-fit on F$^2$ | 1.023 | 1.109 |
| Final R indexes [I>=2σ (I)] | $R_1$ = 0.0446, $wR_2$ = 0.1139 | $R_1$ = 0.0337, $wR_2$ = 0.0889 |
| Final R indexes [all data] | $R_1$ = 0.0614, $wR_2$ = 0.1234 | $R_1$ = 0.0358, $wR_2$ = 0.0906 |
| Largest diff. peak/hole / e Å$^{-3}$ | 0.78/-0.44 | 0.34/-0.32 |
| Flack parameter | - | -0.01(5) |

**8.2 ORTEP plots of X-ray structures and solid-state parameters**

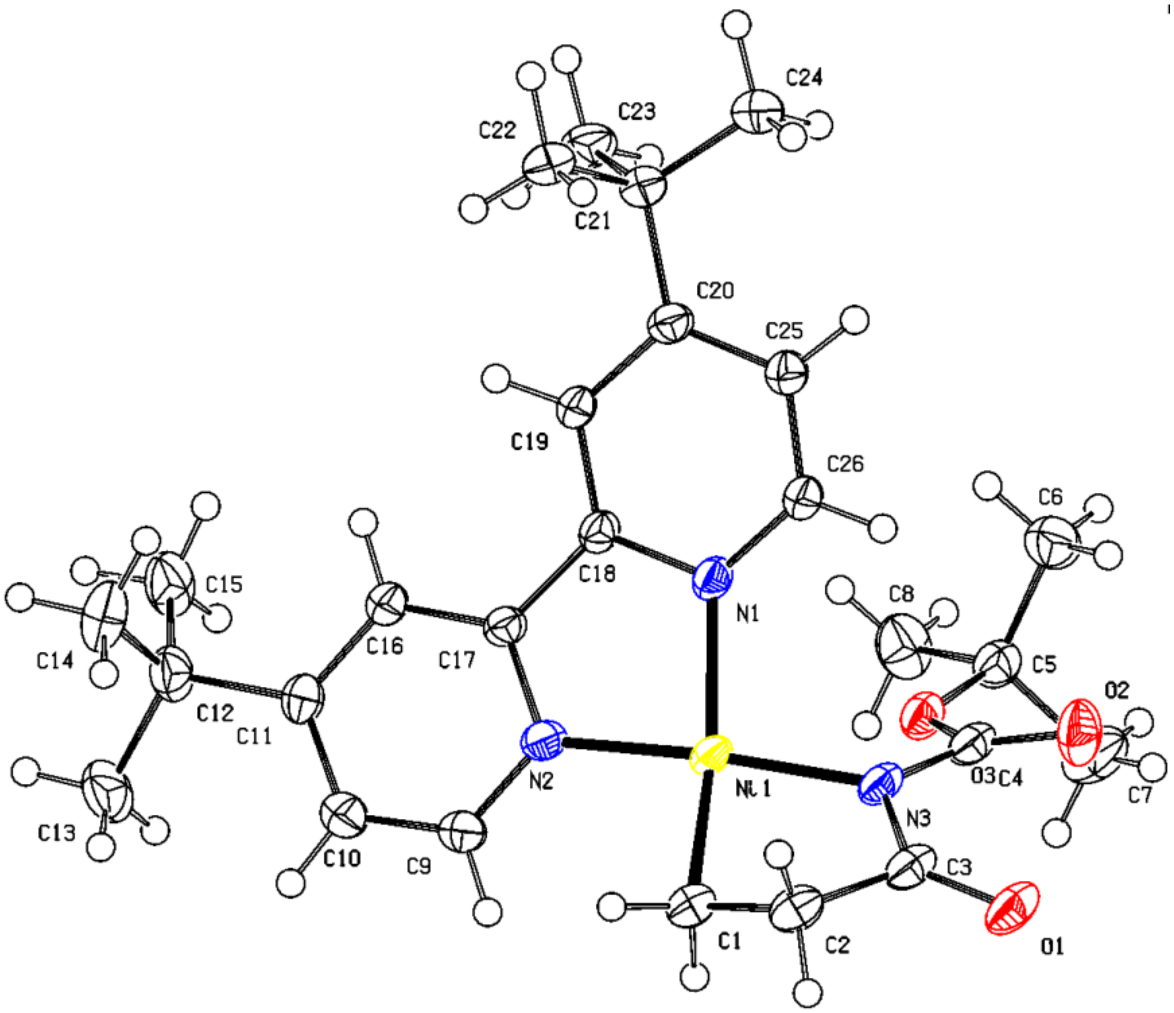


***Supplementary Figure 136. ORTEP plot of Ni-1 in 50% thermal ellipsoids. Torsion angle ∠C4–N3–Ni1–C1: 28.3(1)°. Bond angles ∠C1–Ni1–N3: 84.14(9)°, ∠N1–Ni1–N3: 100.63(8)°. Bond distances (Å) Ni1–C1: 1.925(2), Ni1–N3: 1.9009(19), Ni1–N1: 1.9185(19), Ni1–N2: 1.8962(18), C3–O1: 1.230(3), N3–C4: 1.380(3).***

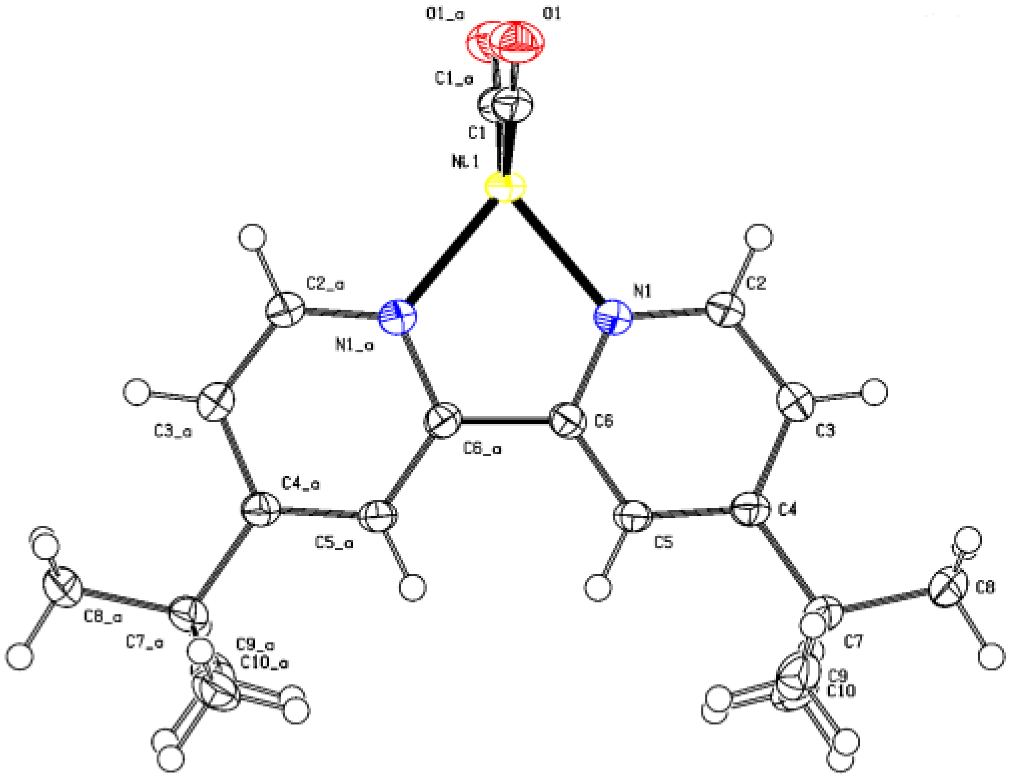


***Supplementary Figure 137. ORTEP plot of Ni-2 in 50% thermal ellipsoids. The molecule sits on a two-fold axis. Bond angles ∠C1-Ni1-N1: 115.20(13)°, ∠N1–Ni1–N1α: 79.410°, ∠C1-Ni1-C1α: 112.640°. Bond distances (Å) Ni1–C1: 1.753(4), Ni1-N1: 2.022(3), C1-O1: 1.161(4).***

## 9. References

References 1-52 are cited within the article.


53 CrysAlisPro and ABSPACK. https://www.rigaku.com/products/crystallography/crysalis.

54 G. M. Sheldrick, "*SHELXT* – Integrated space-group and crystal-structure determination" *Acta Crystallogr. Sect. Found. Adv.* **2015**, *71*, 3–8.

55 G. M. Sheldrick, "A short history of *SHELX*" *Acta Crystallogr. A* **2008**, *64*, 112–122.

56 G. M. Sheldrick, "Crystal structure refinement with *SHELXL*" *Acta Crystallogr. Sect. C Struct. Chem.* **2015**, *71*, 3–8.

57 O. V. Dolomanov, L. J. Bourhis, R. J. Gildea, J. A. K. Howard, H. Puschmann, "*OLEX2* : a complete structure solution, refinement and analysis program" *J. Appl. Crystallogr.* **2009**, *42*, 339–341.

58 A. Guzmán, M. Romero, J. M. Muchowski, "Vilsmeier–Haack reaction with succinamidals. A convenient synthesis of 5-chloropyrrole-2-carboxaldehydes and 5-chloropyrrole-2,4-dicarboxaldehydes" *Can. J. Chem.* **1990**, *68*, 791–794.

59 C. H. Schiwek, S. Stegbauer, T. Pickl, T. Bach, "Rhodium(CAAC)-Catalyzed Arene Hydrogenation of Benzo-Fused *N* -Heterocycles to Saturated Building Blocks with an all- *cis* Configuration" *Adv. Synth. Catal.* **2022**, *364*, 3360–3365.

60 S. Rybka, J. Obniska, A. Rapacz, B. Filipek, K. Kamiński, "Synthesis, Physicochemical, and Anticonvulsant Properties of New *N* -Mannich Bases Derived from Pyrrolidine-2,5-dione and Its 3-Methyl Analog" *Arch. Pharm. (Weinheim)* **2014**, *347*, 768–776.

61 Y. Kurimoto, T. Nasu, Y. Fujii, K. Asano, S. Matsubara, "Asymmetric Cycloetherification of in Situ Generated Cyanohydrins through the Concomitant Construction of Three Chiral Carbon Centers" *Org. Lett.* **2019**, *21*, 2156–2160.

62 H. Qu, X.-S. Liang, W.-J. Wang, X.-H. Zhao, Y.-H. Deng, X.-T. An, W.-D. Chu, X.-Z. Zhang, C.-A. Fan, "Catalytic Enantioselective Desymmetrization of Prochiral Triacylamines via Pseudopeptidic Guanidine–Guanidinium Catalysis" *Org. Lett.* **2022**, *24*, 6851–6856.

63 L. Pop, A. Czompa, C. Paizs, M. Toşa, E. Vass, P. Mátyus, F.-D. Irimie, "Lipase-Catalyzed Synthesis of Both Enantiomers of 3-Chloro-1-arylpropan-1-ols" *Synthesis* **2011**, *2011*, 2921–2928.

64 E. Badarau, S. Dilly, J. Wouters, V. Seutin, J.-F. Liégeois, "Chemical modifications of the N -methyl-laudanosine scaffold point to new directions for SK channels exploration" *Bioorg. Med. Chem. Lett.* **2014**, *24*, 5616–5620.

65 K. Sanada, H. Ube, M. Shionoya, "Rotational Control of a Dirhodium-Centered Supramolecular Four-Gear System by Ligand Exchange" *J. Am. Chem. Soc.* **2016**, *138*, 2945–2948.

66 M. Uygur, T. Danelzik, O. García Mancheño, "Metal-free desilylative C–C bond formation by visible-light photoredox catalysis" *Chem. Commun.* **2019**, *55*, 2980–2983.

67 T. Kerackian, A. Reina, T. Krachko, H. Boddaert, D. Bouyssi, N. Monteiro, A. Amgoune, "C(sp3)–H Bond Acylation with N-Acyl Imides under Photoredox/ Nickel Dual Catalysis" *Synlett* **2021**, *32*, 1531–1536.

68 V. W. Bowry, J. Lusztyk, K. U. Ingold, "Calibration of a new horology of fast radical clocks. Ring-opening rates for ring- and .alpha.-alkyl-substituted cyclopropylcarbinyl radicals and for the bicyclo[2.1.0]pent-2-yl radical" *J. Am. Chem. Soc.* **1991**, *113*, 5687–5698.

69 Y. Fu, R.-Q. Li, L. Liu, Q.-X. Guo, "Solvent effect is not significant for the speed of a radical clock" *Res. Chem. Intermed.* **2004**, *30*, 279–286.
